# Supplementary material for: A Humanized Yeast Phenomic Model of Deoxycytidine Kinase to Predict Genetic Buffering of Nucleoside Analog Cytotoxicity
Source: Genes (Basel). 2019 Sep 30;10(10):770. doi: 10.3390/genes10100770 (PMC6826991; doi:10.3390/genes10100770)
Supplement: Supplementary file 1 [file genes-10-00770-s001.zip › Additional_Files/Additional_File7_GOTermHeatmaps.zip!/Additional_File7_GOTermHeatmaps/regulation_of_transcription,_DNA-templated.pdf]

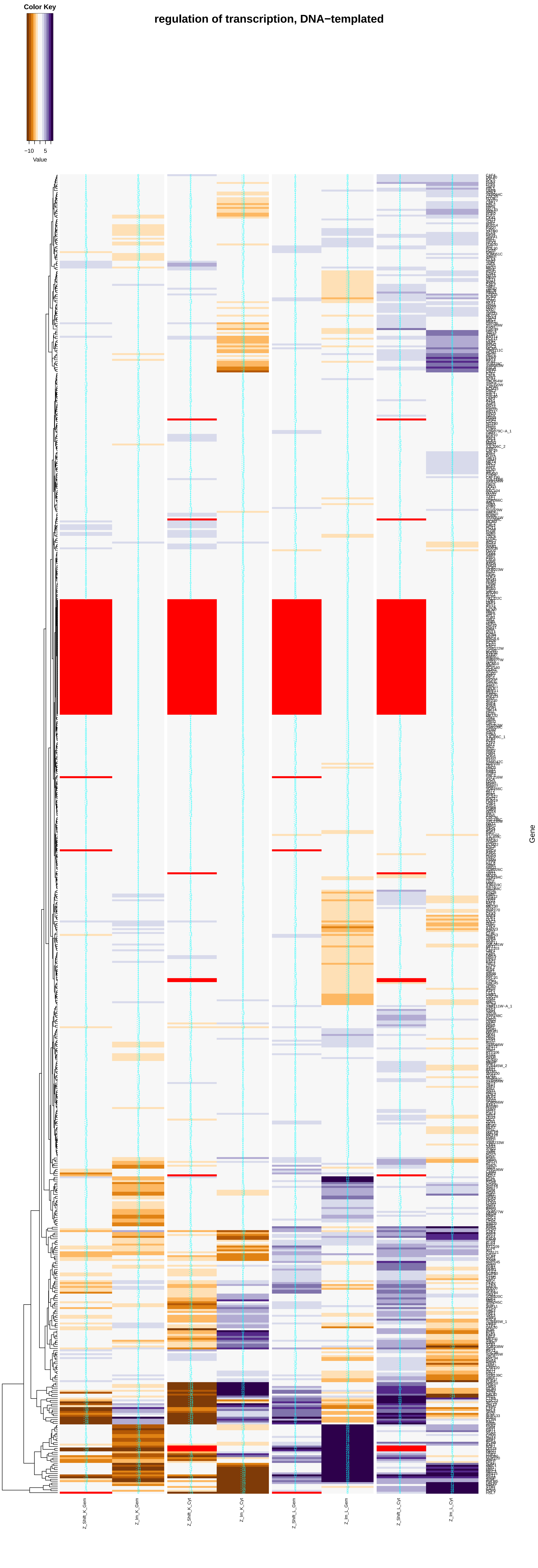

regulation of transcription, DNA−templated
Color Key
−10
5
Value
Gene
Z_Shift_K_Gem
Z_Im_K_Gem
Z_Shift_K_Cyt
Z_Im_K_Cyt
Z_Shift_L_Gem
Z_Im_L_Gem
Z_Shift_L_Cyt
Z_Im_L_Cyt

regulation of transcription involved in G1/S transition of mitotic cell cycle

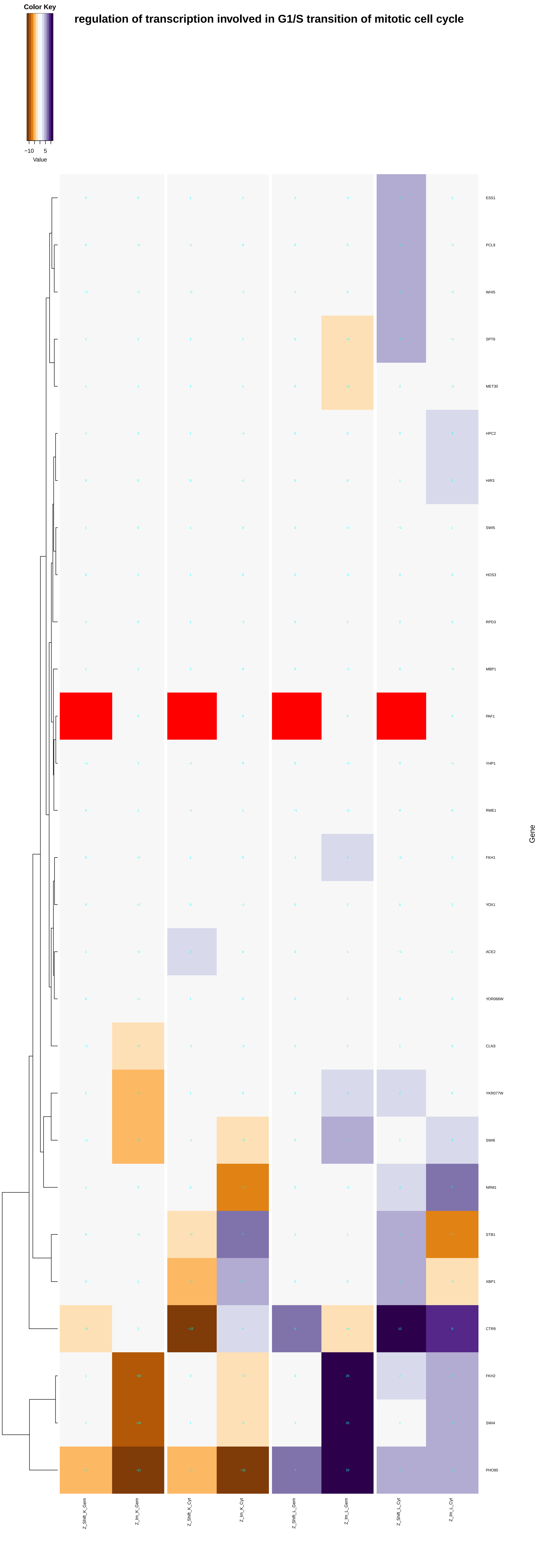

regulation of transcription involved in G2/M transition of mitotic cell cycle

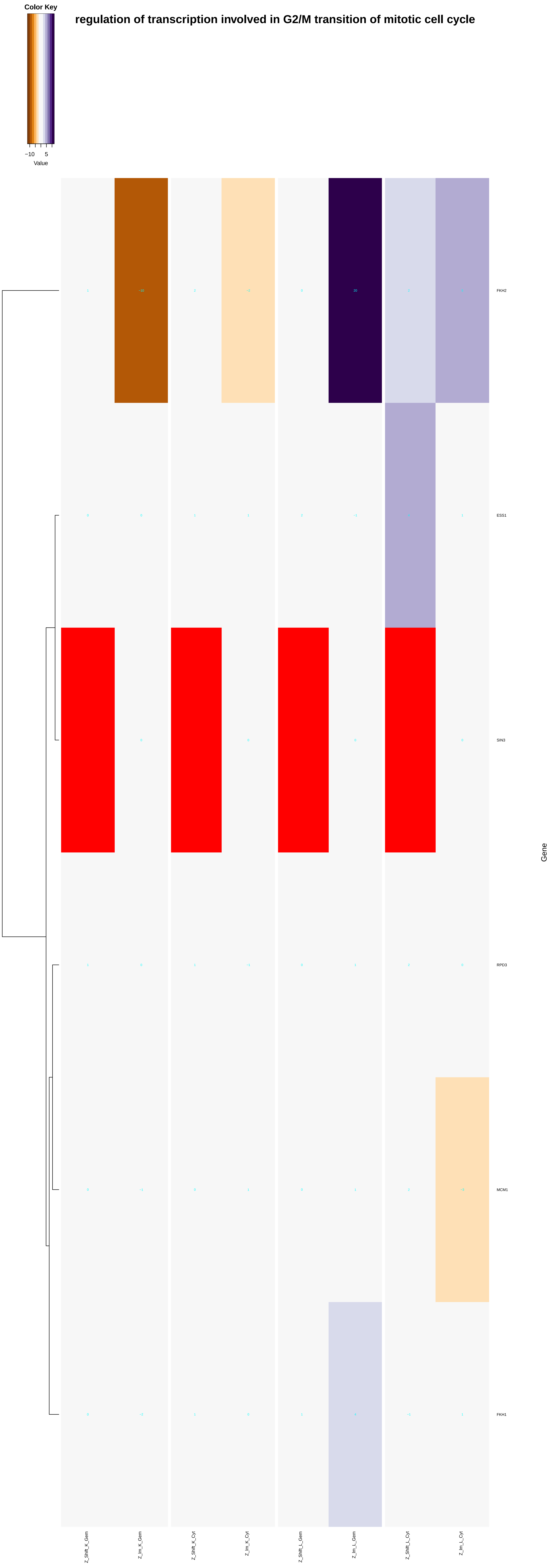

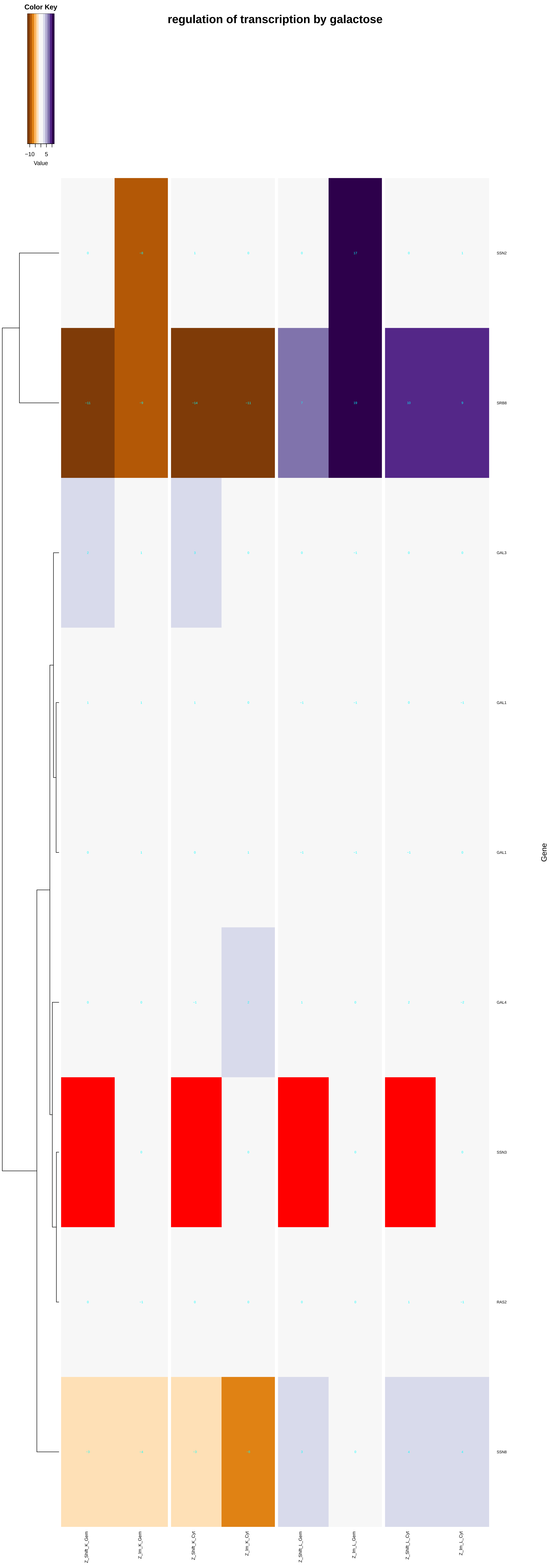
regulation of transcription by galactose
Color Key
−10
5
Value
SSN2
SRB8
GAL3
GAL1
GAL1
GAL4
SSN3
RAS2
SSN8
Gene
Z_Shift_K_Gem
Z_Im_K_Gem
Z_Shift_K_Cyt
Z_Im_K_Cyt
Z_Shift_L_Gem
Z_Im_L_Gem
Z_Shift_L_Cyt
Z_Im_L_Cyt

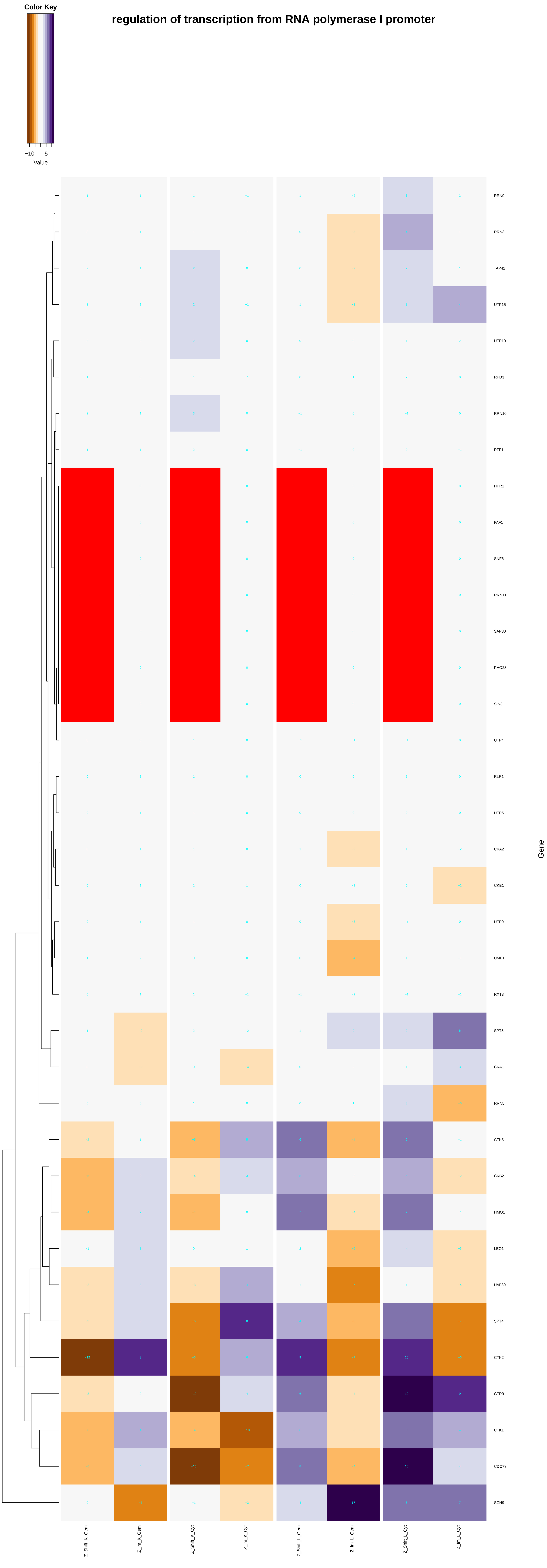

regulation of transcription from RNA polymerase I promoter
Color Key
−10
5
Value
RRN9
RRN3
TAP42
UTP15
UTP10
RPD3
RRN10
RTF1
HPR1
PAF1
SNF6
RRN11
SAP30
PHO23
SIN3
UTP4
RLR1
UTP5
CKA2
CKB1
UTP9
UME1
RXT3
SPT5
CKA1
RRN5
CTK3
CKB2
HMO1
LEO1
UAF30
SPT4
CTK2
CTR9
CTK1
CDC73
SCH9
Gene
Z_Shift_K_Gem
Z_Im_K_Gem
Z_Shift_K_Cyt
Z_Im_K_Cyt
Z_Shift_L_Gem
Z_Im_L_Gem
Z_Shift_L_Cyt
Z_Im_L_Cyt

regulation of transcription from RNA polymerase II promoter

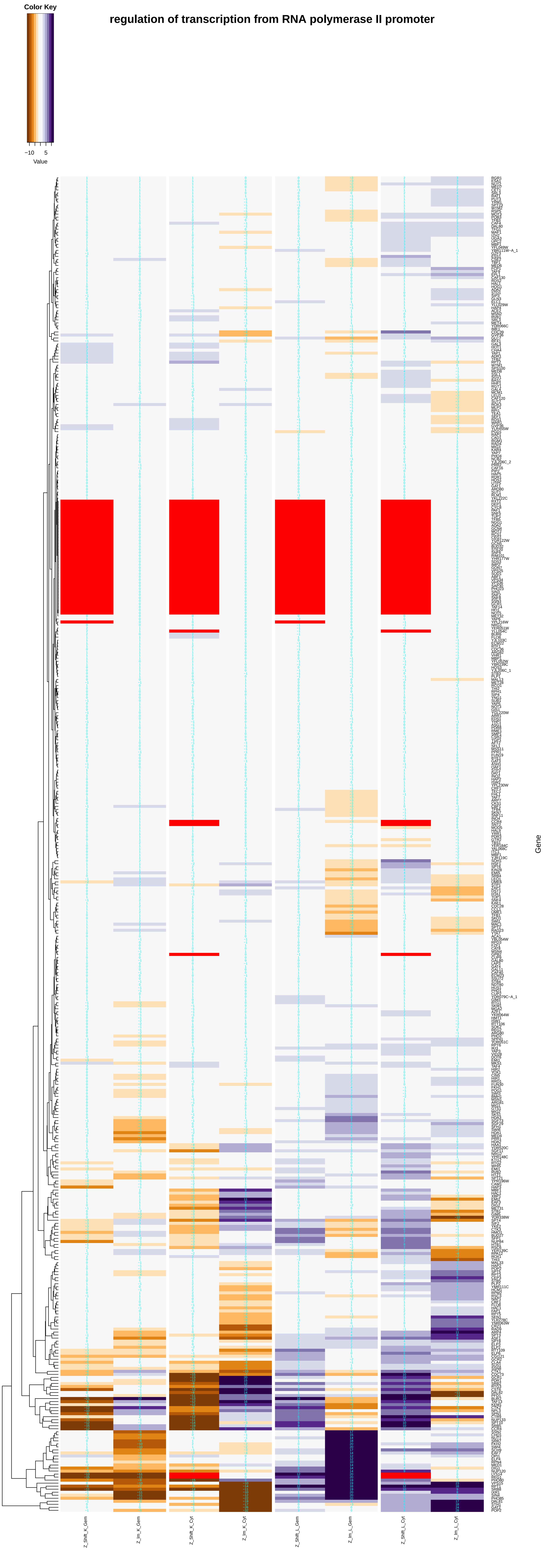

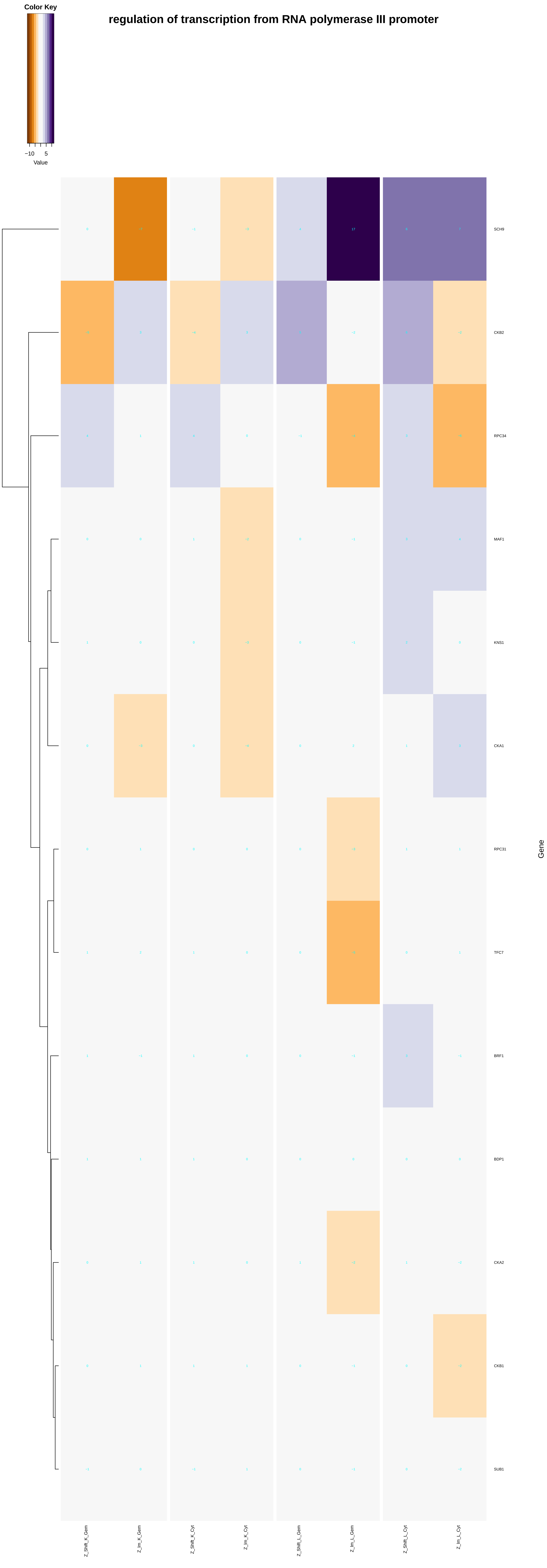
Color Key
Value
−10
5
regulation of transcription from RNA polymerase III promoter
SCH9
CKB2
RPC34
MAF1
KNS1
CKA1
RPC31
TFC7
BRF1
BDP1
CKA2
CKB1
SUB1
Gene
Z_Shift_K_Gem
Z_Im_K_Gem
Z_Shift_K_Cyt
Z_Im_K_Cyt
Z_Shift_L_Gem
Z_Im_L_Gem
Z_Shift_L_Cyt
Z_Im_L_Cyt

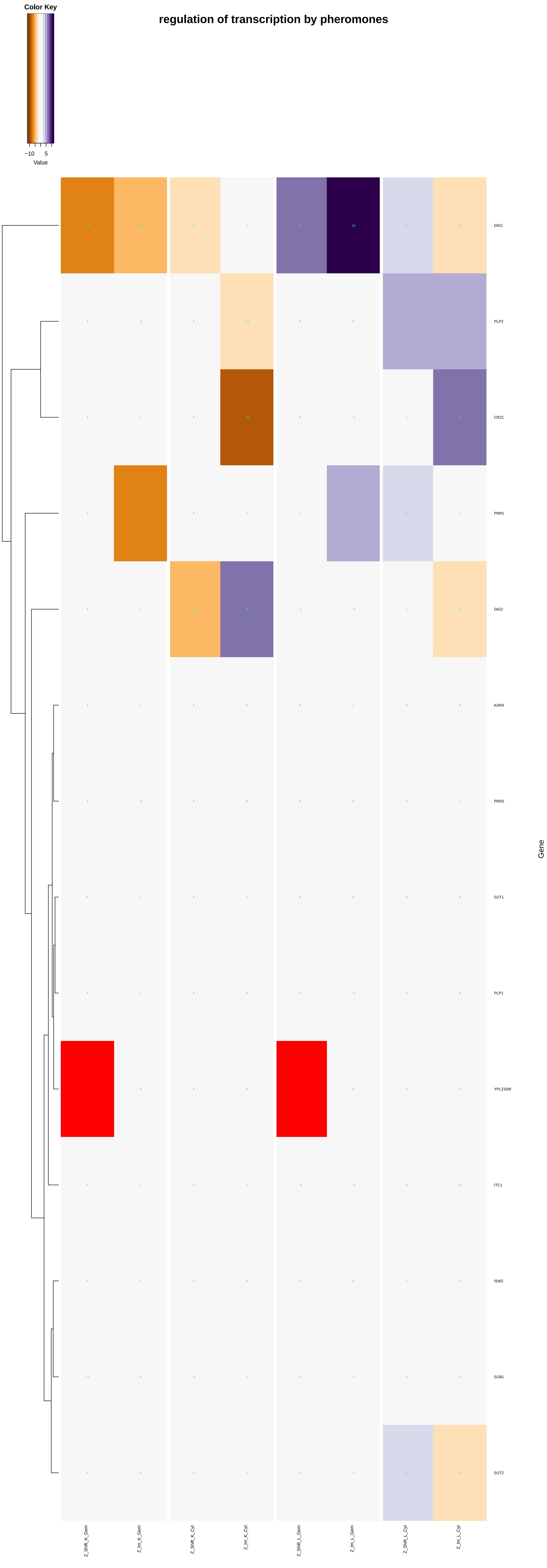

regulation of transcription by pheromones
Color Key
−10 5
Value
DIG1
PLP2
CRZ1
PRR1
DIG2
KAR4
PRR2
SUT1
PLP1
YPL216W
ITC1
ISW2
SUB1
SUT2
Gene
Z_Shift_K_Gem
Z_Im_K_Gem
Z_Shift_K_Cyt
Z_Im_K_Cyt
Z_Shift_L_Gem
Z_Im_L_Gem
Z_Shift_L_Cyt
Z_Im_L_Cyt

regulation of DNA−templated transcription, termination

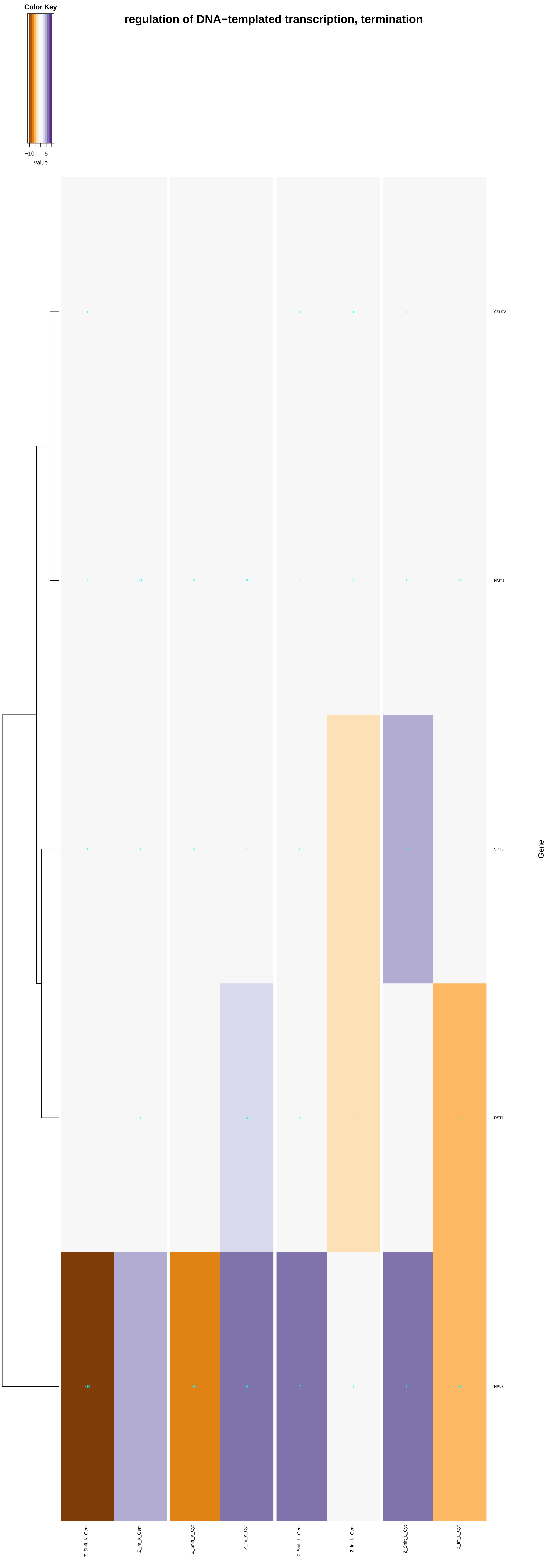

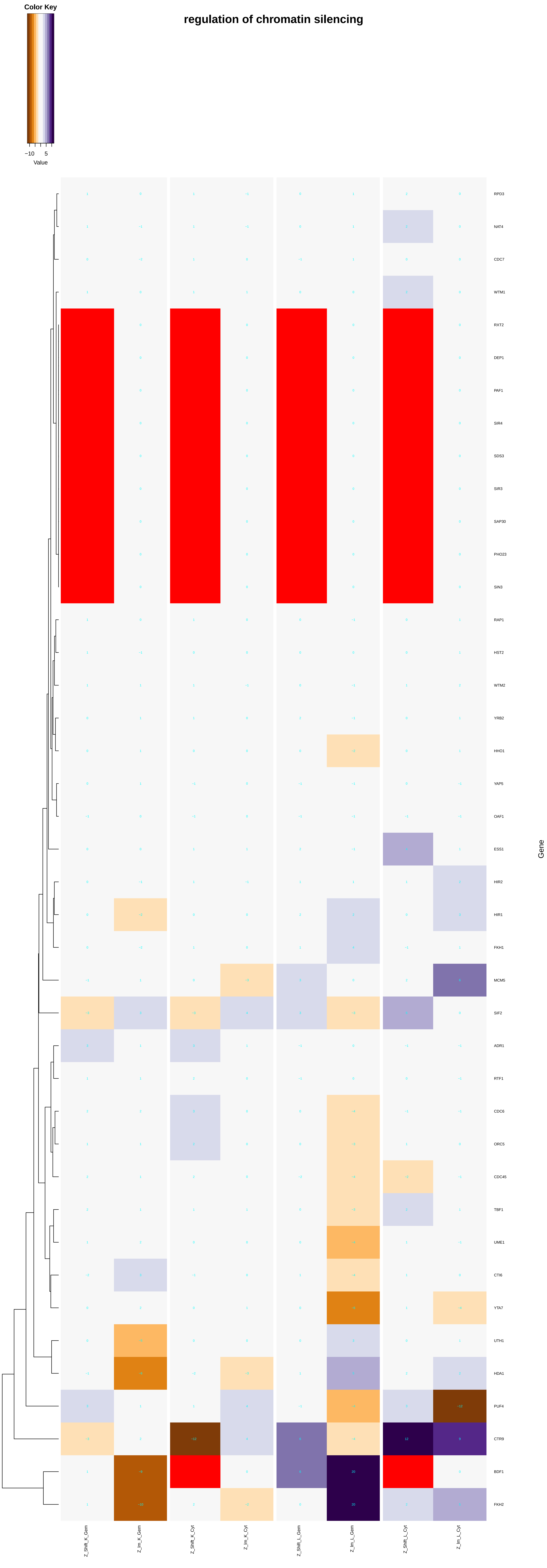
regulation of chromatin silencing
Color Key
Value
Gene

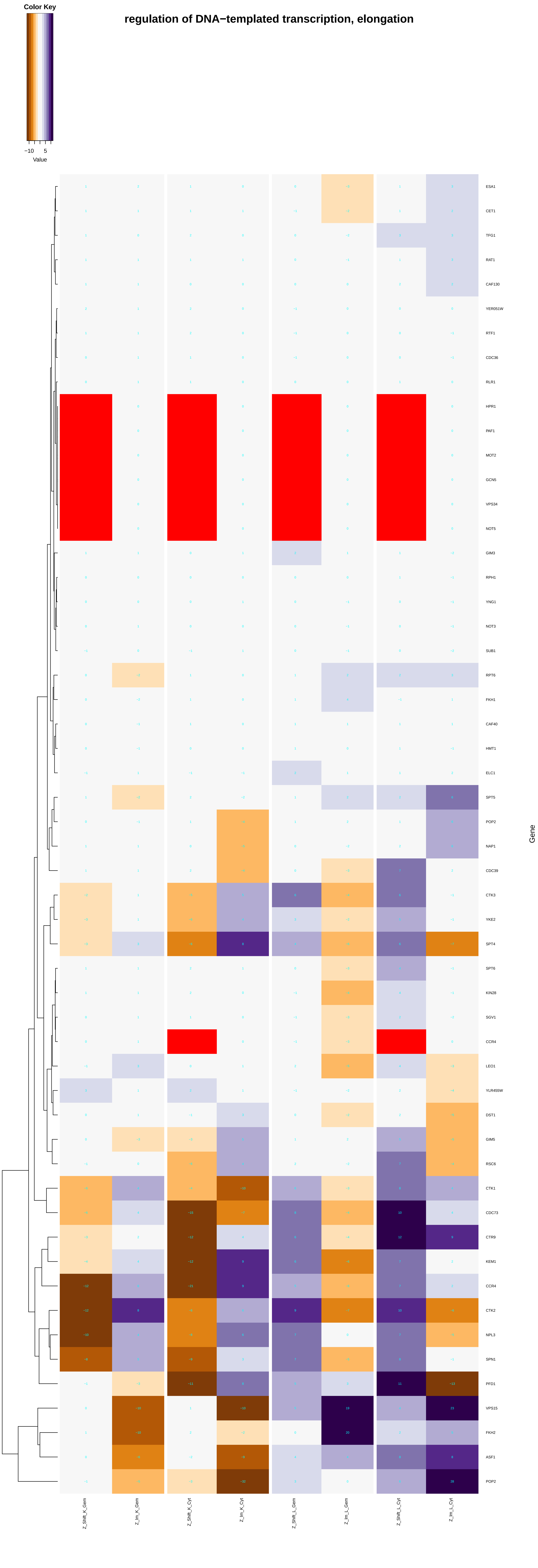

regulation of DNA−templated transcription, elongation
Color Key
−10
5
Value
Gene
ESA1
CET1
TFG1
RAT1
CAF130
YER051W
RTF1
CDC36
RLR1
HPR1
PAF1
MOT2
GCN5
VPS34
NOT5
GIM3
RPH1
YNG1
NOT3
SUB1
RPT6
FKH1
CAF40
HMT1
ELC1
SPT5
POP2
NAP1
CDC39
CTK3
YKE2
SPT4
SPT6
KIN28
SGV1
CCR4
LEO1
YLR455W
DST1
GIM5
RSC6
CTK1
CDC73
CTR9
KEM1
CCR4
CTK2
NPL3
SPN1
PFD1
VPS15
FKH2
ASF1
POP2
Z_Shift_K_Gem
Z_Im_K_Gem
Z_Shift_K_Cyt
Z_Im_K_Cyt
Z_Shift_L_Gem
Z_Im_L_Gem
Z_Shift_L_Cyt
Z_Im_L_Cyt

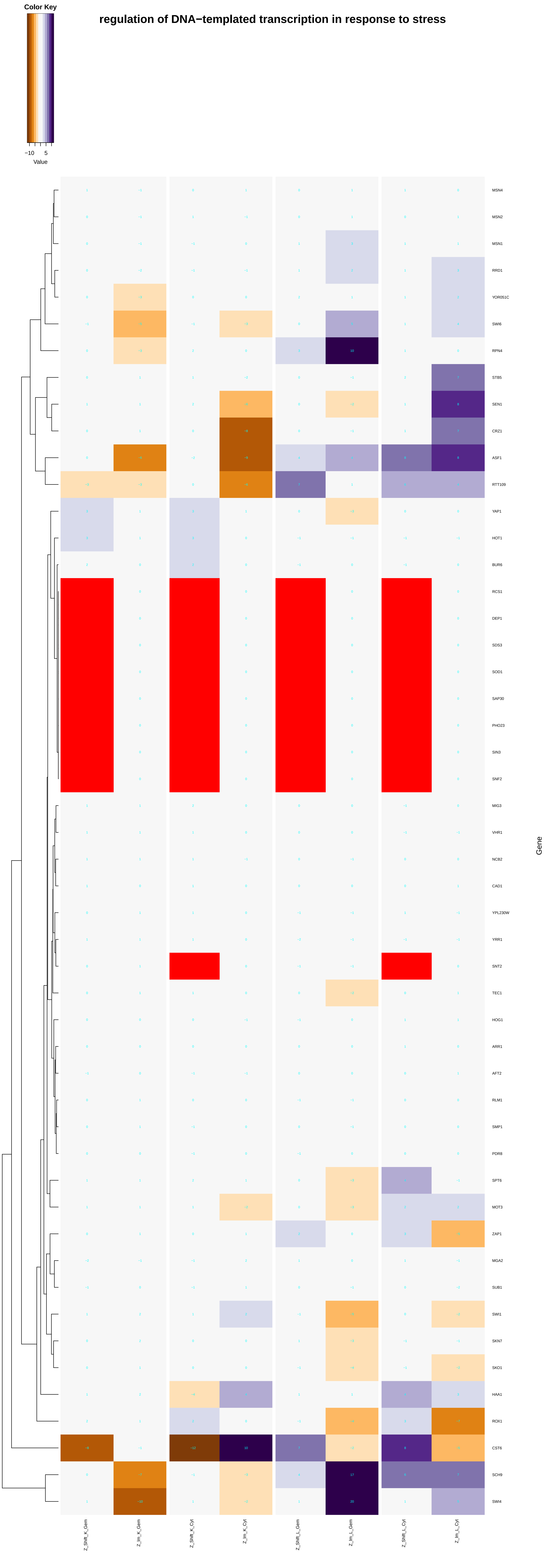
regulation of DNA−templated transcription in response to stress
Color Key
−10
5
Value
MSN4
MSN2
MSN1
RRD1
YOR051C
SWI6
RPN4
STB5
SEN1
CRZ1
ASF1
RTT109
YAP1
HOT1
BUR6
RCS1
DEP1
SDS3
SOD1
SAP30
PHO23
SIN3
SNF2
MIG3
VHR1
NCB2
CAD1
YPL230W
YRR1
SNT2
TEC1
HOG1
ARR1
AFT2
RLM1
SMP1
PDR8
SPT6
MOT3
ZAP1
MGA2
SUB1
SWI1
SKN7
SKO1
HAA1
ROX1
CST6
SCH9
SWI4
Gene
Z_Shift_K_Gem
Z_Im_K_Gem
Z_Shift_K_Cyt
Z_Im_K_Cyt
Z_Shift_L_Gem
Z_Im_L_Gem
Z_Shift_L_Cyt
Z_Im_L_Cyt

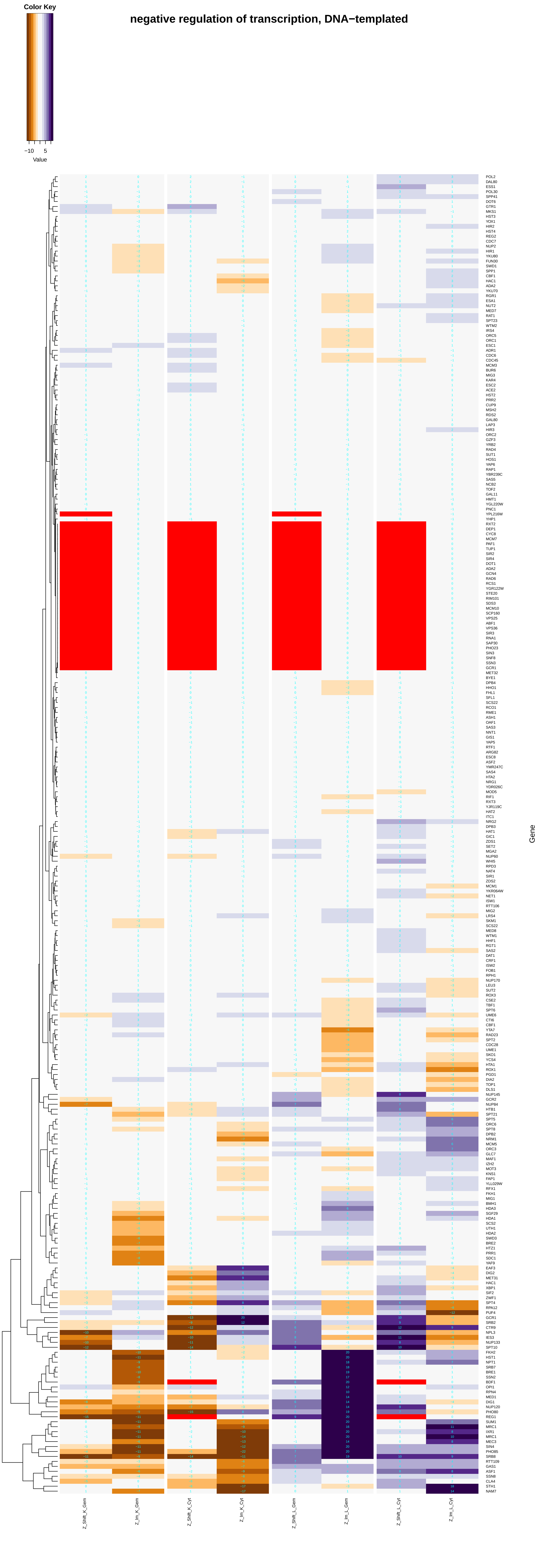
negative regulation of transcription, DNA−templated
Color Key
−10
5
Value
Gene
Z_Shift_K_Gem
Z_Im_K_Gem
Z_Shift_K_Cyt
Z_Im_K_Cyt
Z_Shift_L_Gem
Z_Im_L_Gem
Z_Shift_L_Cyt
Z_Im_L_Cyt
POL2
DAL80
ESS1
POL30
SPP41
DOT6
GTR1
MKS1
HST3
YOX1
HIR2
HST4
REG2
CDC7
NUP2
HIR1
YKU80
FUN30
SWD1
SPP1
CBF1
HAC1
ADA2
YKU70
RGR1
ESA1
NUT2
MED7
RAT1
SPT23
WTM2
IRS4
ORC5
ORC1
ESC1
ADR1
CDC6
CDC45
MCM3
BUR6
MIG3
KAR4
ESC2
ACE2
HST2
PRR2
CUP9
MSH2
RDS2
GAL80
LAP3
HIR3
ORC2
GZF3
YRB2
RAD4
SUT1
HOS1
YAP6
RAP1
YBR239C
SAS5
NCB2
TOF2
GAL11
HMT1
YGL220W
PNC1
YPL216W
YHP1
RXT2
DEP1
CYC8
MCM7
PAF1
TUP1
SIR2
SIR4
DOT1
ADA2
GCN4
RAD6
RCS1
YGR122W
STE20
RIM101
SDS3
MCM10
SCP160
VPS25
ABF1
VPS36
SIR3
RNA1
SAP30
PHO23
SIN3
SNF8
SSN3
GCR1
MET32
BYE1
DPB4
HHO1
FHL1
SFL1
SCS22
RCO1
RME1
ASH1
OAF1
SAS3
NNT1
GIS1
YAP5
RTF1
ARG82
ESC8
ASF2
YMR247C
SAS4
HTA2
NRG1
YDR026C
MOD5
RIF1
RXT3
YJR119C
HAT2
ITC1
NRG2
DPB3
HAT1
GIC1
ZDS1
SET2
MGA2
NUP60
WHI5
RPD3
NAT4
SIR1
ZDS2
MCM1
YKR064W
NET1
ISW1
RTT106
MIG2
LRS4
SKM1
SCS22
MED8
WTM1
HHF1
RGT1
SAS2
DAT1
CRF1
ISW2
FOB1
RPH1
NUP170
LEU3
SUT2
ROX3
CSE2
TBF1
SPT6
UME6
CTI6
CBF1
YTA7
RAD23
SPT2
CDC28
UME1
SKO1
YCS4
HTA1
ROX1
PGD1
DIA2
TOP1
DLS1
NUP145
GCR2
NUP84
HTB1
SPT21
SPT5
ORC6
SPT8
DPB2
NRM1
MCM5
ORC3
GLC7
MAF1
IZH2
MOT3
KNS1
FAP1
YLL029W
RFX1
FKH1
MIG1
BMH1
HDA3
SGF29
HDA1
SCS2
UTH1
HDA2
SWD3
BRE2
HTZ1
PRR1
SDC1
YAF9
EAF3
DIG2
MET31
HAC1
XBP1
SIF2
ZWF1
SPT4
RPA12
PUF4
GCR1
SRB2
CTR9
NPL3
IES3
NUP133
SPT10
FKH2
HST1
NPT1
SRB7
BRE1
SSN2
BDF1
OPI1
RPN4
MED1
DIG1
NUP120
PHO80
REG1
SUM1
MRC1
IXR1
MRC1
MEC3
SIN4
PHO85
SRB8
RTT109
GAS1
ASF1
SSN8
CLA4
STH1
NAM7

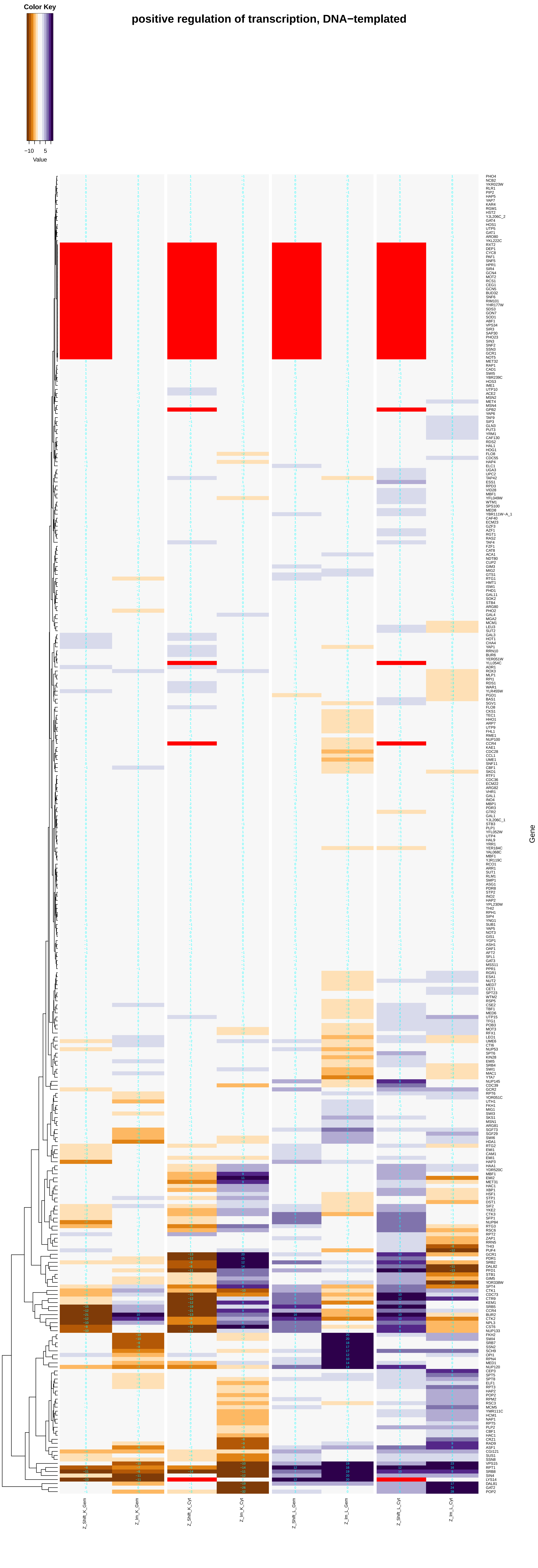

positive regulation of transcription, DNA−templated
Color Key
−10
5
Value
Z_Shift_K_Gem
Z_Im_K_Gem
Z_Shift_K_Cyt
Z_Im_K_Cyt
Z_Shift_L_Gem
Z_Im_L_Gem
Z_Shift_L_Cyt
Z_Im_L_Cyt
Gene

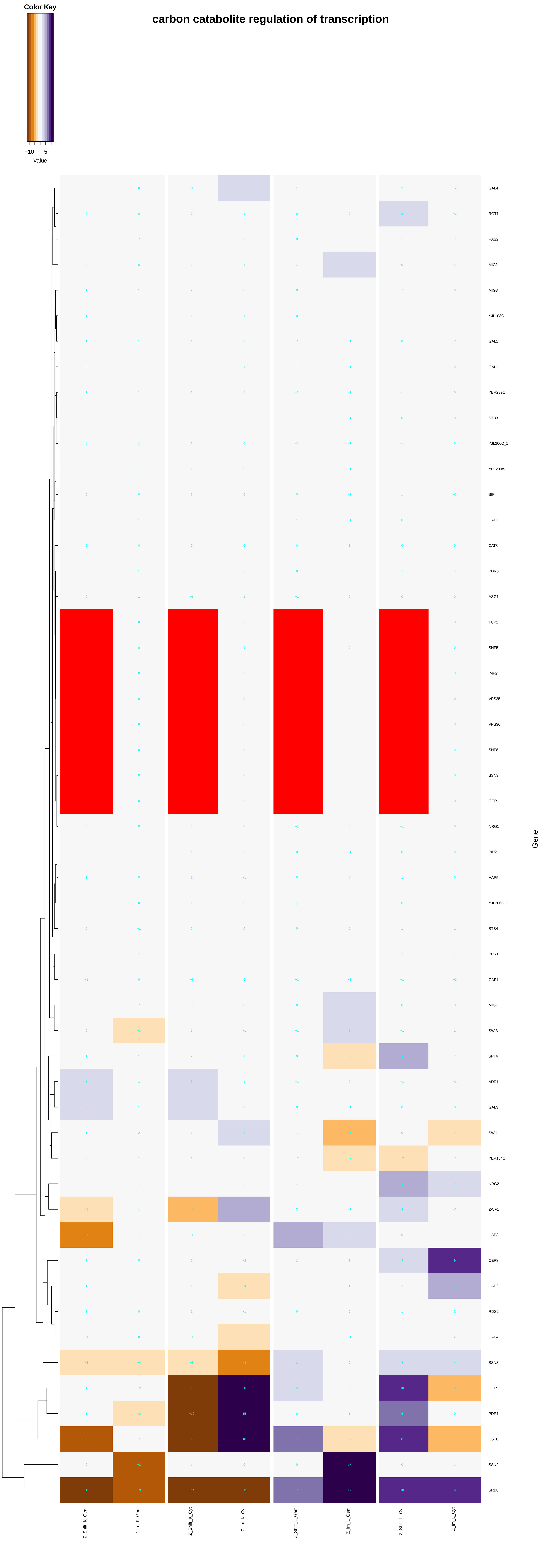

carbon catabolite regulation of transcription
Color Key
−10
5
Value
GAL4
RGT1
RAS2
MIG2
MIG3
YJL103C
GAL1
GAL1
YBR239C
STB3
YJL206C_1
YPL230W
SIP4
HAP2
CAT8
PDR3
ASG1
TUP1
SNF5
IMP2'
VPS25
VPS36
SNF8
SSN3
GCR1
NRG1
PIP2
HAP5
YJL206C_2
STB4
PPR1
OAF1
MIG1
SWI3
SPT6
ADR1
GAL3
SWI1
YER184C
NRG2
ZWF1
HAP3
CEP3
HAP2
RDS2
HAP4
SSN8
GCR1
PDR1
CST6
SSN2
SRB8
Gene
Z_Shift_K_Gem
Z_Im_K_Gem
Z_Shift_K_Cyt
Z_Im_K_Cyt
Z_Shift_L_Gem
Z_Im_L_Gem
Z_Shift_L_Cyt
Z_Im_L_Cyt

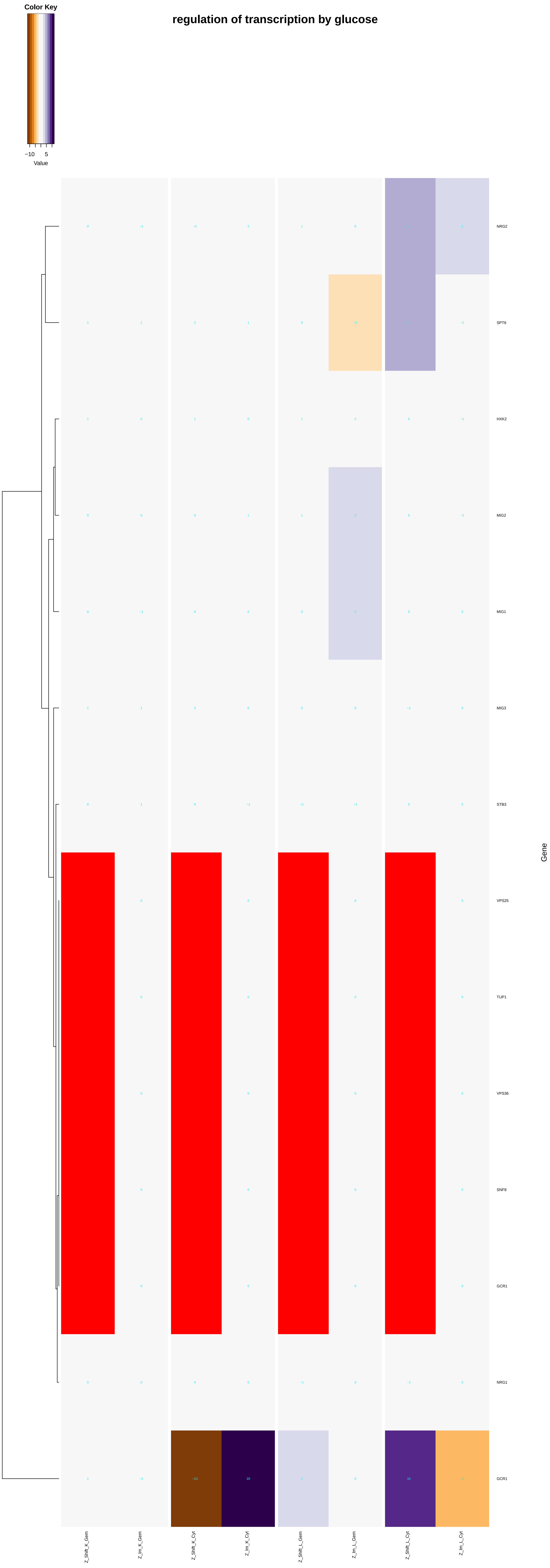

Color Key
−10
5
Value
regulation of transcription by glucose
NRG2
SPT6
HXK2
MIG2
MIG1
MIG3
STB3
VPS25
TUP1
VPS36
SNF8
GCR1
NRG1
GCR1
Gene
Z_Shift_K_Gem
Z_Im_K_Gem
Z_Shift_K_Cyt
Z_Im_K_Cyt
Z_Shift_L_Gem
Z_Im_L_Gem
Z_Shift_L_Cyt
Z_Im_L_Cyt

regulation of transcription involved in meiotic cell cycle

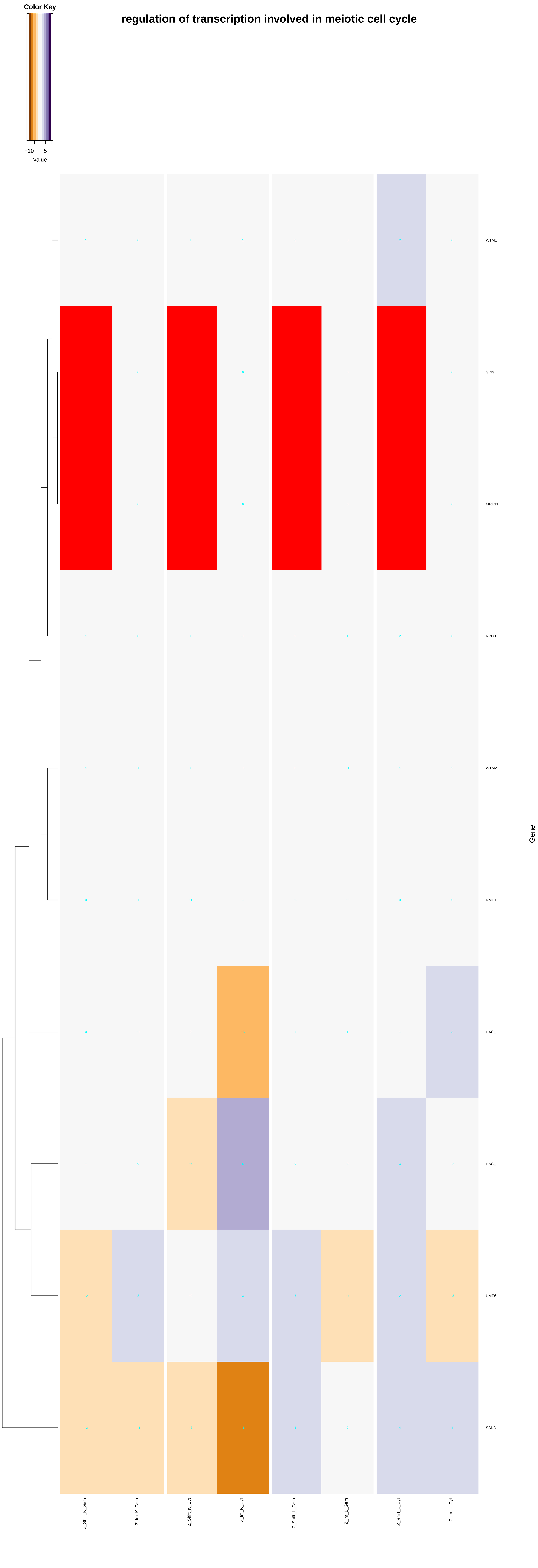

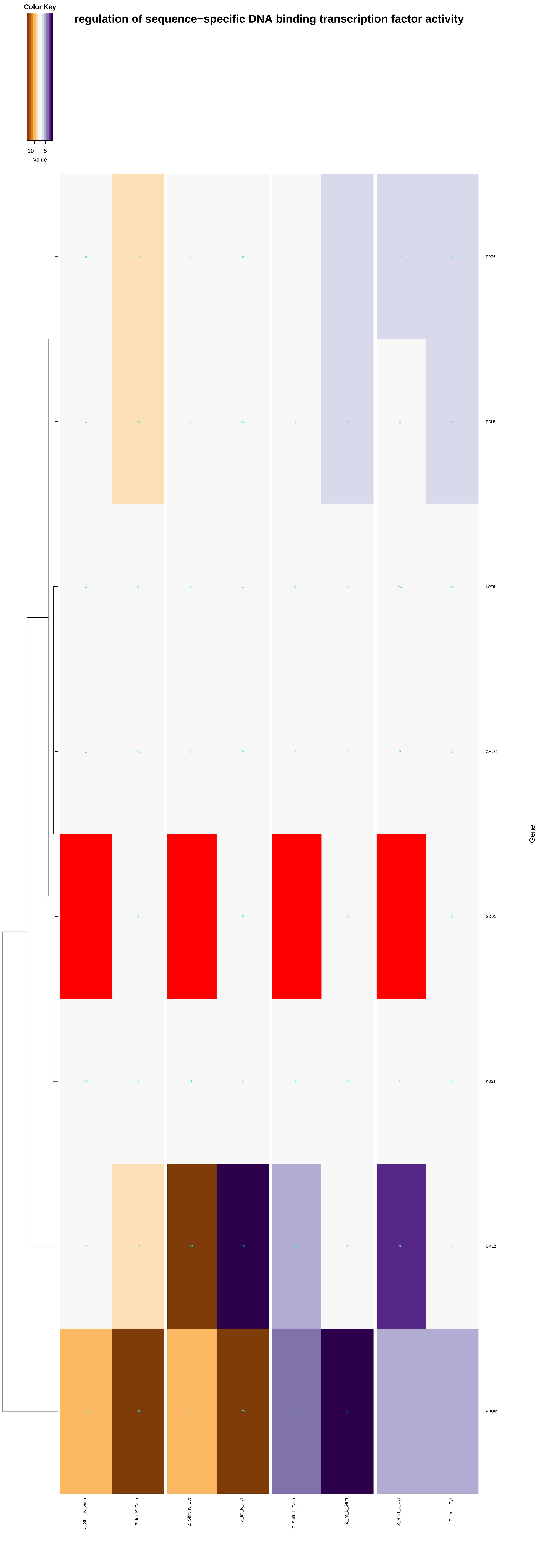

Color Key
−10
5
Value
regulation of sequence−specific DNA binding transcription factor activity
RPT6
PCL5
LOT6
GAL80
SOD1
KSS1
URE2
PHO85
Gene
Z_Shift_K_Gem
Z_Im_K_Gem
Z_Shift_K_Cyt
Z_Im_K_Cyt
Z_Shift_L_Gem
Z_Im_L_Gem
Z_Shift_L_Cyt
Z_Im_L_Cyt

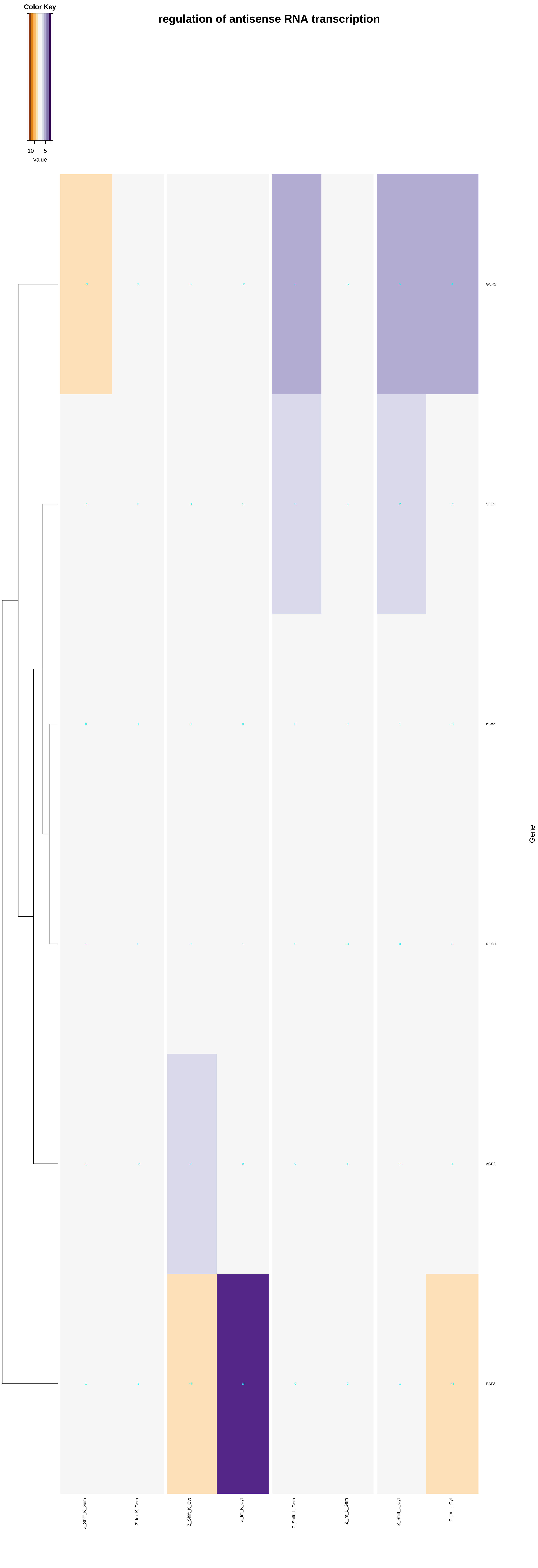
regulation of antisense RNA transcription
Color Key
−10
5
Value
GCR2
SET2
ISW2
RCO1
ACE2
EAF3
Gene
Z_Shift_K_Gem
Z_Im_K_Gem
Z_Shift_K_Cyt
Z_Im_K_Cyt
Z_Shift_L_Gem
Z_Im_L_Gem
Z_Shift_L_Cyt
Z_Im_L_Cyt

nitrogen catabolite regulation of transcription

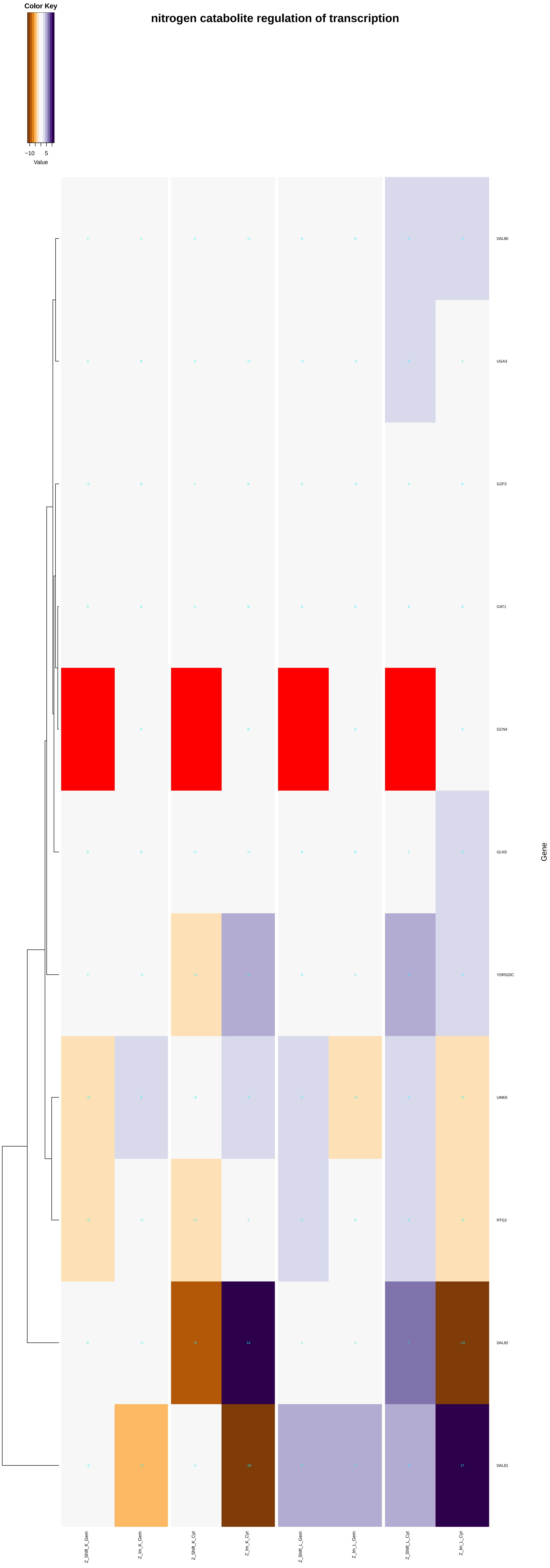

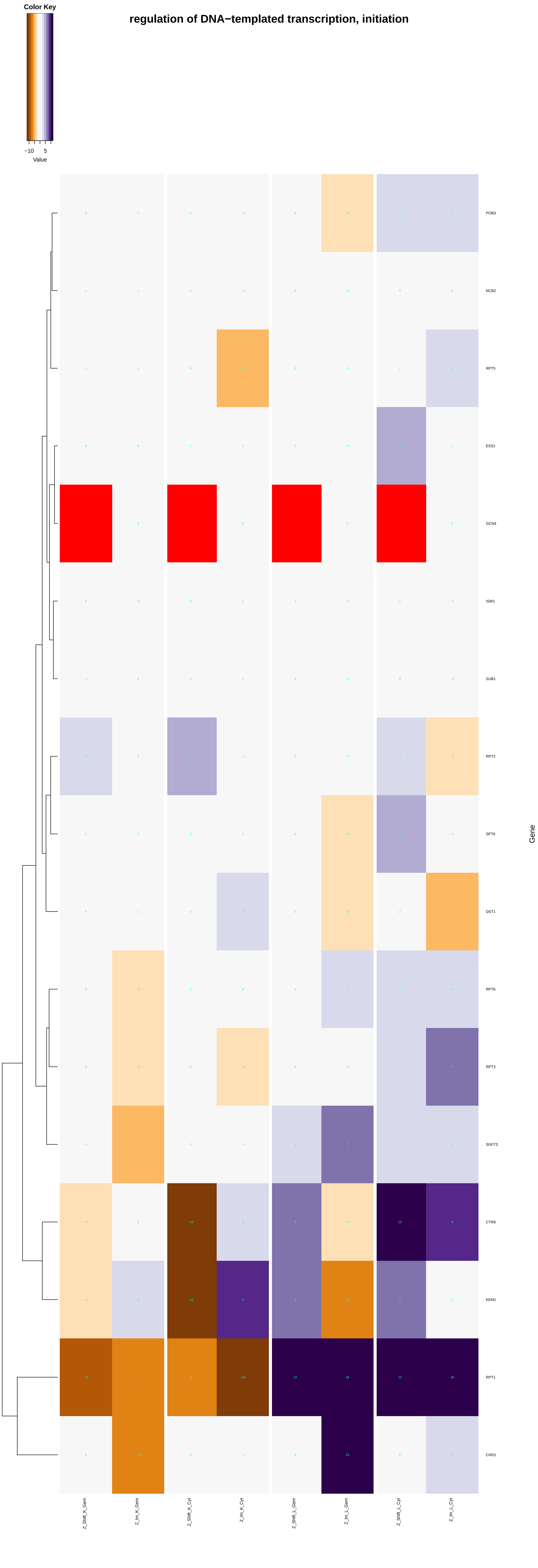

Color Key
−10
5
Value
regulation of DNA−templated transcription, initiation
POB3
NCB2
RPT5
ESS1
GCN4
ISW1
SUB1
RPT2
SPT6
DST1
RPT6
RPT3
SGF73
CTR9
KEM1
RPT1
CHD1
Gene
Z_Shift_K_Gem
Z_Im_K_Gem
Z_Shift_K_Cyt
Z_Im_K_Cyt
Z_Shift_L_Gem
Z_Im_L_Gem
Z_Shift_L_Cyt
Z_Im_L_Cyt

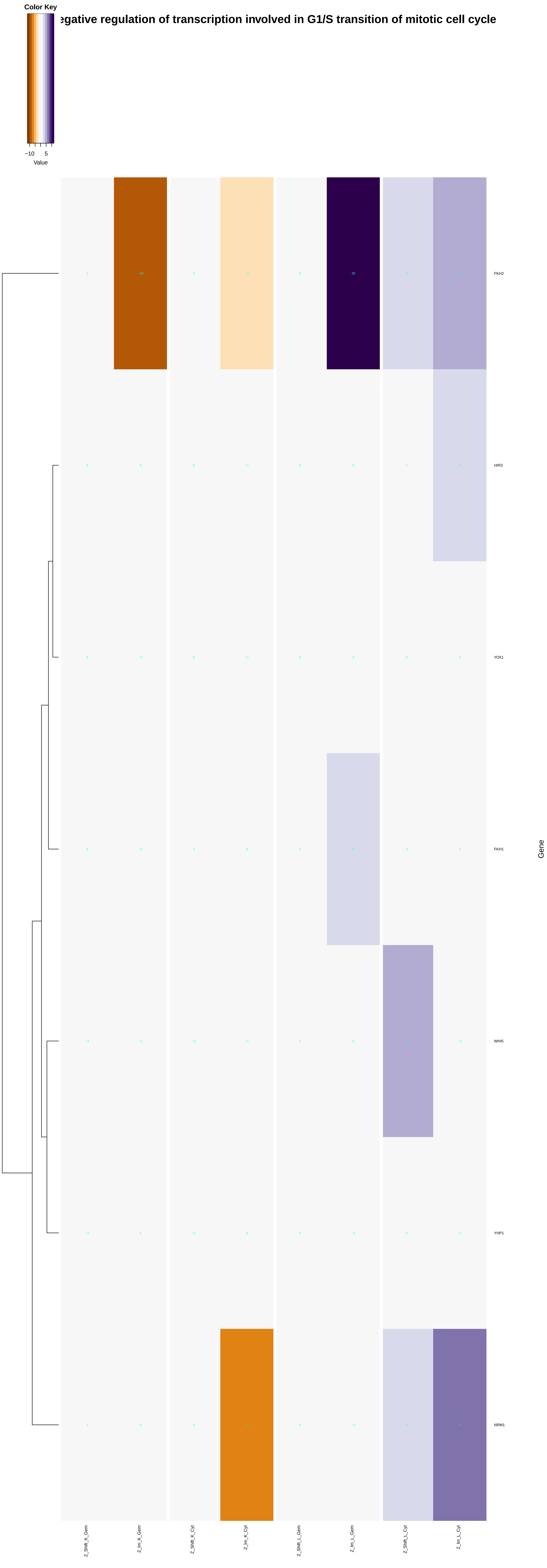

egative regulation of transcription involved in G1/S transition of mitotic cell cycle
Color Key
−10
5
Value
FKH2
HIR3
YOX1
FKH1
WHI5
YHP1
NRM1
Gene
Z_Shift_K_Gem
Z_Im_K_Gem
Z_Shift_K_Cyt
Z_Im_K_Cyt
Z_Shift_L_Gem
Z_Im_L_Gem
Z_Shift_L_Cyt
Z_Im_L_Cyt

ositive regulation of transcription involved in G1/S transition of mitotic cell cycle

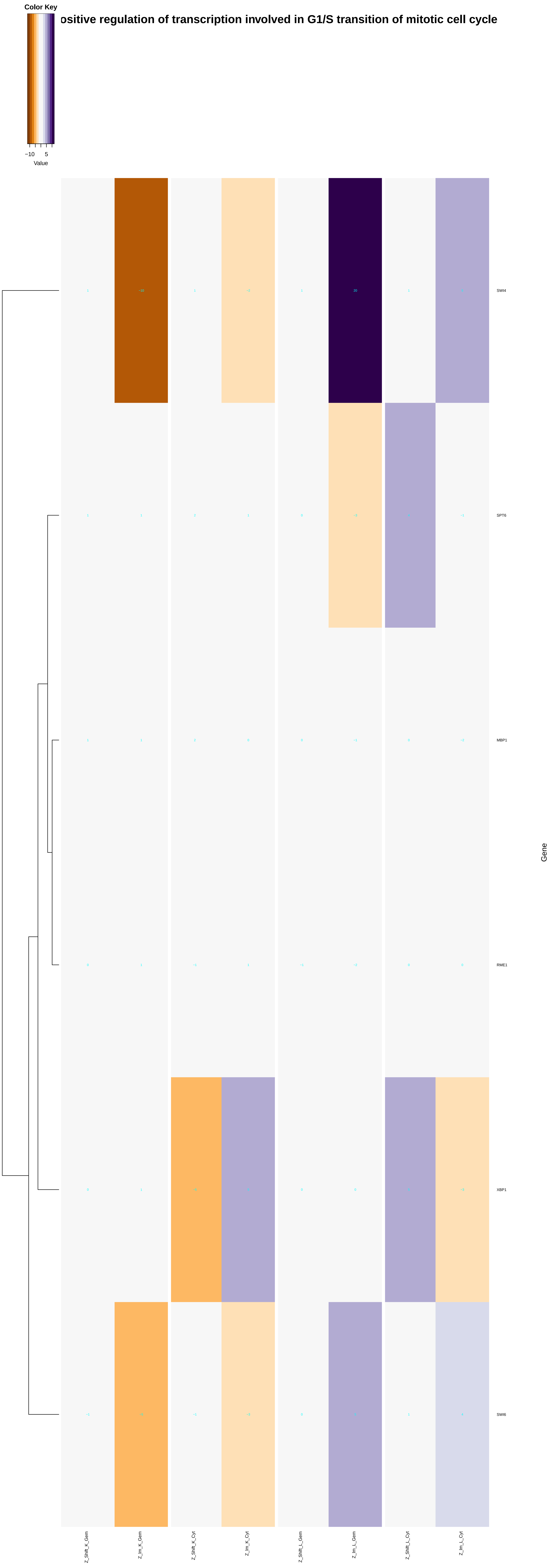

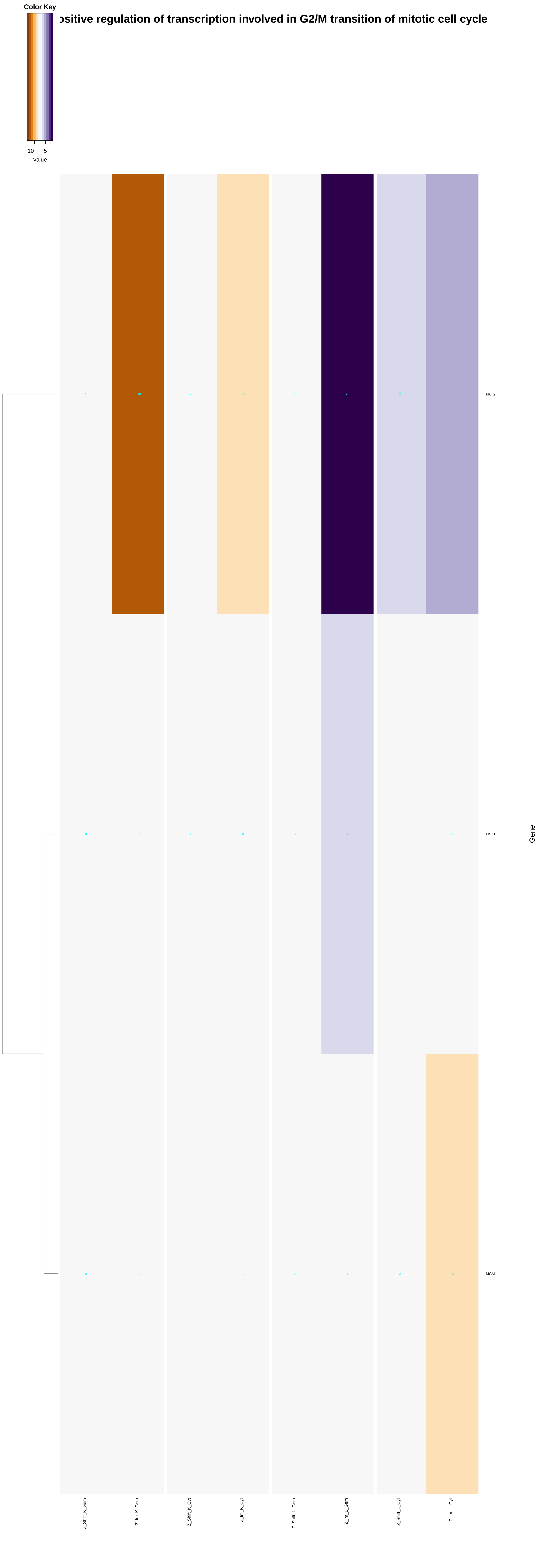
ositive regulation of transcription involved in G2/M transition of mitotic cell cycle
Color Key
−10
5
Value
FKH2
FKH1
MCM1
Gene
Z_Shift_K_Gem
Z_Im_K_Gem
Z_Shift_K_Cyt
Z_Im_K_Cyt
Z_Shift_L_Gem
Z_Im_L_Gem
Z_Shift_L_Cyt
Z_Im_L_Cyt

positive regulation of transcription by galactose

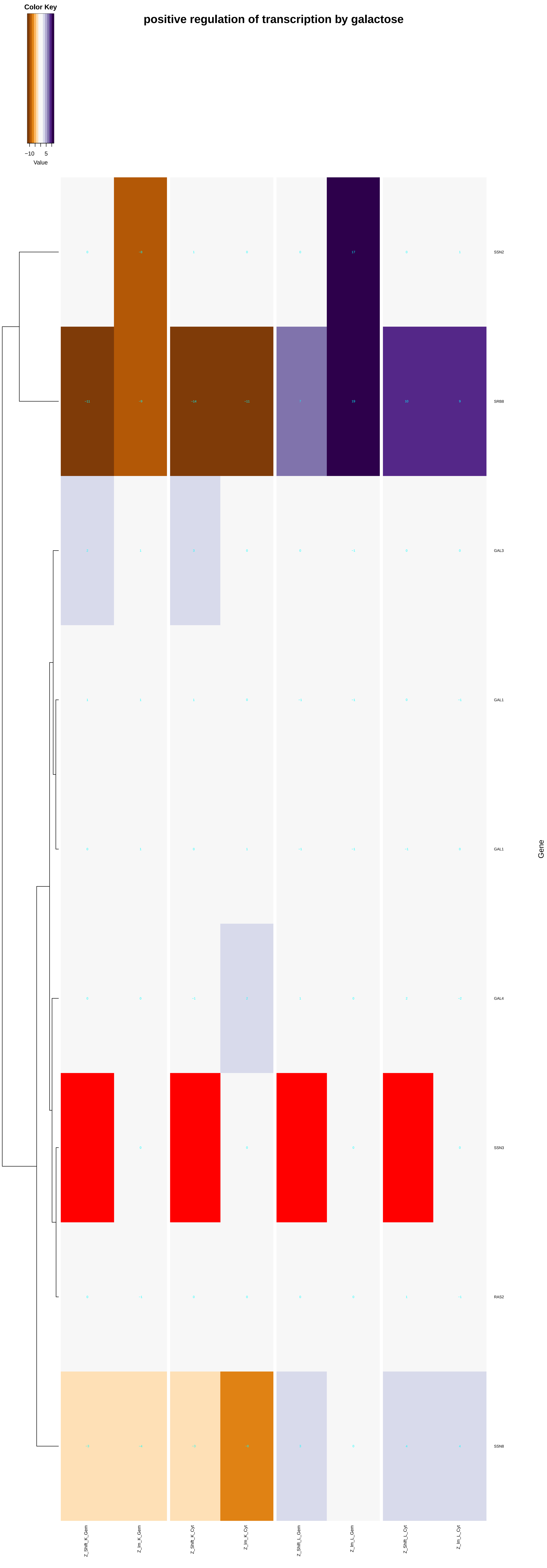

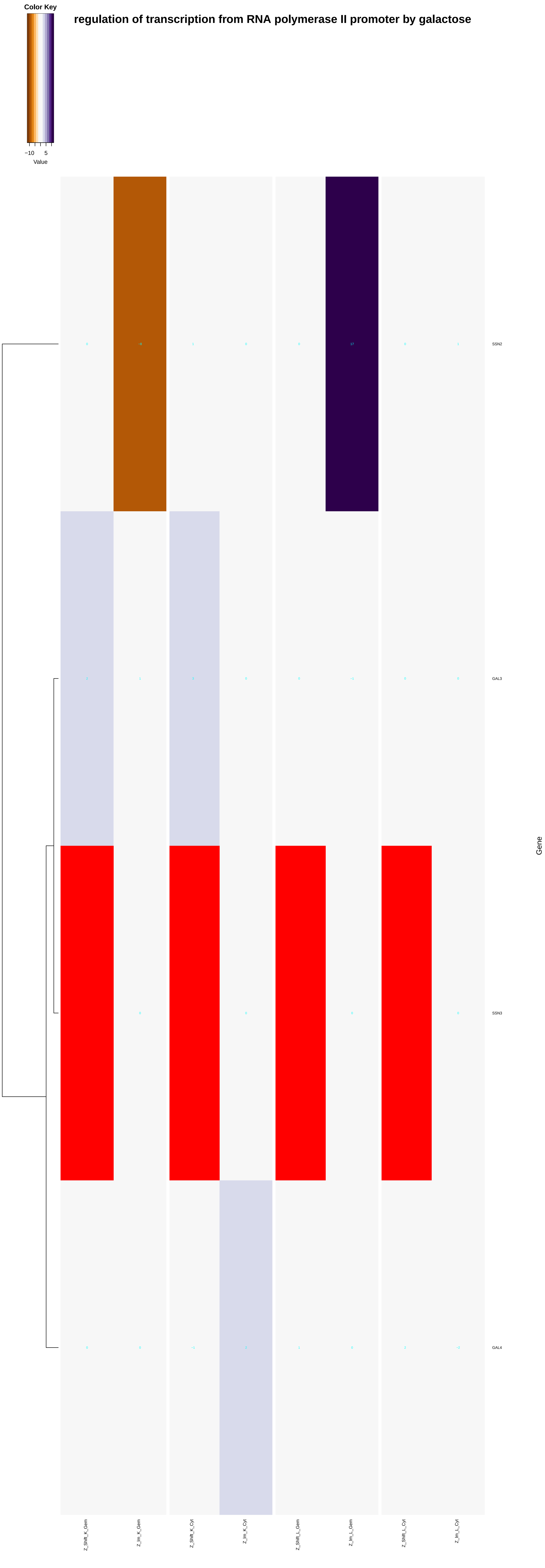
regulation of transcription from RNA polymerase II promoter by galactose
Color Key
−10
5
Value
SSN2
GAL3
SSN3
GAL4
Gene
Z_Shift_K_Gem
Z_lm_K_Gem
Z_Shift_K_Cyt
Z_lm_K_Cyt
Z_Shift_L_Gem
Z_lm_L_Gem
Z_Shift_L_Cyt
Z_lm_L_Cyt

negative regulation of transcription from RNA polymerase I promoter

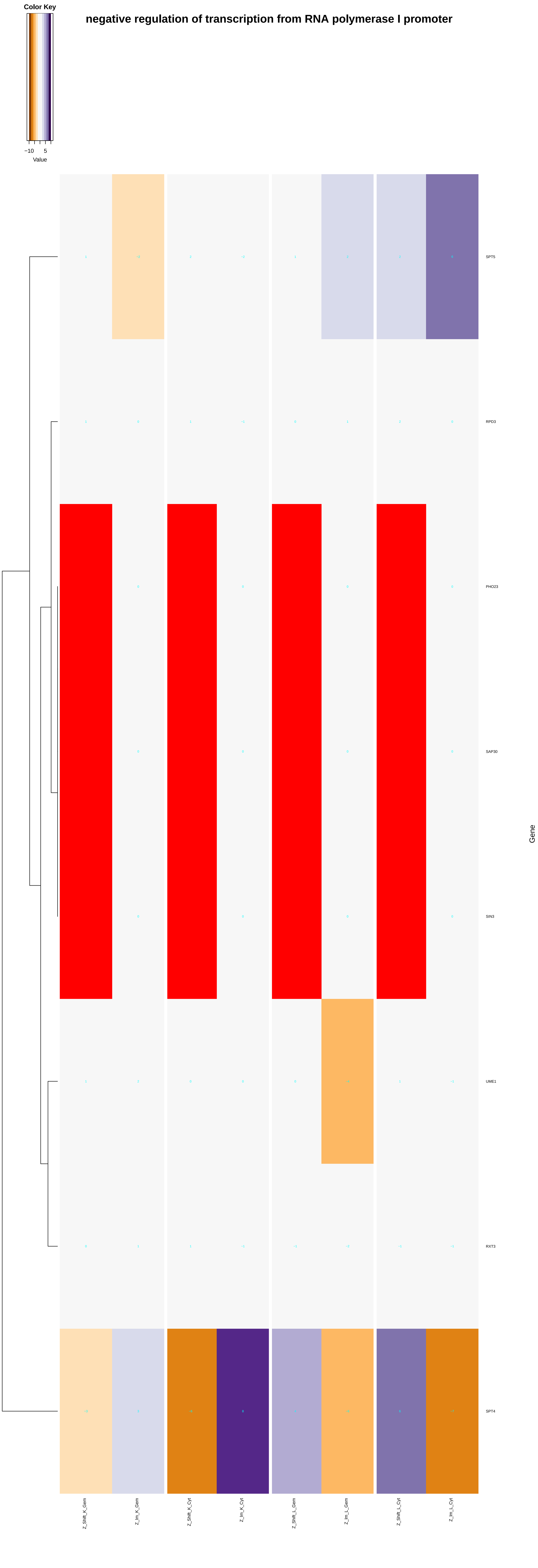

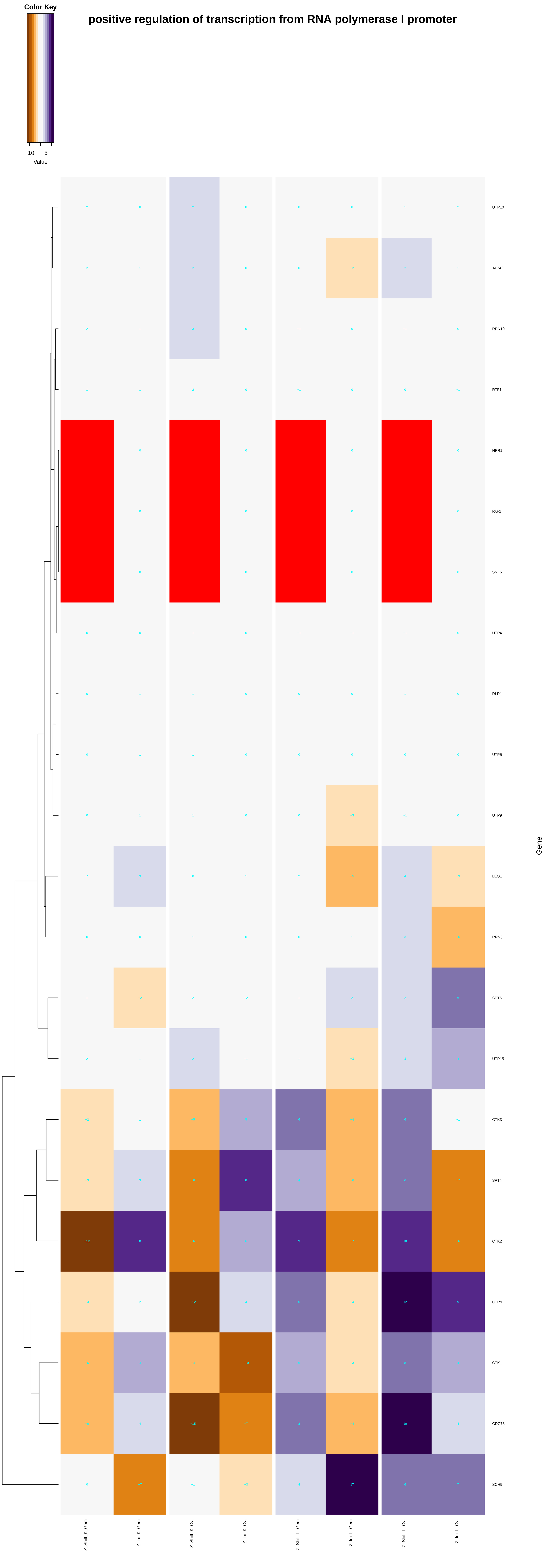
positive regulation of transcription from RNA polymerase I promoter
Color Key
−10
5
Value
Gene
UTP10
TAP42
RRN10
RTF1
HPR1
PAF1
SNF6
UTP4
RLR1
UTP5
UTP9
LEO1
RRN5
SPT5
UTP15
CTK3
SPT4
CTK2
CTR9
CTK1
CDC73
SCH9
Z_Shift_K_Gem
Z_lm_K_Gem
Z_Shift_K_Cyt
Z_lm_K_Cyt
Z_Shift_L_Gem
Z_lm_L_Gem
Z_Shift_L_Cyt
Z_lm_L_Cyt

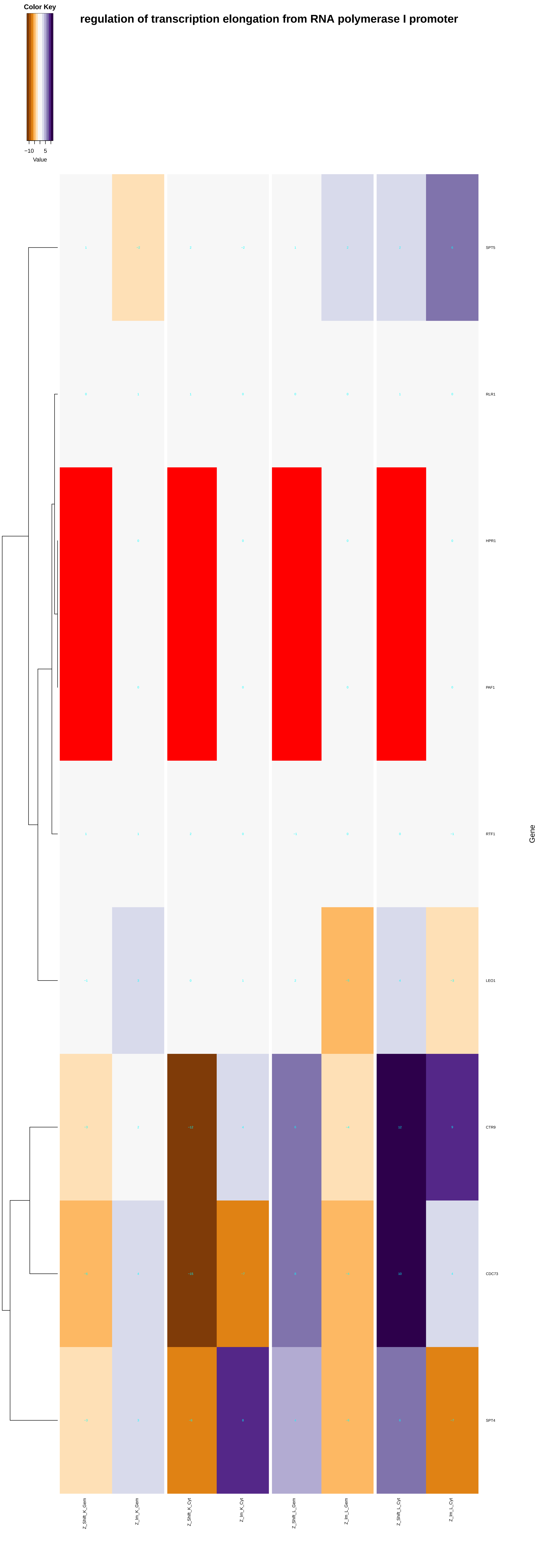

Color Key
−10
5
Value
regulation of transcription elongation from RNA polymerase I promoter
SPT5
RLR1
HPR1
PAF1
RTF1
LEO1
CTR9
CDC73
SPT4
Gene
Z_Shift_K_Gem
Z_Im_K_Gem
Z_Shift_K_Cyt
Z_Im_K_Cyt
Z_Shift_L_Gem
Z_Im_L_Gem
Z_Shift_L_Cyt
Z_Im_L_Cyt

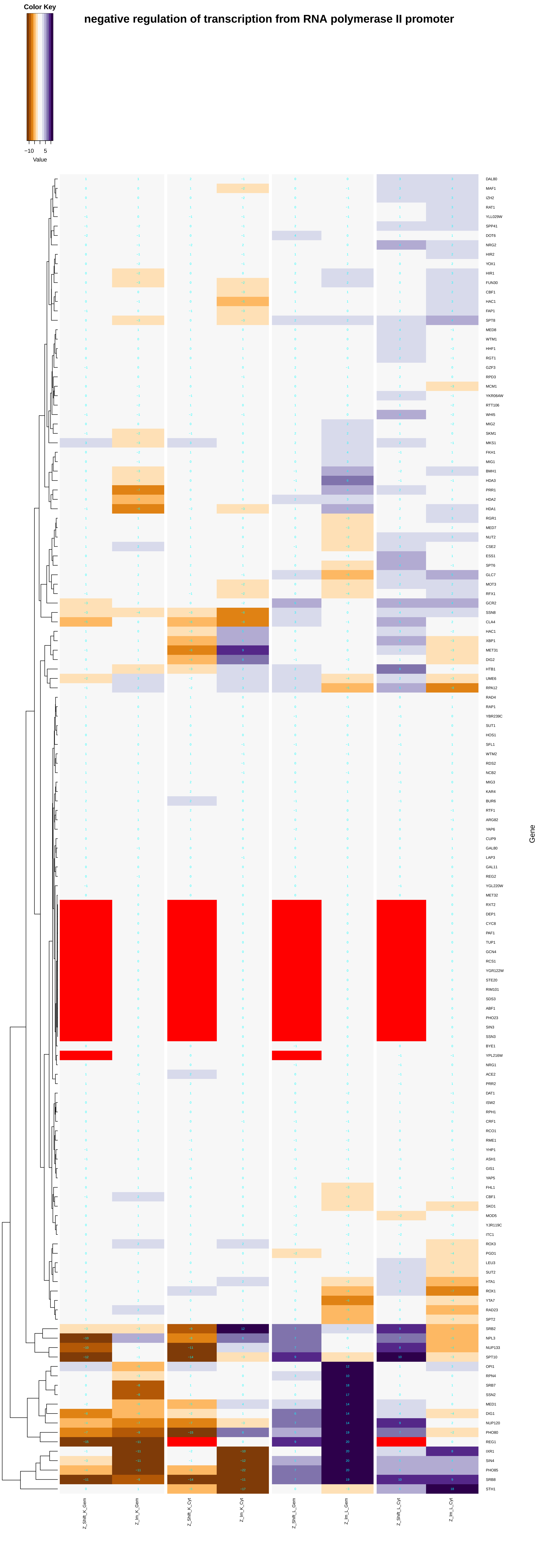
negative regulation of transcription from RNA polymerase II promoter
Color Key
−10 5
Value
Gene
Z_Shift_K_Gem
Z_Im_K_Gem
Z_Shift_K_Cyt
Z_Im_K_Cyt
Z_Shift_L_Gem
Z_Im_L_Gem
Z_Shift_L_Cyt
Z_Im_L_Cyt

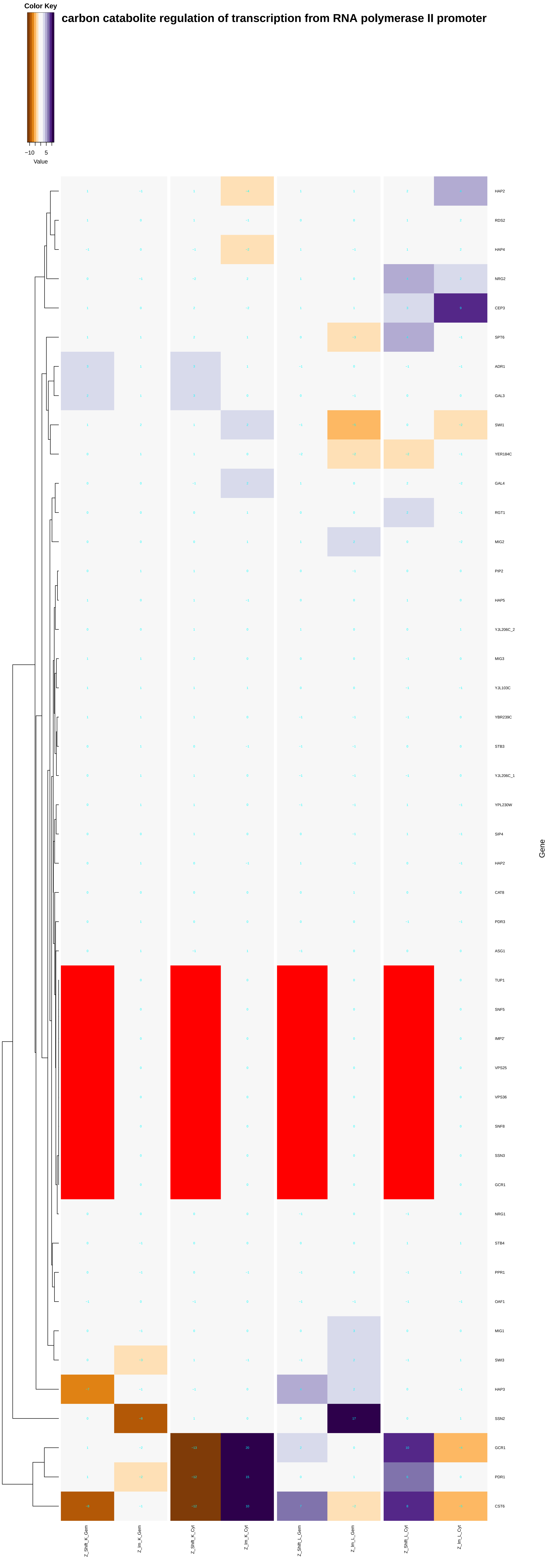
carbon catabolite regulation of transcription from RNA polymerase II promoter
Color Key
−10
5
Value
HAP2
RDS2
HAP4
NRG2
CEP3
SPT6
ADR1
GAL3
SWI1
YER184C
GAL4
RGT1
MIG2
PIP2
HAP5
YJL206C_2
MIG3
YJL103C
YBR239C
STB3
YJL206C_1
YPL230W
SIP4
HAP2
CAT8
PDR3
ASG1
TUP1
SNF5
IMP2'
VPS25
VPS36
SNF8
SSN3
GCR1
NRG1
STB4
PPR1
OAF1
MIG1
SWI3
HAP3
SSN2
GCR1
PDR1
CST6
Gene
Z_Shift_K_Gem
Z_Im_K_Gem
Z_Shift_K_Cyt
Z_Im_K_Cyt
Z_Shift_L_Gem
Z_Im_L_Gem
Z_Shift_L_Cyt
Z_Im_L_Cyt

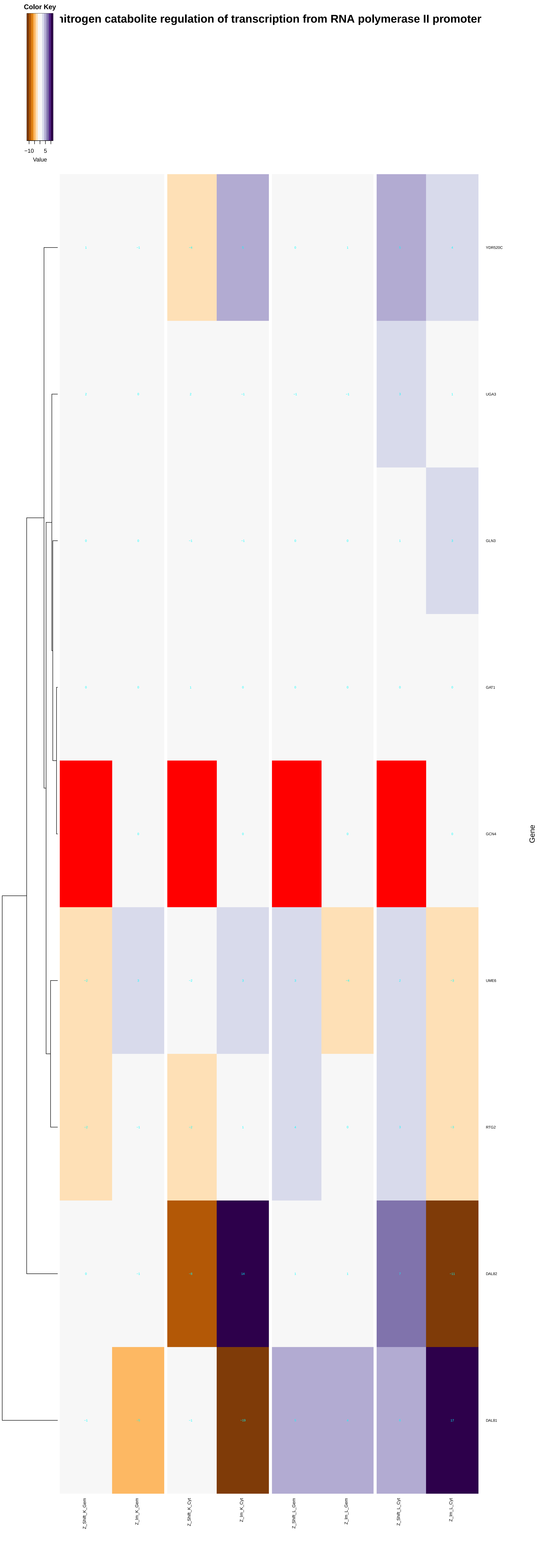
Color Key
−10 5
Value
itrogen catabolite regulation of transcription from RNA polymerase II promoter
YDR520C
UGA3
GLN3
GAT1
GCN4
UME6
RTG2
DAL82
DAL81
Gene
Z_Shift_K_Gem
Z_Im_K_Gem
Z_Shift_K_Cyt
Z_Im_K_Cyt
Z_Shift_L_Gem
Z_Im_L_Gem
Z_Shift_L_Cyt
Z_Im_L_Cyt

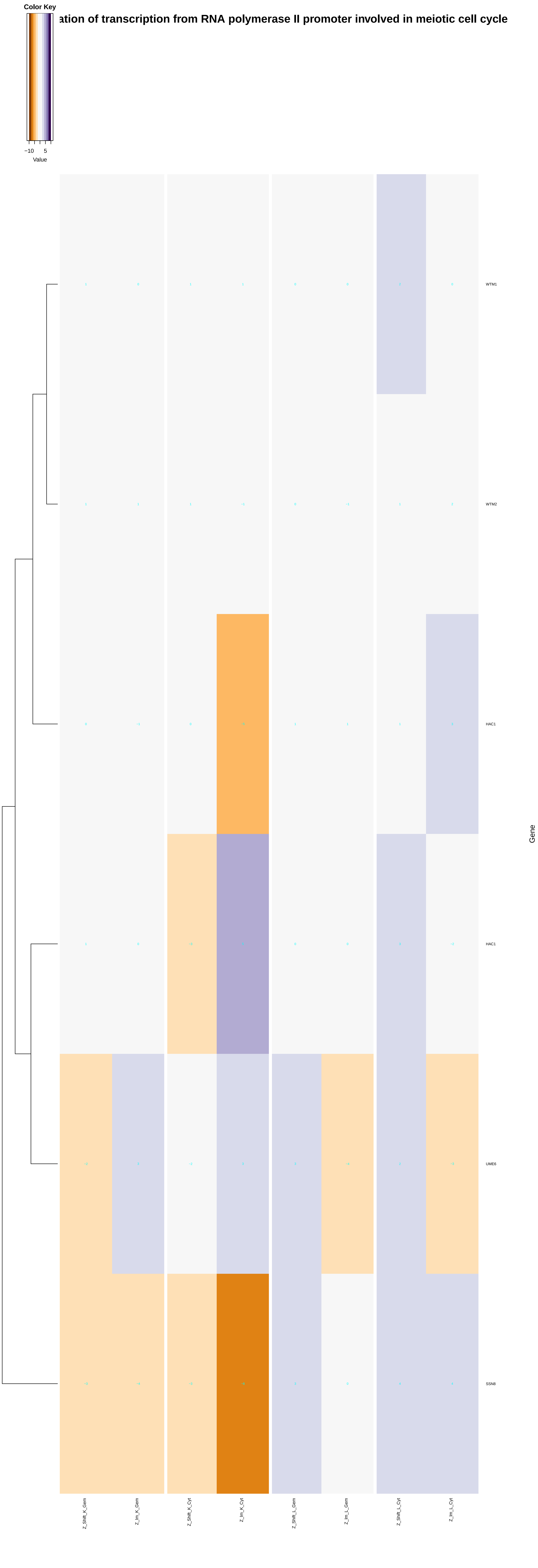
ation of transcription from RNA polymerase II promoter involved in meiotic cell cycle
Color Key
−10
5
Value
WTM1
WTM2
HAC1
HAC1
UME6
SSN8
Gene
Z_Shift_K_Gem
Z_Im_K_Gem
Z_Shift_K_Cyt
Z_Im_K_Cyt
Z_Shift_L_Gem
Z_Im_L_Gem
Z_Shift_L_Cyt
Z_Im_L_Cyt

regulation of transcription elongation from RNA polymerase II promoter

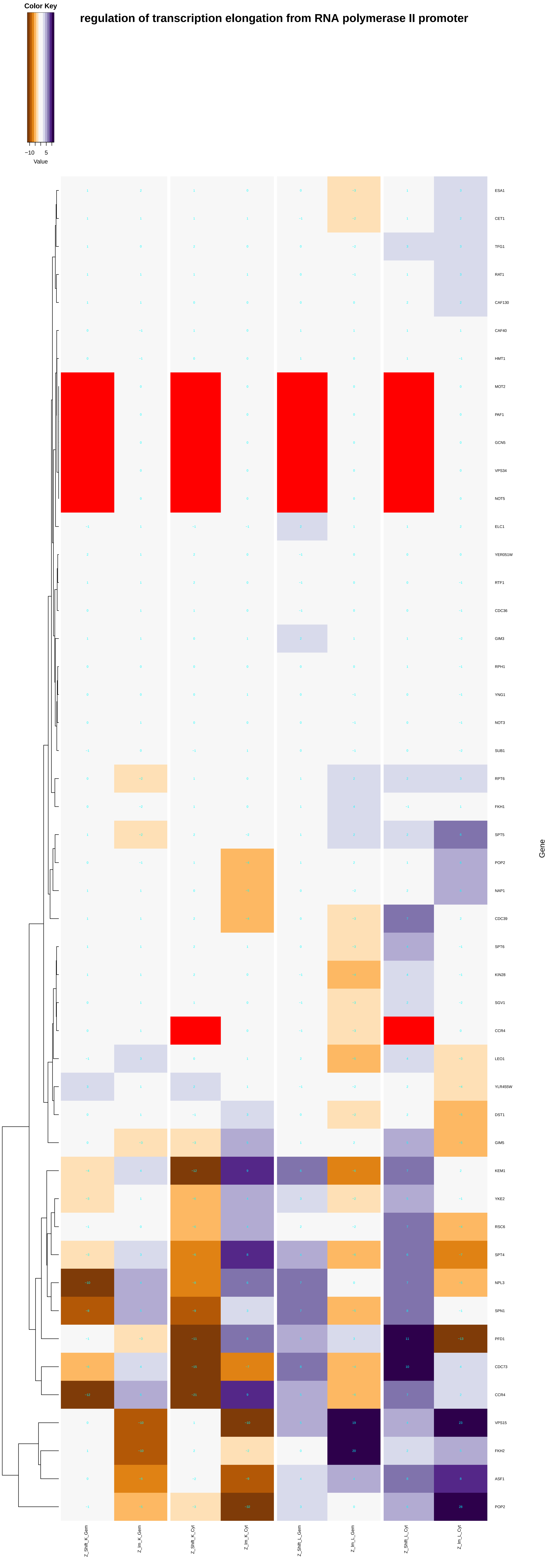

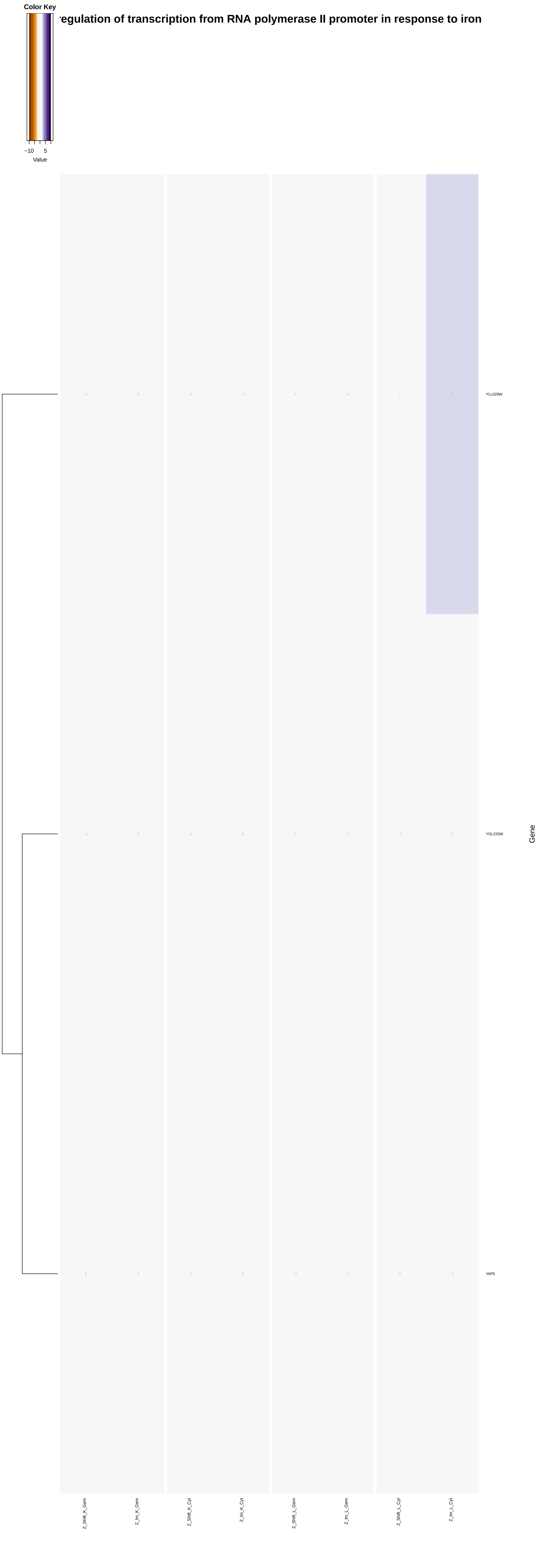

regulation of transcription from RNA polymerase II promoter in response to iron
Color Key
−10
5
Value
YLL029W
YGL220W
YAP5
Gene
Z_Shift_K_Gem
Z_Im_K_Gem
Z_Shift_K_Cyt
Z_Im_K_Cyt
Z_Shift_L_Gem
Z_Im_L_Gem
Z_Shift_L_Cyt
Z_Im_L_Cyt

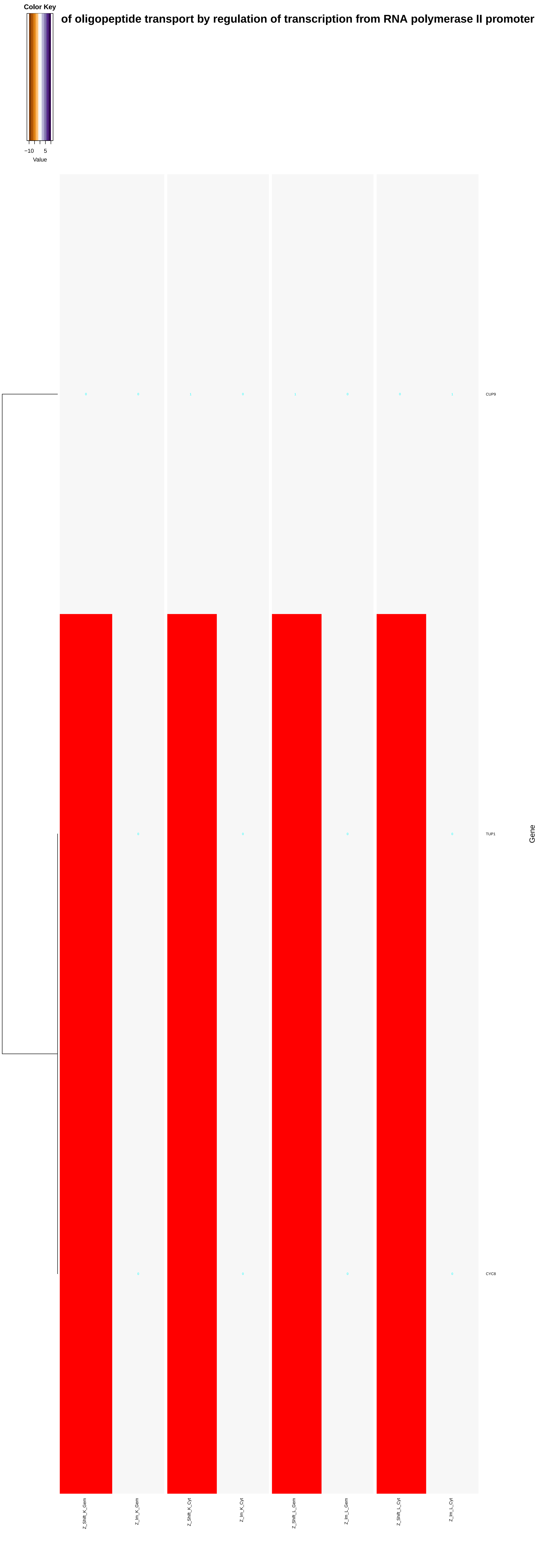
Color Key
−10
5
Value
of oligopeptide transport by regulation of transcription from RNA polymerase II promoter
CUP9
TUP1
CYC8
Gene
Z_Shift_K_Gem
Z_Im_K_Gem
Z_Shift_K_Cyt
Z_Im_K_Cyt
Z_Shift_L_Gem
Z_Im_L_Gem
Z_Shift_L_Cyt
Z_Im_L_Cyt

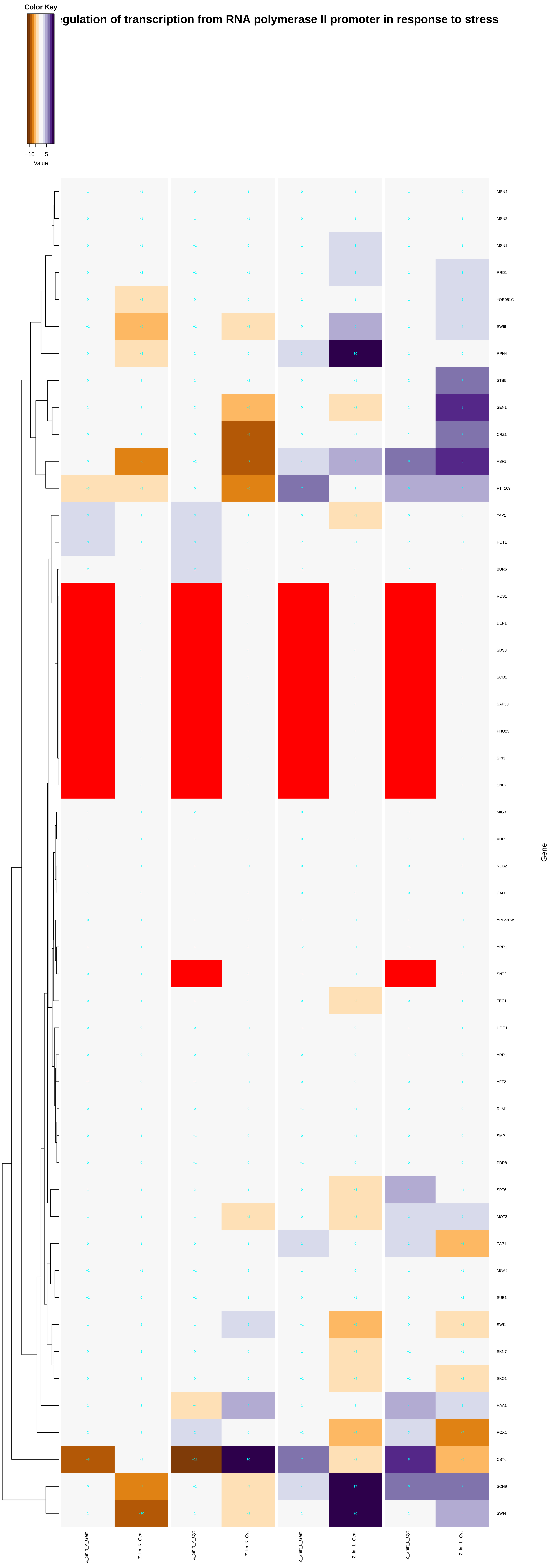

Color Key
−10
5
Value
egulation of transcription from RNA polymerase II promoter in response to stress
Gene
Z_Shift_K_Gem
Z_Im_K_Gem
Z_Shift_K_Cyt
Z_Im_K_Cyt
Z_Shift_L_Gem
Z_Im_L_Gem
Z_Shift_L_Cyt
Z_Im_L_Cyt
MSN4
MSN2
MSN1
RRD1
YOR051C
SWI6
RPN4
STB5
SEN1
CRZ1
ASF1
RTT109
YAP1
HOT1
BUR6
RCS1
DEP1
SDS3
SOD1
SAP30
PHO23
SIN3
SNF2
MIG3
VHR1
NCB2
CAD1
YPL230W
YRR1
SNT2
TEC1
HOG1
ARR1
AFT2
RLM1
SMP1
PDR8
SPT6
MOT3
ZAP1
MGA2
SUB1
SWI1
SKN7
SKO1
HAA1
ROX1
CST6
SCH9
SWI4

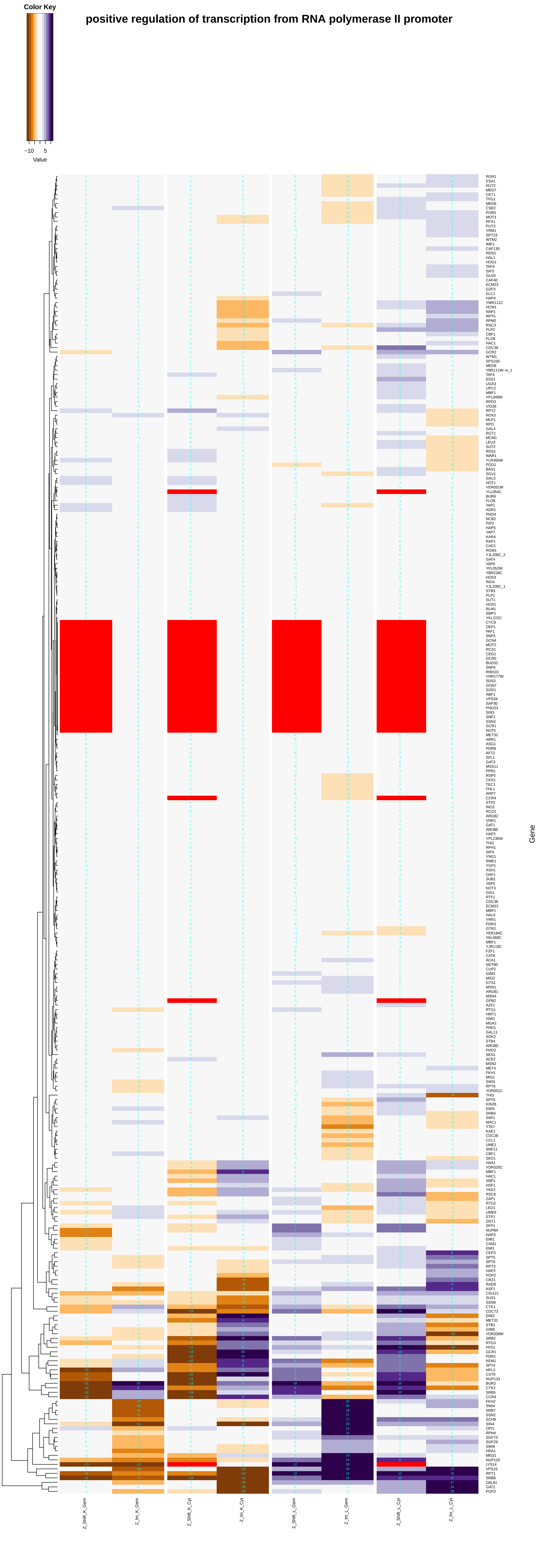
positive regulation of transcription from RNA polymerase II promoter
Color Key
−10
5
Value
Gene
Z_Shift_K_Gem
Z_Im_K_Gem
Z_Shift_K_Cyt
Z_Im_K_Cyt
Z_Shift_L_Gem
Z_Im_L_Gem
Z_Shift_L_Cyt
Z_Im_L_Cyt

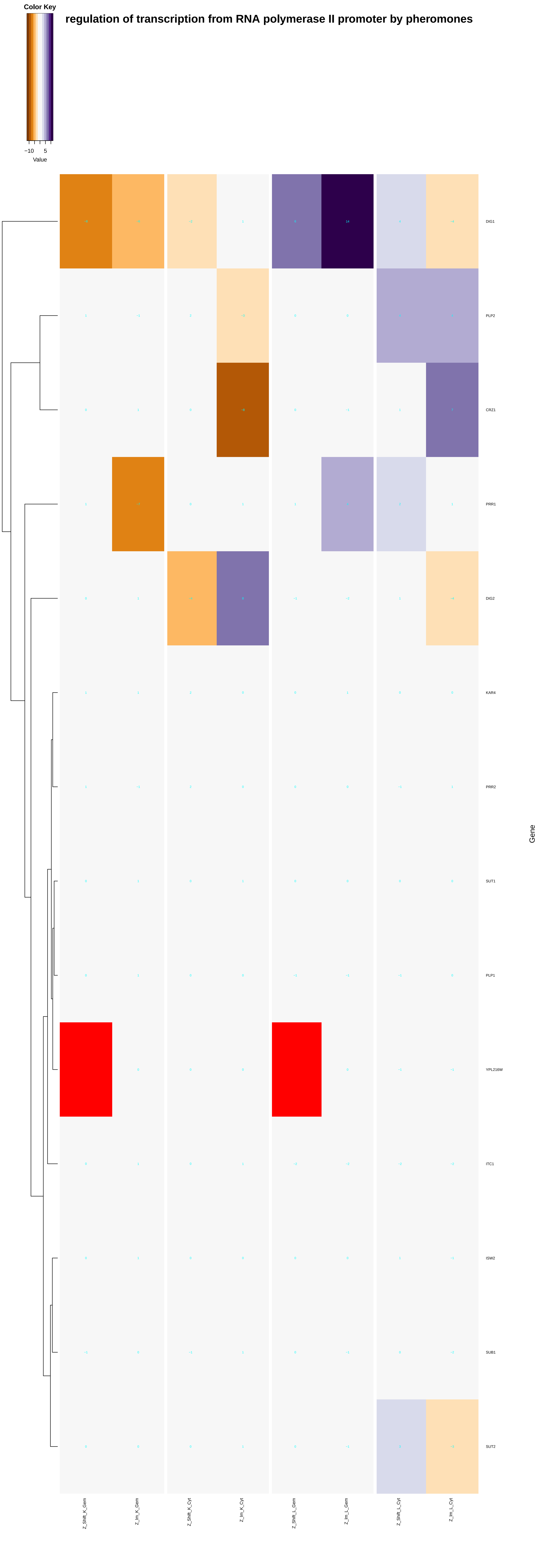
Color Key
−10 5
Value
regulation of transcription from RNA polymerase II promoter by pheromones
DIG1
PLP2
CRZ1
PRR1
DIG2
KAR4
PRR2
SUT1
PLP1
YPL216W
ITC1
ISW2
SUB1
SUT2
Gene
Z_Shift_K_Gem
Z_Im_K_Gem
Z_Shift_K_Cyt
Z_Im_K_Cyt
Z_Shift_L_Gem
Z_Im_L_Gem
Z_Shift_L_Cyt
Z_Im_L_Cyt

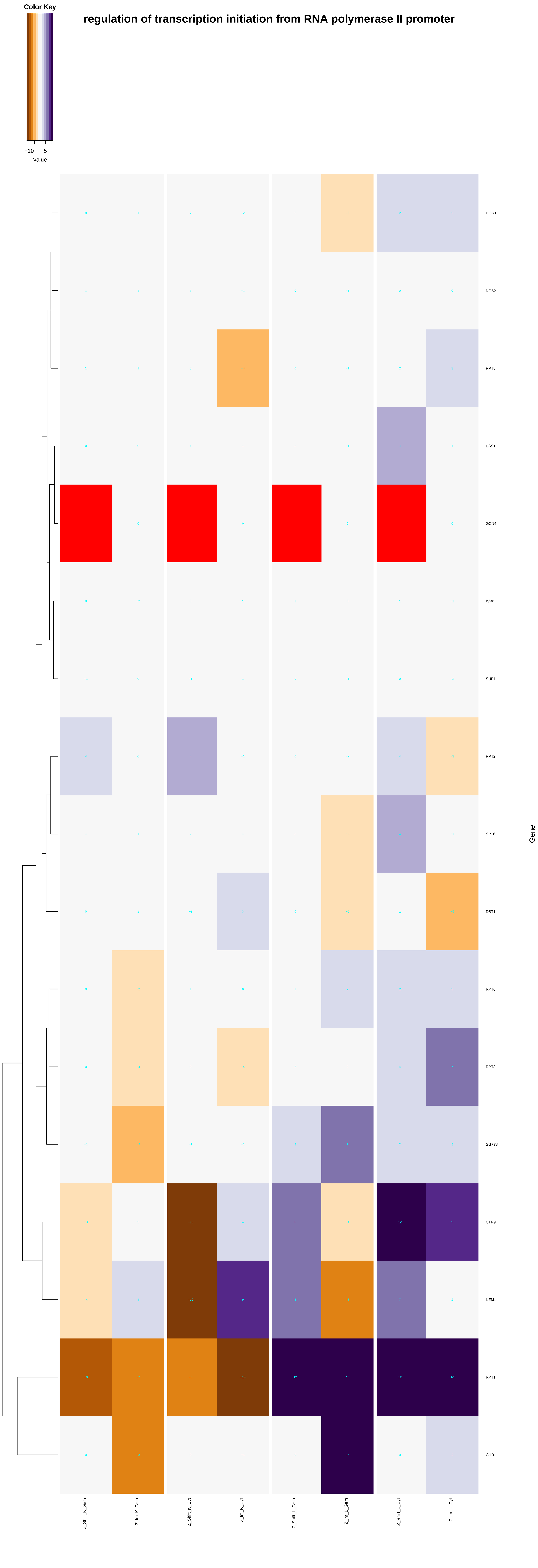
Color Key
−10
5
Value
regulation of transcription initiation from RNA polymerase II promoter
POB3
NCB2
RPT5
ESS1
GCN4
ISW1
SUB1
RPT2
SPT6
DST1
RPT6
RPT3
SGF73
CTR9
KEM1
RPT1
CHD1
Gene
Z_Shift_K_Gem
Z_Im_K_Gem
Z_Shift_K_Cyt
Z_Im_K_Cyt
Z_Shift_L_Gem
Z_Im_L_Gem
Z_Shift_L_Cyt
Z_Im_L_Cyt

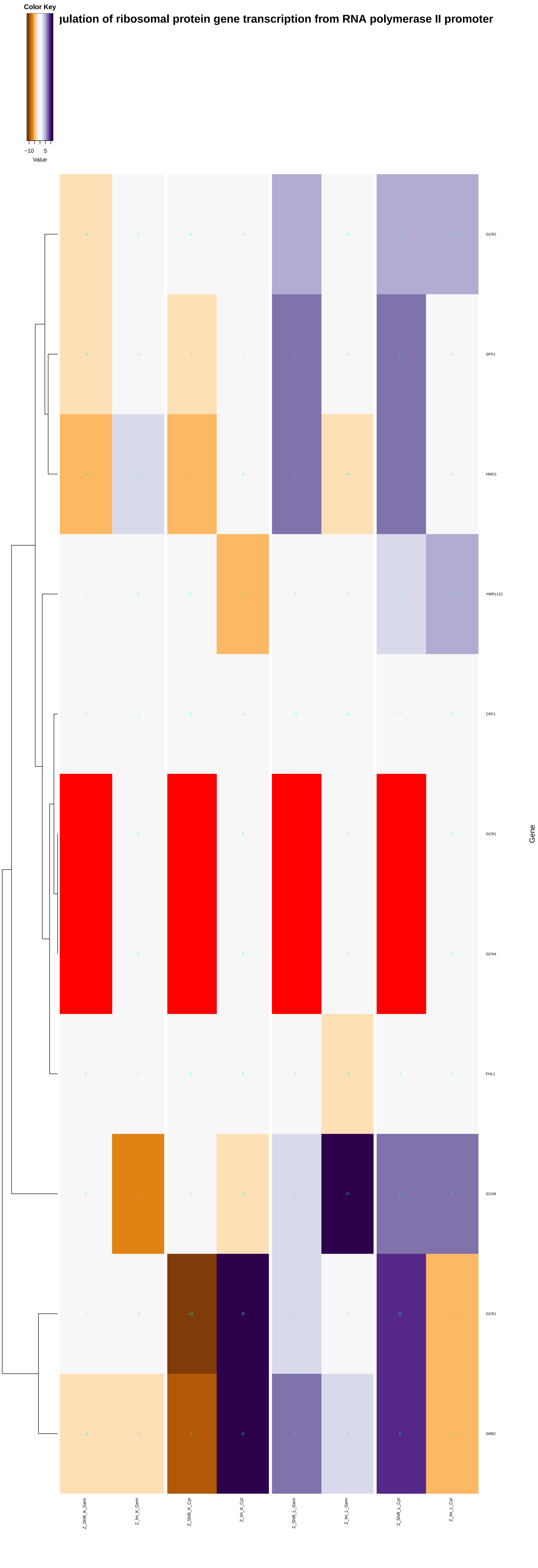
ulation of ribosomal protein gene transcription from RNA polymerase II promoter
Color Key
−10
5
Value
GCR2
SFP1
HMO1
YMR111C
CRF1
GCR1
GCN4
FHL1
SCH9
GCR1
SRB2
Gene
Z_Shift_K_Gem
Z_Im_K_Gem
Z_Shift_K_Cyt
Z_Im_K_Cyt
Z_Shift_L_Gem
Z_Im_L_Gem
Z_Shift_L_Cyt
Z_Im_L_Cyt

phosphorylation of RNA polymerase II C−terminal domain

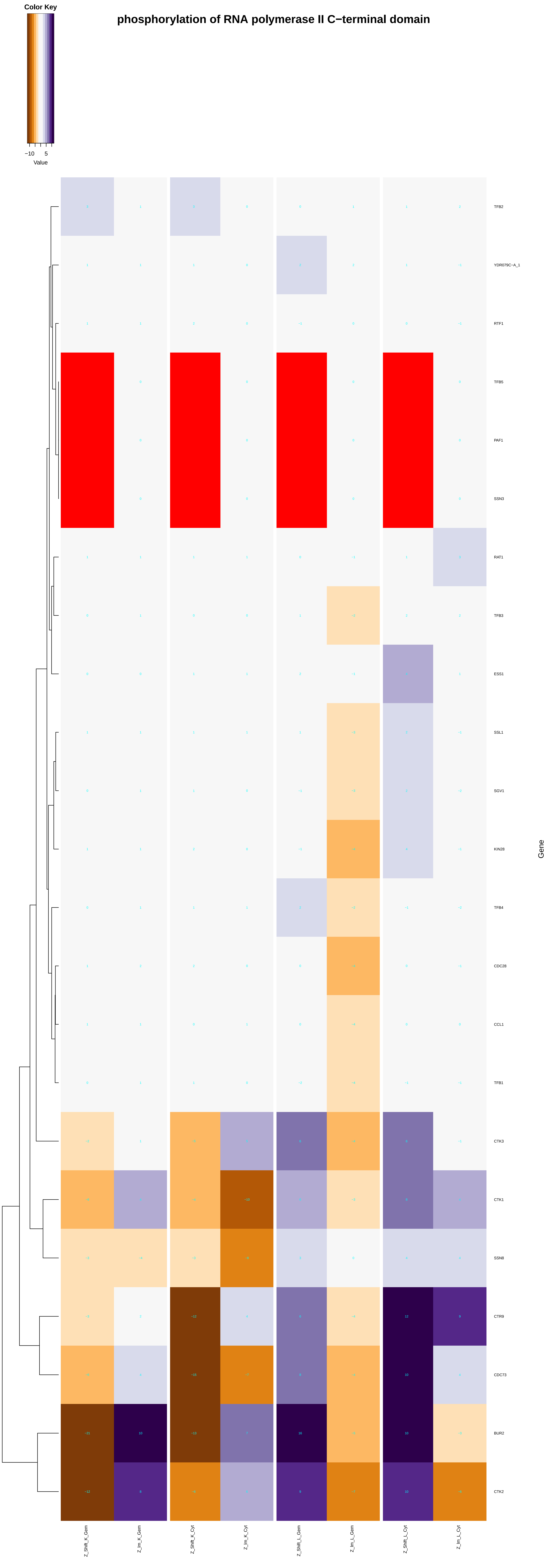

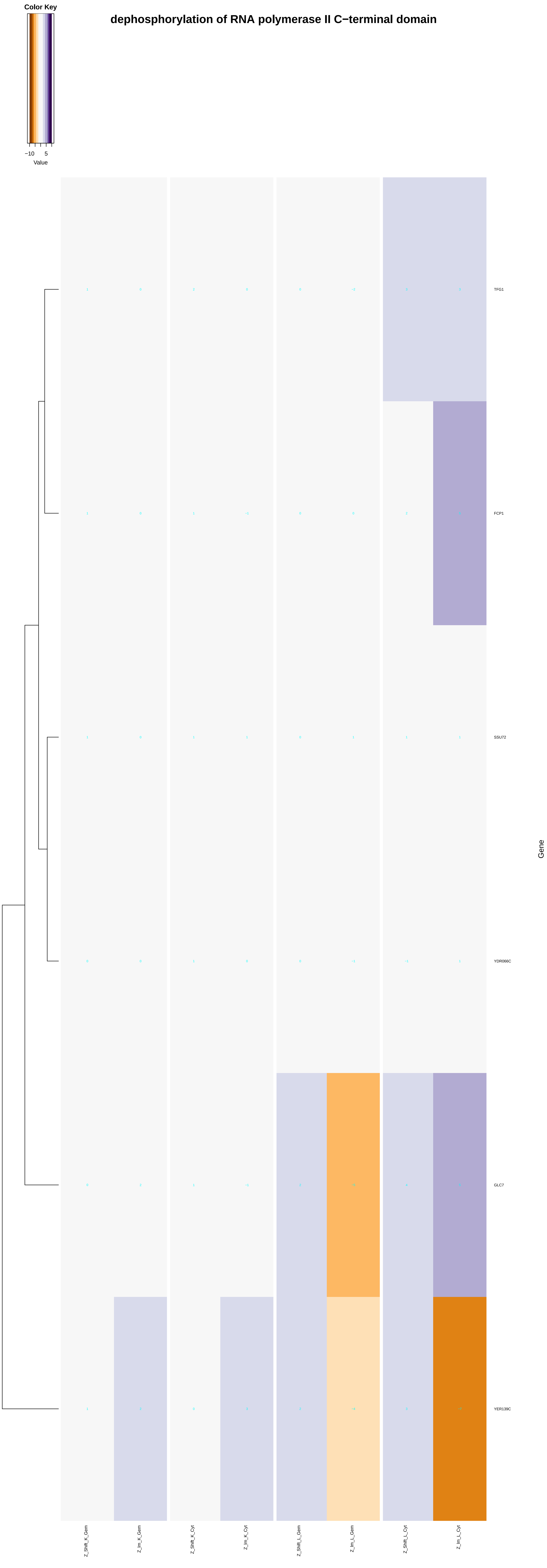
dephosphorylation of RNA polymerase II C−terminal domain
Color Key
−10
5
Value
TFG1
FCP1
SSU72
YDR066C
GLC7
YER139C
Gene
Z_Shift_K_Gem
Z_Im_K_Gem
Z_Shift_K_Cyt
Z_Im_K_Cyt
Z_Shift_L_Gem
Z_Im_L_Gem
Z_Shift_L_Cyt
Z_Im_L_Cyt

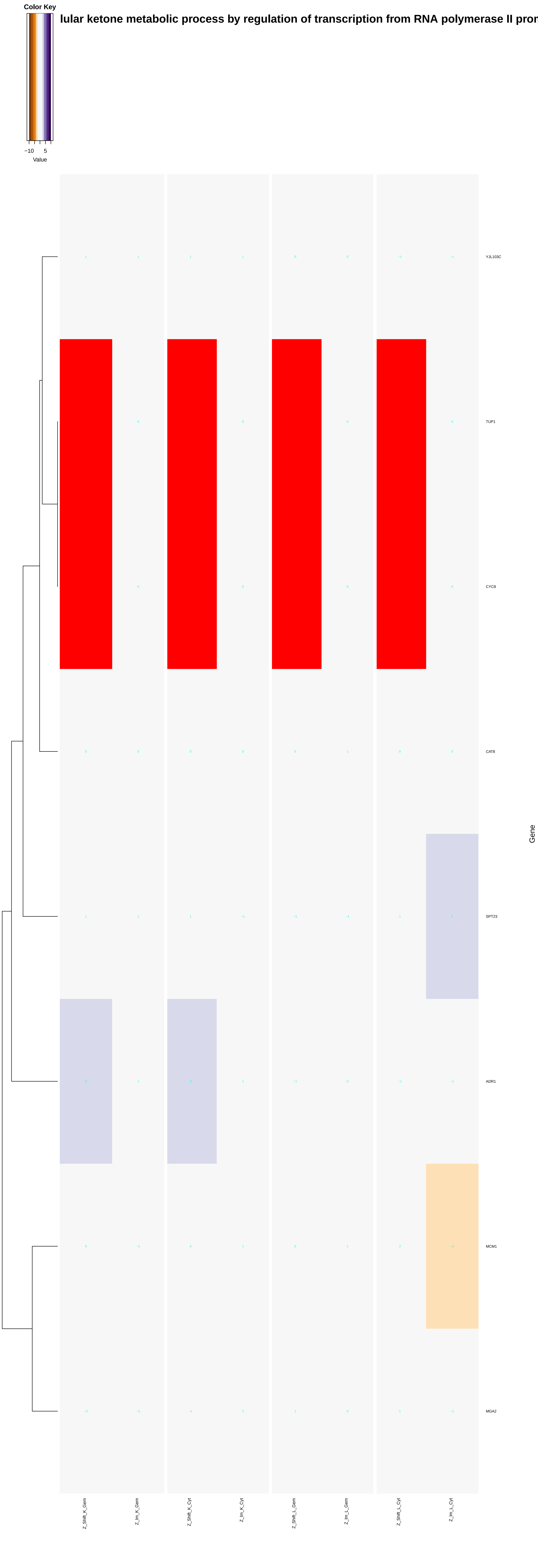

Color Key
−10
5
Value
lular ketone metabolic process by regulation of transcription from RNA polymerase II pron
YJL103C
TUP1
CYC8
CAT8
SPT23
ADR1
MCM1
MGA2
Gene
Z_Shift_K_Gem
Z_Im_K_Gem
Z_Shift_K_Cyt
Z_Im_K_Cyt
Z_Shift_L_Gem
Z_Im_L_Gem
Z_Shift_L_Cyt
Z_Im_L_Cyt

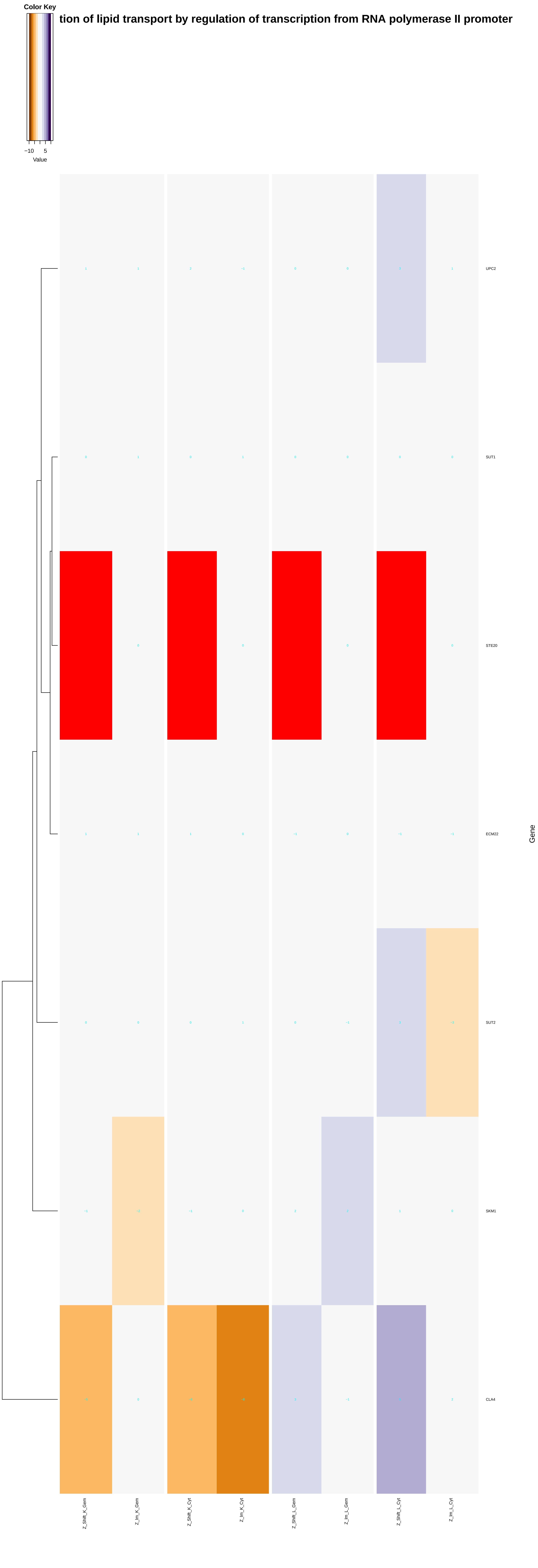

Color Key
−10
5
Value
tion of lipid transport by regulation of transcription from RNA polymerase II promoter
UPC2
SUT1
STE20
ECM22
SUT2
SKM1
CLA4
Gene
Z_Shift_K_Gem
Z_Im_K_Gem
Z_Shift_K_Cyt
Z_Im_K_Cyt
Z_Shift_L_Gem
Z_Im_L_Gem
Z_Shift_L_Cyt
Z_Im_L_Cyt

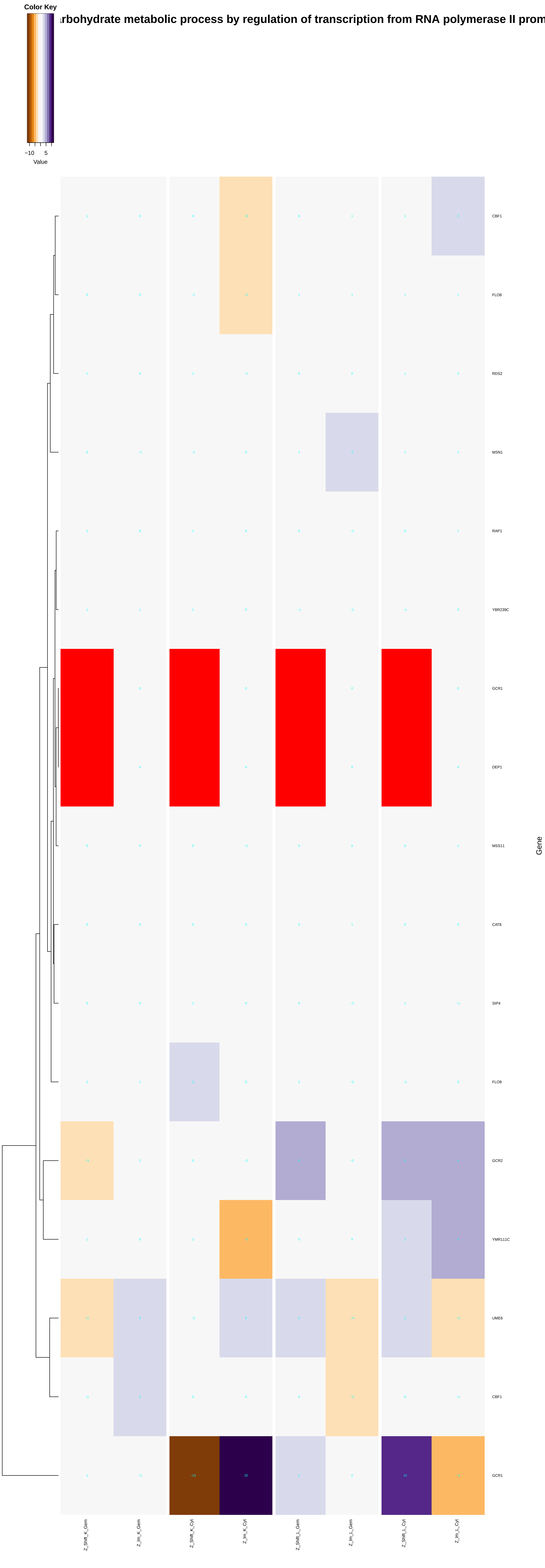

arbohydrate metabolic process by regulation of transcription from RNA polymerase II prom
Color Key
−10
5
Value
CBF1
FLO8
RDS2
MSN1
RAP1
YBR239C
GCR1
DEP1
MSS11
CAT8
SIP4
FLO8
GCR2
YMR111C
UME6
CBF1
GCR1
Gene
Z_Shift_K_Gem
Z_Im_K_Gem
Z_Shift_K_Cyt
Z_Im_K_Cyt
Z_Shift_L_Gem
Z_Im_L_Gem
Z_Shift_L_Cyt
Z_Im_L_Cyt

regulation of phosphorylation of RNA polymerase II C−terminal domain

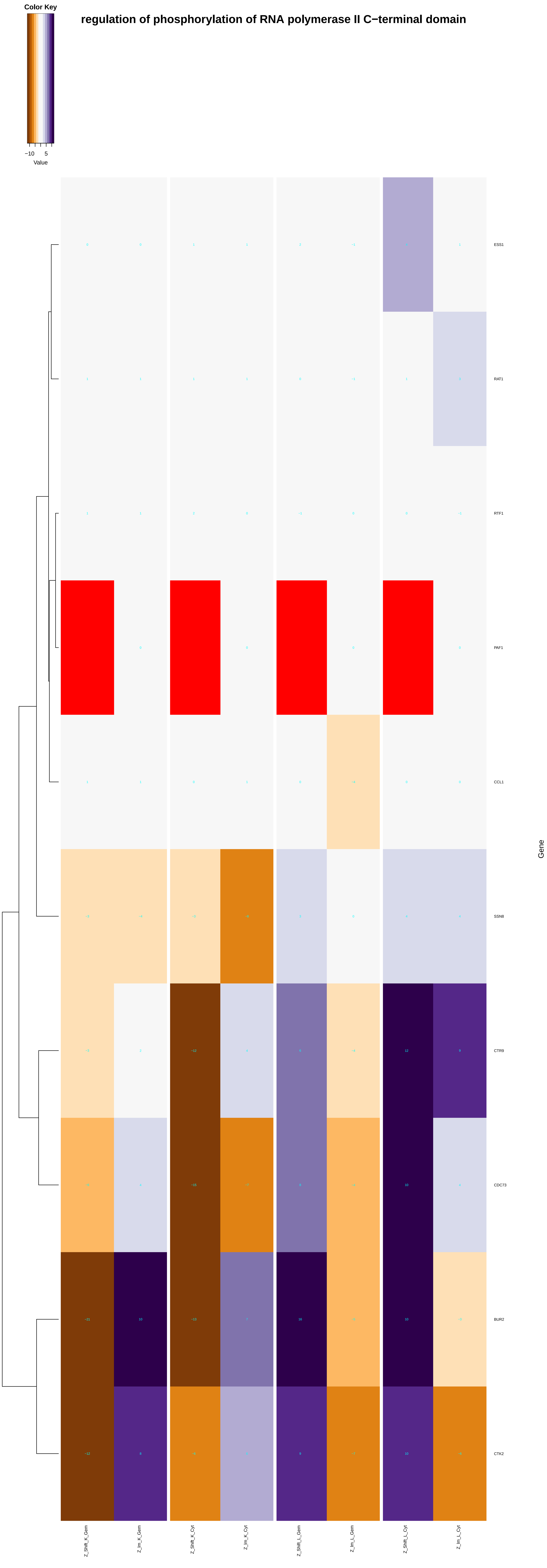

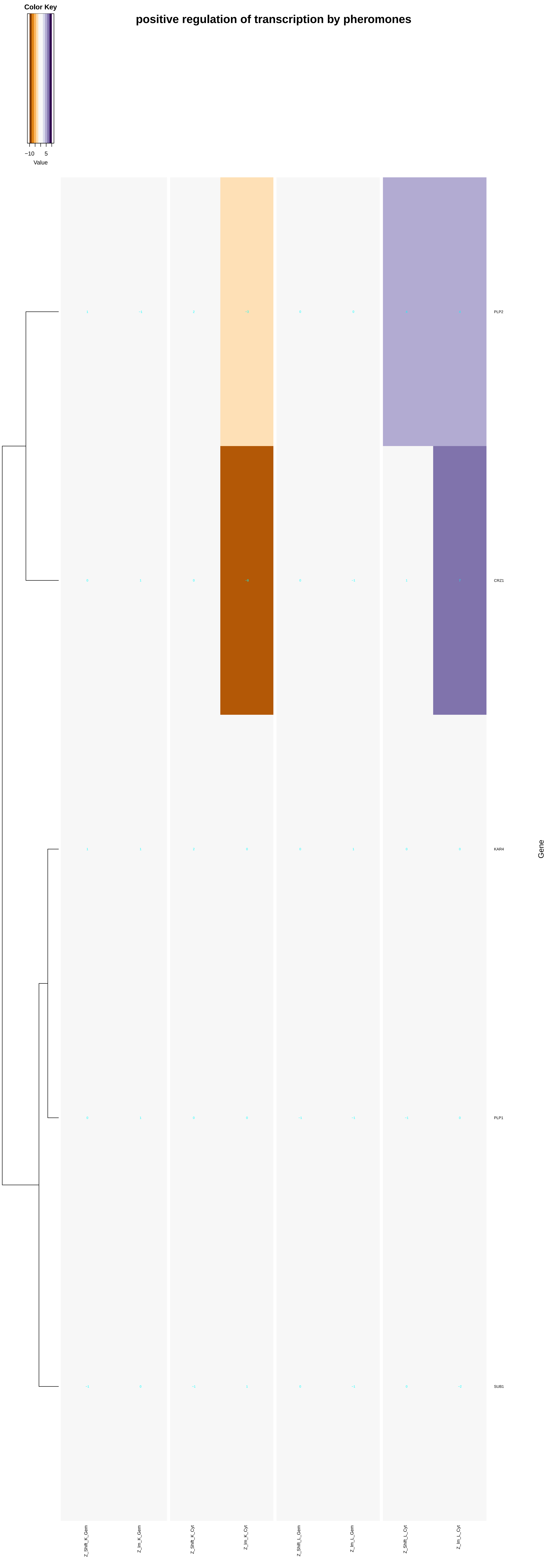
positive regulation of transcription by pheromones
Color Key
−10
5
Value
PLP2
CRZ1
KAR4
PLP1
SUB1
Gene
Z_Shift_K_Gem
Z_Im_K_Gem
Z_Shift_K_Cyt
Z_Im_K_Cyt
Z_Shift_L_Gem
Z_Im_L_Gem
Z_Shift_L_Cyt
Z_Im_L_Cyt

negative regulation of transcription by pheromones

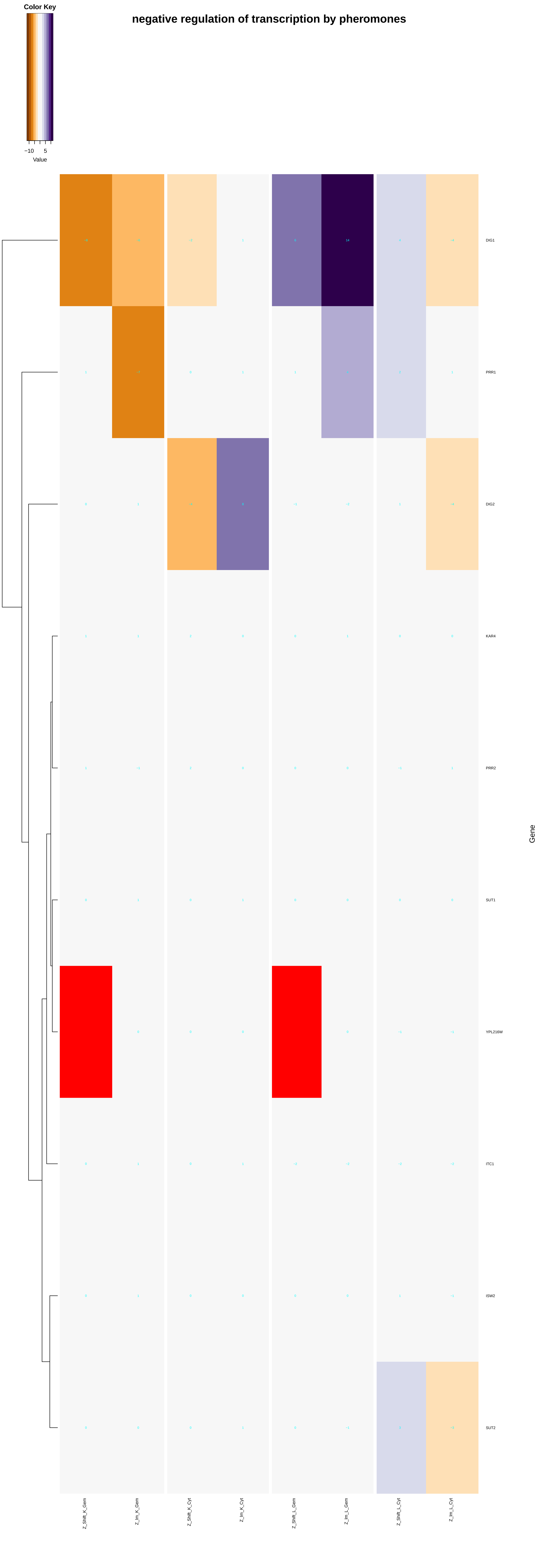

negative regulation of chromatin silencing

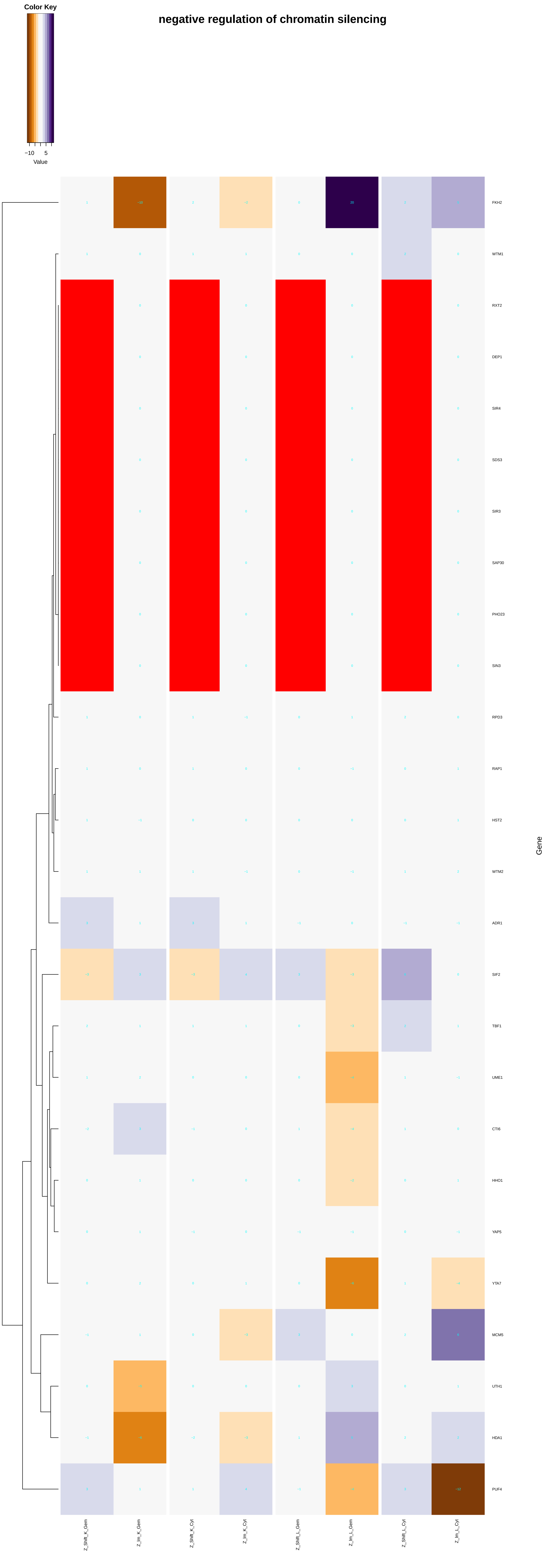

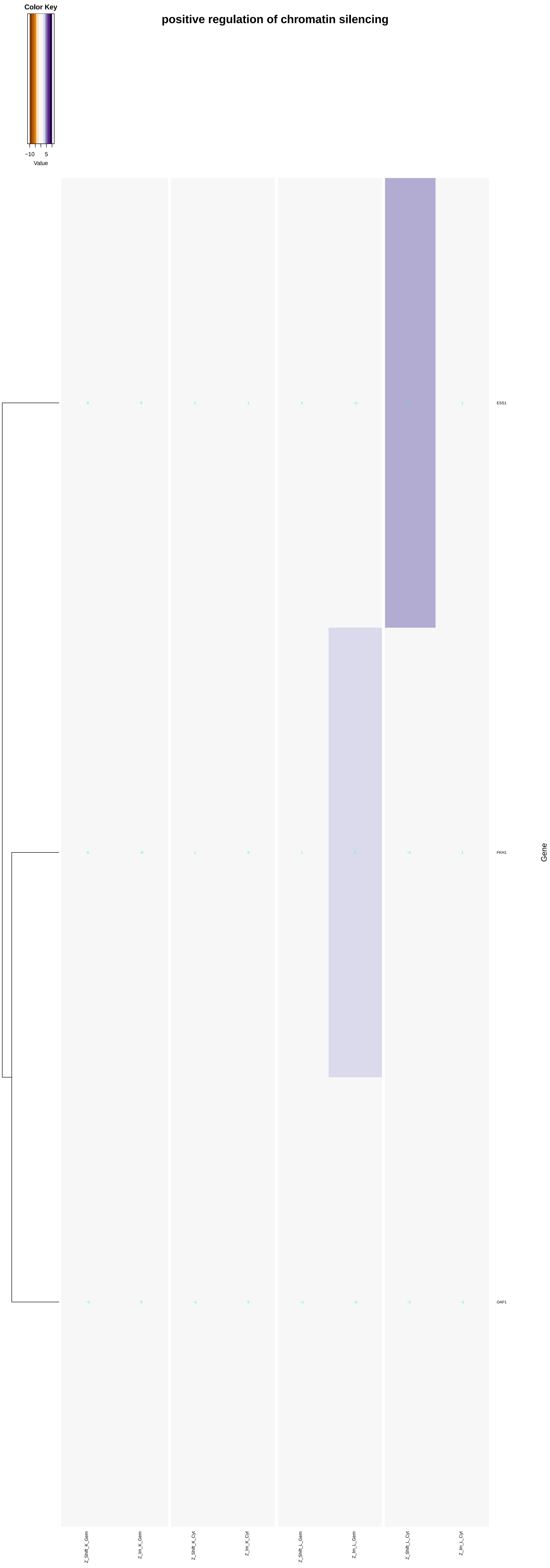

positive regulation of chromatin silencing
Color Key
−10
5
Value
ESS1
FKH1
OAF1
Gene
Z_Shift_K_Gem
Z_Im_K_Gem
Z_Shift_K_Cyt
Z_Im_K_Cyt
Z_Shift_L_Gem
Z_Im_L_Gem
Z_Shift_L_Cyt
Z_Im_L_Cyt

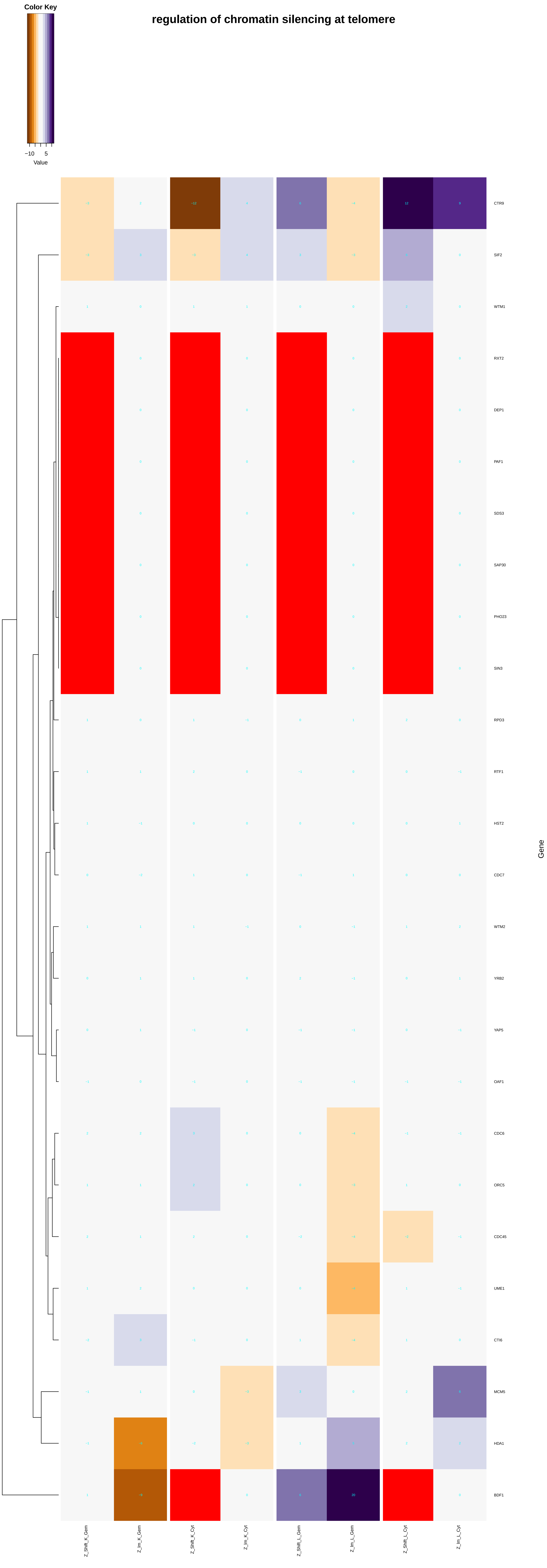

regulation of chromatin silencing at telomere
Color Key
−10
5
Value
CTR9
SIF2
WTM1
RXT2
DEP1
PAF1
SDS3
SAP30
PHO23
SIN3
RPD3
RTF1
HST2
CDC7
WTM2
YRB2
YAP5
OAF1
CDC6
ORC5
CDC45
UME1
CTI6
MCM5
HDA1
BDF1
Gene
Z_Shift_K_Gem
Z_lm_K_Gem
Z_Shift_K_Cyt
Z_lm_K_Cyt
Z_Shift_L_Gem
Z_lm_L_Gem
Z_Shift_L_Cyt
Z_lm_L_Cyt

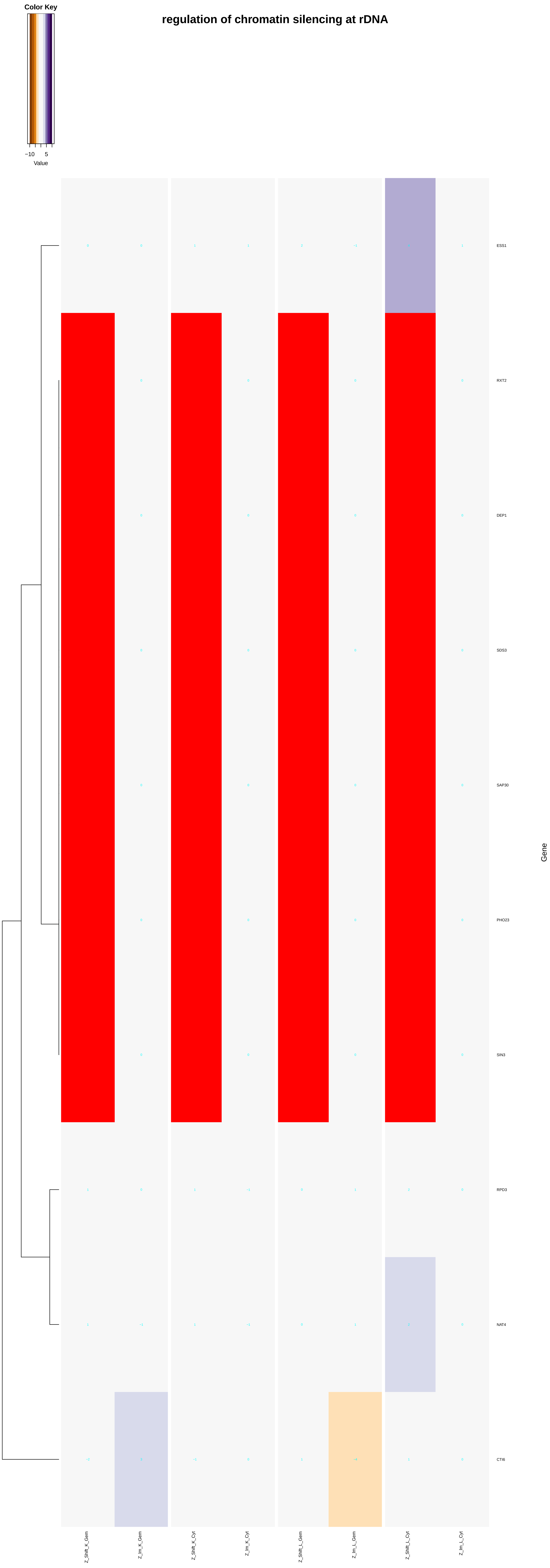

Color Key
−10 5
Value
regulation of chromatin silencing at rDNA
ESS1
RXT2
DEP1
SDS3
SAP30
PHO23
SIN3
RPD3
NAT4
CTI6
Gene
Z_Shift_K_Gem
Z_Int_K_Gem
Z_Shift_K_Cyt
Z_Int_K_Cyt
Z_Shift_L_Gem
Z_Int_L_Gem
Z_Shift_L_Cyt
Z_Int_L_Cyt

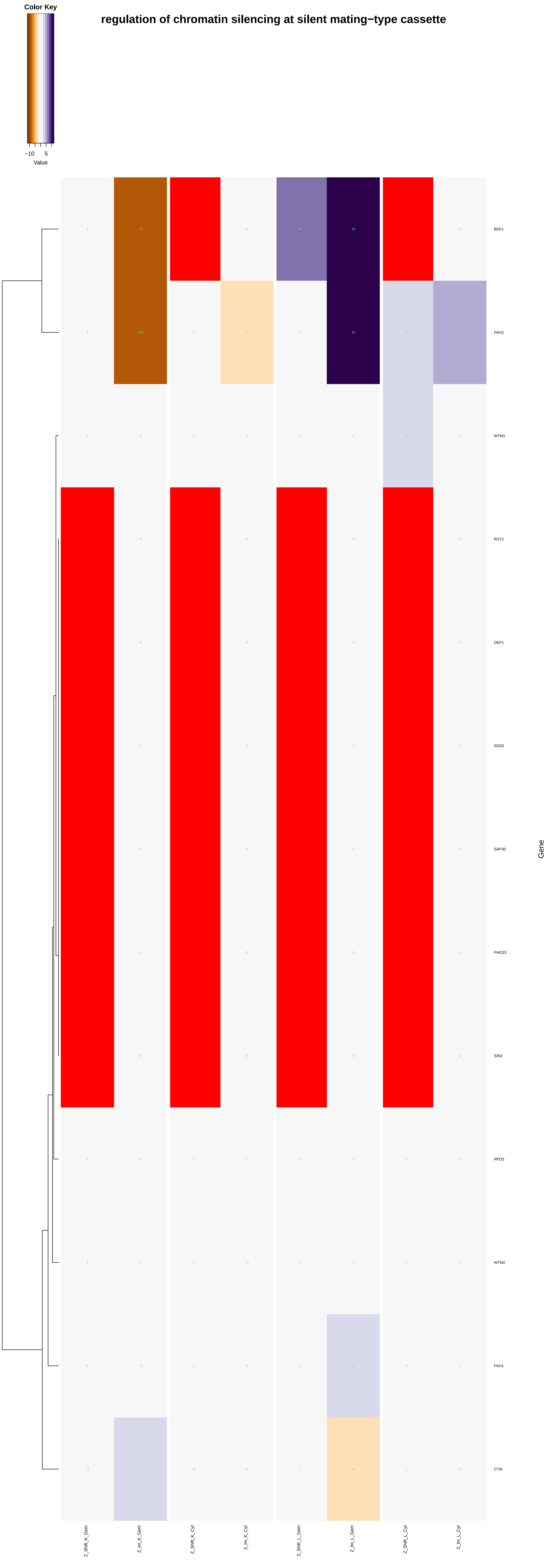
regulation of chromatin silencing at silent mating−type cassette
Color Key
−10
5
Value
BDF1
FKH2
WTM1
RXT2
DEP1
SDS3
SAP30
PHO23
SIN3
RPD3
WTM2
FKH1
CTI6
Gene
Z_Shift_K_Gem
Z_Im_K_Gem
Z_Shift_K_Cyt
Z_Im_K_Cyt
Z_Shift_L_Gem
Z_Im_L_Gem
Z_Shift_L_Cyt
Z_Im_L_Cyt

negative regulation of DNA−templated transcription, elongation

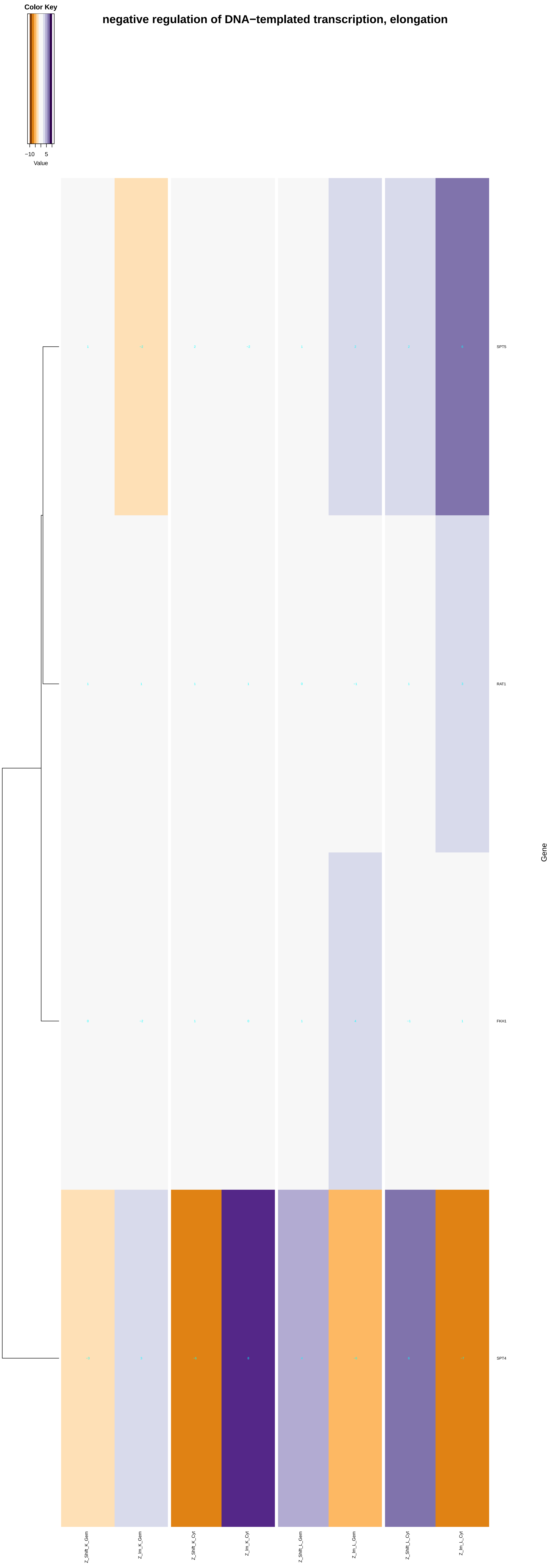

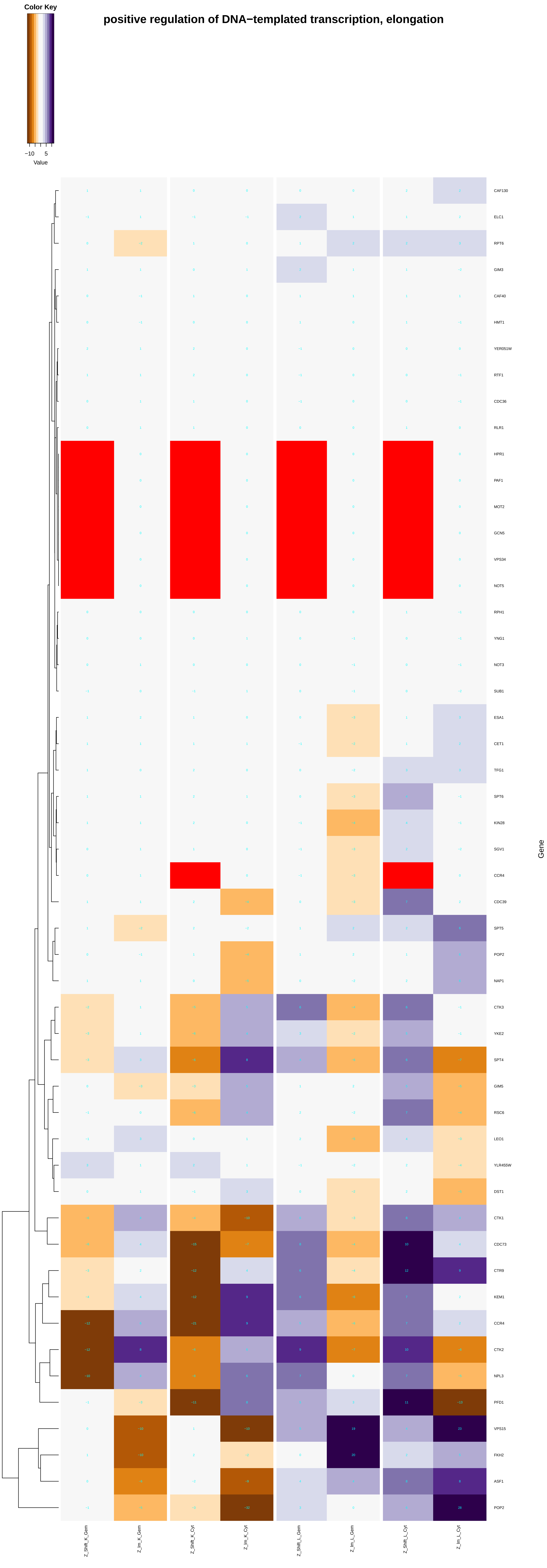
positive regulation of DNA−templated transcription, elongation
Color Key
−10
5
Value
Gene
CAF130
ELC1
RPT6
GIM3
CAF40
HMT1
YER051W
RTF1
CDC36
RLR1
HPR1
PAF1
MOT2
GCN5
VPS34
NOT5
RPH1
YNG1
NOT3
SUB1
ESA1
CET1
TFG1
SPT6
KIN28
SGV1
CCR4
CDC39
SPT5
POP2
NAP1
CTK3
YKE2
SPT4
GIM5
RSC6
LEO1
YLR455W
DST1
CTK1
CDC73
CTR9
KEM1
CCR4
CTK2
NPL3
PFD1
VPS15
FKH2
ASF1
POP2
Z_Shift_K_Gem
Z_Im_K_Gem
Z_Shift_K_Cyt
Z_Im_K_Cyt
Z_Shift_L_Gem
Z_Im_L_Gem
Z_Shift_L_Cyt
Z_Im_L_Cyt

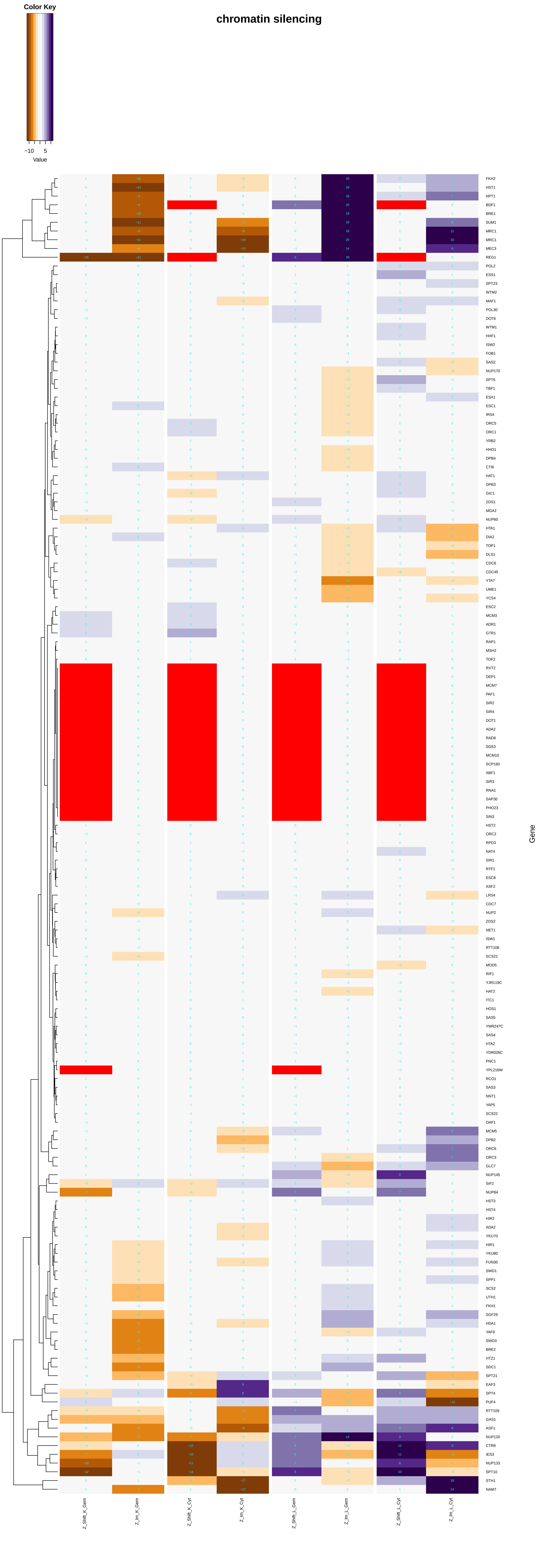
Color Key
−10
5
Value
chromatin silencing
Gene
Z_Shift_K_Gem
Z_Im_K_Gem
Z_Shift_K_Cyt
Z_Im_K_Cyt
Z_Shift_L_Gem
Z_Im_L_Gem
Z_Shift_L_Cyt
Z_Im_L_Cyt

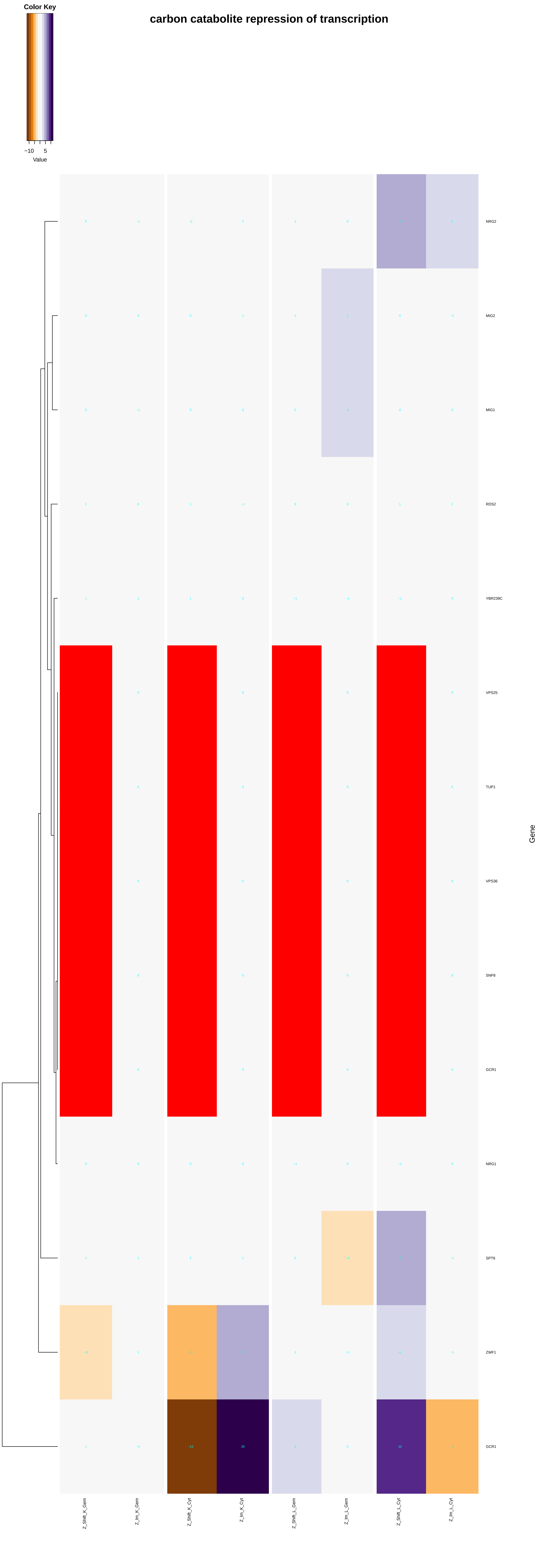

carbon catabolite repression of transcription
Color Key
−10
5
Value
NRG2
MIG2
MIG1
RDS2
YBR239C
VPS25
TUP1
VPS36
SNF8
GCR1
NRG1
SPT6
ZWF1
GCR1
Gene
Z_Shift_K_Gem
Z_Im_K_Gem
Z_Shift_K_Cyt
Z_Im_K_Cyt
Z_Shift_L_Gem
Z_Im_L_Gem
Z_Shift_L_Cyt
Z_Im_L_Cyt

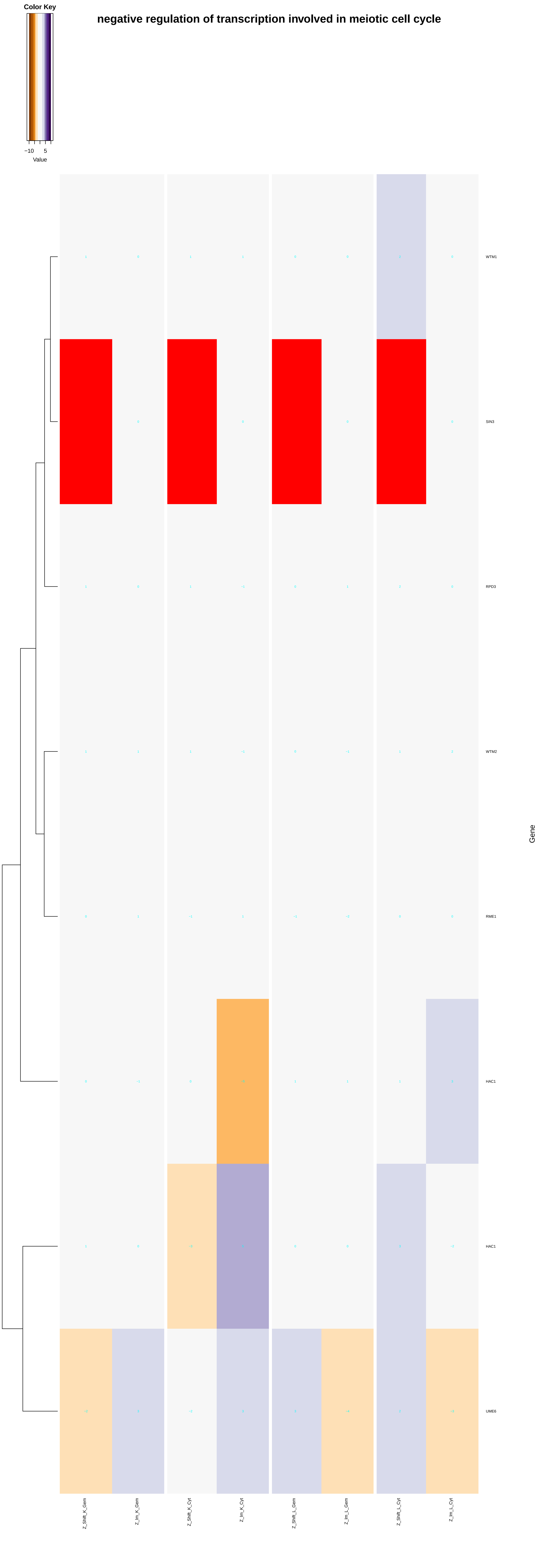

Color Key
−10
5
Value
negative regulation of transcription involved in meiotic cell cycle
WTM1
SIN3
RPD3
WTM2
RME1
HAC1
HAC1
UME6
Gene
Z_Shift_K_Gem
Z_Im_K_Gem
Z_Shift_K_Cyt
Z_Im_K_Cyt
Z_Shift_L_Gem
Z_Im_L_Gem
Z_Shift_L_Cyt
Z_Im_L_Cyt

negative regulation of antisense RNA transcription

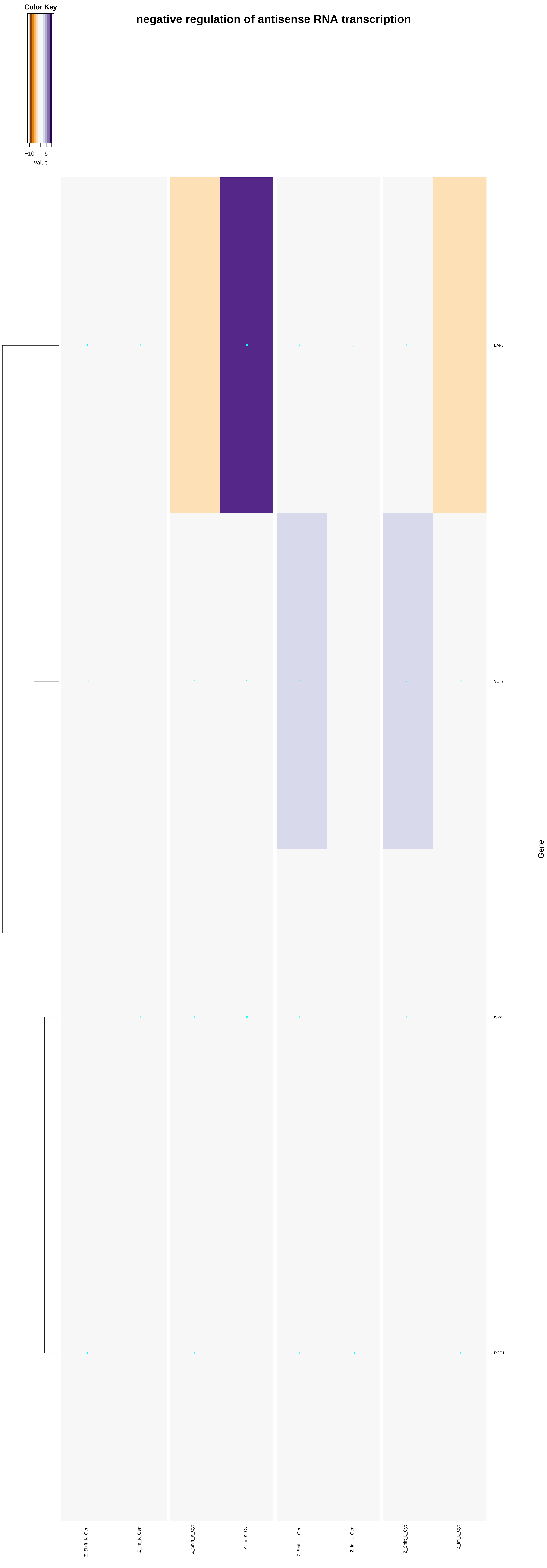

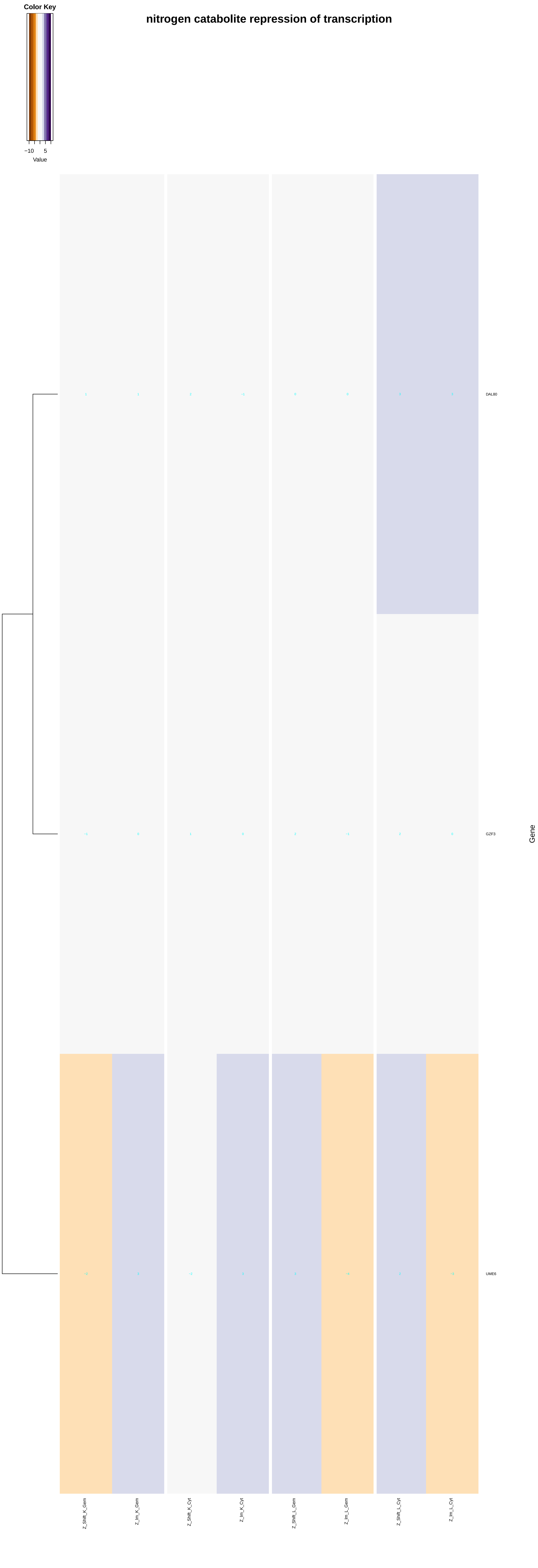
nitrogen catabolite repression of transcription
Color Key
−10
5
Value
DAL80
GZF3
UME6
Gene
Z_Shift_K_Gem
Z_Im_K_Gem
Z_Shift_K_Cyt
Z_Im_K_Cyt
Z_Shift_L_Gem
Z_Im_L_Gem
Z_Shift_L_Cyt
Z_Im_L_Cyt

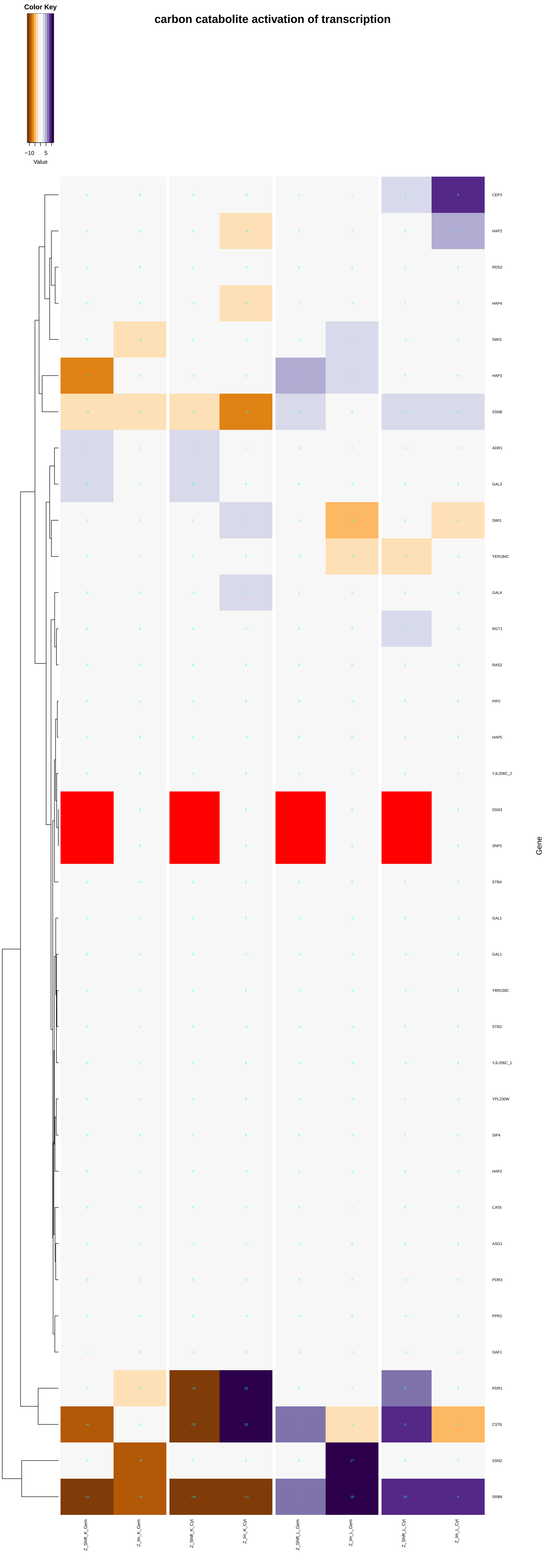
carbon catabolite activation of transcription
Color Key
−10
5
Value
CEP3
HAP2
RDS2
HAP4
SWI3
HAP3
SSN8
ADR1
GAL3
SWI1
YER184C
GAL4
RGT1
RAS2
PIP2
HAP5
YJL206C_2
SSN3
SNF5
STB4
GAL1
GAL1
YBR239C
STB3
YJL206C_1
YPL230W
SIP4
HAP2
CAT8
ASG1
PDR3
PPR1
OAF1
PDR1
CST6
SSN2
SRB8
Gene
Z_Shift_K_Gem
Z_Im_K_Gem
Z_Shift_K_Cyt
Z_Im_K_Cyt
Z_Shift_L_Gem
Z_Im_L_Gem
Z_Shift_L_Cyt
Z_Im_L_Cyt

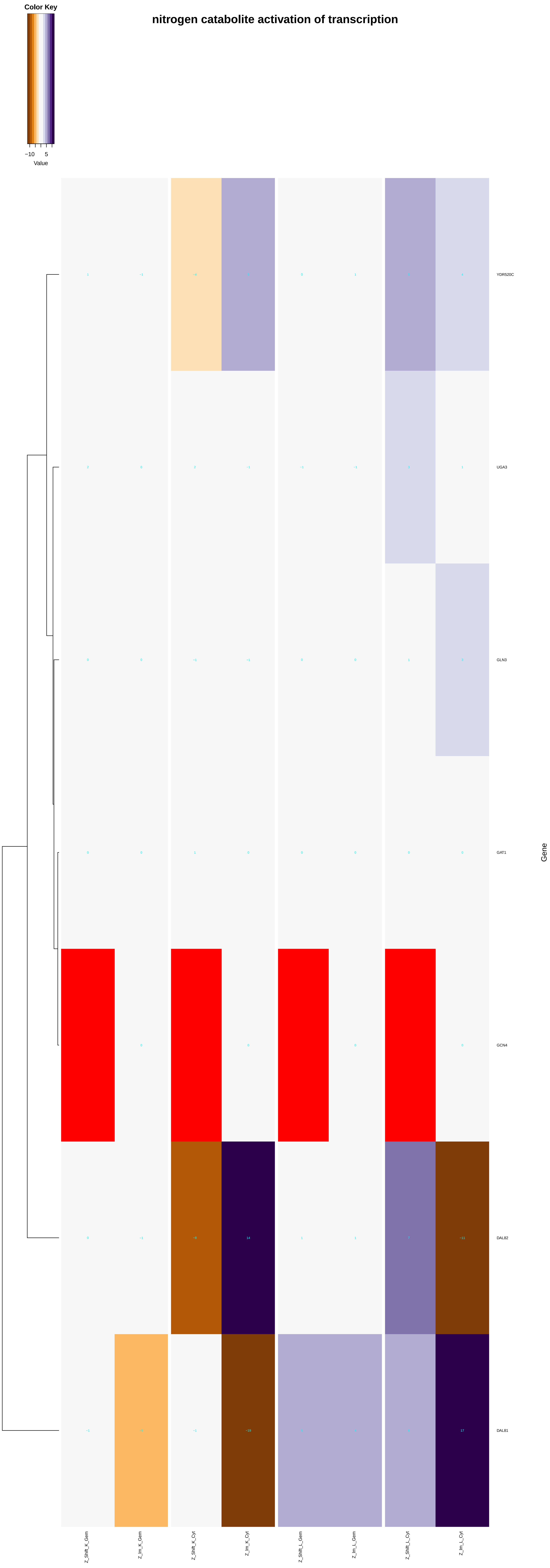
nitrogen catabolite activation of transcription
Color Key
−10
5
Value
YDR520C
UGA3
GLN3
GAT1
GCN4
DAL82
DAL81
Gene
Z_Shift_K_Gem
Z_Im_K_Gem
Z_Shift_K_Cyt
Z_Im_K_Cyt
Z_Shift_L_Gem
Z_Im_L_Gem
Z_Shift_L_Cyt
Z_Im_L_Cyt

positive regulation of DNA−templated transcription, initiation

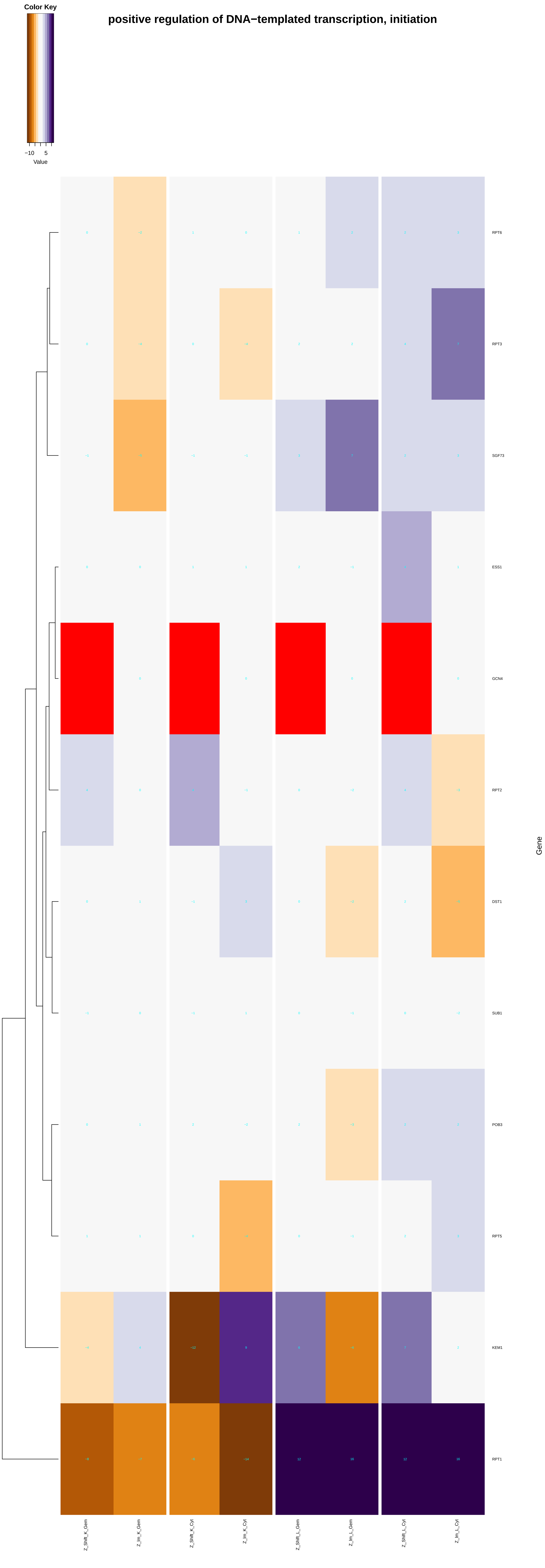

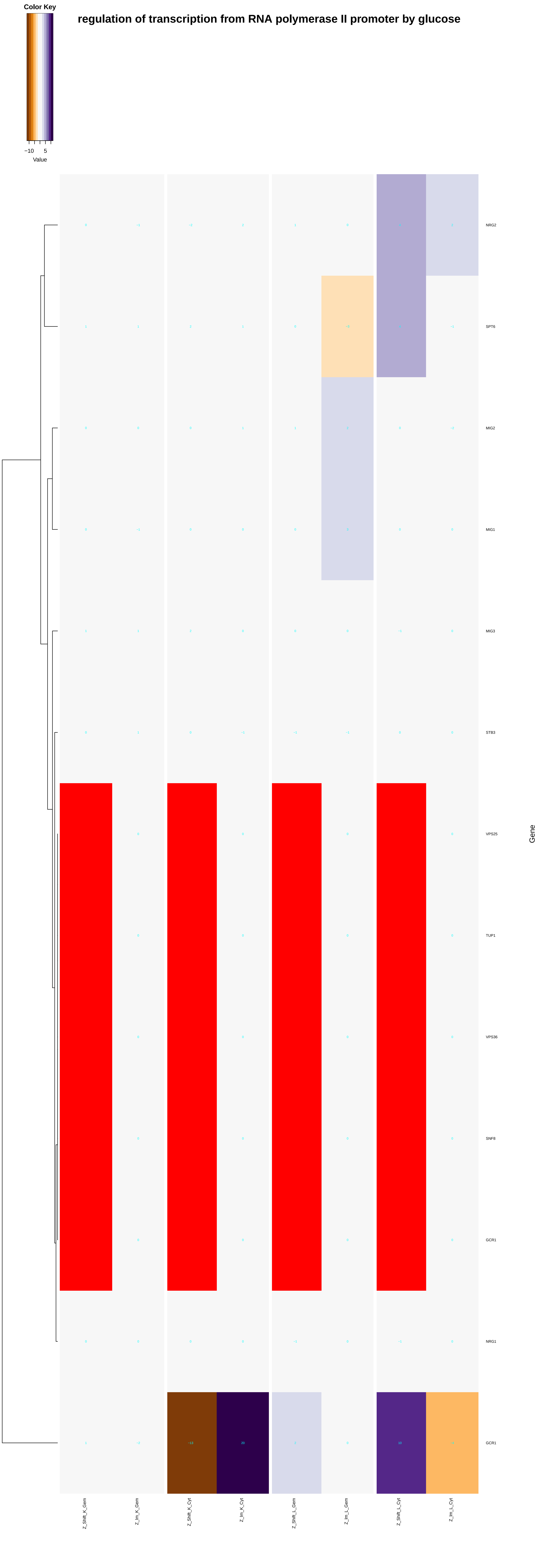

regulation of transcription from RNA polymerase II promoter by glucose
Color Key
−10
5
Value
NRG2
SPT6
MIG2
MIG1
MIG3
STB3
VPS25
TUP1
VPS36
SNF8
GCR1
NRG1
GCR1
Gene
Z_Shift_K_Gem
Z_Im_K_Gem
Z_Shift_K_Cyt
Z_Im_K_Cyt
Z_Shift_L_Gem
Z_Im_L_Gem
Z_Shift_L_Cyt
Z_Im_L_Cyt

negative regulation of transcription by glucose

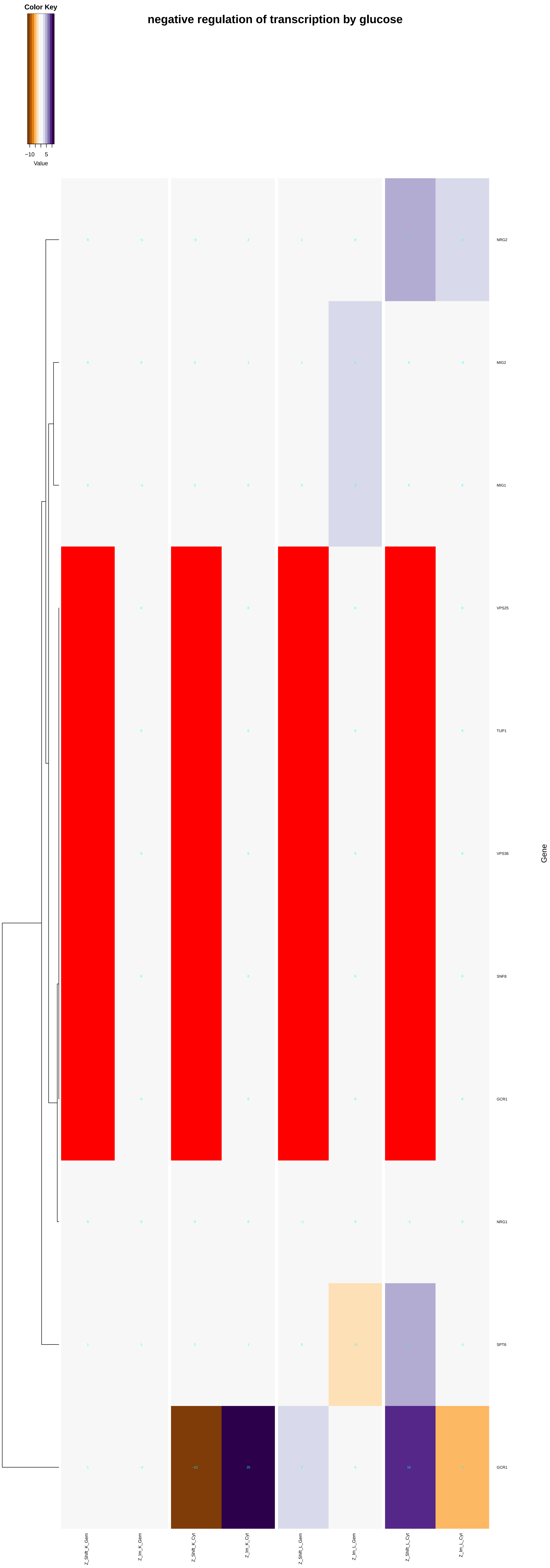

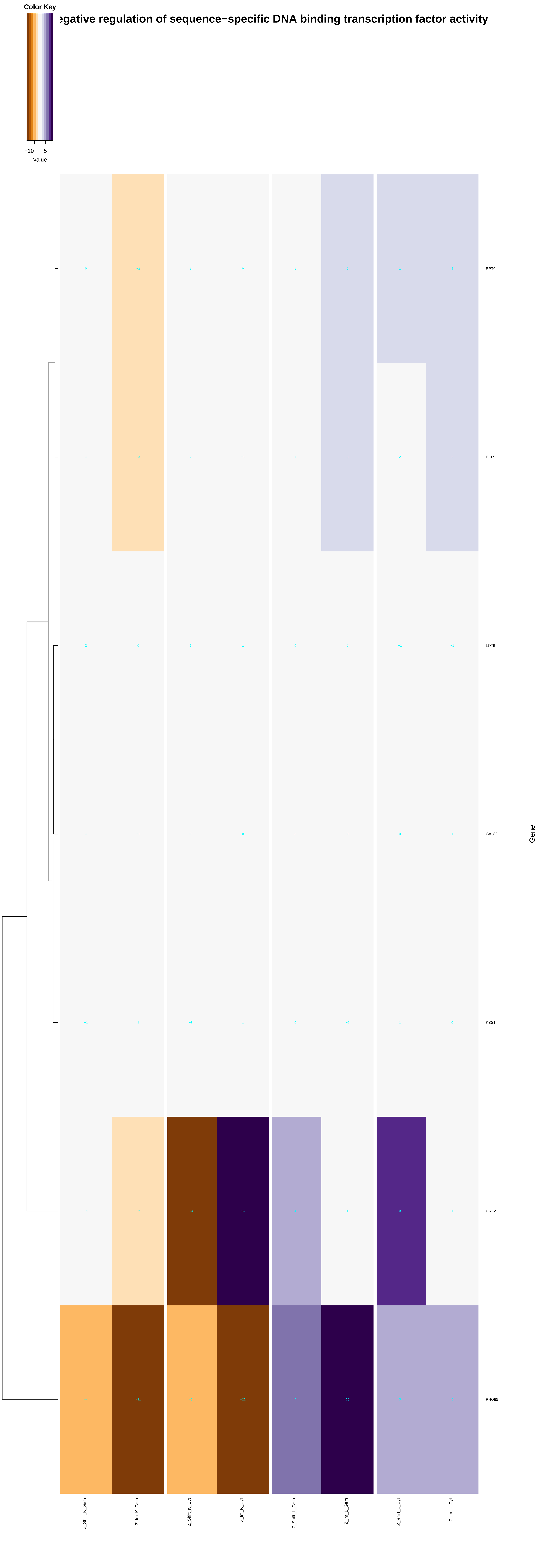
Color Key
−10
5
Value
egative regulation of sequence−specific DNA binding transcription factor activity
RPT6
PCL5
LOT6
GAL80
KSS1
URE2
PHO85
Gene
Z_Shift_K_Gem
Z_Im_K_Gem
Z_Shift_K_Cyt
Z_Im_K_Cyt
Z_Shift_L_Gem
Z_Im_L_Gem
Z_Shift_L_Cyt
Z_Im_L_Cyt

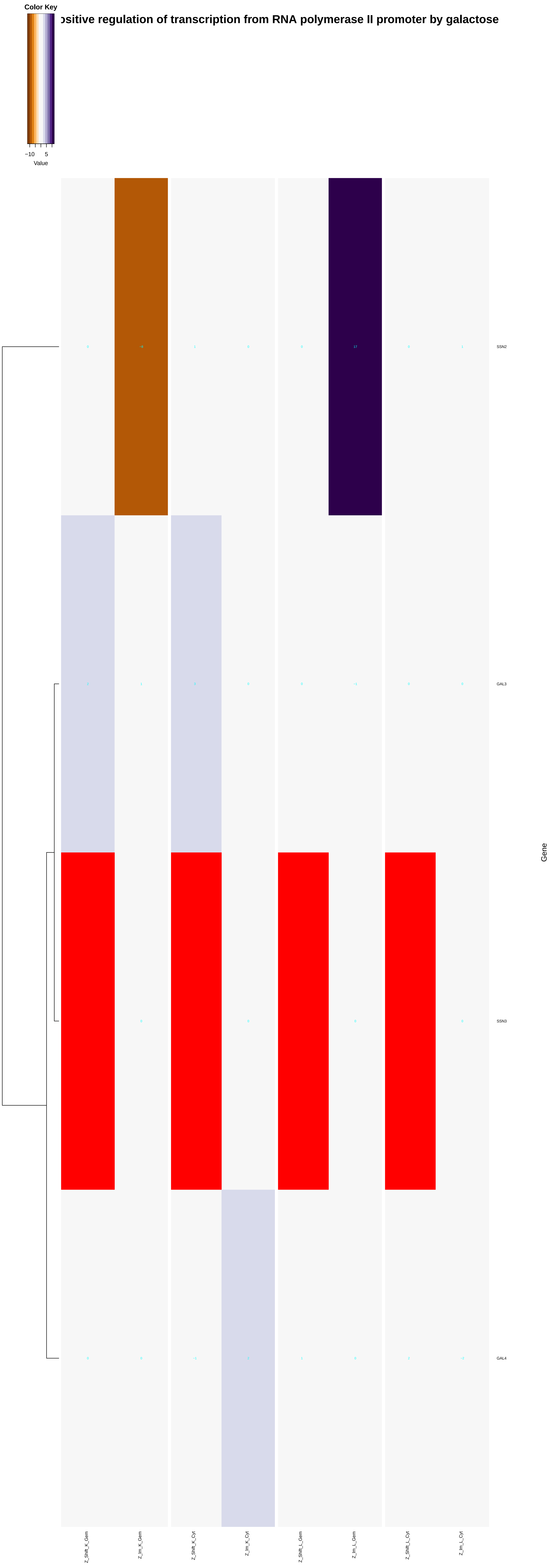

Color Key
−10
5
Value
ositive regulation of transcription from RNA polymerase II promoter by galactose
0
−8
1
0
0
17
0
1
SSN2
2
1
3
0
0
−1
0
0
GAL3
0
0
0
0
SSN3
0
0
−1
2
1
0
2
−2
GAL4
Gene
Z_Shift_K_Gem
Z_Im_K_Gem
Z_Shift_K_Cyt
Z_Im_K_Cyt
Z_Shift_L_Gem
Z_Im_L_Gem
Z_Shift_L_Cyt
Z_Im_L_Cyt

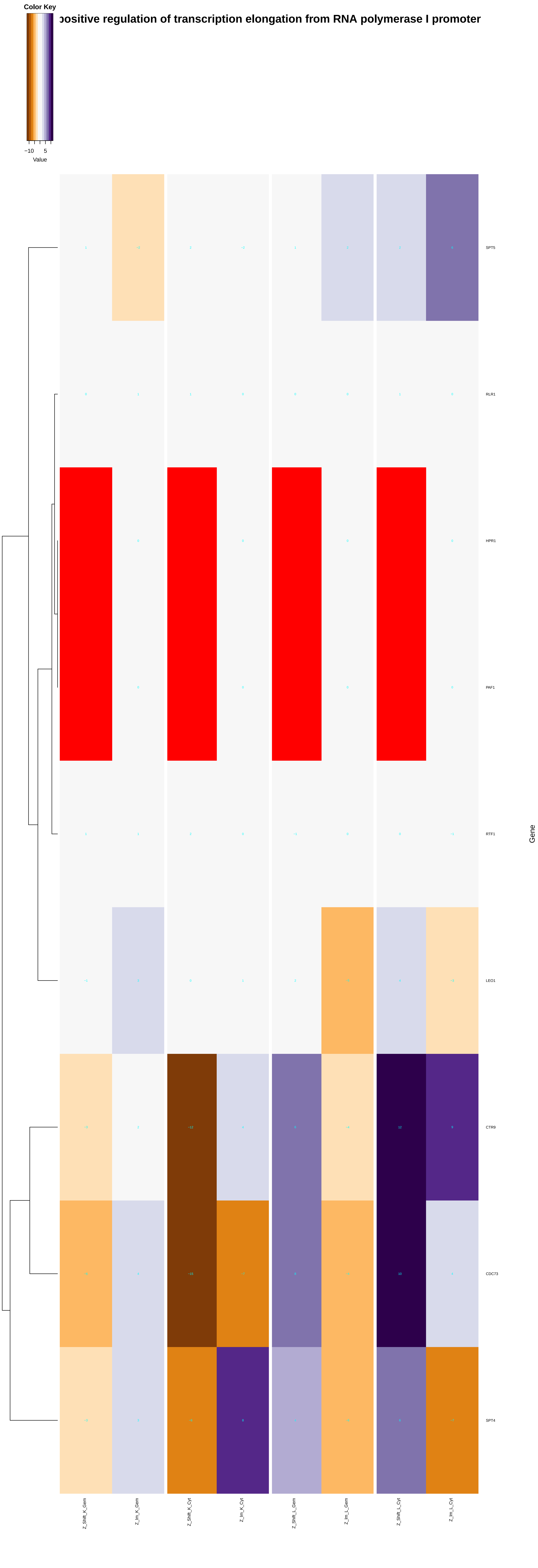
positive regulation of transcription elongation from RNA polymerase I promoter
Color Key
−10
5
Value
SPT5
RLR1
HPR1
PAF1
RTF1
LEO1
CTR9
CDC73
SPT4
Gene
Z_Shift_K_Gem
Z_Im_K_Gem
Z_Shift_K_Cyt
Z_Im_K_Cyt
Z_Shift_L_Gem
Z_Im_L_Gem
Z_Shift_L_Cyt
Z_Im_L_Cyt

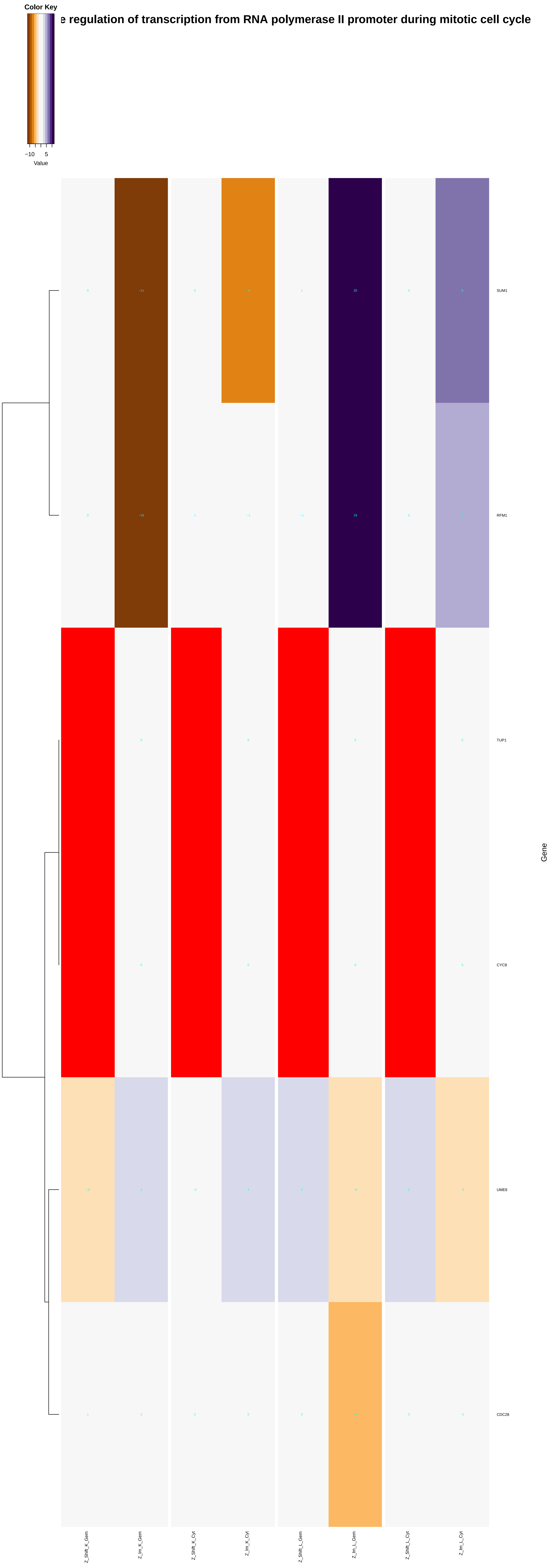
e regulation of transcription from RNA polymerase II promoter during mitotic cell cycle
Color Key
−10 5
Value
SUM1
RFM1
TUP1
CYC8
UME6
CDC28
Gene
Z_Shift_K_Gem
Z_Im_K_Gem
Z_Shift_K_Cyt
Z_Im_K_Cyt
Z_Shift_L_Gem
Z_Im_L_Gem
Z_Shift_L_Cyt
Z_Im_L_Cyt

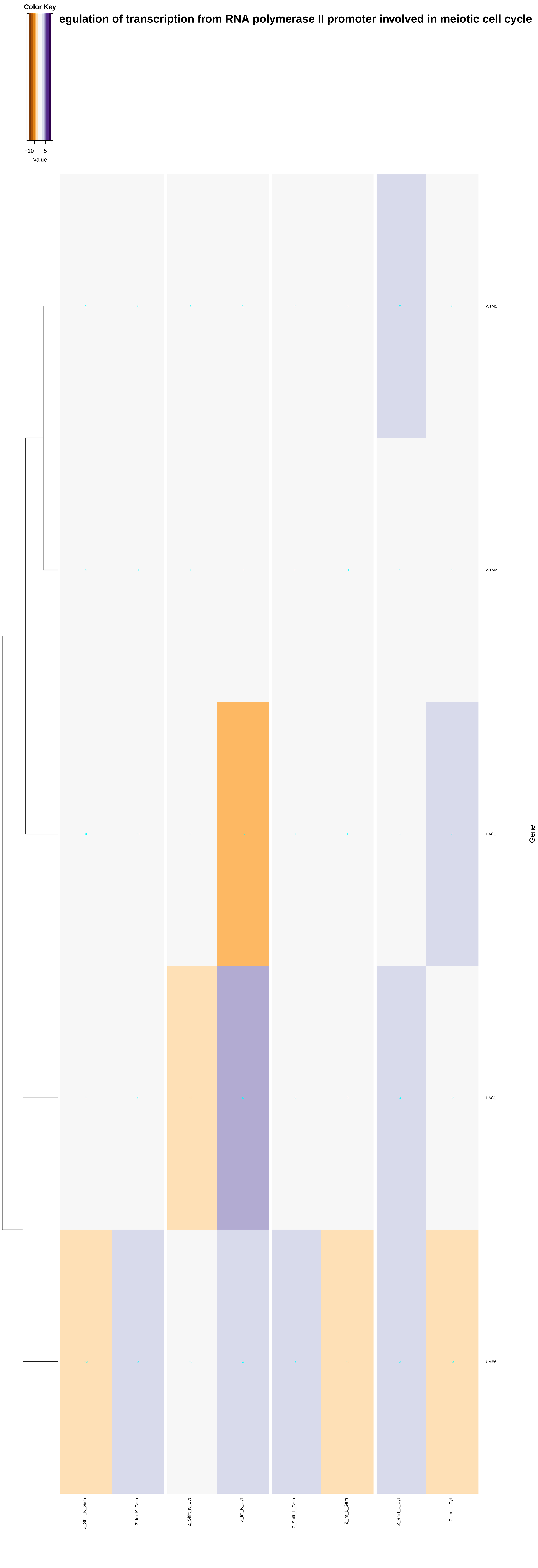

Color Key
−10
5
Value
egulation of transcription from RNA polymerase II promoter involved in meiotic cell cycle
WTM1
WTM2
HAC1
HAC1
UME6
Gene
Z_Shift_K_Gem
Z_Im_K_Gem
Z_Shift_K_Cyt
Z_Im_K_Cyt
Z_Shift_L_Gem
Z_Im_L_Gem
Z_Shift_L_Cyt
Z_Im_L_Cyt

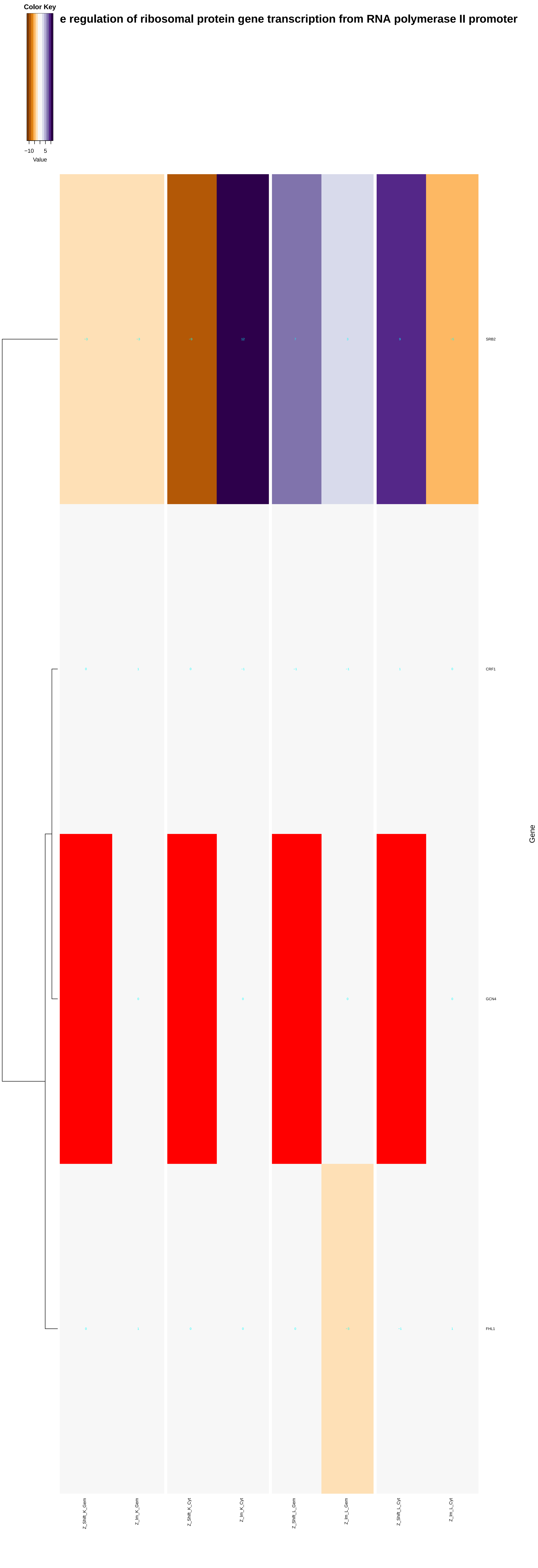
e regulation of ribosomal protein gene transcription from RNA polymerase II promoter
Color Key
−10
5
Value
SRB2
CRF1
GCN4
FHL1
Gene
Z_Shift_K_Gem
Z_Im_K_Gem
Z_Shift_K_Cyt
Z_Im_K_Cyt
Z_Shift_L_Gem
Z_Im_L_Gem
Z_Shift_L_Cyt
Z_Im_L_Cyt

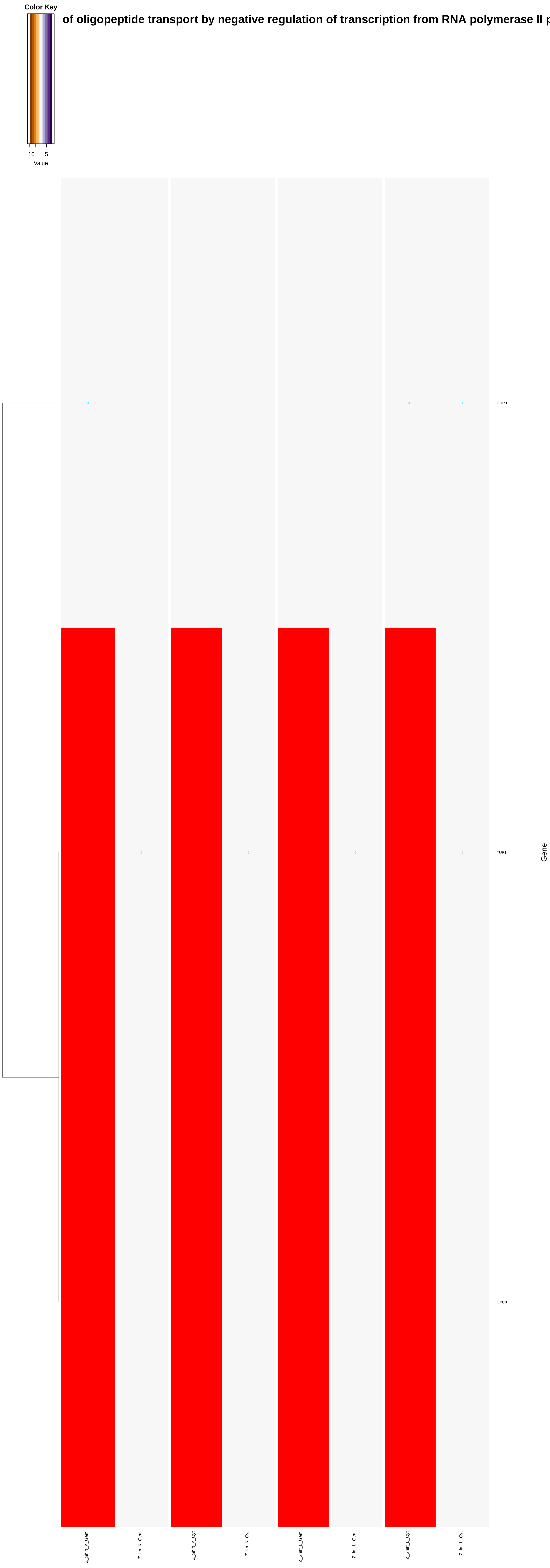

Color Key
−10
5
Value
of oligopeptide transport by negative regulation of transcription from RNA polymerase II p
CUP9
TUP1
CYC8
Gene
Z_Shift_K_Gem
Z_Im_K_Gem
Z_Shift_K_Cyt
Z_Im_K_Cyt
Z_Shift_L_Gem
Z_Im_L_Gem
Z_Shift_L_Cyt
Z_Im_L_Cyt

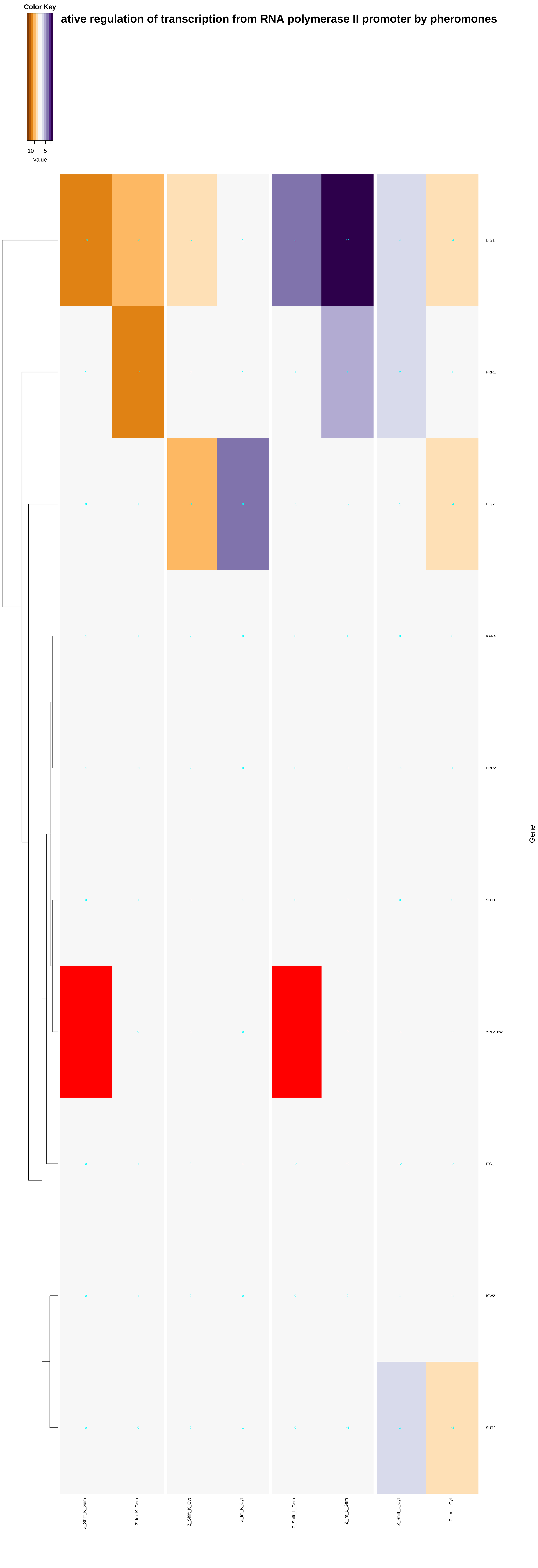
ative regulation of transcription from RNA polymerase II promoter by pheromones
Color Key
−10
5
Value
DIG1
PRR1
DIG2
KAR4
PRR2
SUT1
YPL216W
ITC1
ISW2
SUT2
Gene
Z_Shift_K_Gem
Z_Im_K_Gem
Z_Shift_K_Cyt
Z_Im_K_Cyt
Z_Shift_L_Gem
Z_Im_L_Gem
Z_Shift_L_Cyt
Z_Im_L_Cyt

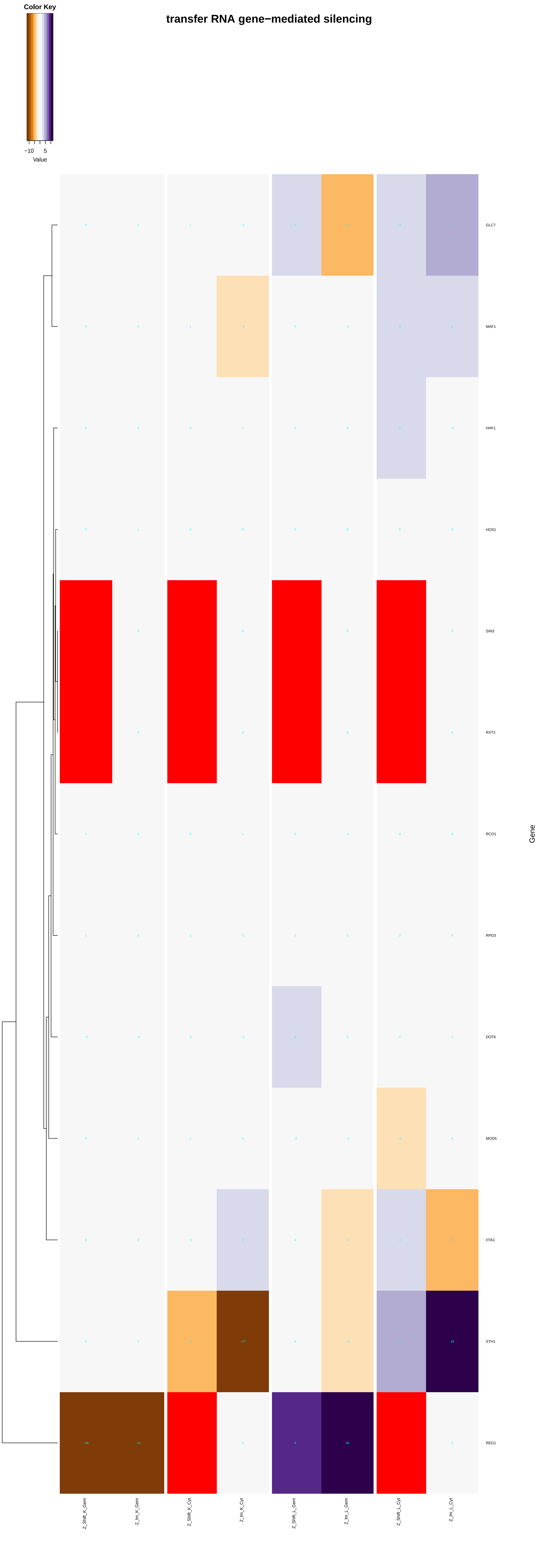
transfer RNA gene−mediated silencing
Color Key
−10
5
Value
GLC7
MAF1
HHF1
HOS1
SIN3
RXT2
RCO1
RPD3
DOT6
MOD5
HTA1
STH1
REG1
Gene
Z_Shift_K_Gem
Z_Im_K_Gem
Z_Shift_K_Cyt
Z_Im_K_Cyt
Z_Shift_L_Gem
Z_Im_L_Gem
Z_Shift_L_Cyt
Z_Im_L_Cyt

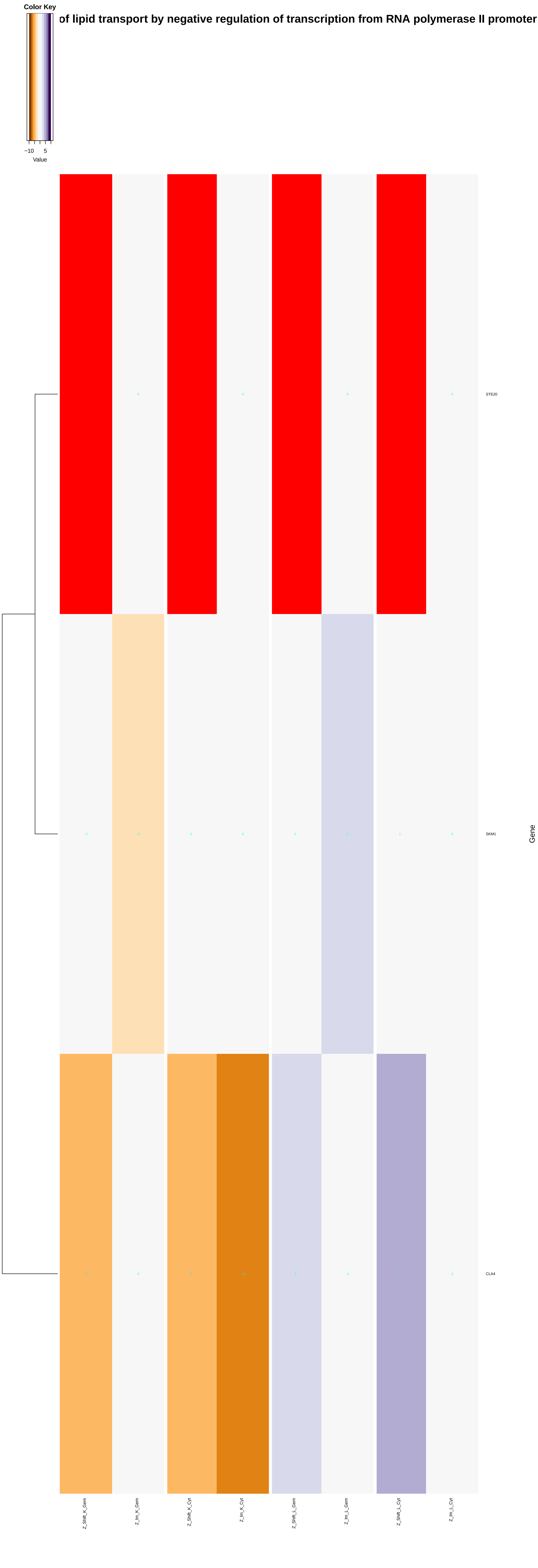
of lipid transport by negative regulation of transcription from RNA polymerase II promoter
Color Key
−10
5
Value
STE20
SKM1
CLA4
Gene
Z_Shift_K_Gem
Z_Im_K_Gem
Z_Shift_K_Cyt
Z_Im_K_Cyt
Z_Shift_L_Gem
Z_Im_L_Gem
Z_Shift_L_Cyt
Z_Im_L_Cyt

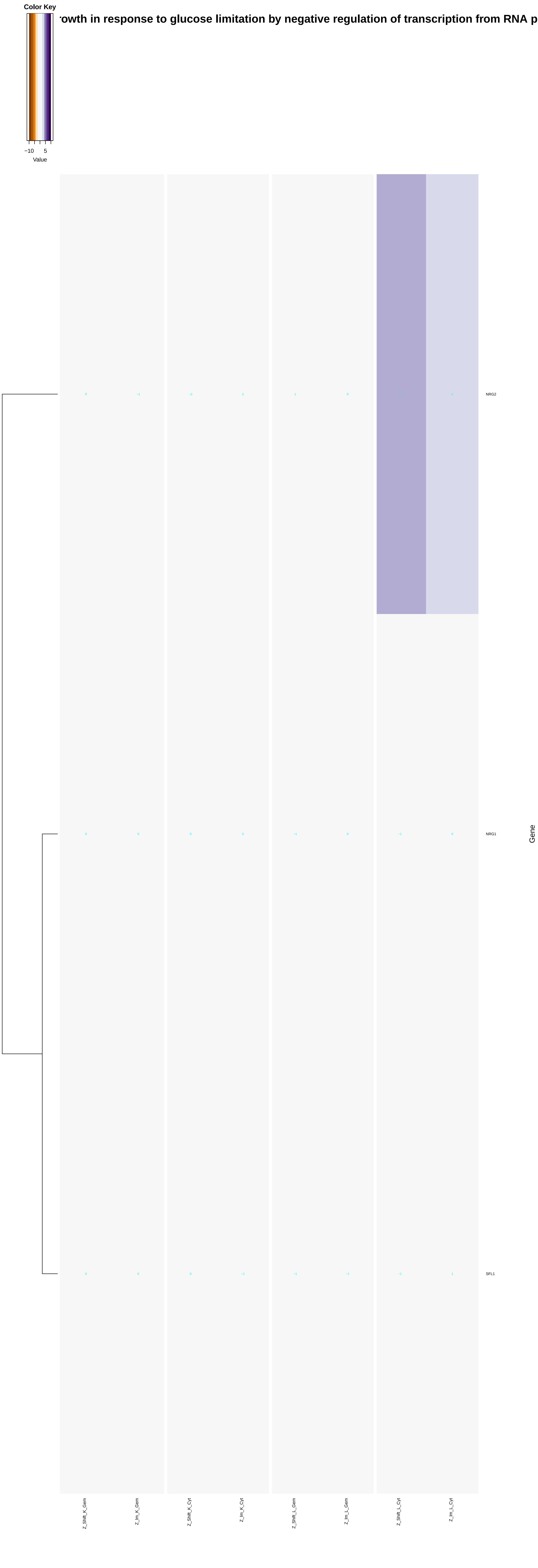

Color Key
−10
5
Value
rowth in response to glucose limitation by negative regulation of transcription from RNA p
NRG2
NRG1
SFL1
Gene
Z_Shift_K_Gem
Z_Im_K_Gem
Z_Shift_K_Cyt
Z_Im_K_Cyt
Z_Shift_L_Gem
Z_Im_L_Gem
Z_Shift_L_Cyt
Z_Im_L_Cyt

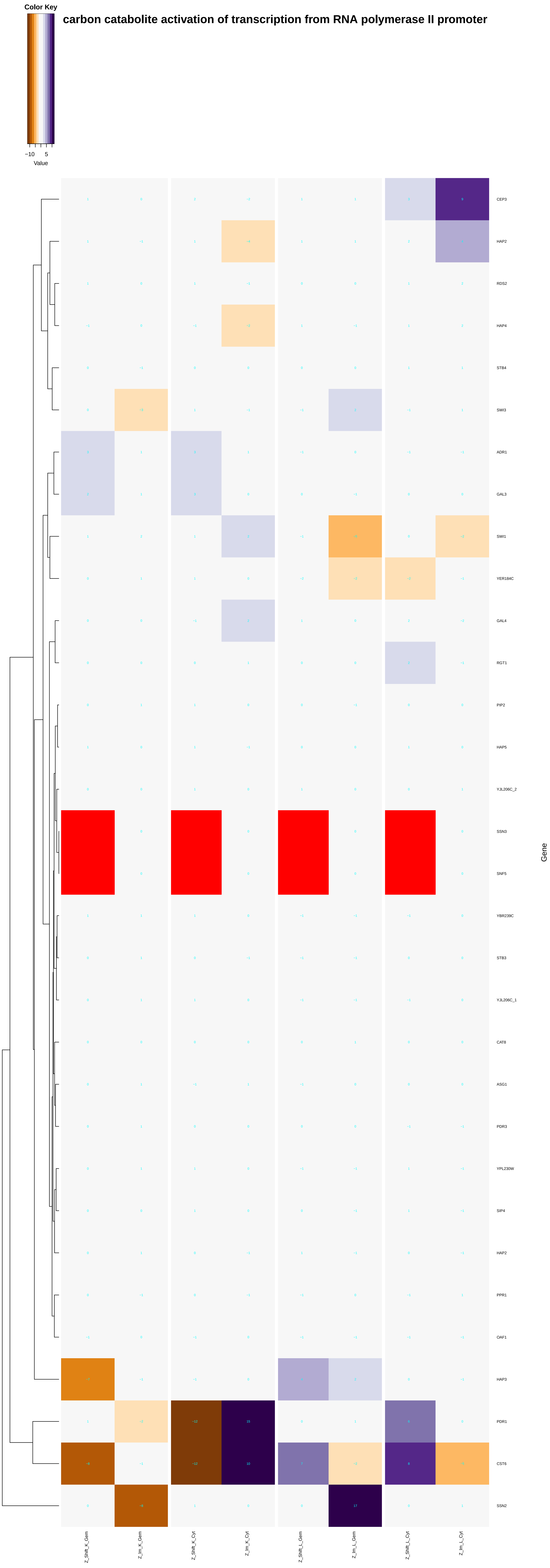
Color Key
−10 5
Value
carbon catabolite activation of transcription from RNA polymerase II promoter
CEP3
HAP2
RDS2
HAP4
STB4
SWI3
ADR1
GAL3
SWI1
YER184C
GAL4
RGT1
PIP2
HAP5
YJL206C_2
SSN3
SNF5
YBR239C
STB3
YJL206C_1
CAT8
ASG1
PDR3
YPL230W
SIP4
HAP2
PPR1
OAF1
HAP3
PDR1
CST6
SSN2
Gene
Z_Shift_K_Gem
Z_Im_K_Gem
Z_Shift_K_Cyt
Z_Im_K_Cyt
Z_Shift_L_Gem
Z_Im_L_Gem
Z_Shift_L_Cyt
Z_Im_L_Cyt

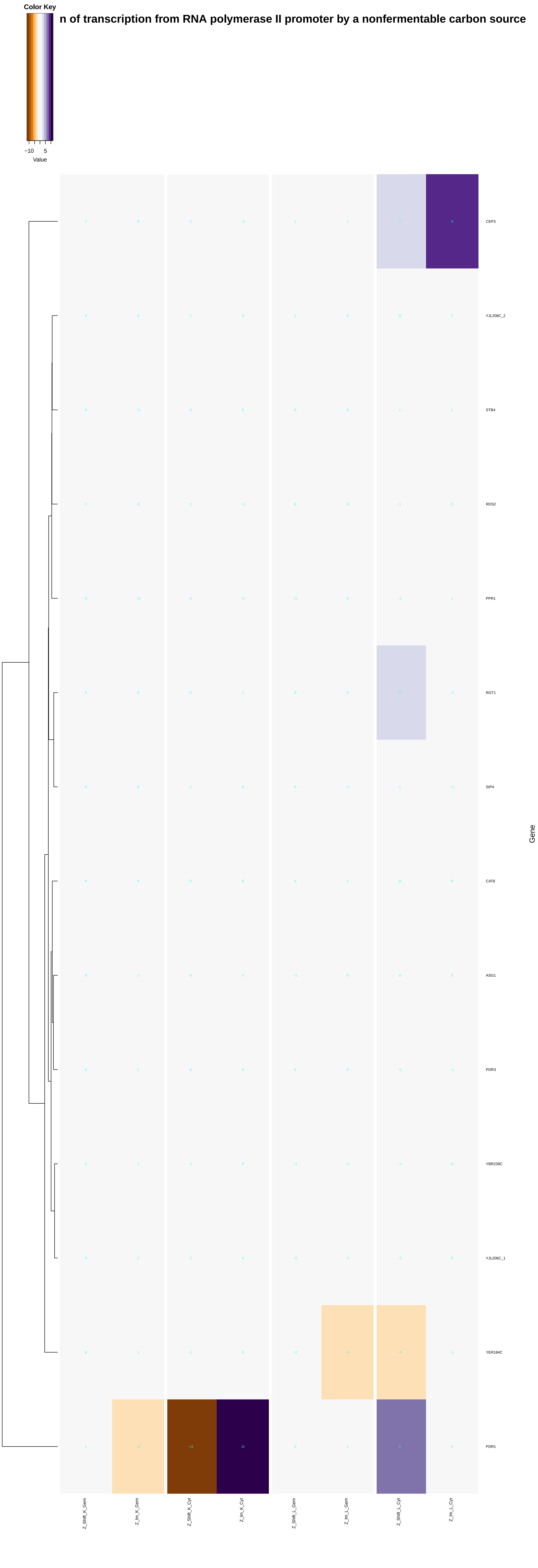
Color Key
−10
5
Value
n of transcription from RNA polymerase II promoter by a nonfermentable carbon source
CEP3
YJL206C_2
STB4
RDS2
PPR1
RGT1
SIP4
CAT8
ASG1
PDR3
YBR239C
YJL206C_1
YER184C
PDR1
Gene
Z_Shift_K_Gem
Z_lm_K_Gem
Z_Shift_K_Cyt
Z_lm_K_Cyt
Z_Shift_L_Gem
Z_lm_L_Gem
Z_Shift_L_Cyt
Z_lm_L_Cyt

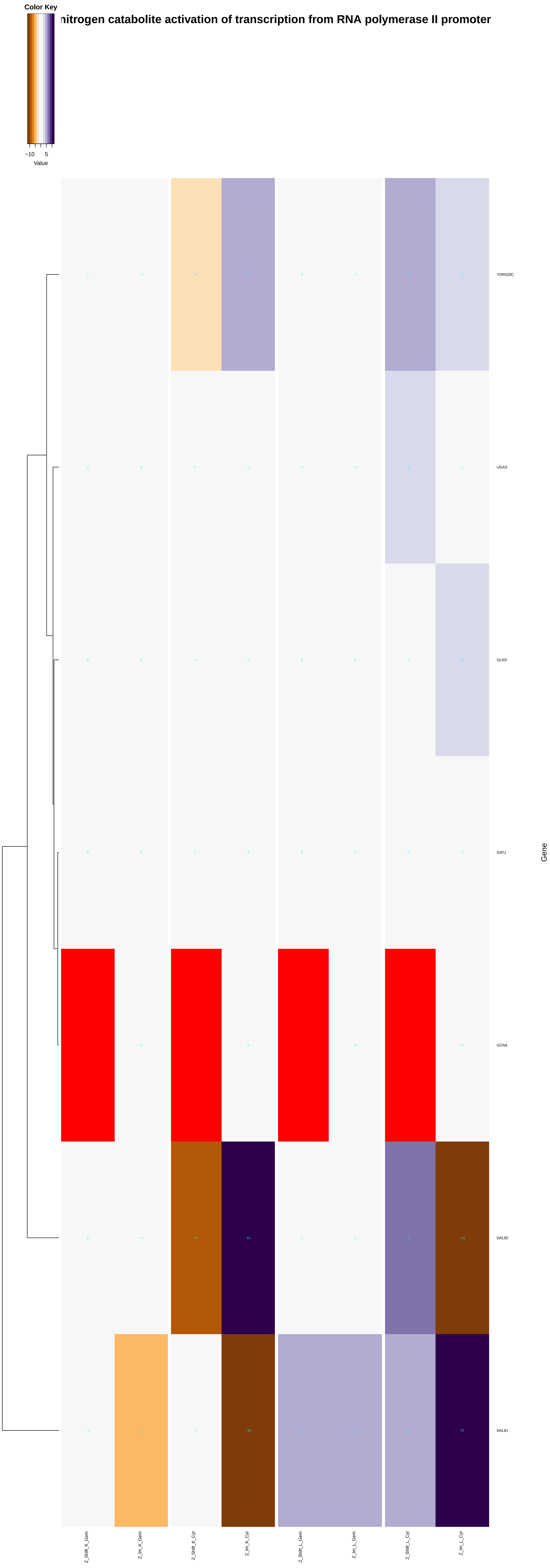
nitrogen catabolite activation of transcription from RNA polymerase II promoter
Color Key
−10
5
Value
YDR520C
UGA3
GLN3
GAT1
GCN4
DAL82
DAL81
Gene
Z_Shift_K_Gem
Z_Im_K_Gem
Z_Shift_K_Cyt
Z_Im_K_Cyt
Z_Shift_L_Gem
Z_Im_L_Gem
Z_Shift_L_Cyt
Z_Im_L_Cyt

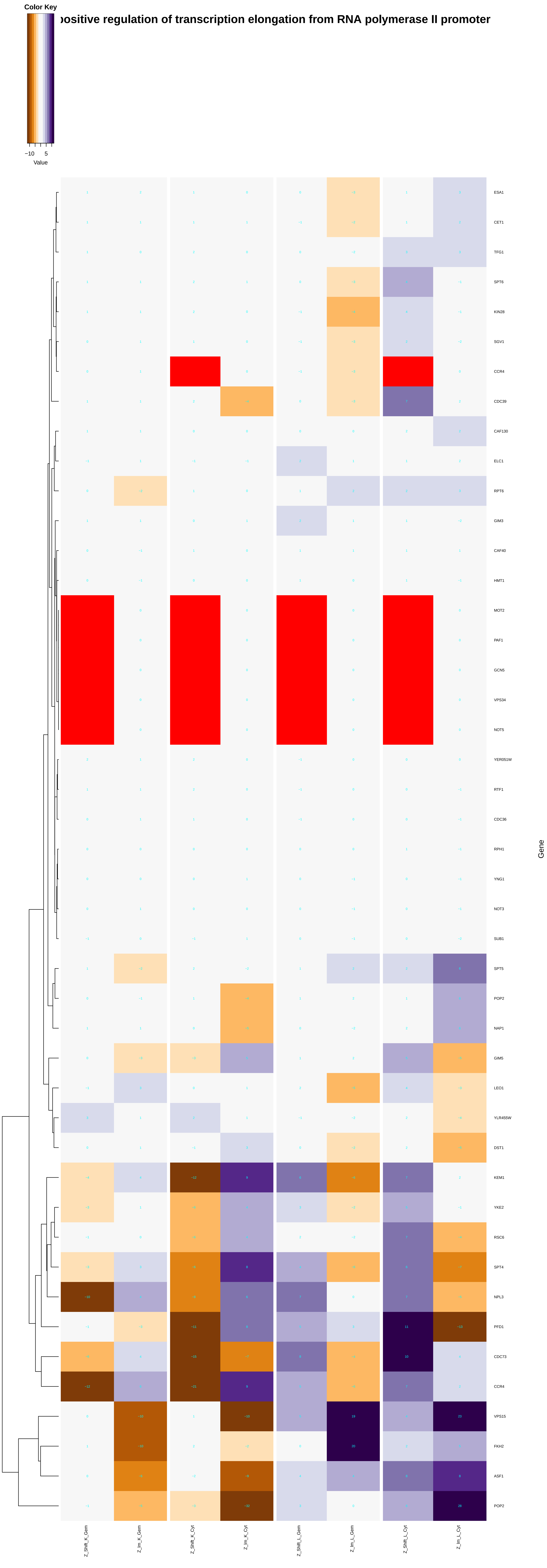
Color Key
−10
5
Value
positive regulation of transcription elongation from RNA polymerase II promoter
ESA1
CET1
TFG1
SPT6
KIN28
SGV1
CCR4
CDC39
CAF130
ELC1
RPT6
GIM3
CAF40
HMT1
MOT2
PAF1
GCN5
VPS34
NOT5
YER051W
RTF1
CDC36
RPH1
YNG1
NOT3
SUB1
SPT5
POP2
NAP1
GIM5
LEO1
YLR455W
DST1
KEM1
YKE2
RSC6
SPT4
NPL3
PFD1
CDC73
CCR4
VPS15
FKH2
ASF1
POP2
Gene
Z_Shift_K_Gem
Z_Im_K_Gem
Z_Shift_K_Cyt
Z_Im_K_Cyt
Z_Shift_L_Gem
Z_Im_L_Gem
Z_Shift_L_Cyt
Z_Im_L_Cyt

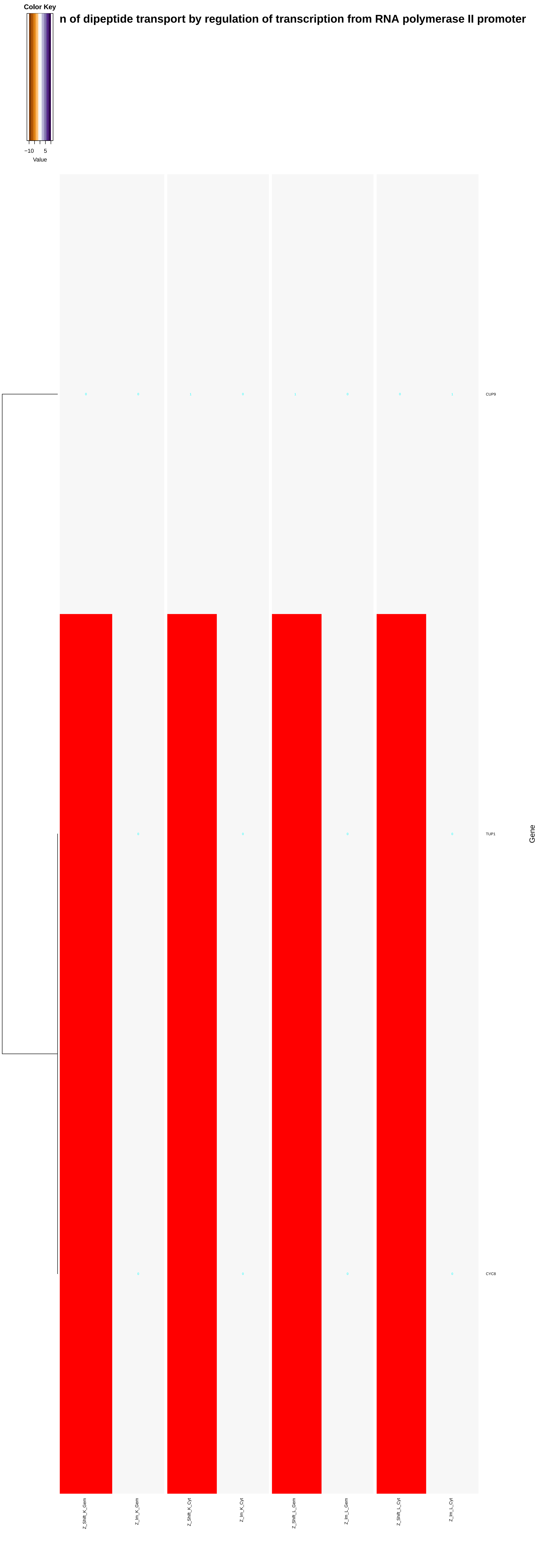
n of dipeptide transport by regulation of transcription from RNA polymerase II promoter
Color Key
−10
5
Value
CUP9
TUP1
CYC8
Gene
Z_Shift_K_Gem
Z_Im_K_Gem
Z_Shift_K_Cyt
Z_Im_K_Cyt
Z_Shift_L_Gem
Z_Im_L_Gem
Z_Shift_L_Cyt
Z_Im_L_Cyt

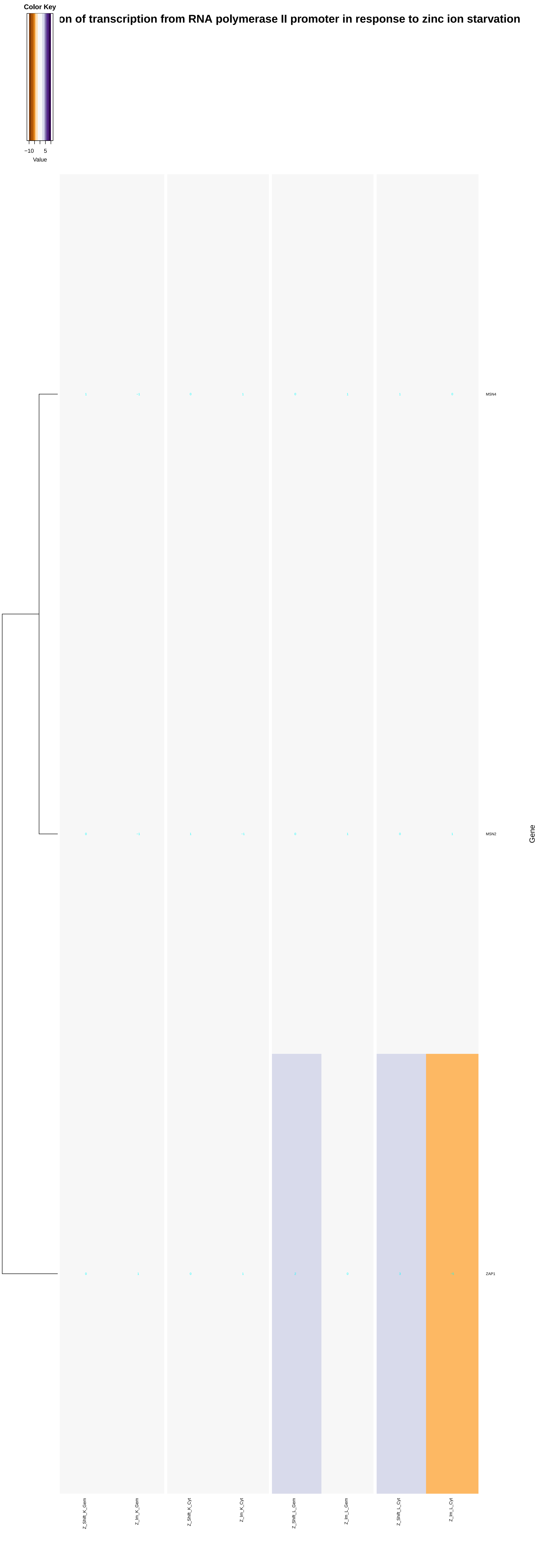
on of transcription from RNA polymerase II promoter in response to zinc ion starvation
Color Key
−10
5
Value
MSN4
MSN2
ZAP1
Gene
Z_Shift_K_Gem
Z_Im_K_Gem
Z_Shift_K_Cyt
Z_Im_K_Cyt
Z_Shift_L_Gem
Z_Im_L_Gem
Z_Shift_L_Cyt
Z_Im_L_Cyt

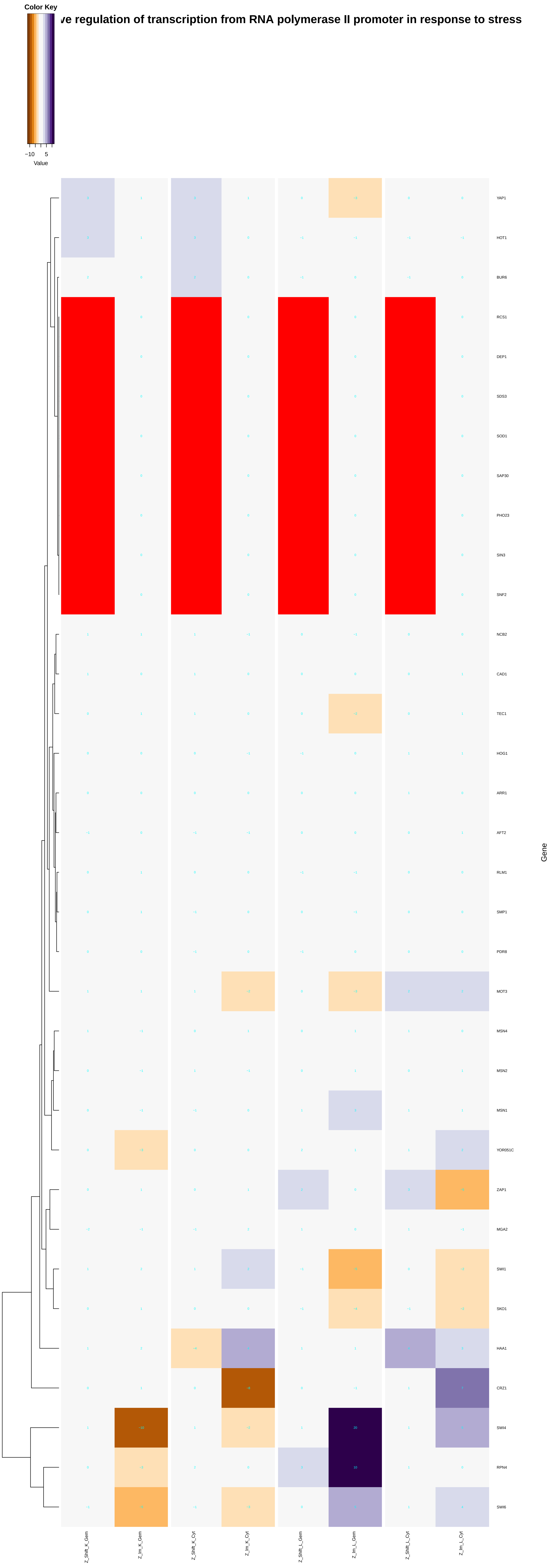
ve regulation of transcription from RNA polymerase II promoter in response to stress
Color Key
−10
5
Value
YAP1
HOT1
BUR6
RCS1
DEP1
SDS3
SOD1
SAP30
PHO23
SIN3
SNF2
NCB2
CAD1
TEC1
HOG1
ARR1
AFT2
RLM1
SMP1
PDR8
MOT3
MSN4
MSN2
MSN1
YOR051C
ZAP1
MGA2
SWI1
SKO1
HAA1
CRZ1
SWI4
RPN4
SWI6
Gene
Z_Shift_K_Gem
Z_Im_K_Gem
Z_Shift_K_Cyt
Z_Im_K_Cyt
Z_Shift_L_Gem
Z_Im_L_Gem
Z_Shift_L_Cyt
Z_Im_L_Cyt

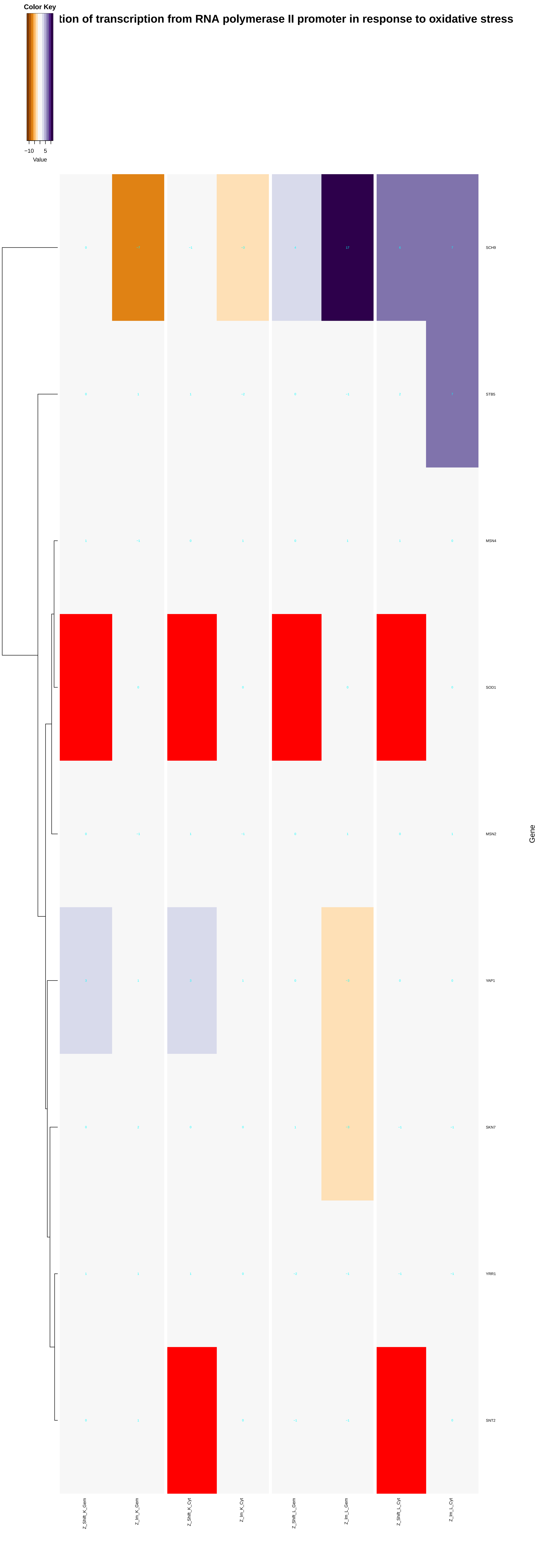
tion of transcription from RNA polymerase II promoter in response to oxidative stress
Color Key
−10
5
Value
SCH9
STB5
MSN4
SOD1
MSN2
YAP1
SKN7
YRR1
SNT2
Gene
Z_Shift_K_Gem
Z_Im_K_Gem
Z_Shift_K_Cyt
Z_Im_K_Cyt
Z_Shift_L_Gem
Z_Im_L_Gem
Z_Shift_L_Cyt
Z_Im_L_Cyt

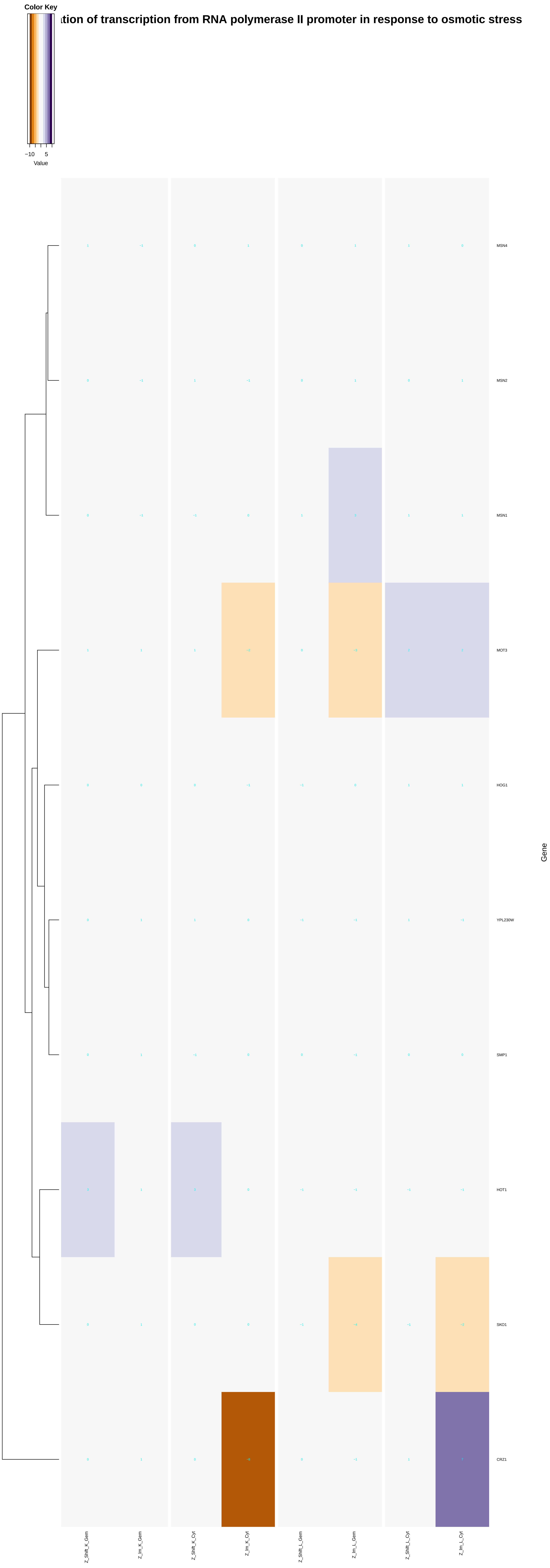
ation of transcription from RNA polymerase II promoter in response to osmotic stress
Color Key
−10
5
Value
MSN4
MSN2
MSN1
MOT3
HOG1
YPL230W
SMP1
HOT1
SKO1
CRZ1
Gene
Z_Shift_K_Gem
Z_Im_K_Gem
Z_Shift_K_Cyt
Z_Im_K_Cyt
Z_Shift_L_Gem
Z_Im_L_Gem
Z_Shift_L_Cyt
Z_Im_L_Cyt

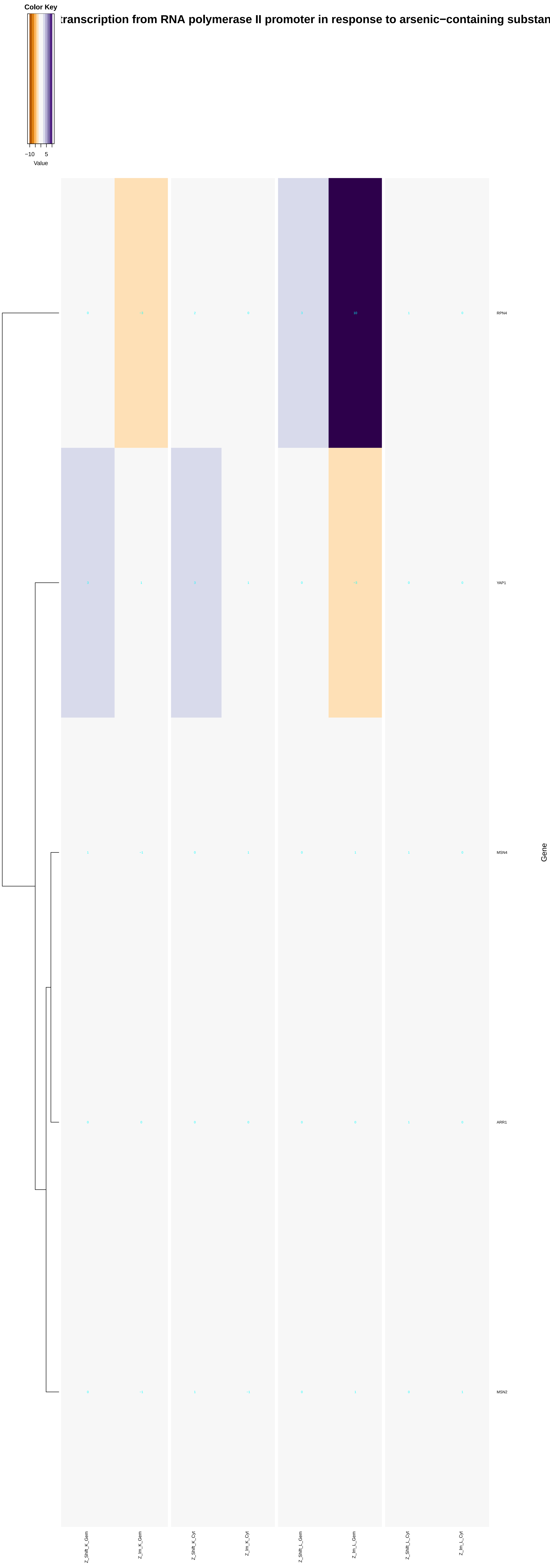
transcription from RNA polymerase II promoter in response to arsenic−containing substan
Color Key
−10
5
Value
RPN4
YAP1
MSN4
ARR1
MSN2
Gene
Z_Shift_K_Gem
Z_lm_K_Gem
Z_Shift_K_Cyt
Z_lm_K_Cyt
Z_Shift_L_Gem
Z_lm_L_Gem
Z_Shift_L_Cyt
Z_lm_L_Cyt

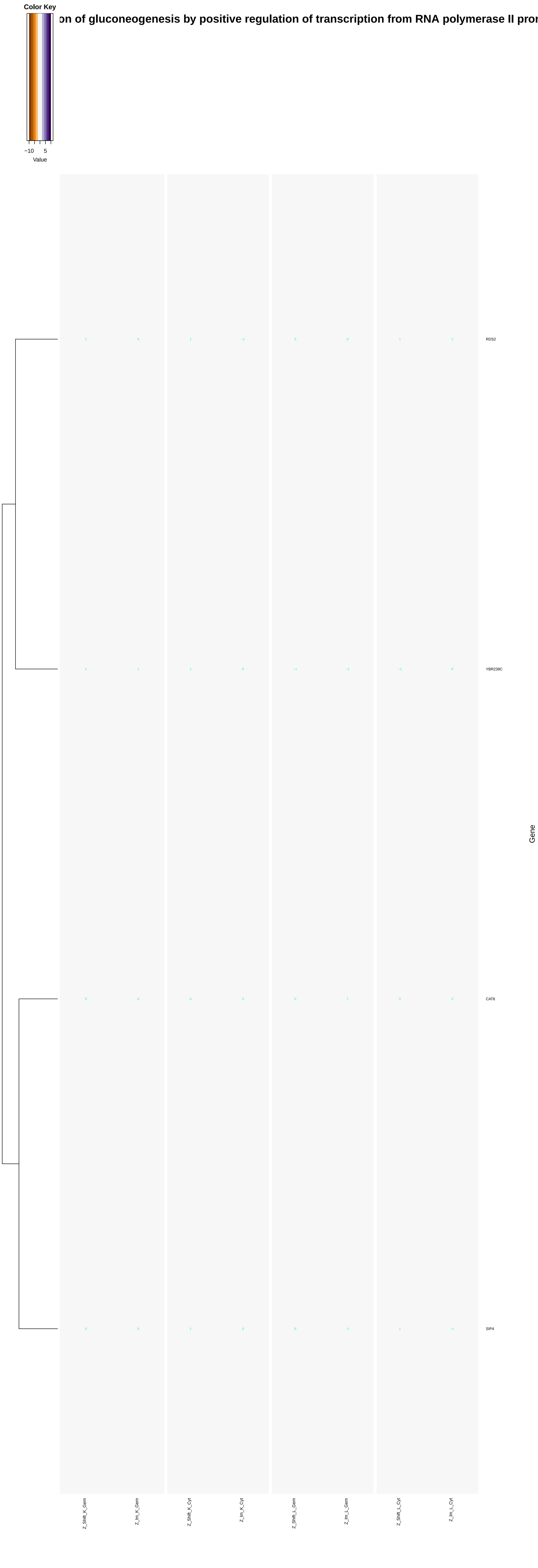
on of gluconeogenesis by positive regulation of transcription from RNA polymerase II pro
Color Key
−10
5
Value
ROS2
YBR239C
CAT8
SIP4
Gene
Z_Shift_K_Gem
Z_Im_K_Gem
Z_Shift_K_Cyt
Z_Im_K_Cyt
Z_Shift_L_Gem
Z_Im_L_Gem
Z_Shift_L_Cyt
Z_Im_L_Cyt

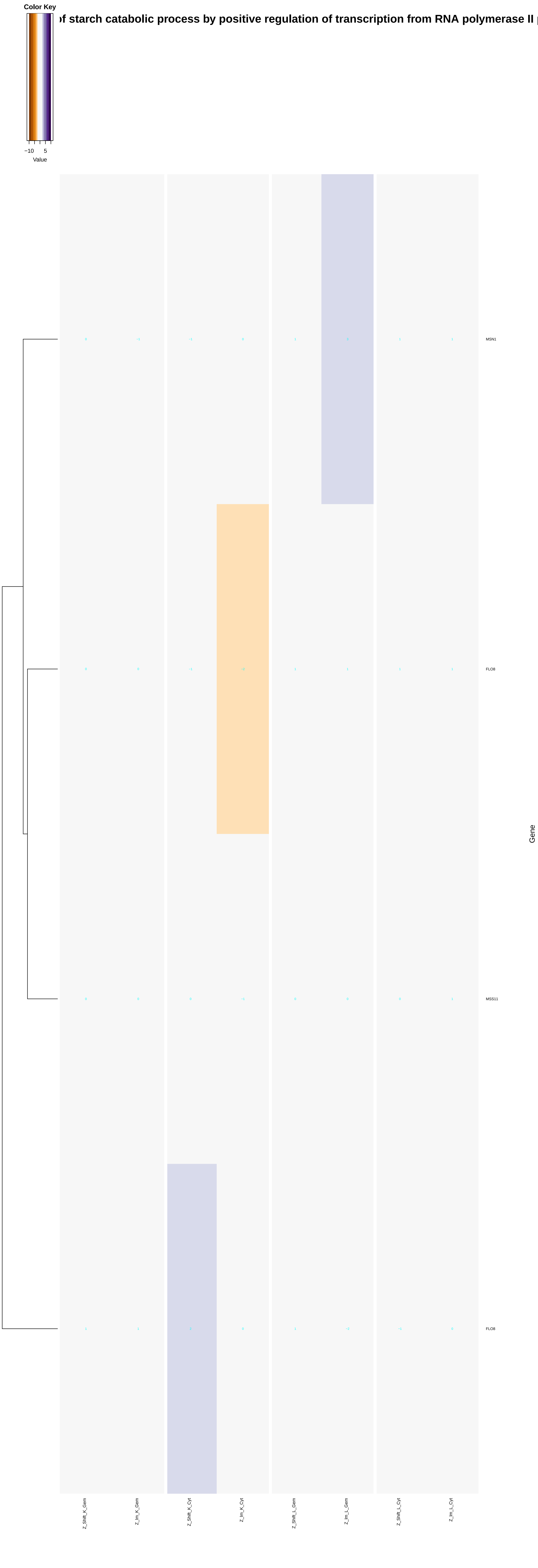
Color Key
−10
5
Value
of starch catabolic process by positive regulation of transcription from RNA polymerase II p
MSN1
FLO8
MSS11
FLO8
Gene
Z_Shift_K_Gem
Z_Im_K_Gem
Z_Shift_K_Cyt
Z_Im_K_Cyt
Z_Shift_L_Gem
Z_Im_L_Gem
Z_Shift_L_Cyt
Z_Im_L_Cyt

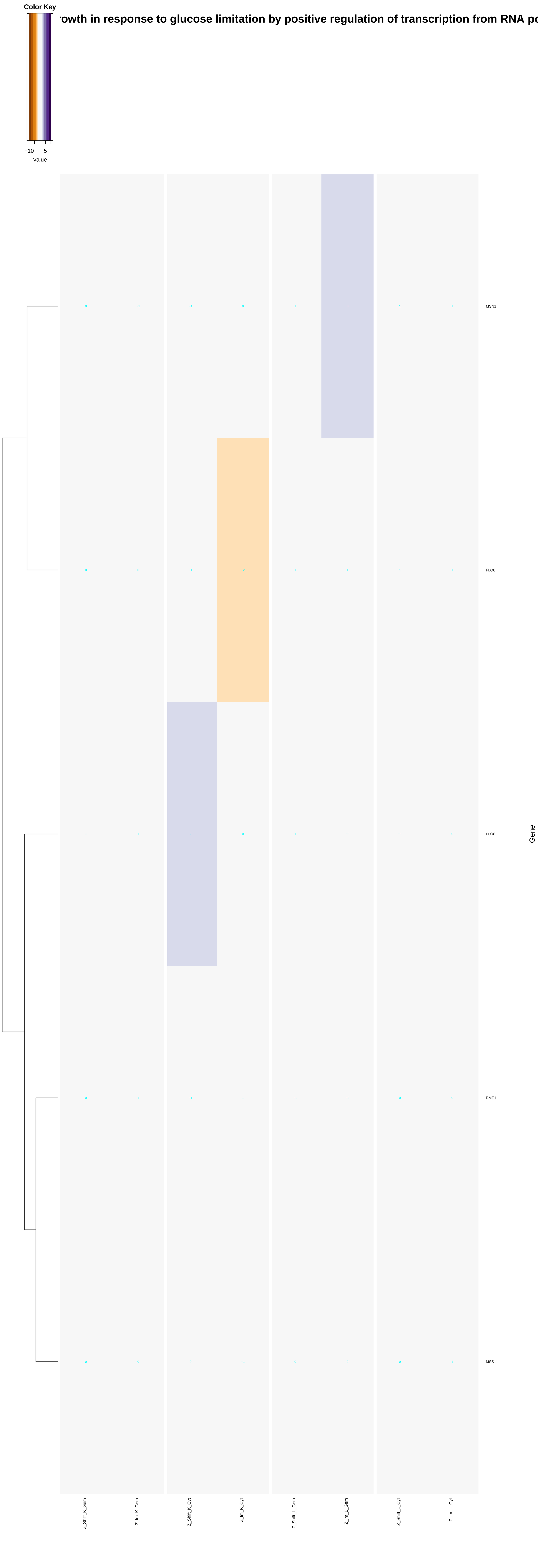

Color Key
−10
5
Value
rowth in response to glucose limitation by positive regulation of transcription from RNA po
MSN1
FLO8
FLO8
RME1
MSS11
Gene
Z_Shift_K_Gem
Z_Im_K_Gem
Z_Shift_K_Cyt
Z_Im_K_Cyt
Z_Shift_L_Gem
Z_Im_L_Gem
Z_Shift_L_Cyt
Z_Im_L_Cyt

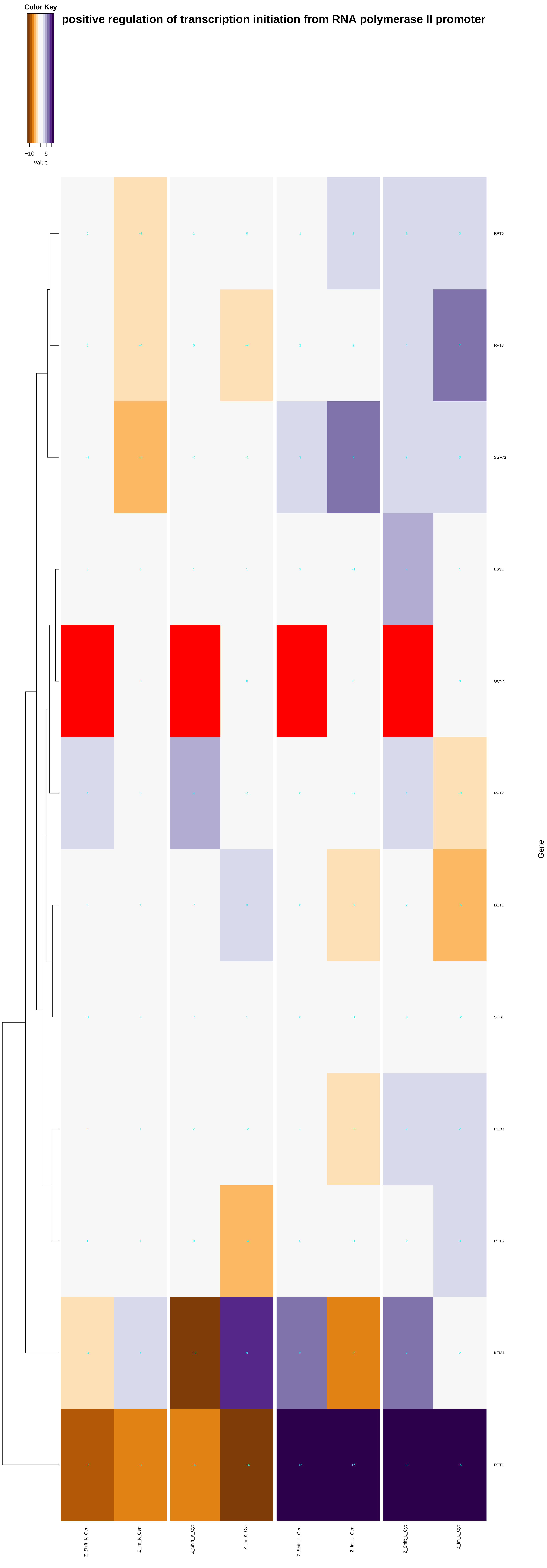

positive regulation of transcription initiation from RNA polymerase II promoter
Color Key
−10
5
Value
RPT6
RPT3
SGF73
ESS1
GCN4
RPT2
DST1
SUB1
POB3
RPT5
KEM1
RPT1
Gene
Z_Shift_K_Gem
Z_Im_K_Gem
Z_Shift_K_Cyt
Z_Im_K_Cyt
Z_Shift_L_Gem
Z_Im_L_Gem
Z_Shift_L_Cyt
Z_Im_L_Cyt

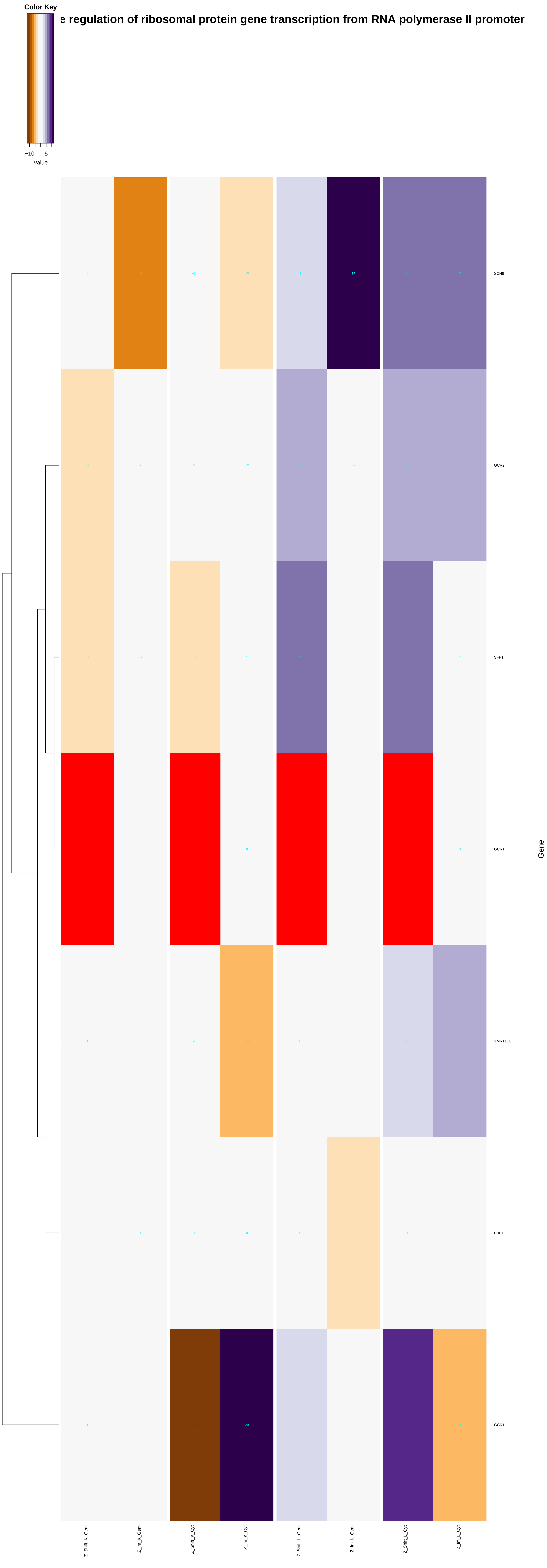
e regulation of ribosomal protein gene transcription from RNA polymerase II promoter
Color Key
−10
5
Value
SCH9
GCR2
SFP1
GCR1
YMR111C
FHL1
GCR1
Gene
Z_Shift_K_Gem
Z_Im_K_Gem
Z_Shift_K_Cyt
Z_Im_K_Cyt
Z_Shift_L_Gem
Z_Im_L_Gem
Z_Shift_L_Cyt
Z_Im_L_Cyt

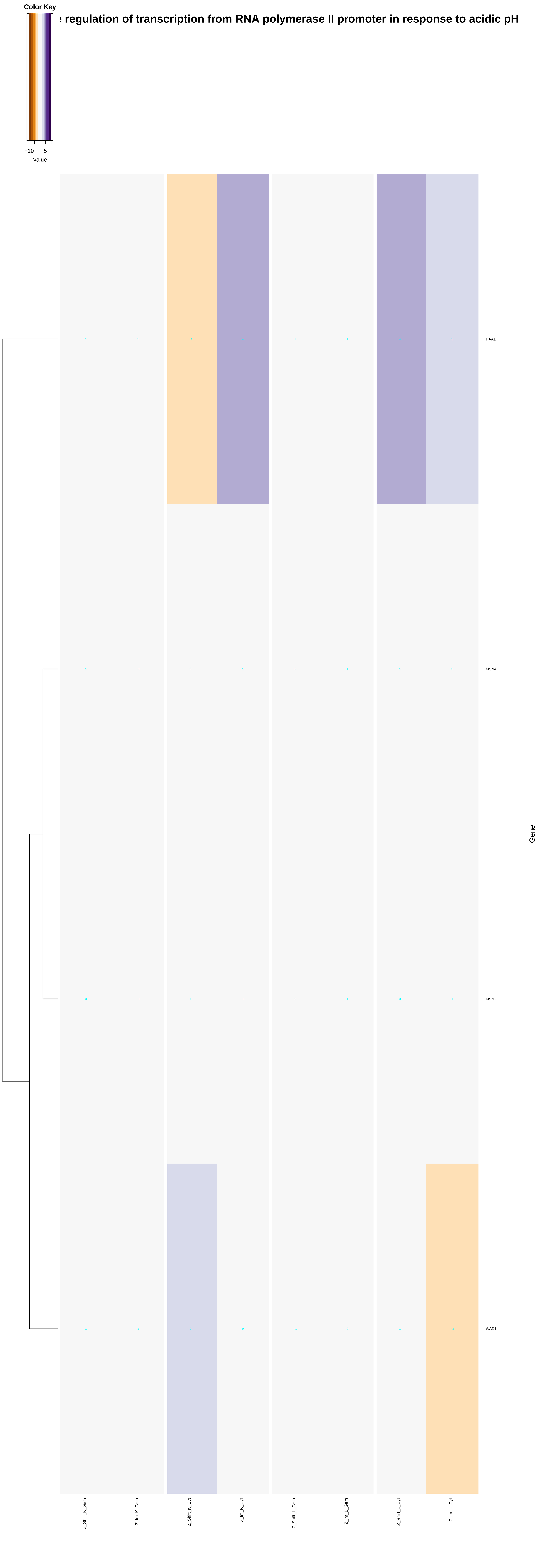

e regulation of transcription from RNA polymerase II promoter in response to acidic pH
Color Key
−10
5
Value
HAA1
MSN4
MSN2
WAR1
Gene
Z_Shift_K_Gem
Z_Im_K_Gem
Z_Shift_K_Cyt
Z_Im_K_Cyt
Z_Shift_L_Gem
Z_Im_L_Gem
Z_Shift_L_Cyt
Z_Im_L_Cyt

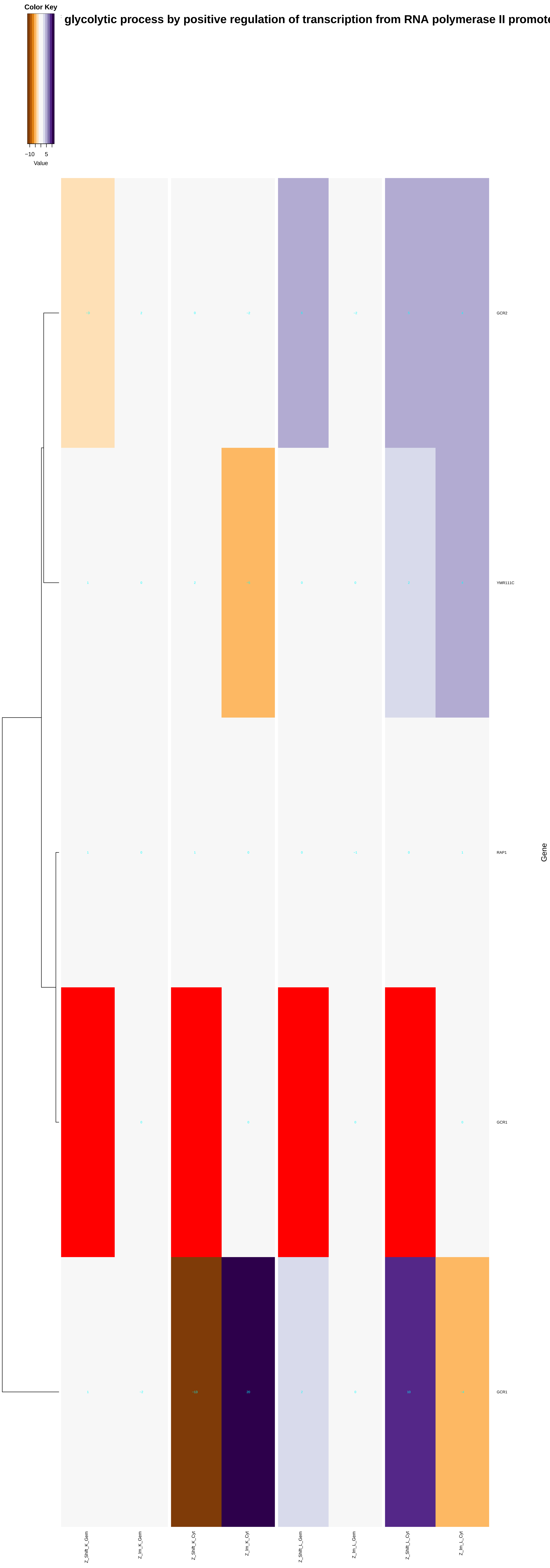

Color Key
−10
5
Value
glycolytic process by positive regulation of transcription from RNA polymerase II promote
GCR2
YMR111C
RAP1
GCR1
GCR1
Gene
Z_Shift_K_Gem
Z_Im_K_Gem
Z_Shift_K_Cyt
Z_Im_K_Cyt
Z_Shift_L_Gem
Z_Im_L_Gem
Z_Shift_L_Cyt
Z_Im_L_Cyt

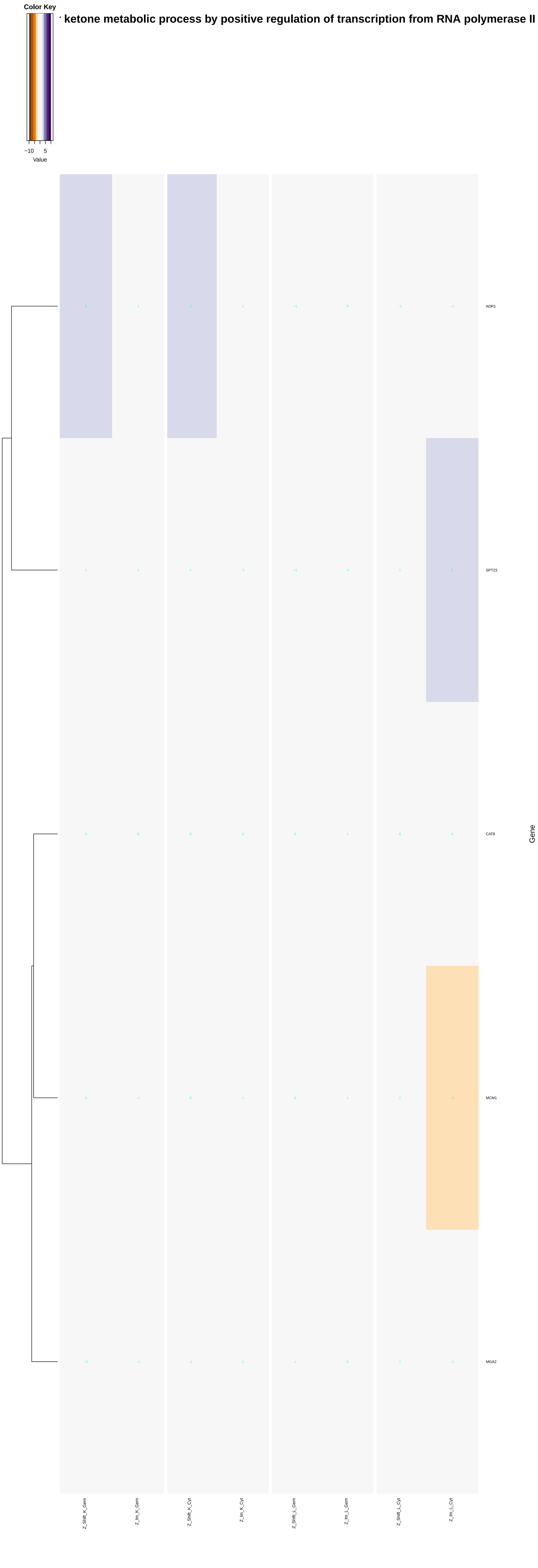
ketone metabolic process by positive regulation of transcription from RNA polymerase II
Color Key
−10
5
Value
ADR1
SPT23
CAT8
MCM1
MGA2
Gene
Z_Shift_K_Gem
Z_Im_K_Gem
Z_Shift_K_Cyt
Z_Im_K_Cyt
Z_Shift_L_Gem
Z_Im_L_Gem
Z_Shift_L_Cyt
Z_Im_L_Cyt

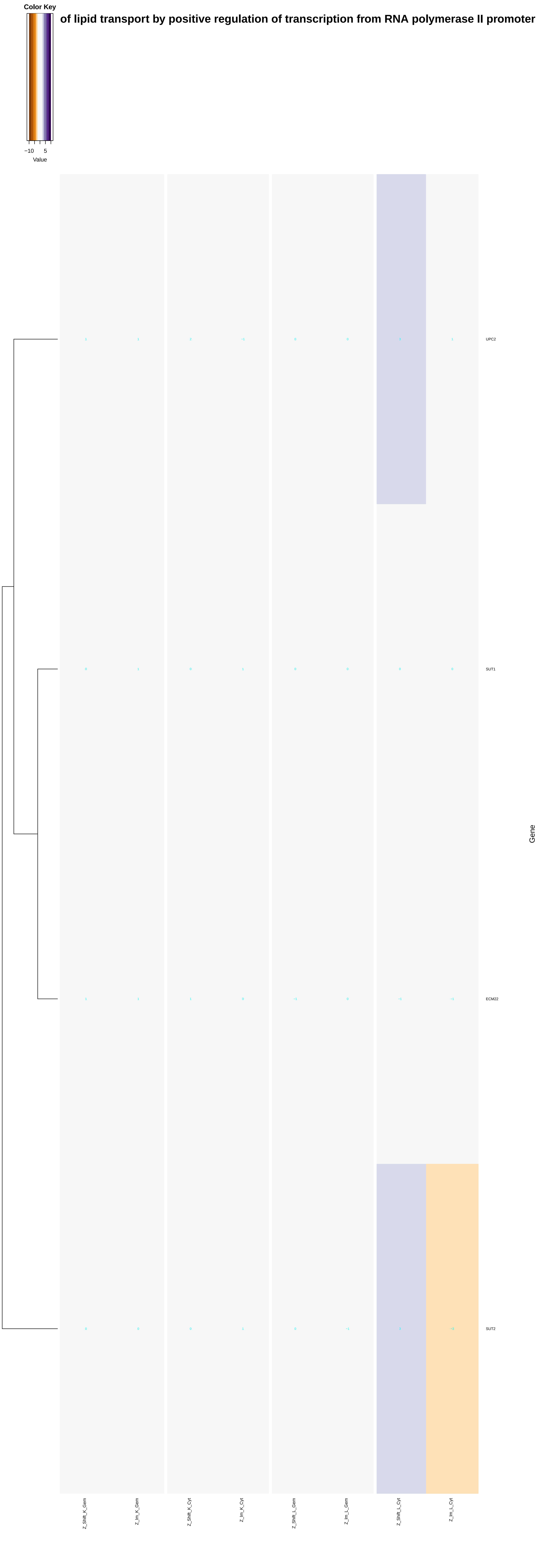
Color Key
−10
5
Value
of lipid transport by positive regulation of transcription from RNA polymerase II promoter
UPC2
SUT1
ECM22
SUT2
Gene
Z_Shift_K_Gem
Z_Im_K_Gem
Z_Shift_K_Cyt
Z_Im_K_Cyt
Z_Shift_L_Gem
Z_Im_L_Gem
Z_Shift_L_Cyt
Z_Im_L_Cyt

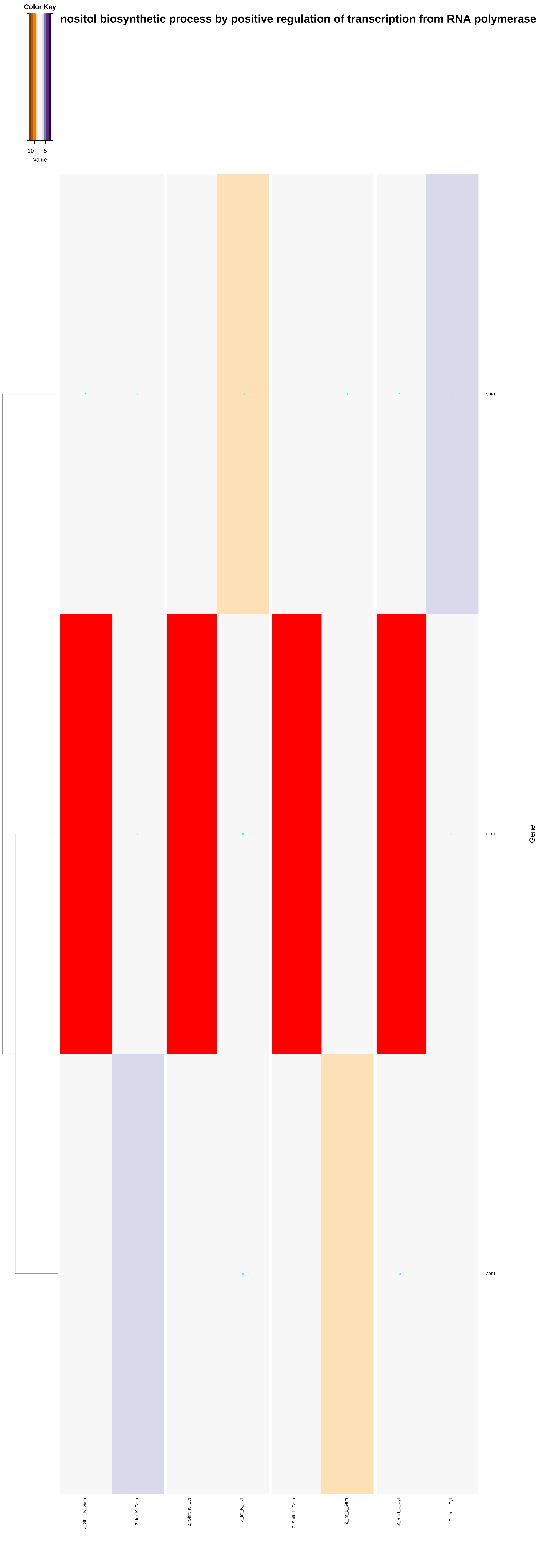
nositol biosynthetic process by positive regulation of transcription from RNA polymerase
Color Key
−10
5
Value
CBF1
DEP1
CBF1
Gene
Z_Shift_K_Gem
Z_Im_K_Gem
Z_Shift_K_Cyt
Z_Im_K_Cyt
Z_Shift_L_Gem
Z_Im_L_Gem
Z_Shift_L_Cyt
Z_Im_L_Cyt

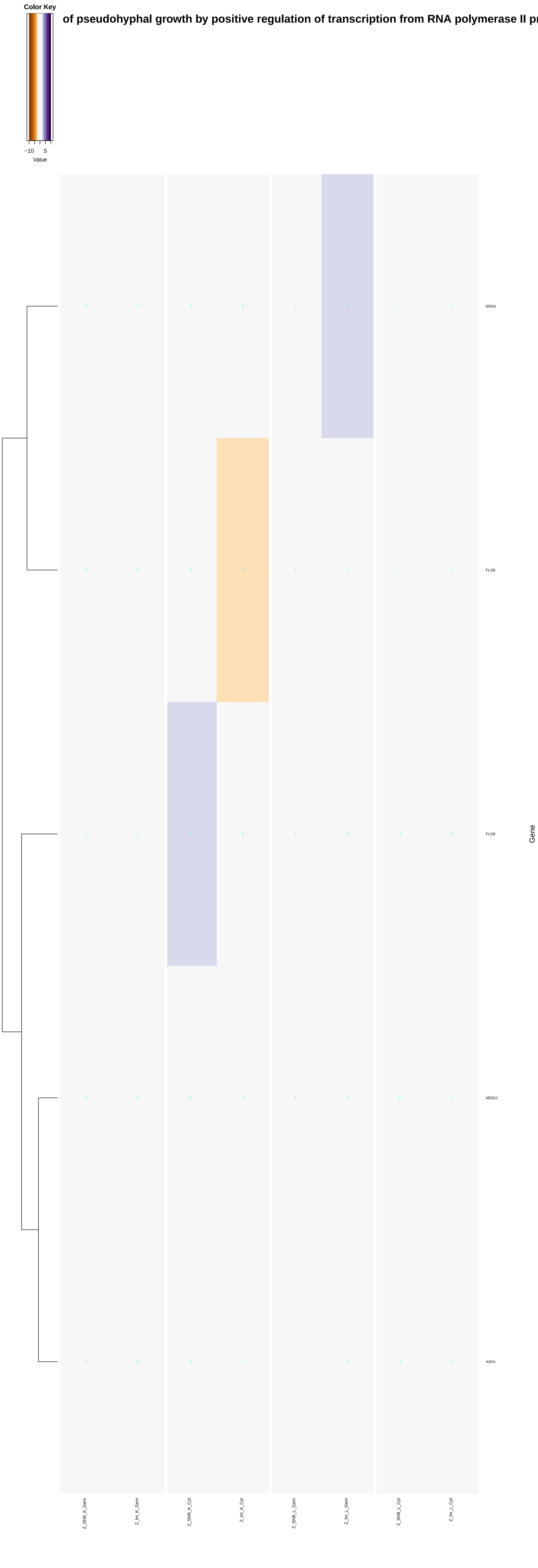
Color Key
−10 5
Value
of pseudohyphal growth by positive regulation of transcription from RNA polymerase II pr
MSN1
FLO8
FLO8
MSS11
ASH1
Gene
Z_Shift_K_Gem
Z_Im_K_Gem
Z_Shift_K_Cyt
Z_Im_K_Cyt
Z_Shift_L_Gem
Z_Im_L_Gem
Z_Shift_L_Cyt
Z_Im_L_Cyt

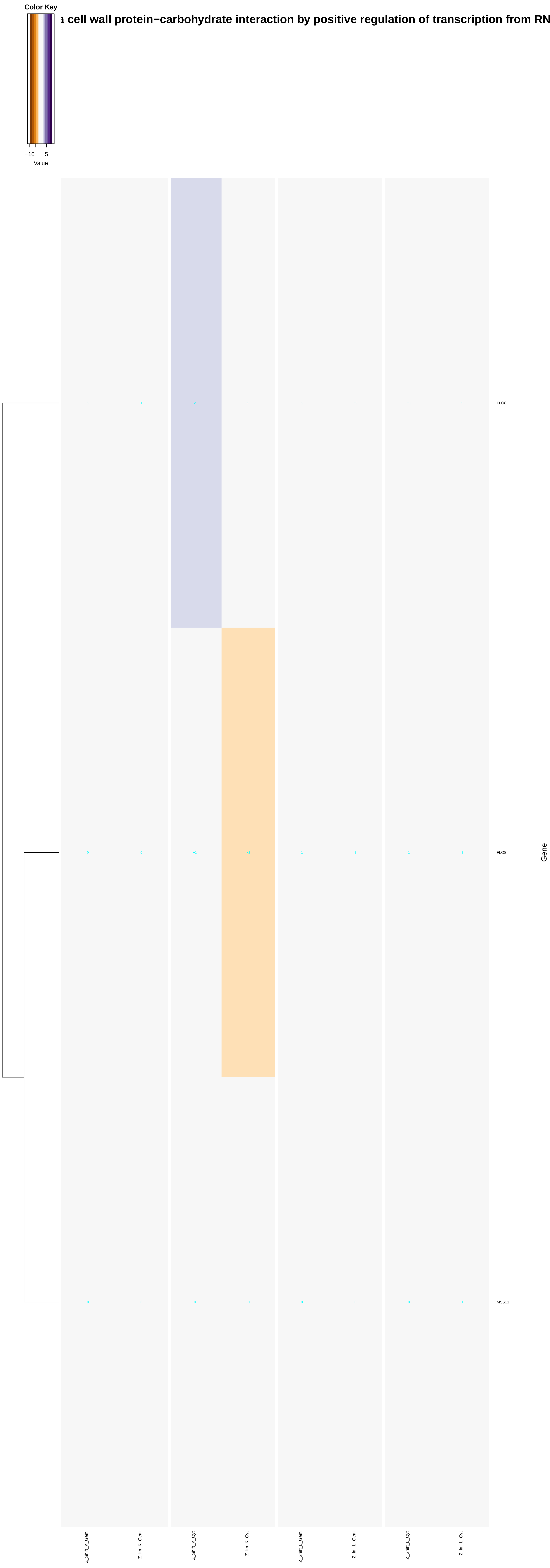
a cell wall protein−carbohydrate interaction by positive regulation of transcription from RN
Color Key
−10
5
Value
FLO8
FLO8
MSS11
Gene
Z_Shift_K_Gem
Z_Im_K_Gem
Z_Shift_K_Cyt
Z_Im_K_Cyt
Z_Shift_L_Gem
Z_Im_L_Gem
Z_Shift_L_Cyt
Z_Im_L_Cyt

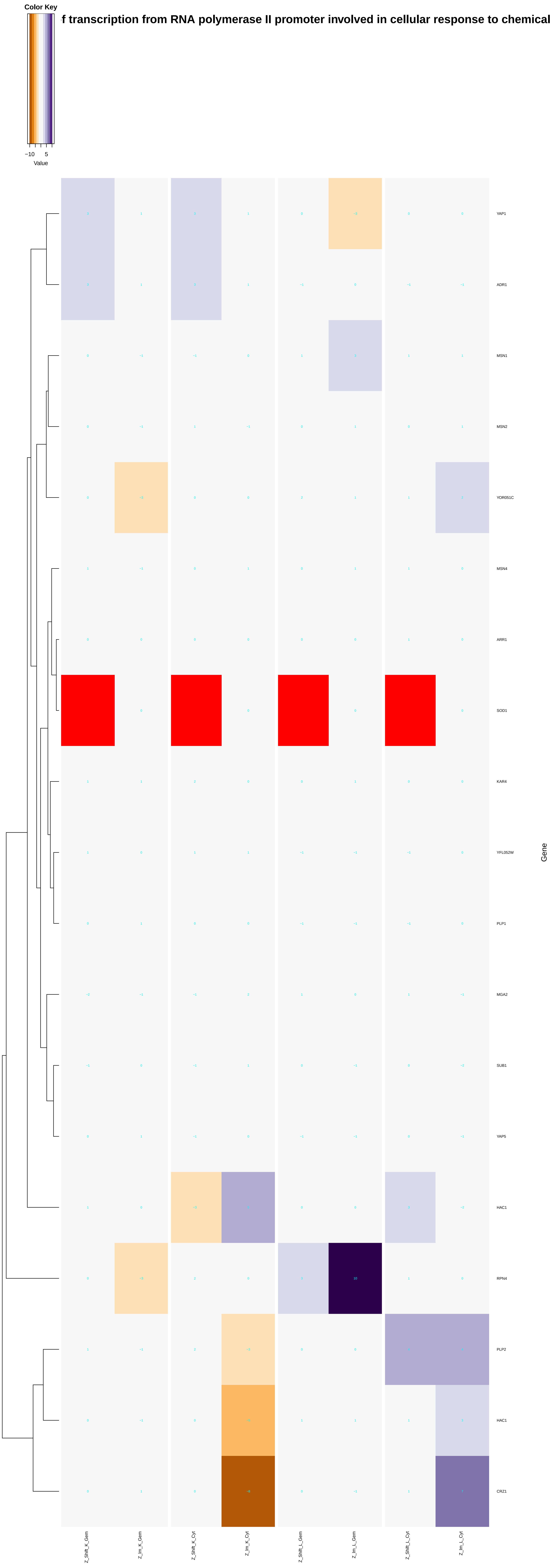
Color Key
−10
5
Value
f transcription from RNA polymerase II promoter involved in cellular response to chemical
YAP1
ADR1
MSN1
MSN2
YOR051C
MSN4
ARR1
SOD1
KAR4
YFL052W
PLP1
MGA2
SUB1
YAP5
HAC1
RPN4
PLP2
HAC1
CRZ1
Gene
Z_Shift_K_Gem
Z_lm_K_Gem
Z_Shift_K_Cyt
Z_lm_K_Cyt
Z_Shift_L_Gem
Z_lm_L_Gem
Z_Shift_L_Cyt
Z_lm_L_Cyt

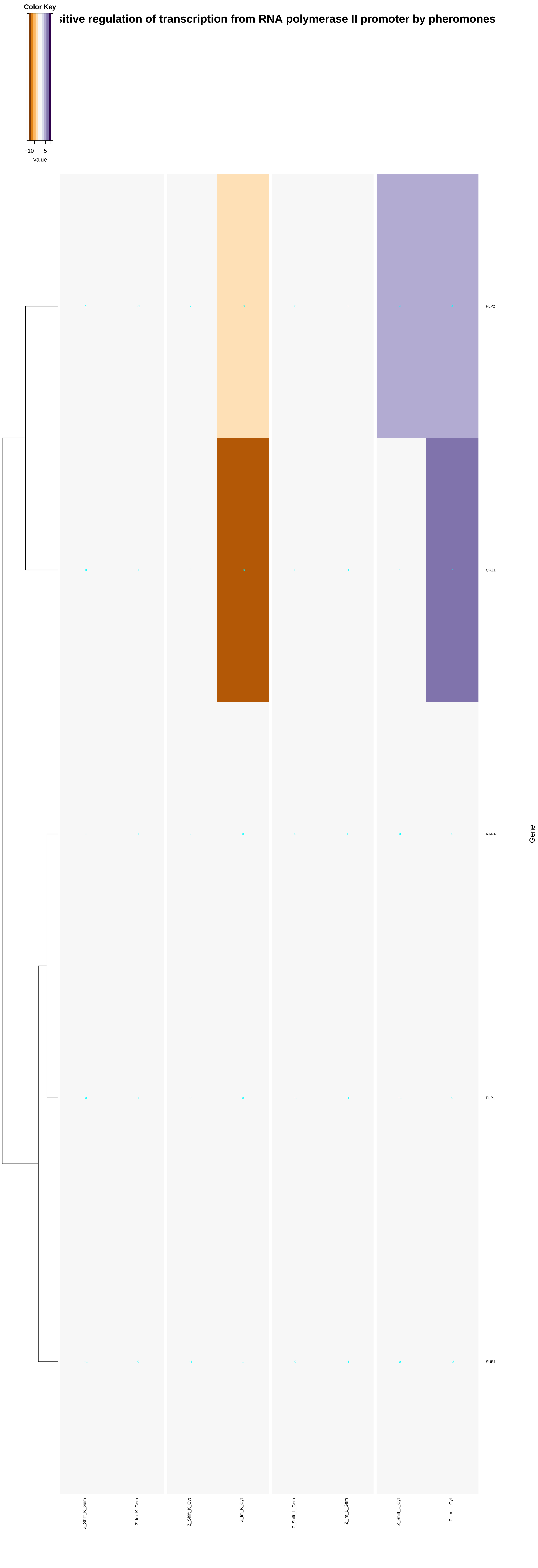
sitive regulation of transcription from RNA polymerase II promoter by pheromones
Color Key
−10
5
Value
PLP2
CRZ1
KAR4
PLP1
SUB1
Gene
Z_Shift_K_Gem
Z_Im_K_Gem
Z_Shift_K_Cyt
Z_Im_K_Cyt
Z_Shift_L_Gem
Z_Im_L_Gem
Z_Shift_L_Cyt
Z_Im_L_Cyt

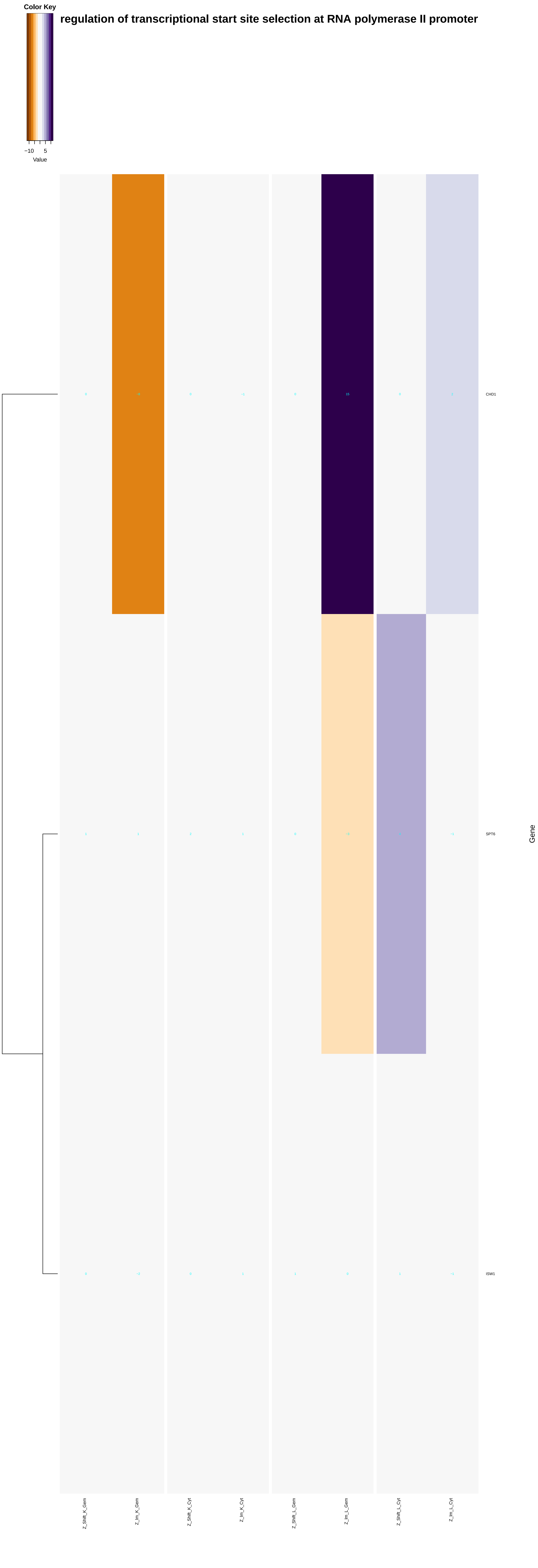
regulation of transcriptional start site selection at RNA polymerase II promoter
Color Key
−10
5
Value
CHD1
SPT6
ISW1
Gene
Z_Shift_K_Gem
Z_Im_K_Gem
Z_Shift_K_Cyt
Z_Im_K_Cyt
Z_Shift_L_Gem
Z_Im_L_Gem
Z_Shift_L_Cyt
Z_Im_L_Cyt

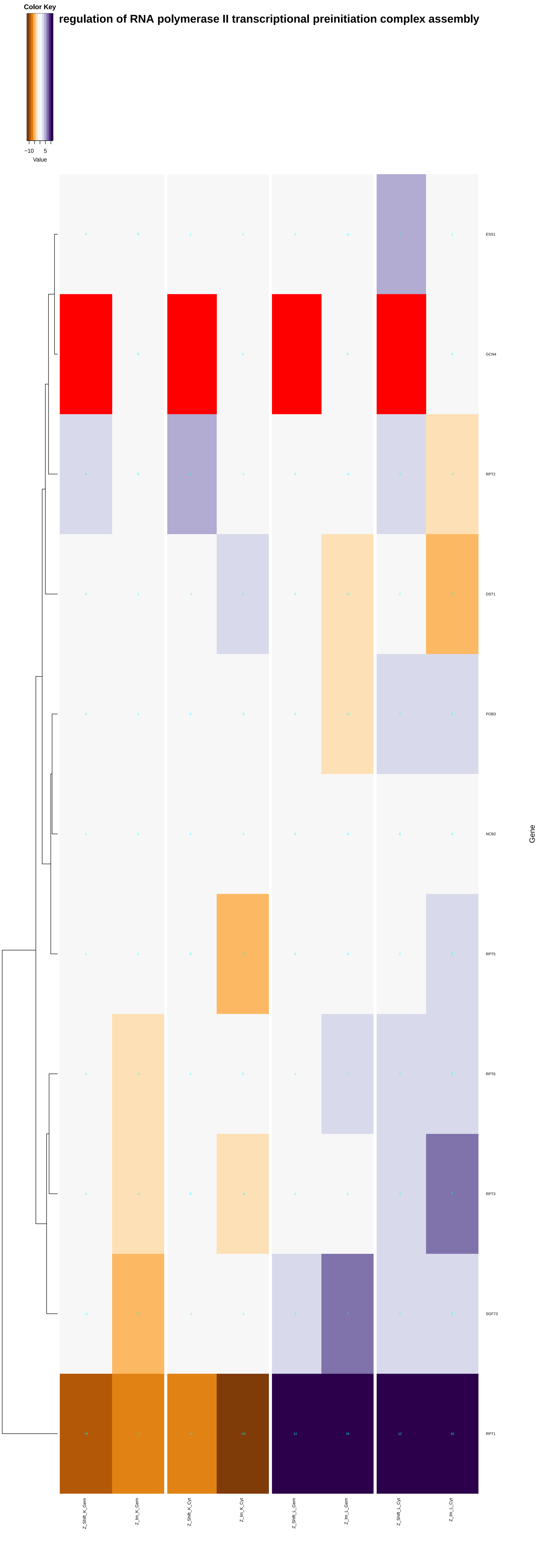
regulation of RNA polymerase II transcriptional preinitiation complex assembly
Color Key
Value
−10
5
ESS1
GCN4
RPT2
DST1
POB3
NCB2
RPT5
RPT6
RPT3
SGF73
RPT1
Gene
Z_Shift_K_Gem
Z_Im_K_Gem
Z_Shift_K_Cyt
Z_Im_K_Cyt
Z_Shift_L_Gem
Z_Im_L_Gem
Z_Shift_L_Cyt
Z_Im_L_Cyt

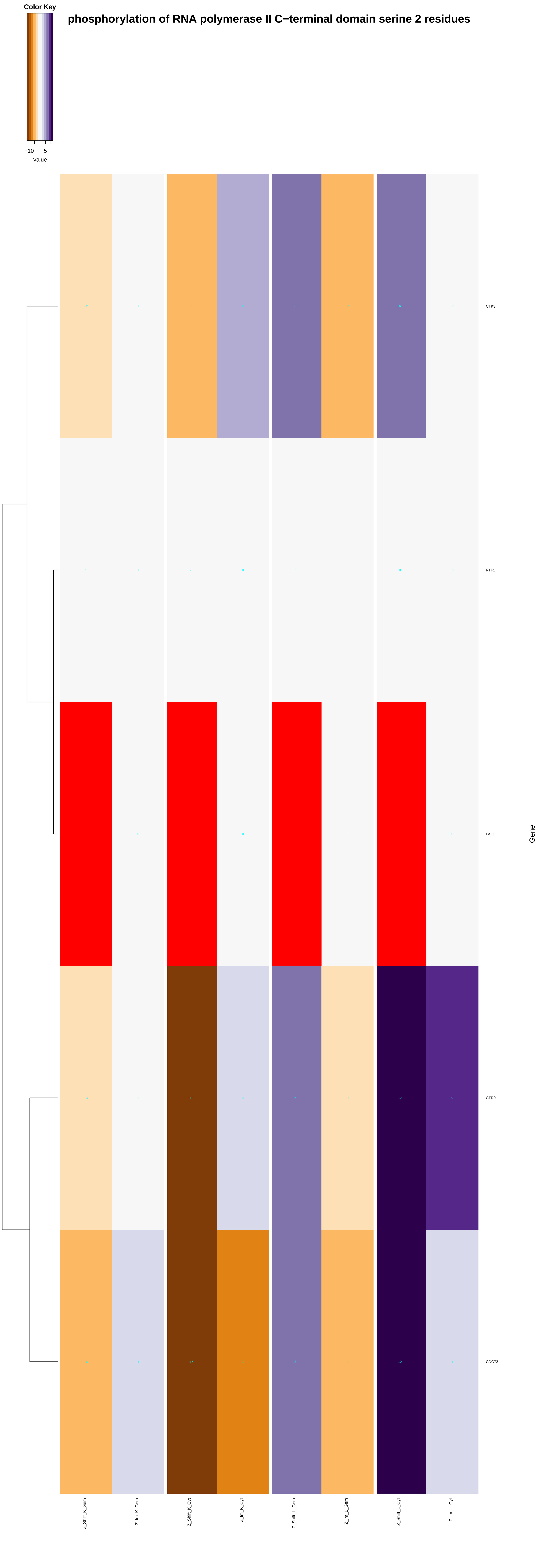
phosphorylation of RNA polymerase II C−terminal domain serine 2 residues
Color Key
−10
5
Value
CTK3
RTF1
PAF1
CTR9
CDC73
Gene
Z_Shift_K_Gem
Z_Im_K_Gem
Z_Shift_K_Cyt
Z_Im_K_Cyt
Z_Shift_L_Gem
Z_Im_L_Gem
Z_Shift_L_Cyt
Z_Im_L_Cyt

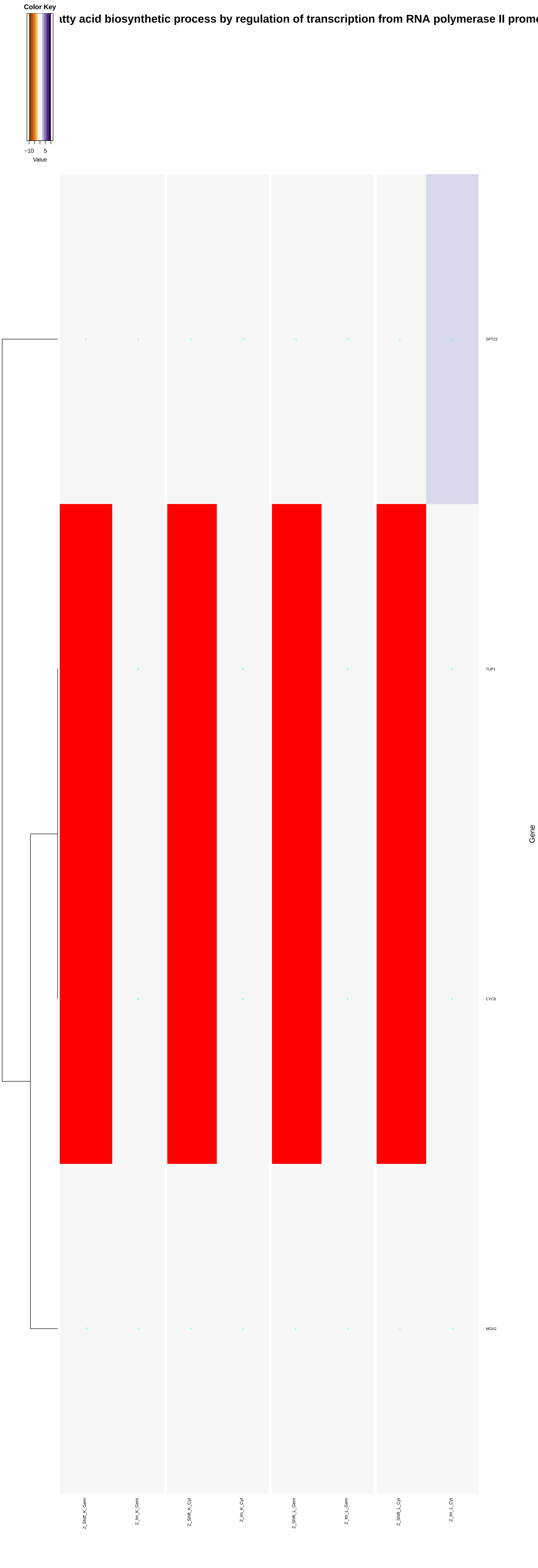
Color Key
−10
5
Value
atty acid biosynthetic process by regulation of transcription from RNA polymerase II prom
SPT23
TUP1
CYC8
MGA2
Gene
Z_Shift_K_Gem
Z_Im_K_Gem
Z_Shift_K_Cyt
Z_Im_K_Cyt
Z_Shift_L_Gem
Z_Im_L_Gem
Z_Shift_L_Cyt
Z_Im_L_Cyt

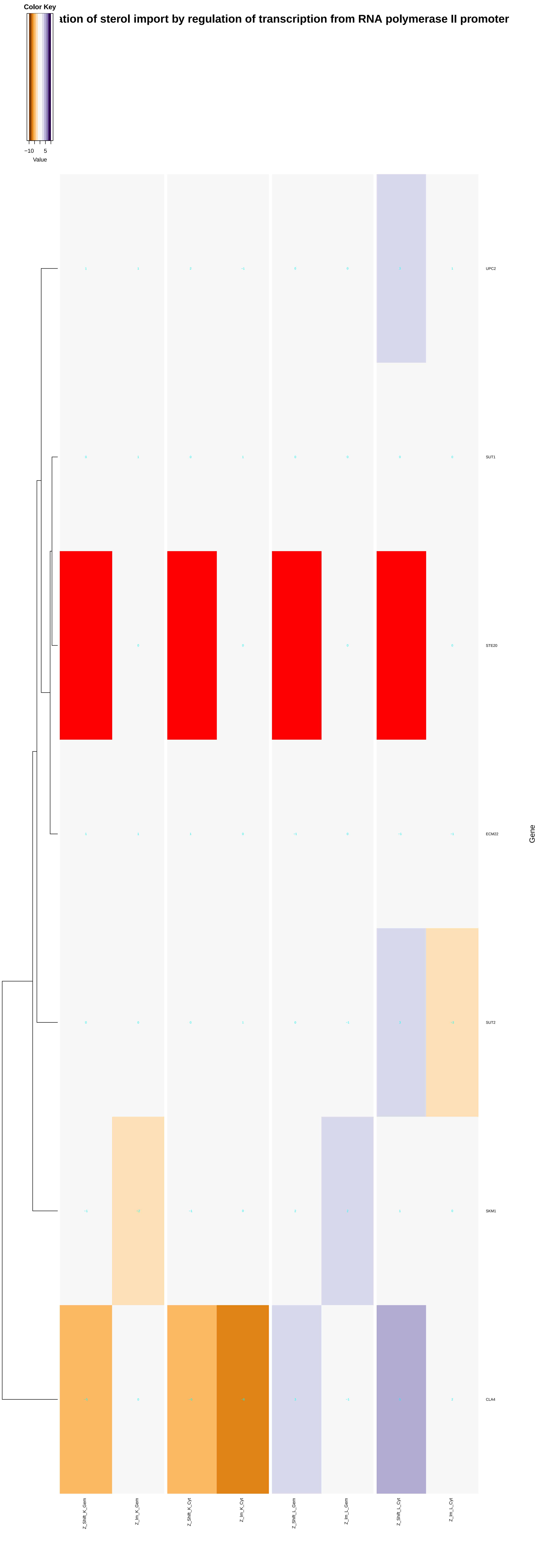
ation of sterol import by regulation of transcription from RNA polymerase II promoter
Color Key
−10
5
Value
UPC2
SUT1
STE20
ECM22
SUT2
SKM1
CLA4
Gene
Z_Shift_K_Gem
Z_Im_K_Gem
Z_Shift_K_Cyt
Z_Im_K_Cyt
Z_Shift_L_Gem
Z_Im_L_Gem
Z_Shift_L_Cyt
Z_Im_L_Cyt

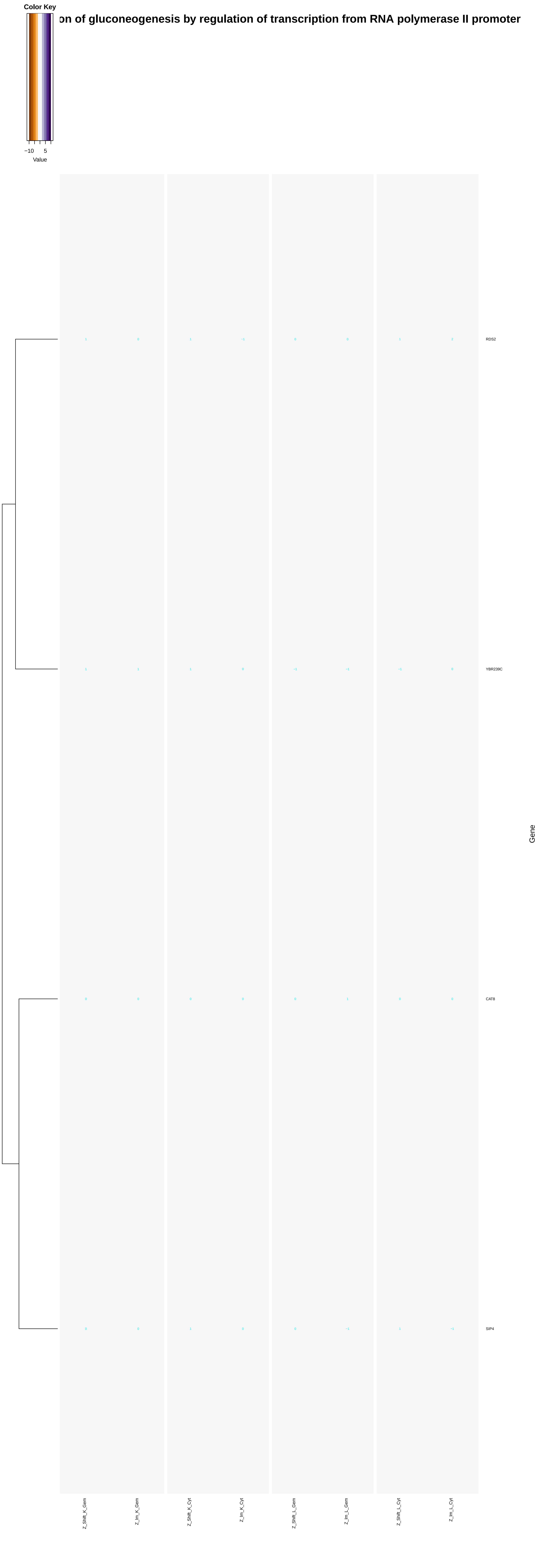

Color Key
−10
5
Value
on of gluconeogenesis by regulation of transcription from RNA polymerase II promoter
1 0 1 −1 0 0 1 2
RDS2
1 1 1 0 −1 −1 −1 0
YBR239C
0 0 0 0 0 1 0 0
CAT8
0 0 1 0 0 −1 1 −1
SIP4
Gene
Z_Shift_K_Gem
Z_Im_K_Gem
Z_Shift_K_Cyt
Z_Im_K_Cyt
Z_Shift_L_Gem
Z_Im_L_Gem
Z_Shift_L_Cyt
Z_Im_L_Cyt

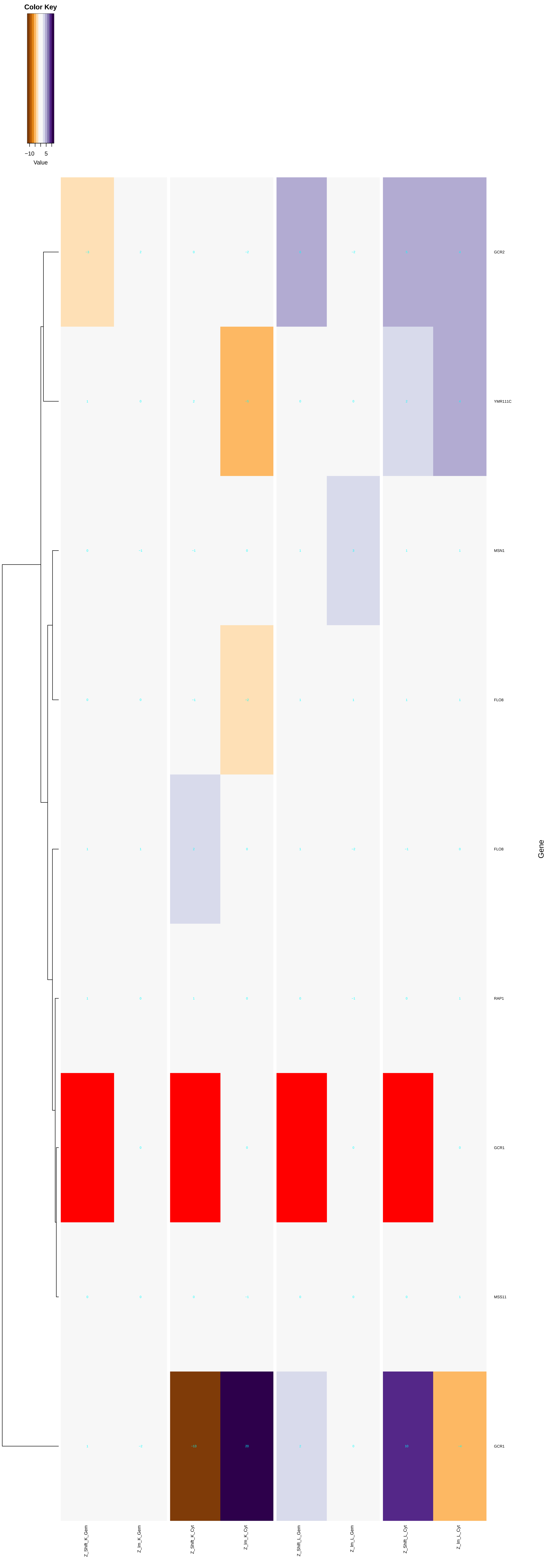

Color Key
−10
5
Value
GCR2
YMR111C
MSN1
FLO8
FLO8
RAP1
GCR1
MSS11
GCR1
Gene
Z_Shift_K_Gem
Z_Im_K_Gem
Z_Shift_K_Cyt
Z_Im_K_Cyt
Z_Shift_L_Gem
Z_Im_L_Gem
Z_Shift_L_Cyt
Z_Im_L_Cyt

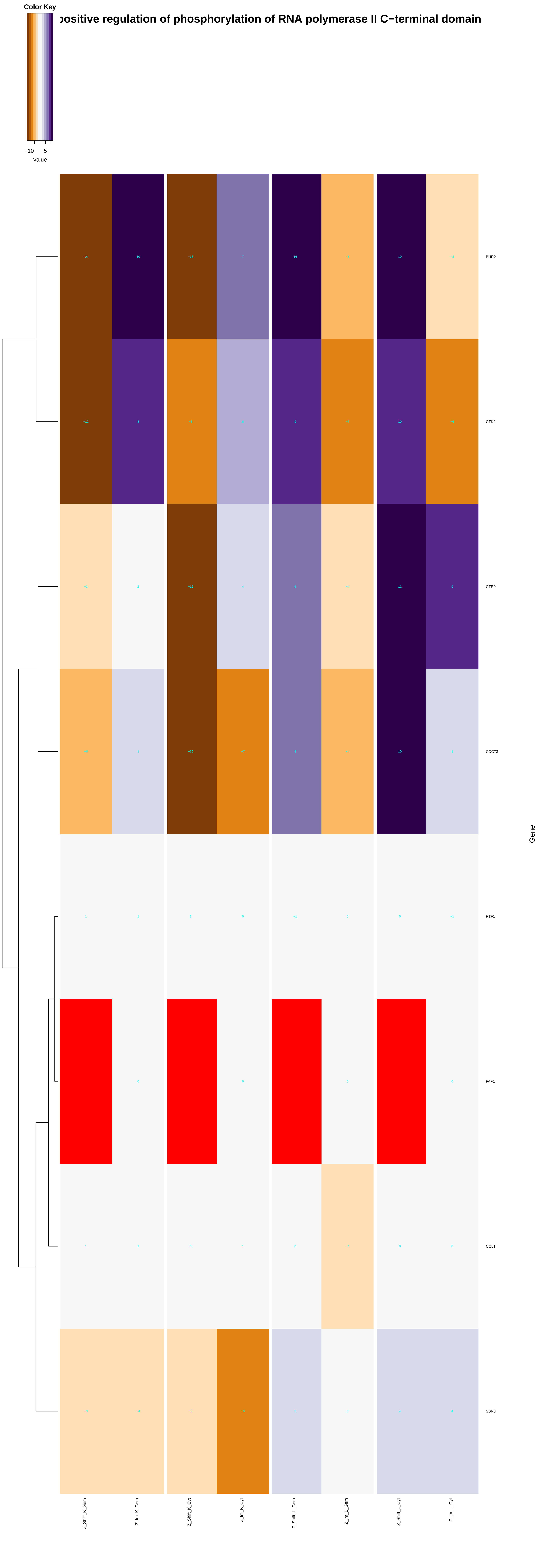

Color Key
−10
5
Value
ɔositive regulation of phosphorylation of RNA polymerase II C−terminal domain
BUR2
CTK2
CTR9
CDC73
RTF1
PAF1
CCL1
SSN8
Gene
Z_Shift_K_Gem
Z_Im_K_Gem
Z_Shift_K_Cyt
Z_Im_K_Cyt
Z_Shift_L_Gem
Z_Im_L_Gem
Z_Shift_L_Cyt
Z_Im_L_Cyt

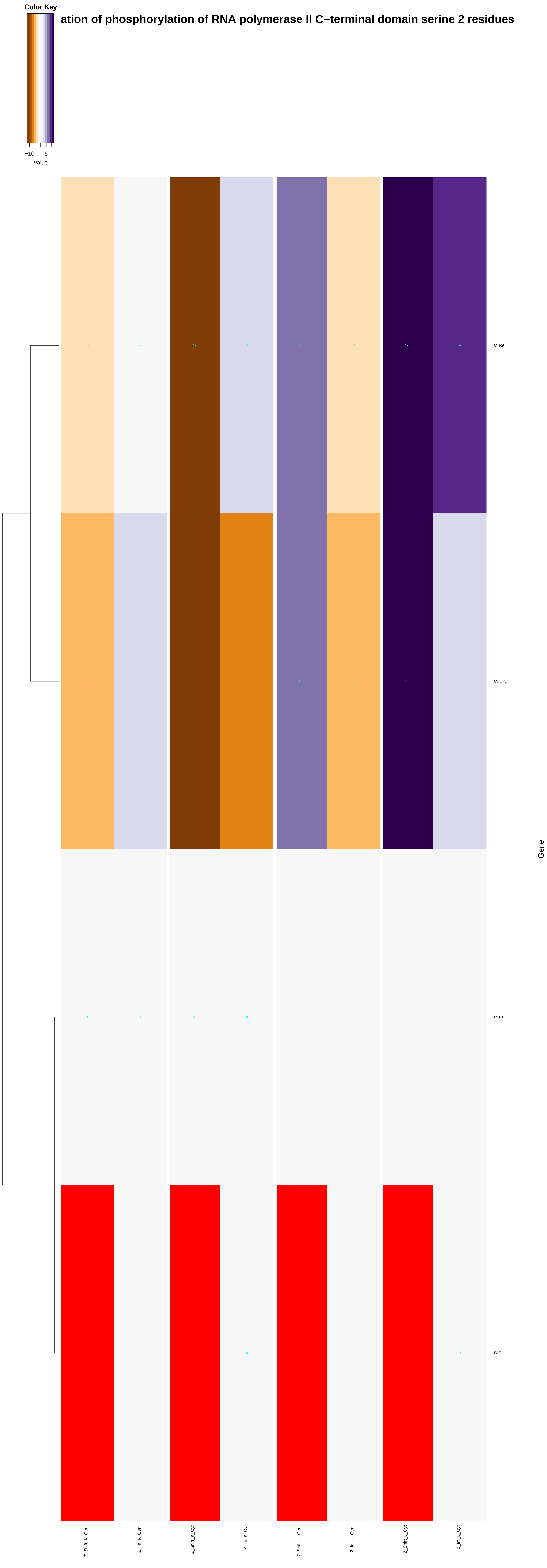
Color Key
−10
5
Value
ation of phosphorylation of RNA polymerase II C−terminal domain serine 2 residues
CTR9
CDC73
RTF1
PAF1
Gene
Z_Shift_K_Gem
Z_Im_K_Gem
Z_Shift_K_Cyt
Z_Im_K_Cyt
Z_Shift_L_Gem
Z_Im_L_Gem
Z_Shift_L_Cyt
Z_Im_L_Cyt

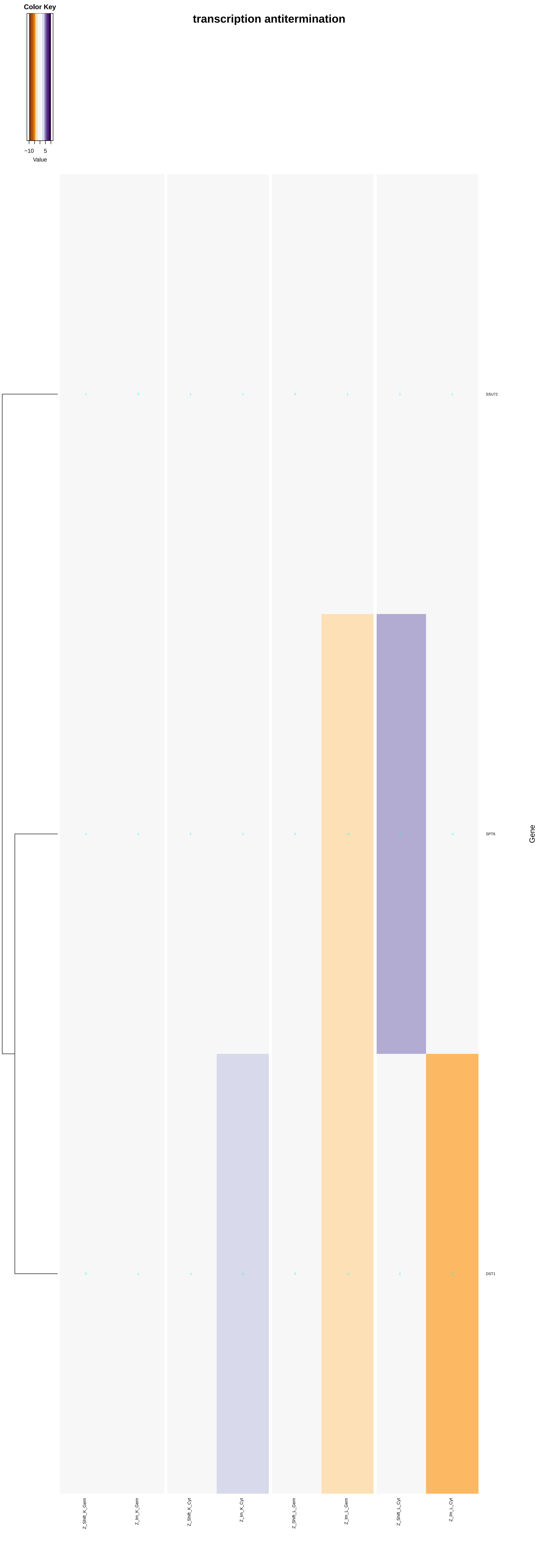
Color Key
−10
5
Value
transcription antitermination
SSU72
SPT6
DST1
Gene
Z_Shift_K_Gem
Z_Im_K_Gem
Z_Shift_K_Cyt
Z_Im_K_Cyt
Z_Shift_L_Gem
Z_Im_L_Gem
Z_Shift_L_Cyt
Z_Im_L_Cyt

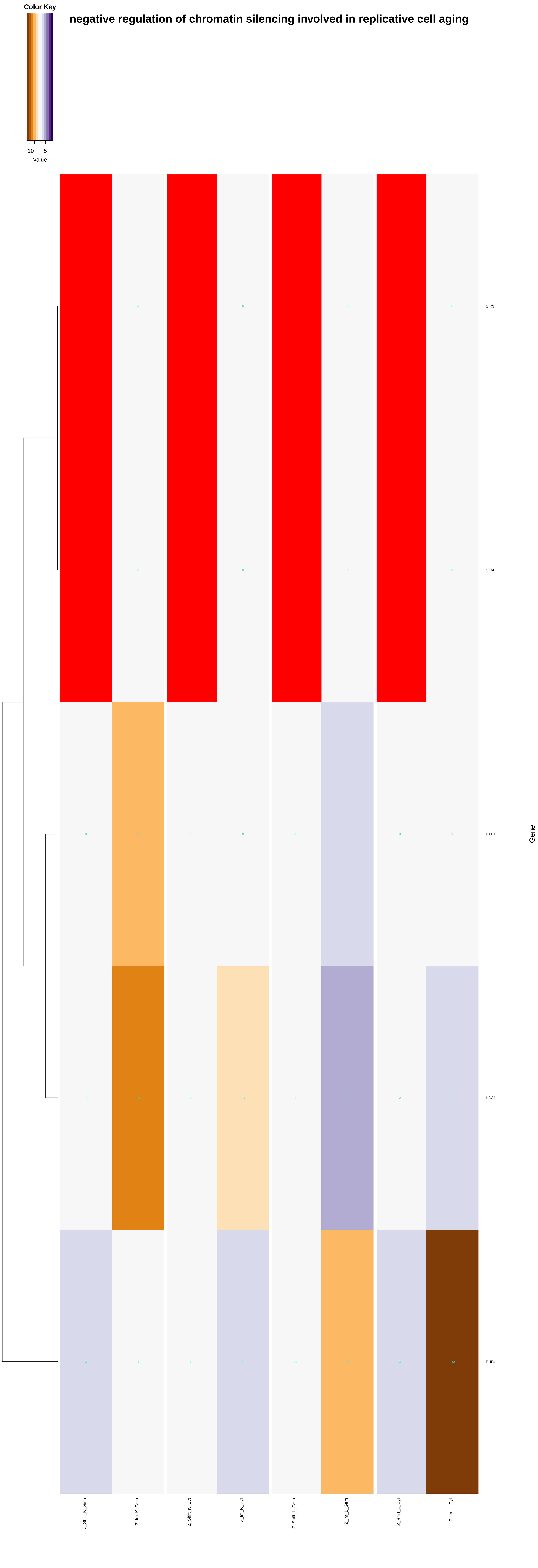
negative regulation of chromatin silencing involved in replicative cell aging
Color Key
−10
5
Value
SIR3
SIR4
UTH1
HDA1
PUF4
Gene
Z_Shift_K_Gem
Z_Im_K_Gem
Z_Shift_K_Cyt
Z_Im_K_Cyt
Z_Shift_L_Gem
Z_Im_L_Gem
Z_Shift_L_Cyt
Z_Im_L_Cyt

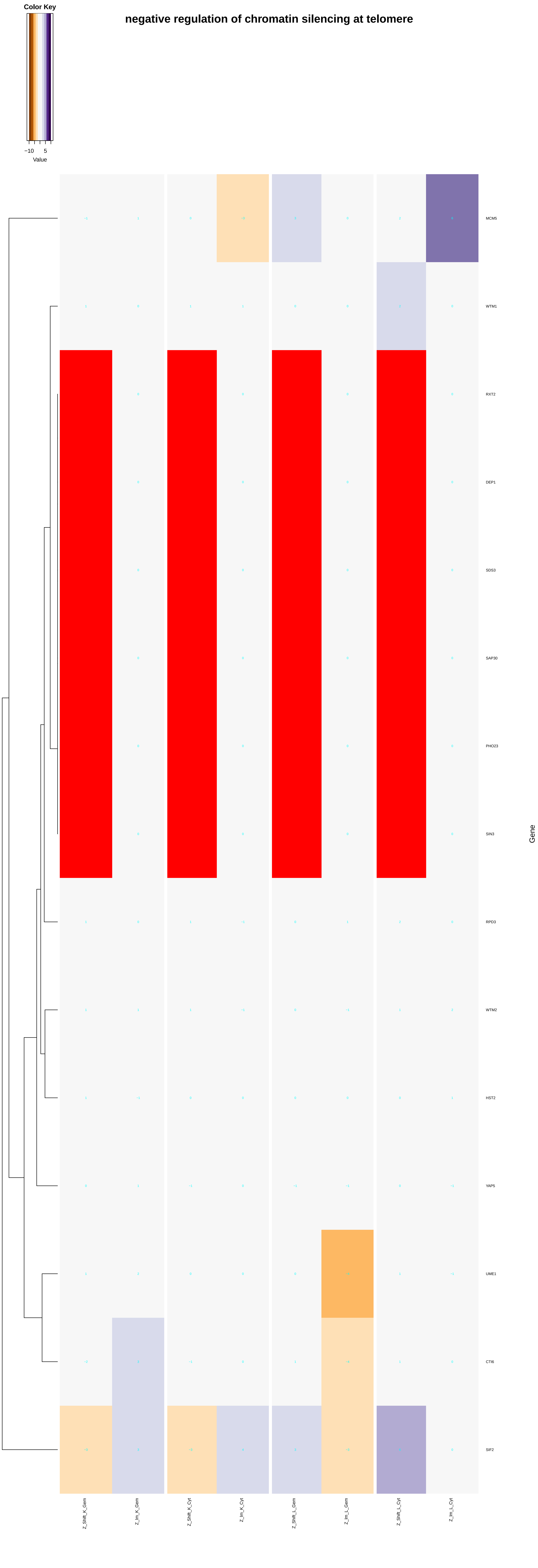
negative regulation of chromatin silencing at telomere
Color Key
−10
5
Value
MCM5
WTM1
RXT2
DEP1
SDS3
SAP30
PHO23
SIN3
RPD3
WTM2
HST2
YAP5
UME1
CTI6
SIF2
Gene
Z_Shift_K_Gem
Z_Im_K_Gem
Z_Shift_K_Cyt
Z_Im_K_Cyt
Z_Shift_L_Gem
Z_Im_L_Gem
Z_Shift_L_Cyt
Z_Im_L_Cyt

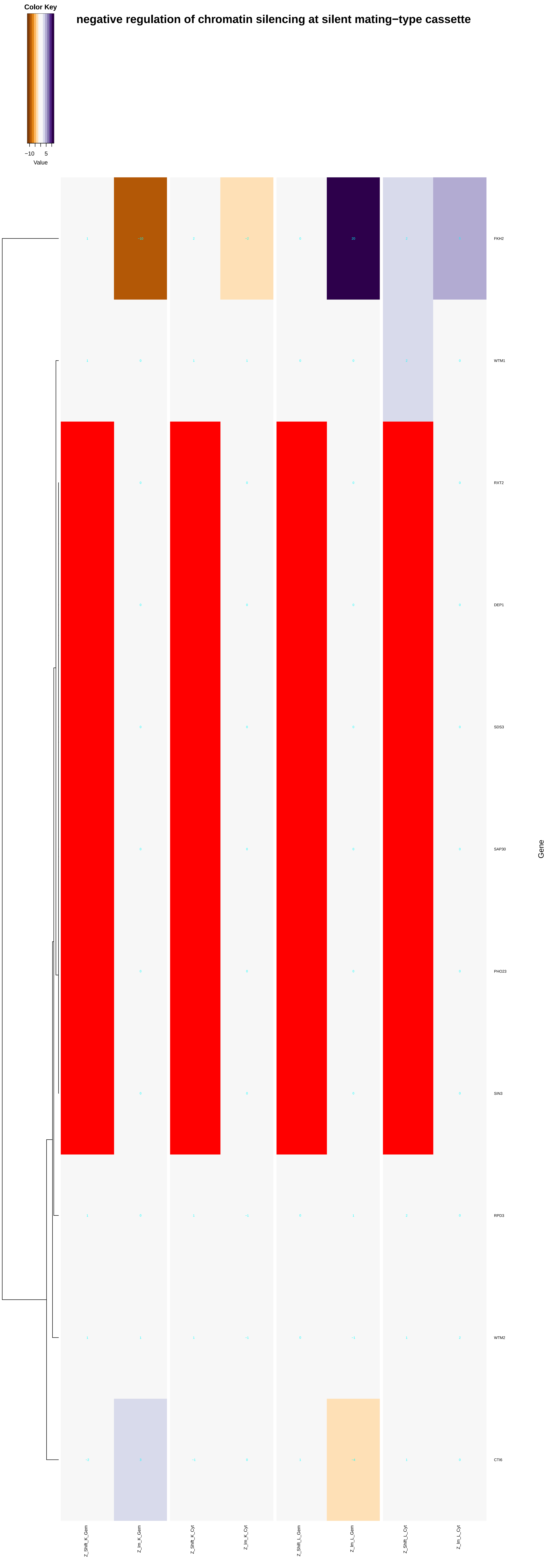
negative regulation of chromatin silencing at silent mating−type cassette
Color Key
−10
5
Value
FKH2
WTM1
RXT2
DEP1
SDS3
SAP30
PHO23
SIN3
RPD3
WTM2
CTI6
Gene
Z_Shift_K_Gem
Z_Im_K_Gem
Z_Shift_K_Cyt
Z_Im_K_Cyt
Z_Shift_L_Gem
Z_Im_L_Gem
Z_Shift_L_Cyt
Z_Im_L_Cyt

negative regulation of chromatin silencing at rDNA

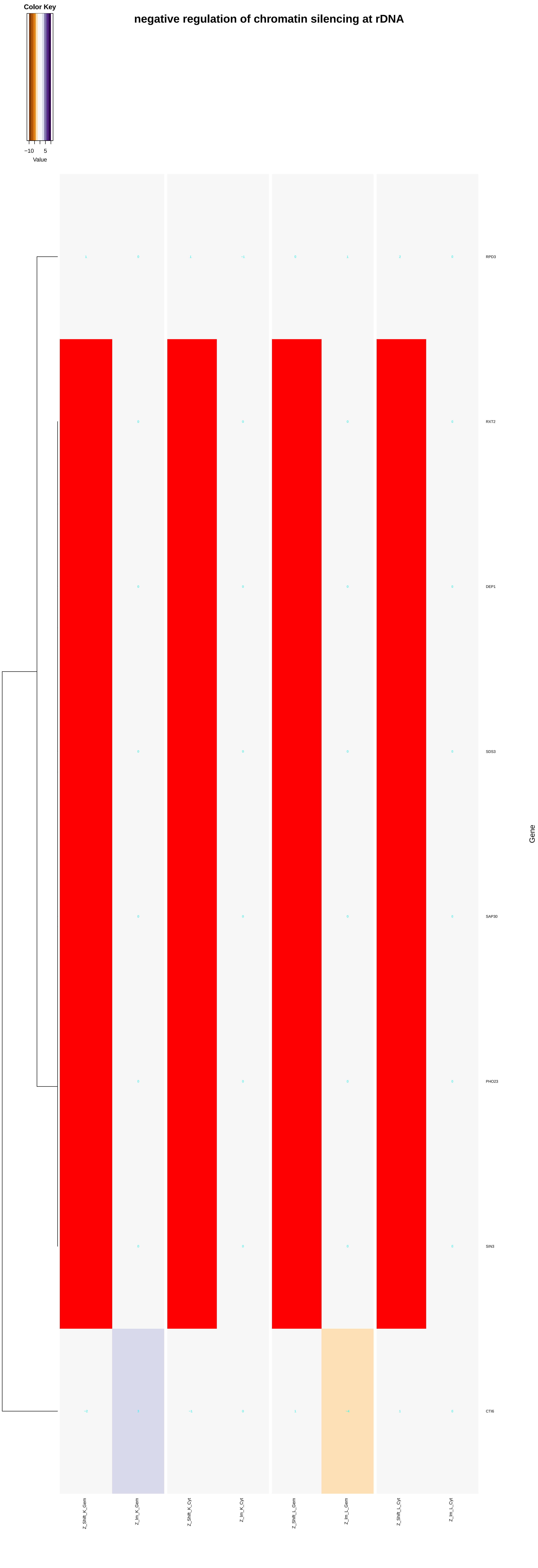

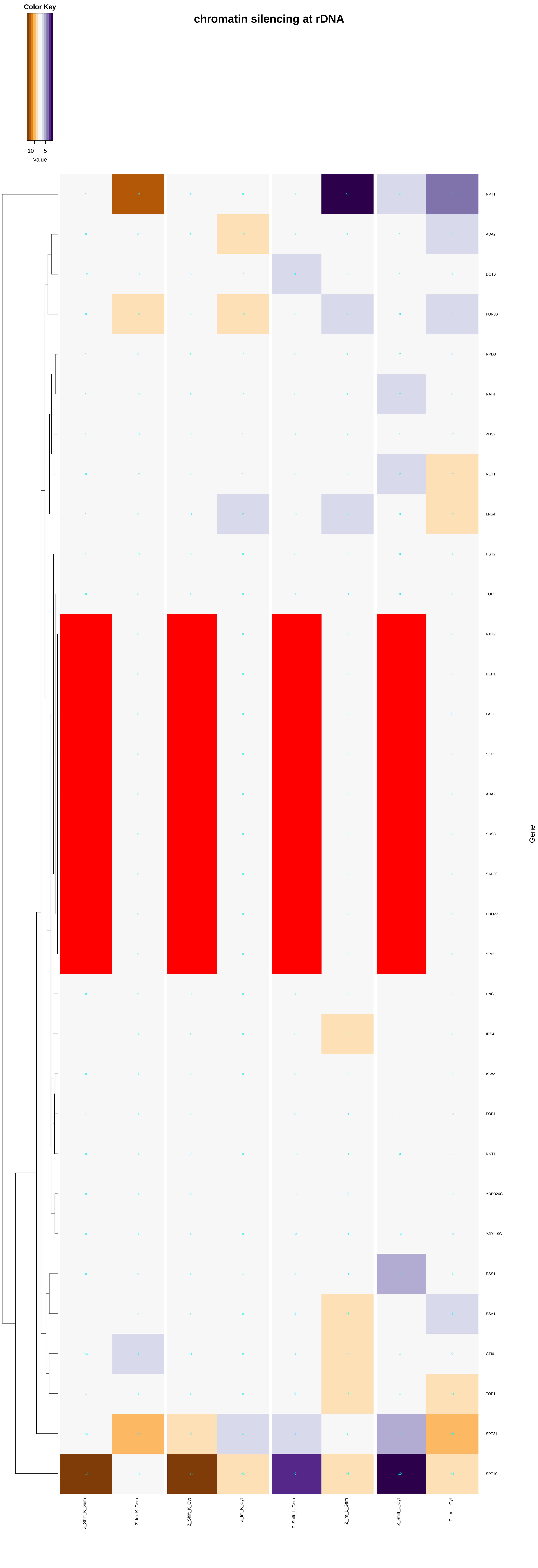

chromatin silencing at rDNA
Color Key
−10
5
Value
Gene
NPT1
ADA2
DOT6
FUN30
RPD3
NAT4
ZDS2
NET1
LRS4
HST2
TOF2
RXT2
DEP1
PAF1
SIR2
ADA2
SDS3
SAP30
PHO23
SIN3
PNC1
IRS4
ISW2
FOB1
NNT1
YDR026C
YJR119C
ESS1
ESA1
CTI6
TOP1
SPT21
SPT10
Z_Shift_K_Gem
Z_lm_K_Gem
Z_Shift_K_Cyt
Z_lm_K_Cyt
Z_Shift_L_Gem
Z_lm_L_Gem
Z_Shift_L_Cyt
Z_lm_L_Cyt

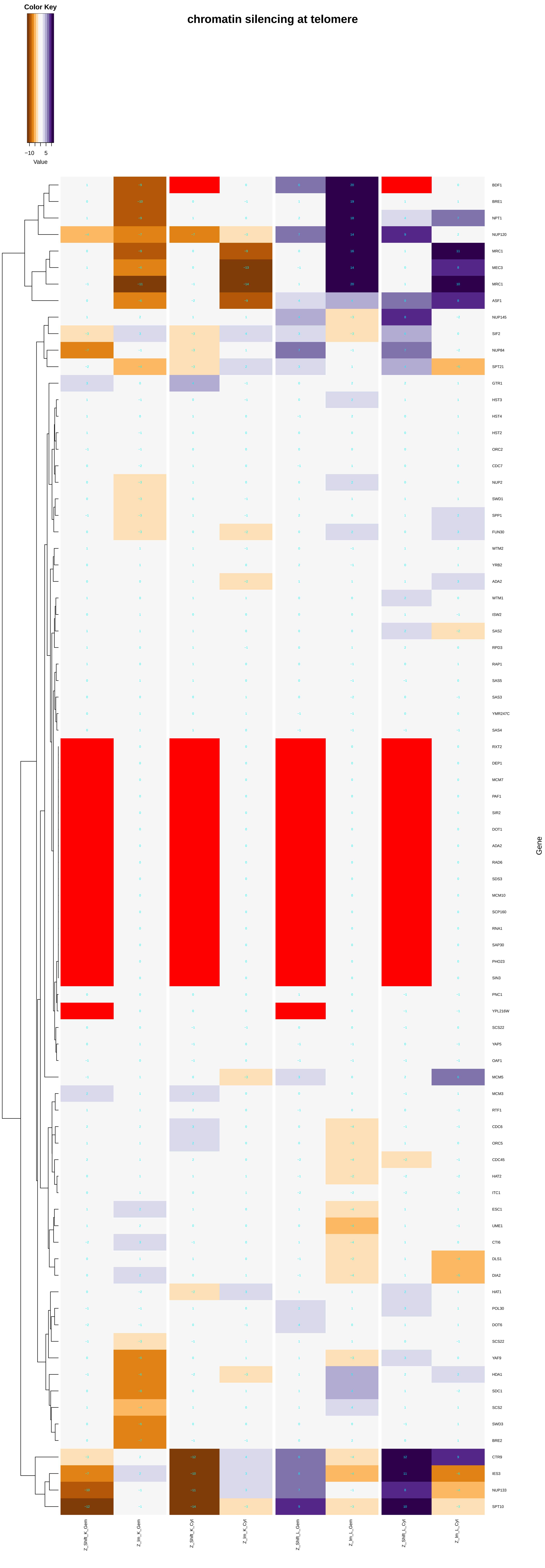

chromatin silencing at telomere
Color Key
−10
5
Value
Gene
BDF1
BRE1
NPT1
NUP120
MRC1
MEC3
MRC1
ASF1
NUP145
SIF2
NUP84
SPT21
GTR1
HST3
HST4
HST2
ORC2
CDC7
NUP2
SWD1
SPP1
FUN30
WTM2
YRB2
ADA2
WTM1
ISW2
SAS2
RPD3
RAP1
SAS5
SAS3
YMR247C
SAS4
RXT2
DEP1
MCM7
PAF1
SIR2
DOT1
ADA2
RAD6
SDS3
MCM10
SCP160
RNA1
SAP30
PHO23
SIN3
PNC1
YPL216W
SCS22
YAP5
OAF1
MCM5
MCM3
RTF1
CDC6
ORC5
CDC45
HAT2
ITC1
ESC1
UME1
CTI6
DLS1
DIA2
HAT1
POL30
DOT6
SCS22
YAF9
HDA1
SDC1
SCS2
SWD3
BRE2
CTR9
IES3
NUP133
SPT10
Z_Shift_K_Gem
Z_lm_K_Gem
Z_Shift_K_Cyt
Z_lm_K_Cyt
Z_Shift_L_Gem
Z_lm_L_Gem
Z_Shift_L_Cyt
Z_lm_L_Cyt

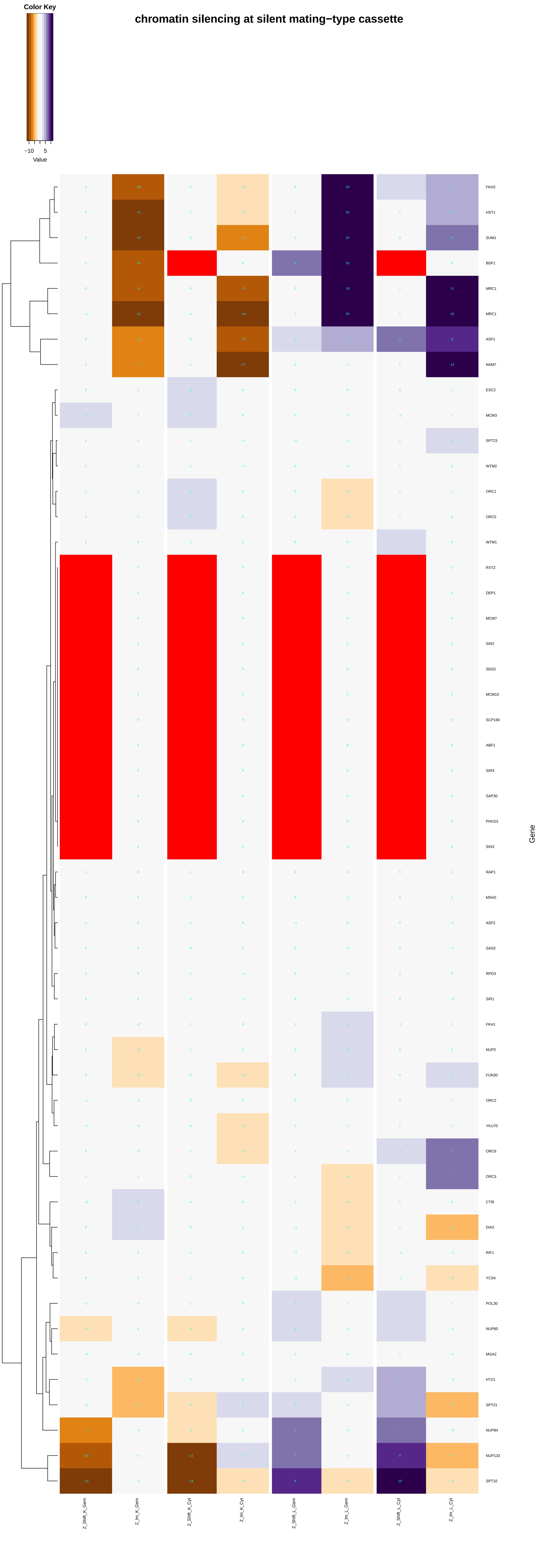
chromatin silencing at silent mating−type cassette
Color Key
−10
5
Value
FKH2
HST1
SUM1
BDF1
MRC1
MRC1
ASF1
NAM7
ESC2
MCM3
SPT23
WTM2
ORC1
ORC5
WTM1
RXT2
DEP1
MCM7
SIR2
SDS3
MCM10
SCP160
ABF1
SIR3
SAP30
PHO23
SIN3
RAP1
MSH2
ASF2
SAS3
RPD3
SIR1
FKH1
NUP2
FUN30
ORC2
YKU70
ORC6
ORC3
CTI6
DIA2
RIF1
YCS4
POL30
NUP60
MGA2
HTZ1
SPT21
NUP84
NUP133
SPT10
Gene
Z_Shift_K_Gem
Z_Im_K_Gem
Z_Shift_K_Cyt
Z_Im_K_Cyt
Z_Shift_L_Gem
Z_Im_L_Gem
Z_Shift_L_Cyt
Z_Im_L_Cyt

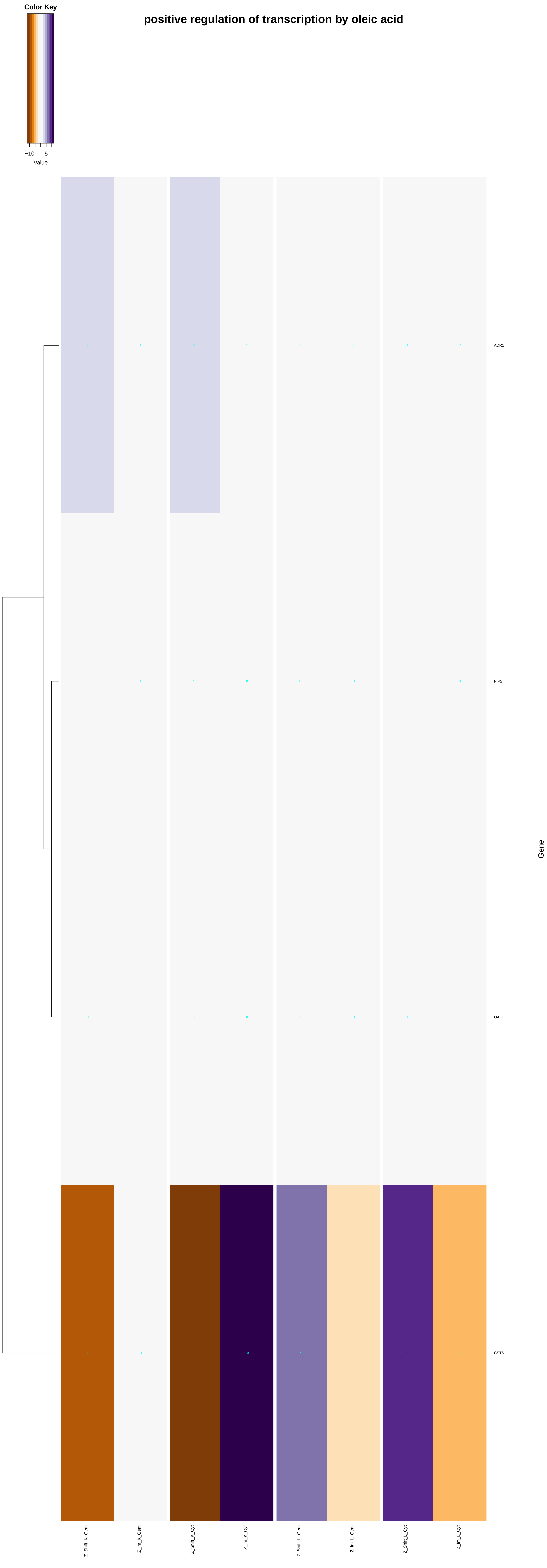
positive regulation of transcription by oleic acid
Color Key
−10
5
Value
ADR1
PIP2
OAF1
CST6
Gene
Z_Shift_K_Gem
Z_lm_K_Gem
Z_Shift_K_Cyt
Z_lm_K_Cyt
Z_Shift_L_Gem
Z_lm_L_Gem
Z_Shift_L_Cyt
Z_lm_L_Cyt

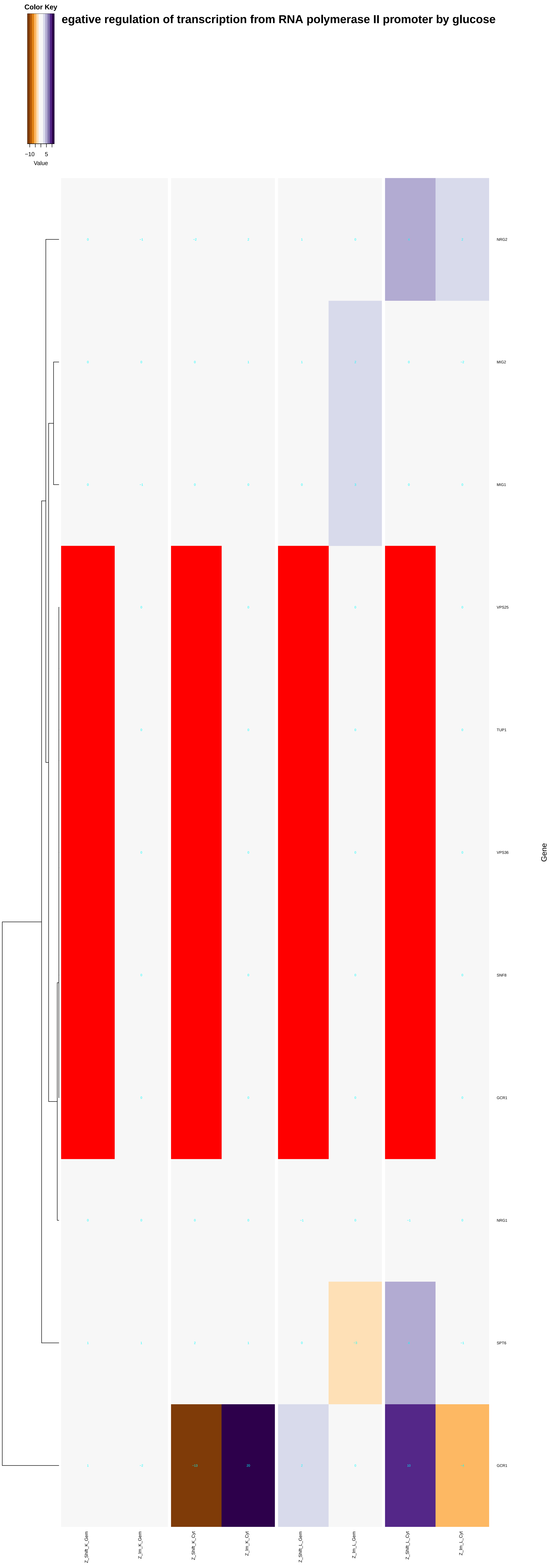

Color Key
−10
5
Value
egative regulation of transcription from RNA polymerase II promoter by glucose
NRG2
MIG2
MIG1
VPS25
TUP1
VPS36
SNF8
GCR1
NRG1
SPT6
GCR1
Gene
Z_Shift_K_Gem
Z_Im_K_Gem
Z_Shift_K_Cyt
Z_Im_K_Cyt
Z_Shift_L_Gem
Z_Im_L_Gem
Z_Shift_L_Cyt
Z_Im_L_Cyt

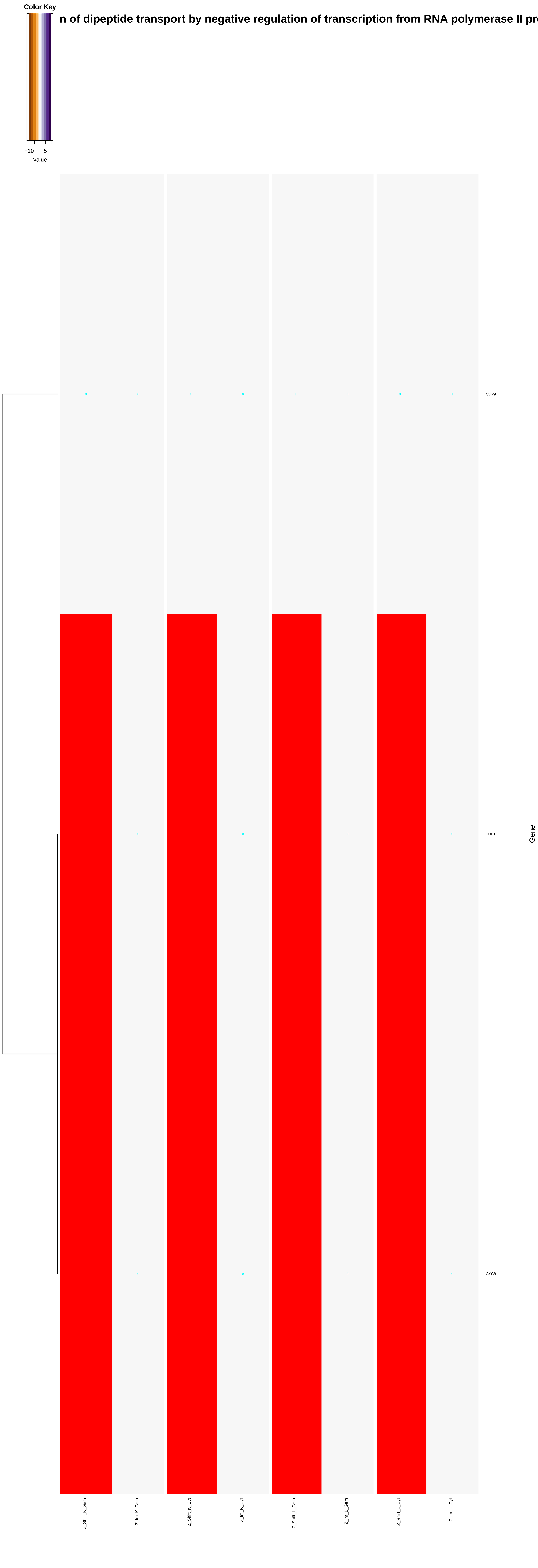

n of dipeptide transport by negative regulation of transcription from RNA polymerase II pr
Color Key
−10
5
Value
CUP9
TUP1
CYC8
Gene
Z_Shift_K_Gem
Z_Im_K_Gem
Z_Shift_K_Cyt
Z_Im_K_Cyt
Z_Shift_L_Gem
Z_Im_L_Gem
Z_Shift_L_Cyt
Z_Im_L_Cyt

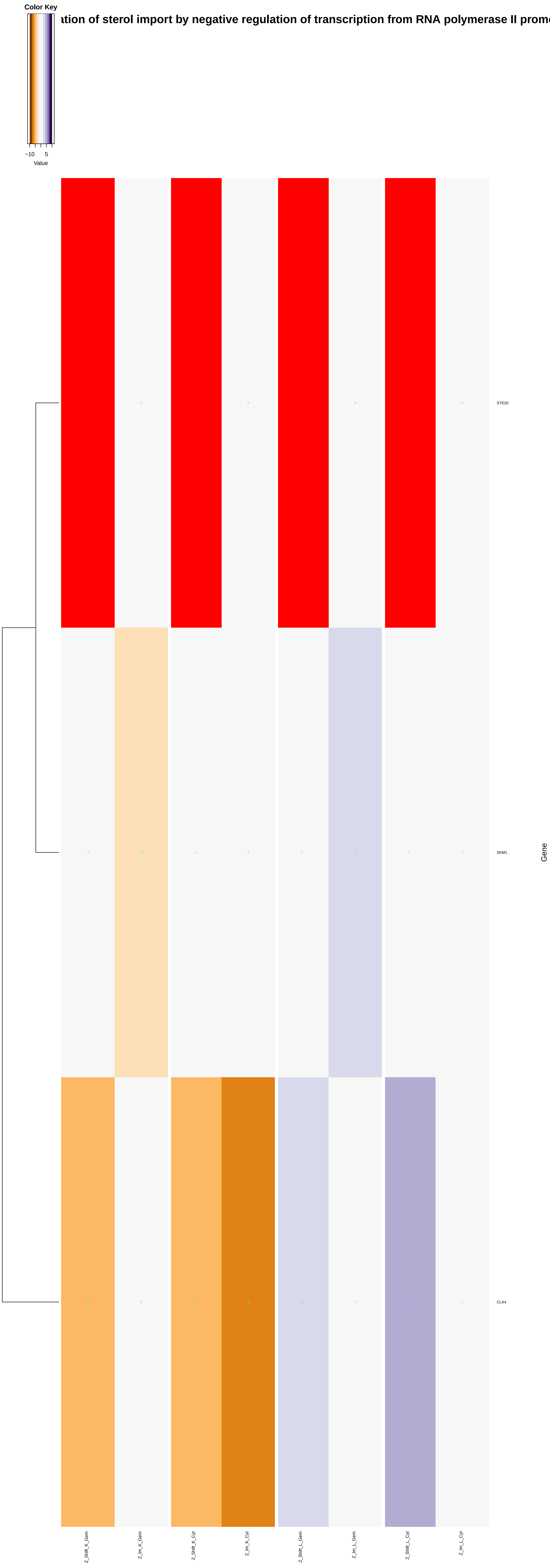
ation of sterol import by negative regulation of transcription from RNA polymerase II prom
Color Key
−10
5
Value
STE20
SKM1
CLA4
Gene
Z_Shift_K_Gem
Z_Im_K_Gem
Z_Shift_K_Cyt
Z_Im_K_Cyt
Z_Shift_L_Gem
Z_Im_L_Gem
Z_Shift_L_Cyt
Z_Im_L_Cyt

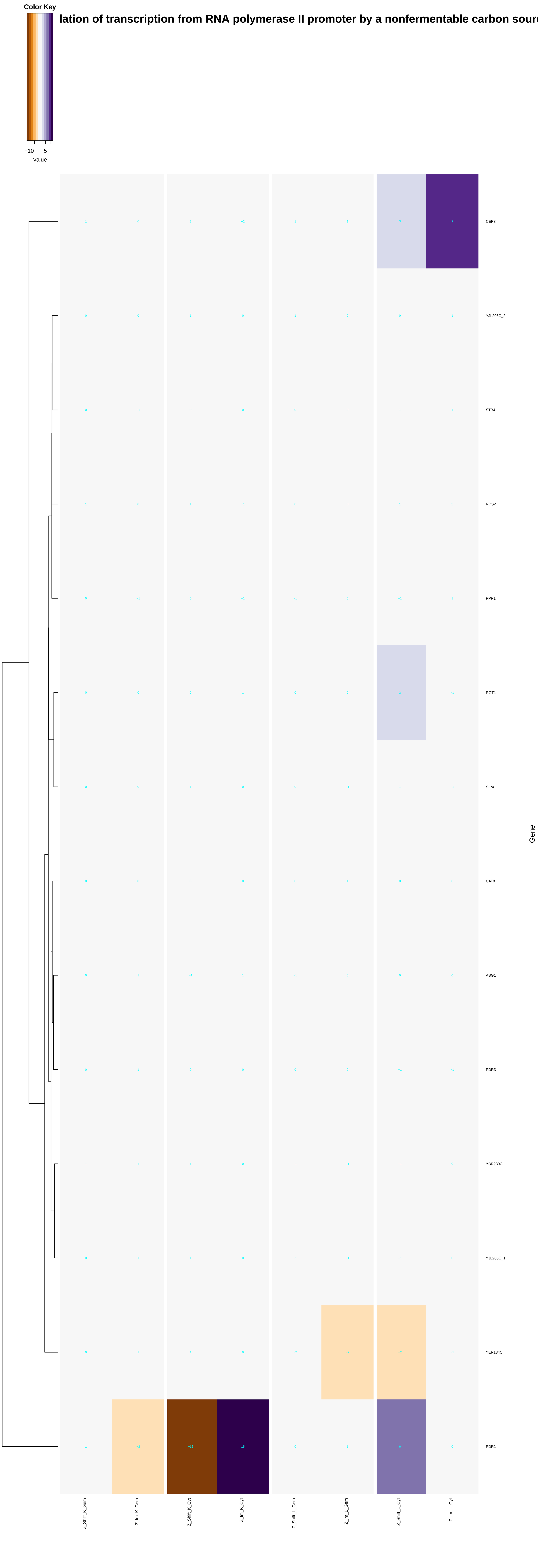

lation of transcription from RNA polymerase II promoter by a nonfermentable carbon sourc
Color Key
−10
5
Value
CEP3
YJL206C_2
STB4
RDS2
PPR1
RGT1
SIP4
CAT8
ASG1
PDR3
YBR239C
YJL206C_1
YER184C
PDR1
Gene
Z_Shift_K_Gem
Z_lm_K_Gem
Z_Shift_K_Cyt
Z_lm_K_Cyt
Z_Shift_L_Gem
Z_lm_L_Gem
Z_Shift_L_Cyt
Z_lm_L_Cyt

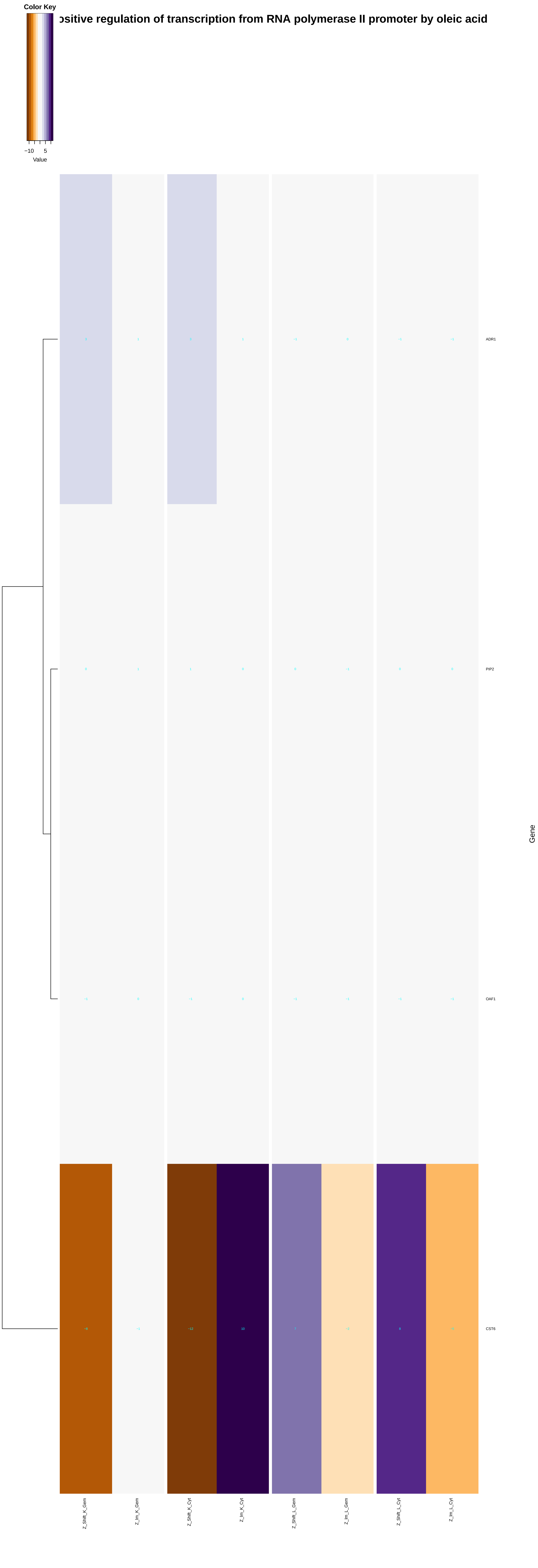
ositive regulation of transcription from RNA polymerase II promoter by oleic acid
Color Key
−10
5
Value
ADR1
PIP2
OAF1
CST6
Gene
Z_Shift_K_Gem
Z_Im_K_Gem
Z_Shift_K_Cyt
Z_Im_K_Cyt
Z_Shift_L_Gem
Z_Im_L_Gem
Z_Shift_L_Cyt
Z_Im_L_Cyt

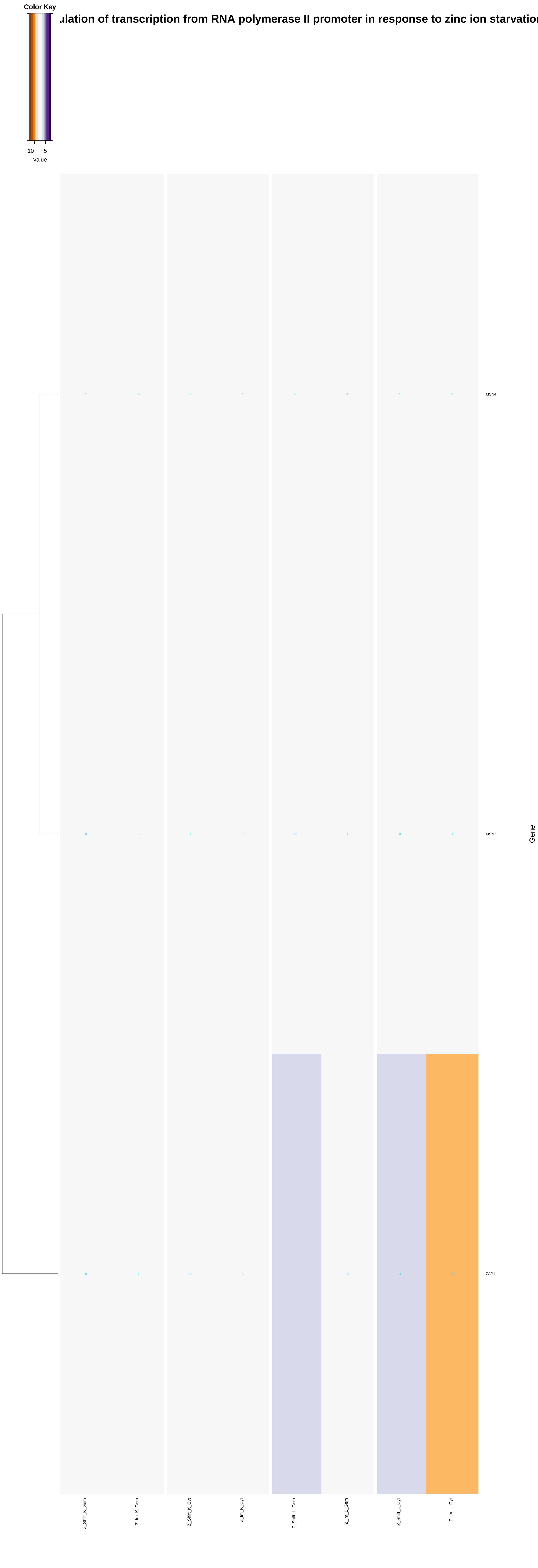

ulation of transcription from RNA polymerase II promoter in response to zinc ion starvatio
Color Key
−10
5
Value
MSN4
MSN2
ZAP1
Gene
Z_Shift_K_Gem
Z_Im_K_Gem
Z_Shift_K_Cyt
Z_Im_K_Cyt
Z_Shift_L_Gem
Z_Im_L_Gem
Z_Shift_L_Cyt
Z_Im_L_Cyt

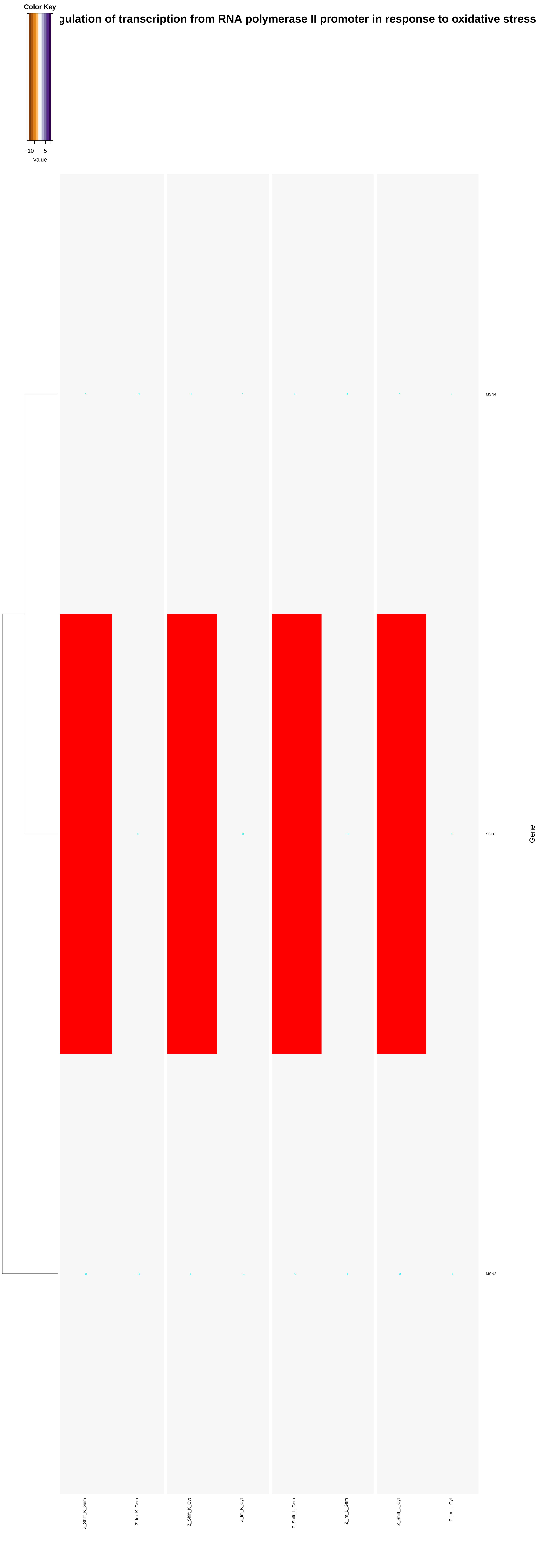
gulation of transcription from RNA polymerase II promoter in response to oxidative stress
Color Key
−10
5
Value
MSN4
SOD1
MSN2
Gene
Z_Shift_K_Gem
Z_Im_K_Gem
Z_Shift_K_Cyt
Z_Im_K_Cyt
Z_Shift_L_Gem
Z_Im_L_Gem
Z_Shift_L_Cyt
Z_Im_L_Cyt

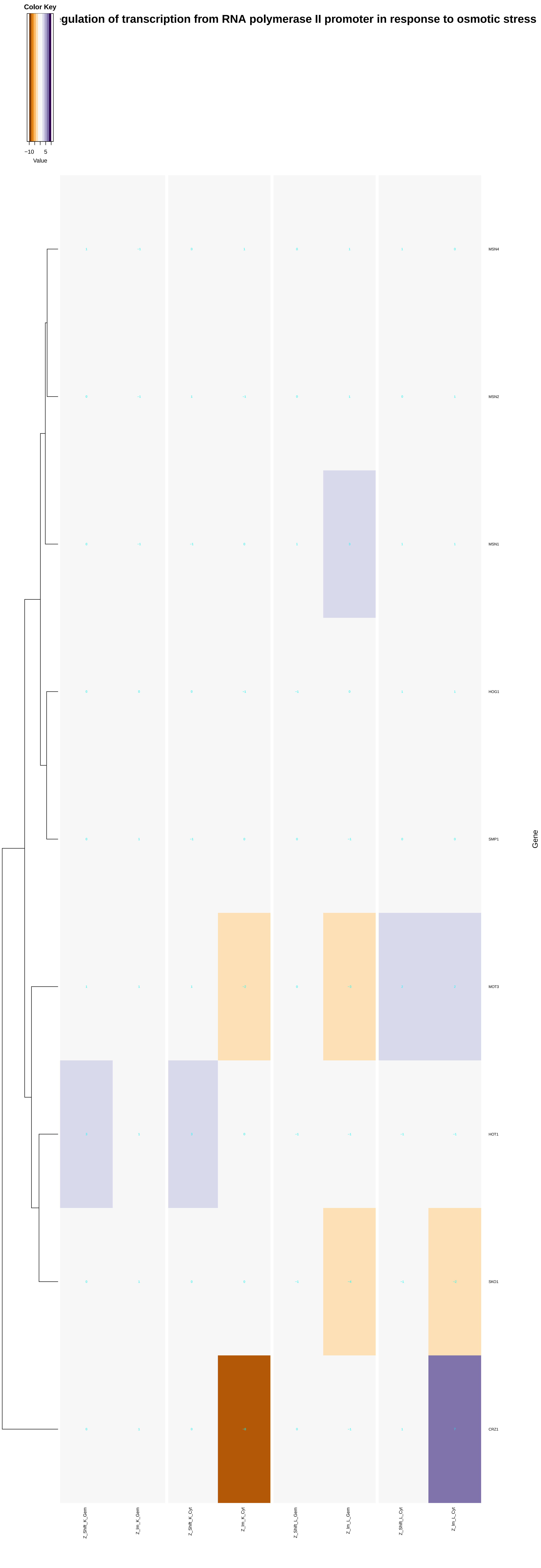

Color Key
−10
5
Value
gulation of transcription from RNA polymerase II promoter in response to osmotic stress
MSN4
MSN2
MSN1
HOG1
SMP1
MOT3
HOT1
SKO1
CRZ1
Gene
Z_Shift_K_Gem
Z_Im_K_Gem
Z_Shift_K_Cyt
Z_Im_K_Cyt
Z_Shift_L_Gem
Z_Im_L_Gem
Z_Shift_L_Cyt
Z_Im_L_Cyt

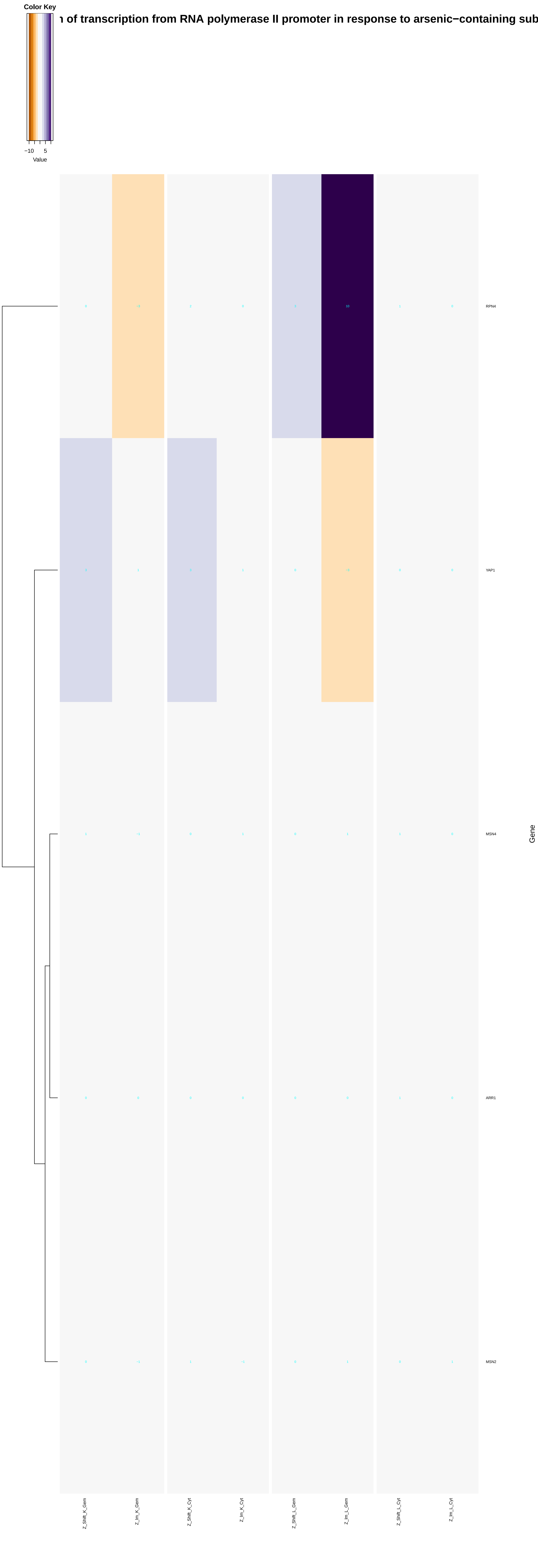
n of transcription from RNA polymerase II promoter in response to arsenic−containing sub
Color Key
−10
5
Value
RPN4
YAP1
MSN4
ARR1
MSN2
Gene
Z_Shift_K_Gem
Z_Im_K_Gem
Z_Shift_K_Cyt
Z_Im_K_Cyt
Z_Shift_L_Gem
Z_Im_L_Gem
Z_Shift_L_Cyt
Z_Im_L_Cyt

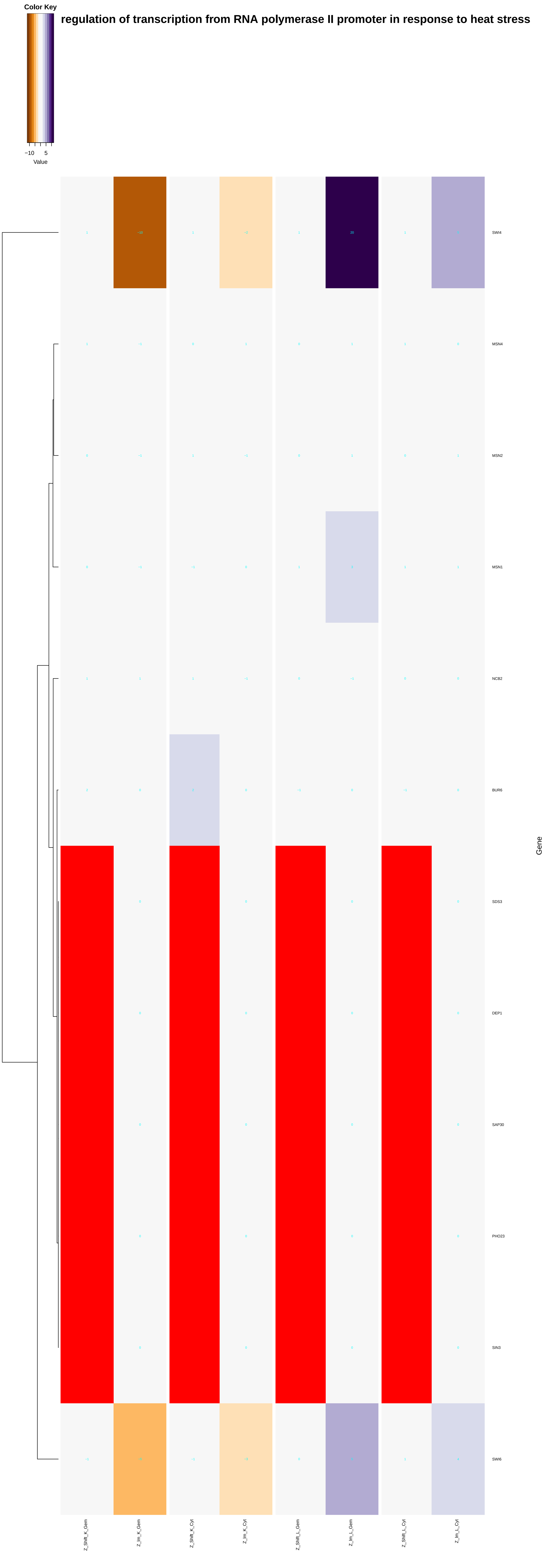
Color Key
−10
5
Value
regulation of transcription from RNA polymerase II promoter in response to heat stress
SWI4
MSN4
MSN2
MSN1
NCB2
BUR6
SDS3
DEP1
SAP30
PHO23
SIN3
SWI6
Gene
Z_Shift_K_Gem
Z_Im_K_Gem
Z_Shift_K_Cyt
Z_Im_K_Cyt
Z_Shift_L_Gem
Z_Im_L_Gem
Z_Shift_L_Cyt
Z_Im_L_Cyt

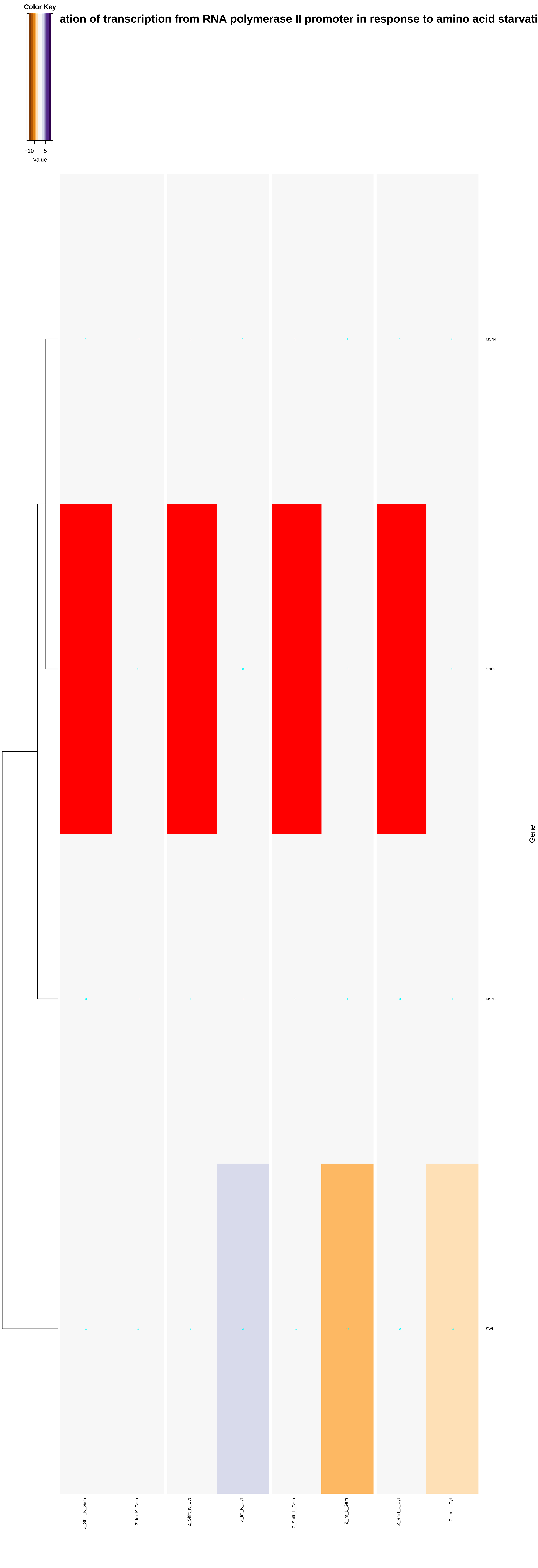
Color Key
−10
5
Value
ation of transcription from RNA polymerase II promoter in response to amino acid starvati
MSN4
SNF2
MSN2
SWI1
Gene
Z_Shift_K_Gem
Z_Im_K_Gem
Z_Shift_K_Cyt
Z_Im_K_Cyt
Z_Shift_L_Gem
Z_Im_L_Gem
Z_Shift_L_Cyt
Z_Im_L_Cyt

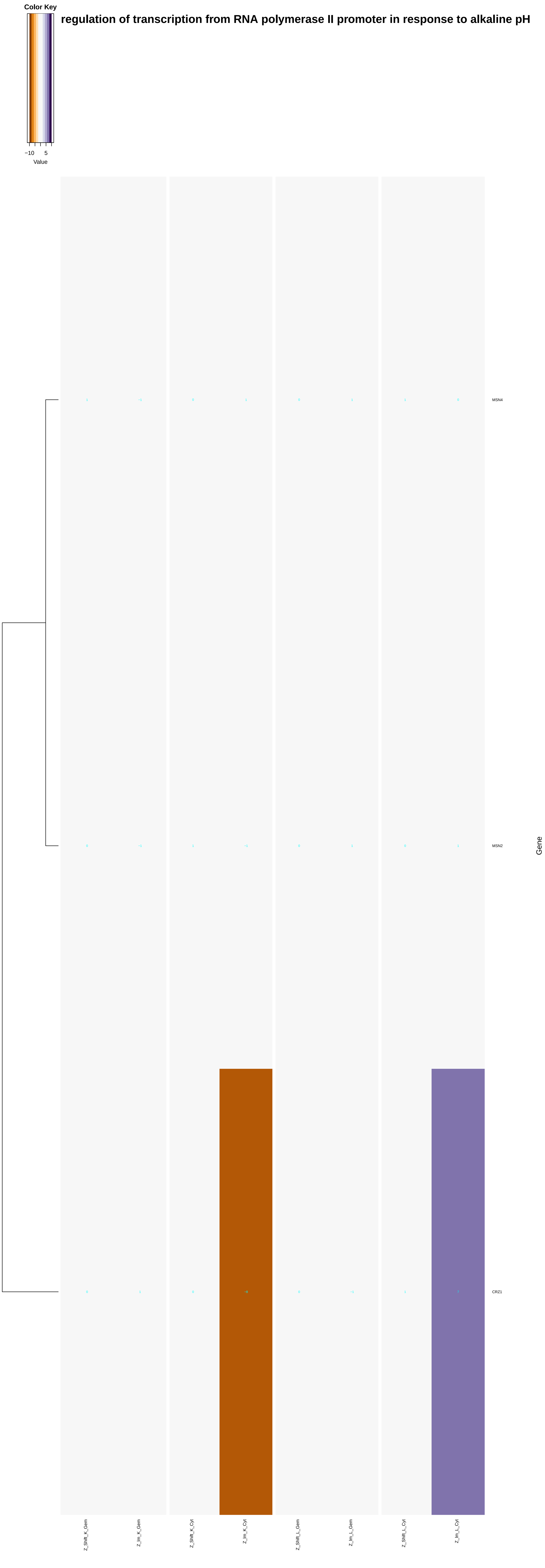

regulation of transcription from RNA polymerase II promoter in response to alkaline pH
Color Key
−10
5
Value
MSN4
MSN2
CRZ1
Gene
Z_Shift_K_Gem
Z_Im_K_Gem
Z_Shift_K_Cyt
Z_Im_K_Cyt
Z_Shift_L_Gem
Z_Im_L_Gem
Z_Shift_L_Cyt
Z_Im_L_Cyt

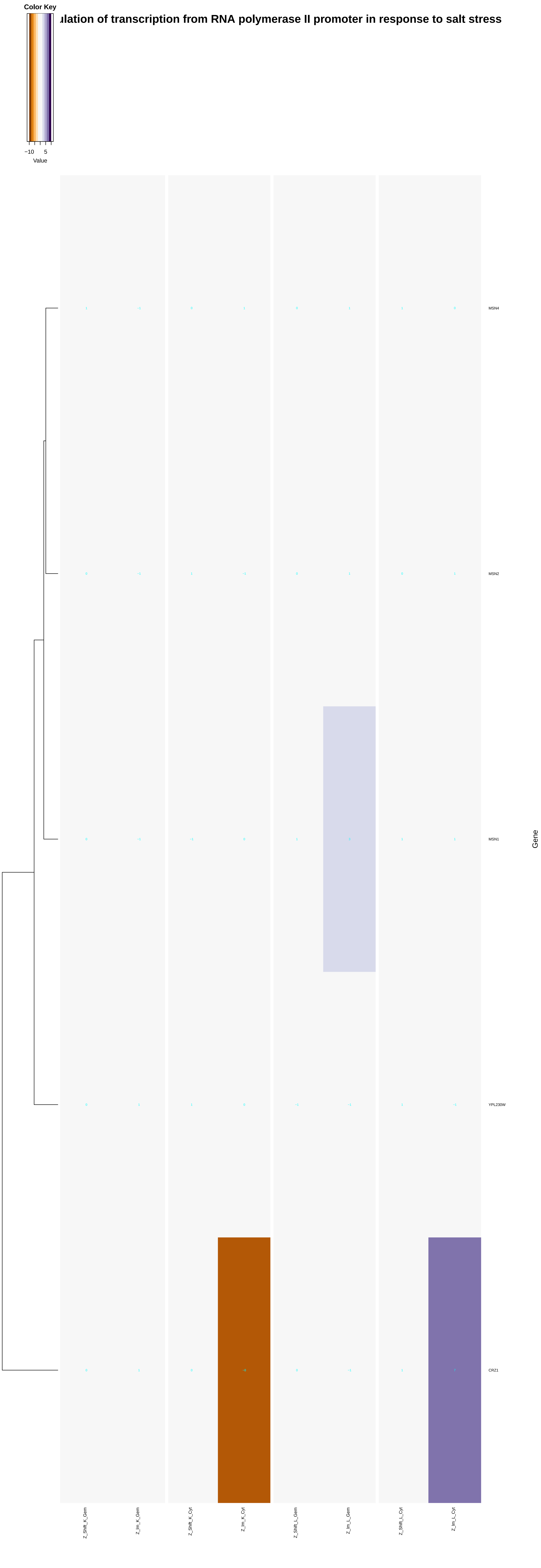
ulation of transcription from RNA polymerase II promoter in response to salt stress
Color Key
−10
5
Value
MSN4
MSN2
MSN1
YPL230W
CRZ1
Gene
Z_Shift_K_Gem
Z_Im_K_Gem
Z_Shift_K_Cyt
Z_Im_K_Cyt
Z_Shift_L_Gem
Z_Im_L_Gem
Z_Shift_L_Cyt
Z_Im_L_Cyt

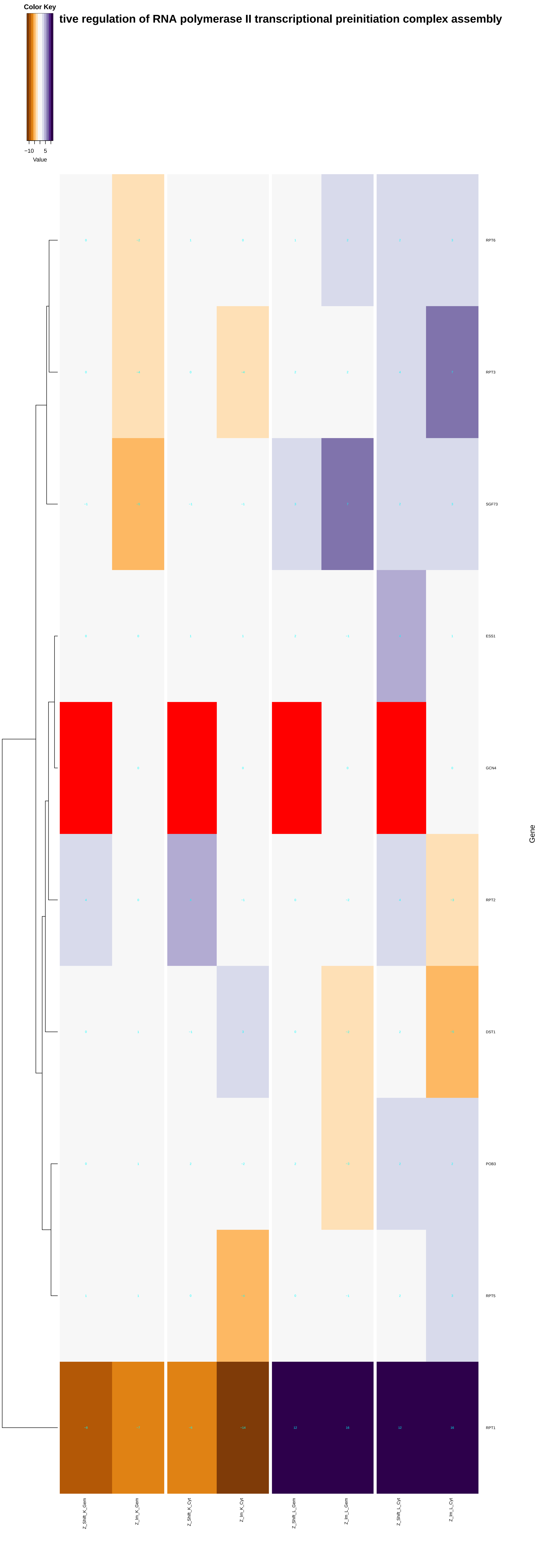

tive regulation of RNA polymerase II transcriptional preinitiation complex assembly
Color Key
−10
5
Value
RPT6
RPT3
SGF73
ESS1
GCN4
RPT2
DST1
POB3
RPT5
RPT1
Gene
Z_Shift_K_Gem
Z_Im_K_Gem
Z_Shift_K_Cyt
Z_Im_K_Cyt
Z_Shift_L_Gem
Z_Im_L_Gem
Z_Shift_L_Cyt
Z_Im_L_Cyt

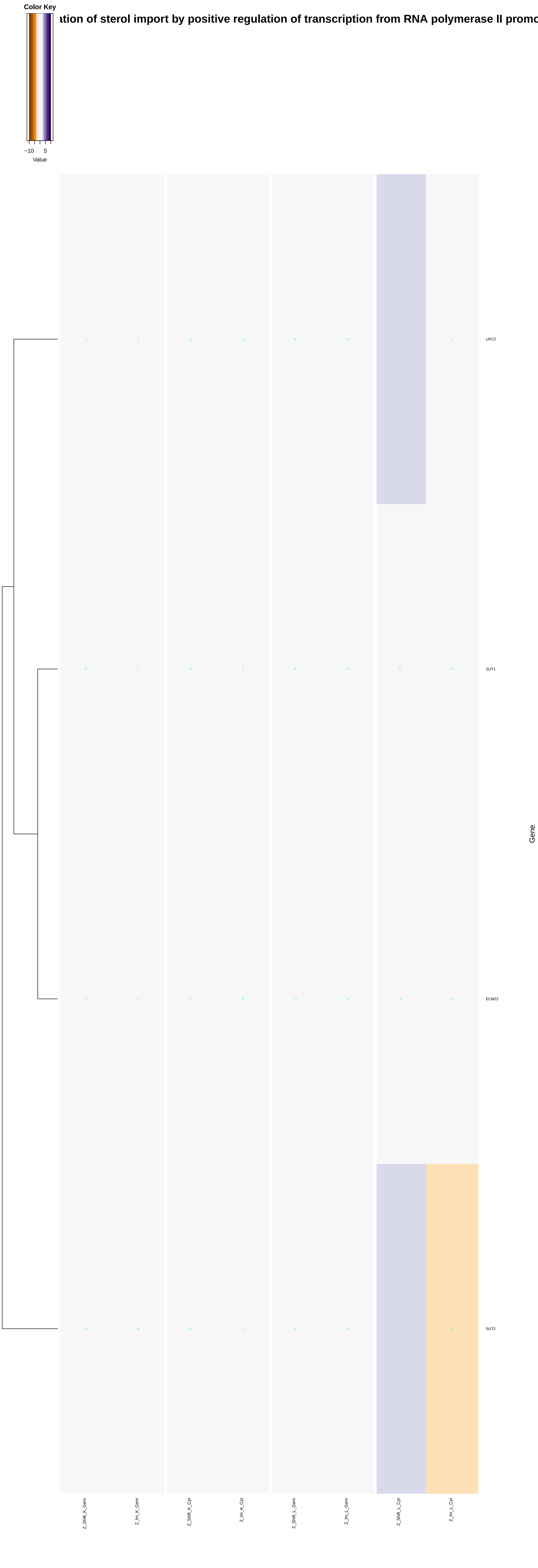
ation of sterol import by positive regulation of transcription from RNA polymerase II promo
Color Key
−10
5
Value
UPC2
SUT1
ECM22
SUT2
Gene
Z_Shift_K_Gem
Z_Im_K_Gem
Z_Shift_K_Cyt
Z_Im_K_Cyt
Z_Shift_L_Gem
Z_Im_L_Gem
Z_Shift_L_Cyt
Z_Im_L_Cyt

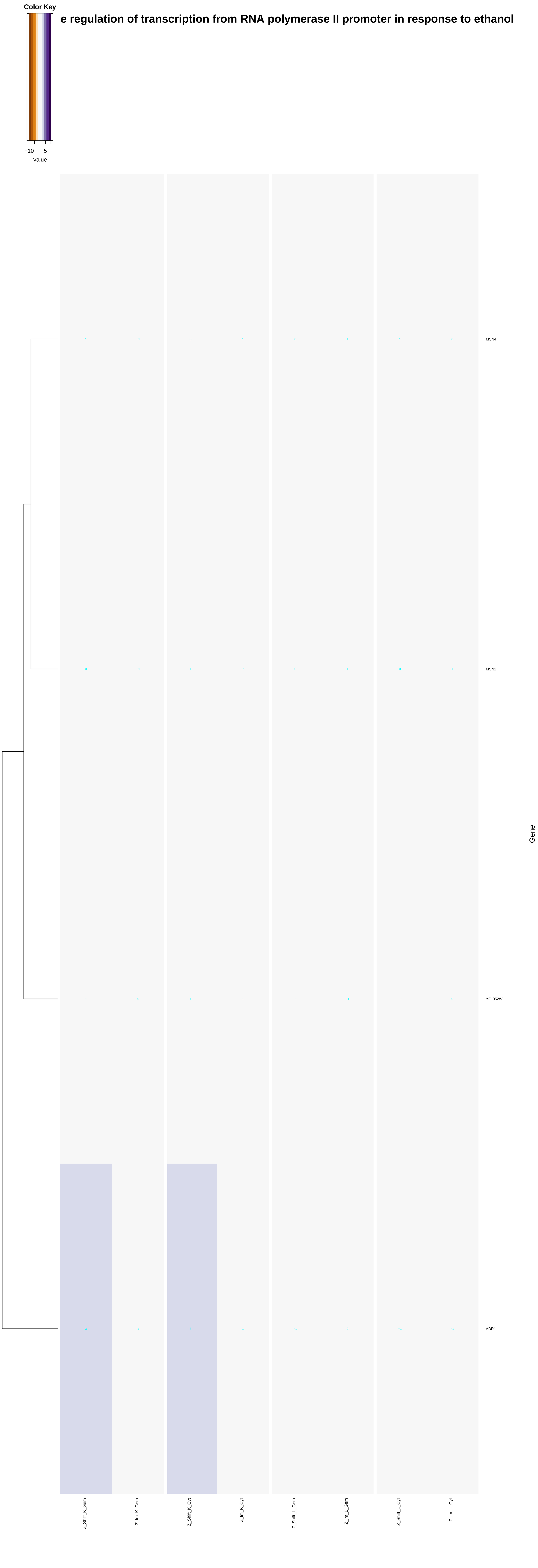
Color Key
−10
5
Value
e regulation of transcription from RNA polymerase II promoter in response to ethanol
MSN4
MSN2
YFL052W
ADR1
Gene
Z_Shift_K_Gem
Z_Im_K_Gem
Z_Shift_K_Cyt
Z_Im_K_Cyt
Z_Shift_L_Gem
Z_Im_L_Gem
Z_Shift_L_Cyt
Z_Im_L_Cyt

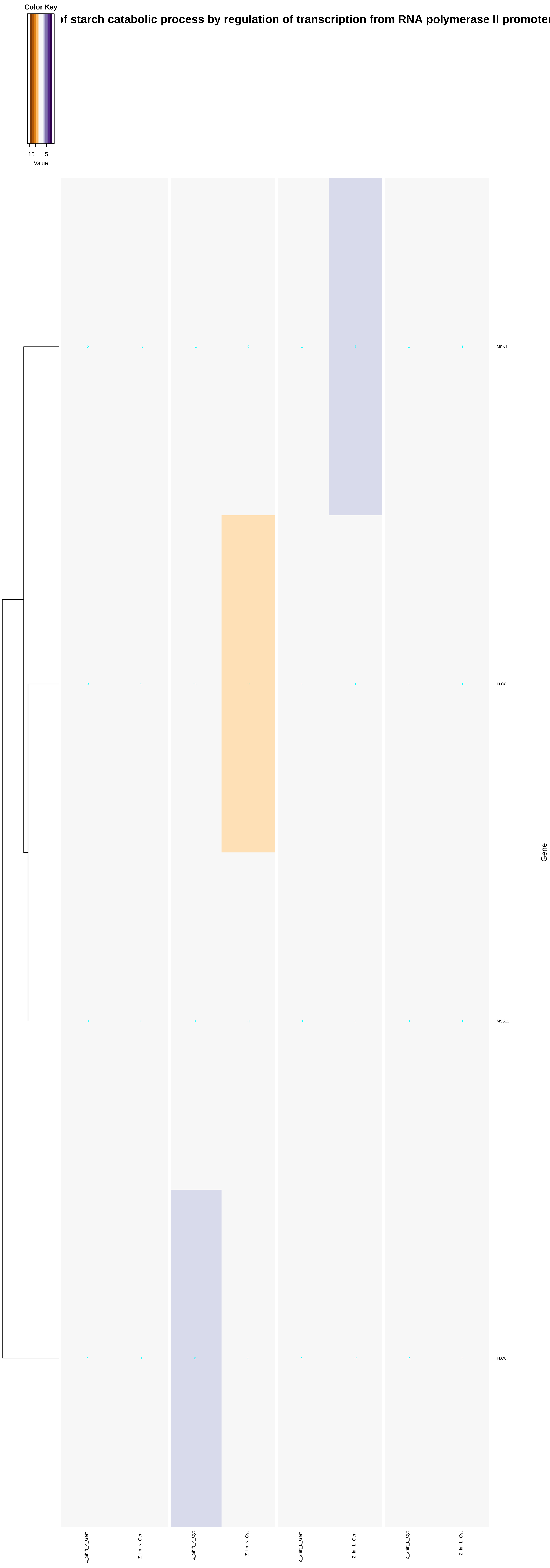
Color Key
−10
5
Value
of starch catabolic process by regulation of transcription from RNA polymerase II promoter
MSN1
FLO8
MSS11
FLO8
Gene
Z_Shift_K_Gem
Z_Im_K_Gem
Z_Shift_K_Cyt
Z_Im_K_Cyt
Z_Shift_L_Gem
Z_Im_L_Gem
Z_Shift_L_Cyt
Z_Im_L_Cyt

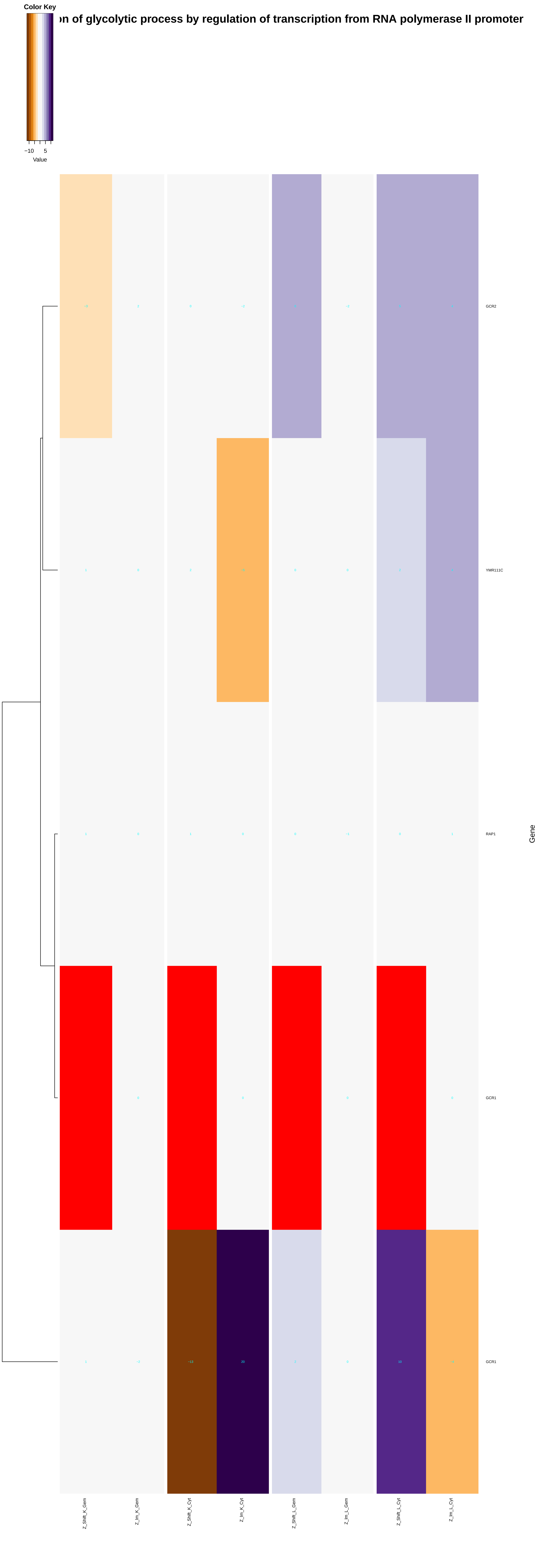
Color Key
−10
5
Value
ɔn of glycolytic process by regulation of transcription from RNA polymerase II promoter
GCR2
YMR111C
RAP1
GCR1
GCR1
Gene
Z_Shift_K_Gem
Z_Im_K_Gem
Z_Shift_K_Cyt
Z_Im_K_Cyt
Z_Shift_L_Gem
Z_Im_L_Gem
Z_Shift_L_Cyt
Z_Im_L_Cyt

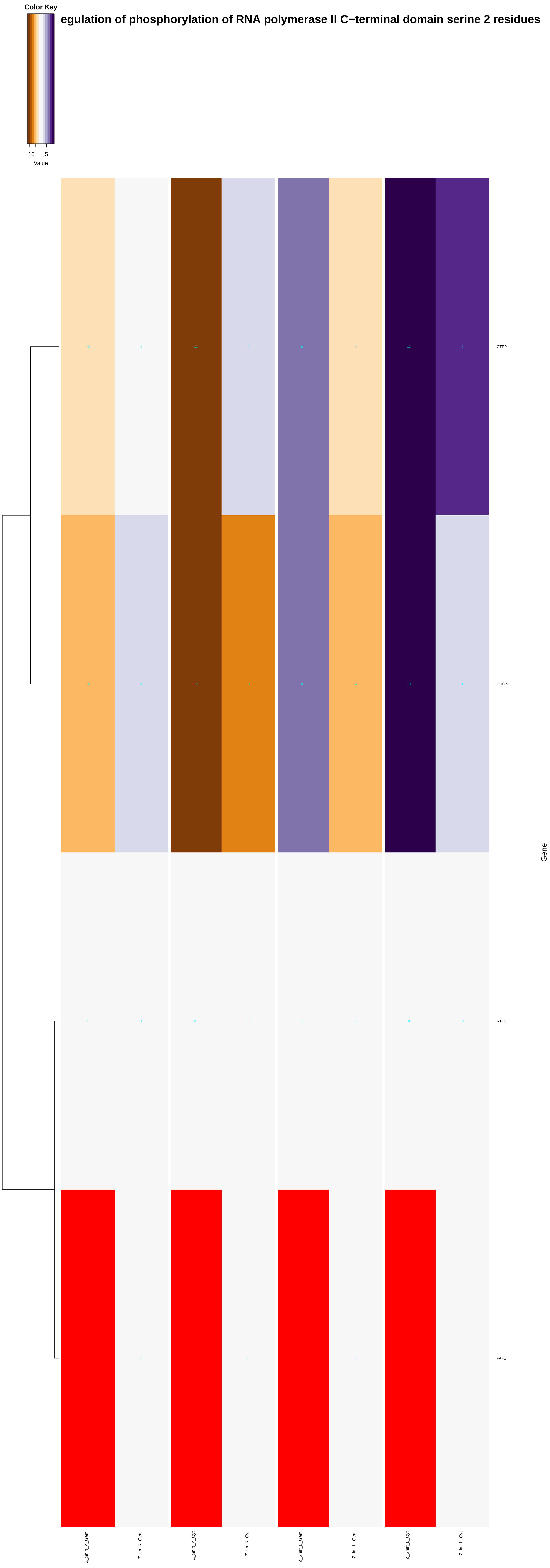
egulation of phosphorylation of RNA polymerase II C−terminal domain serine 2 residues
Color Key
−10
5
Value
−3
2
−12
4
6
−4
12
9
CTR9
−6
4
−15
−7
8
−4
10
4
CDC73
1
1
2
0
−1
0
0
−1
RTF1
0
0
0
0
PAF1
Gene
Z_Shift_K_Gem
Z_Im_K_Gem
Z_Shift_K_Cyt
Z_Im_K_Cyt
Z_Shift_L_Gem
Z_Im_L_Gem
Z_Shift_L_Cyt
Z_Im_L_Cyt

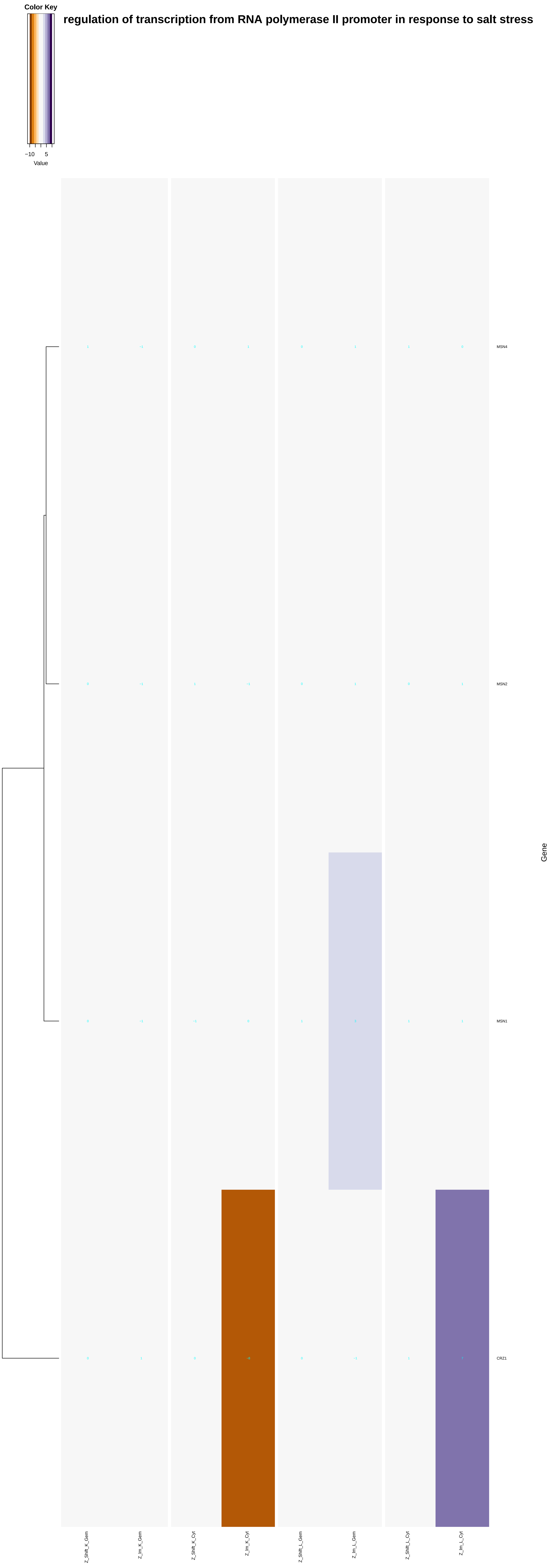

Color Key
−10
5
Value
regulation of transcription from RNA polymerase II promoter in response to salt stress
MSN4
MSN2
MSN1
CRZ1
Gene
Z_Shift_K_Gem
Z_Im_K_Gem
Z_Shift_K_Cyt
Z_Im_K_Cyt
Z_Shift_L_Gem
Z_Im_L_Gem
Z_Shift_L_Cyt
Z_Im_L_Cyt

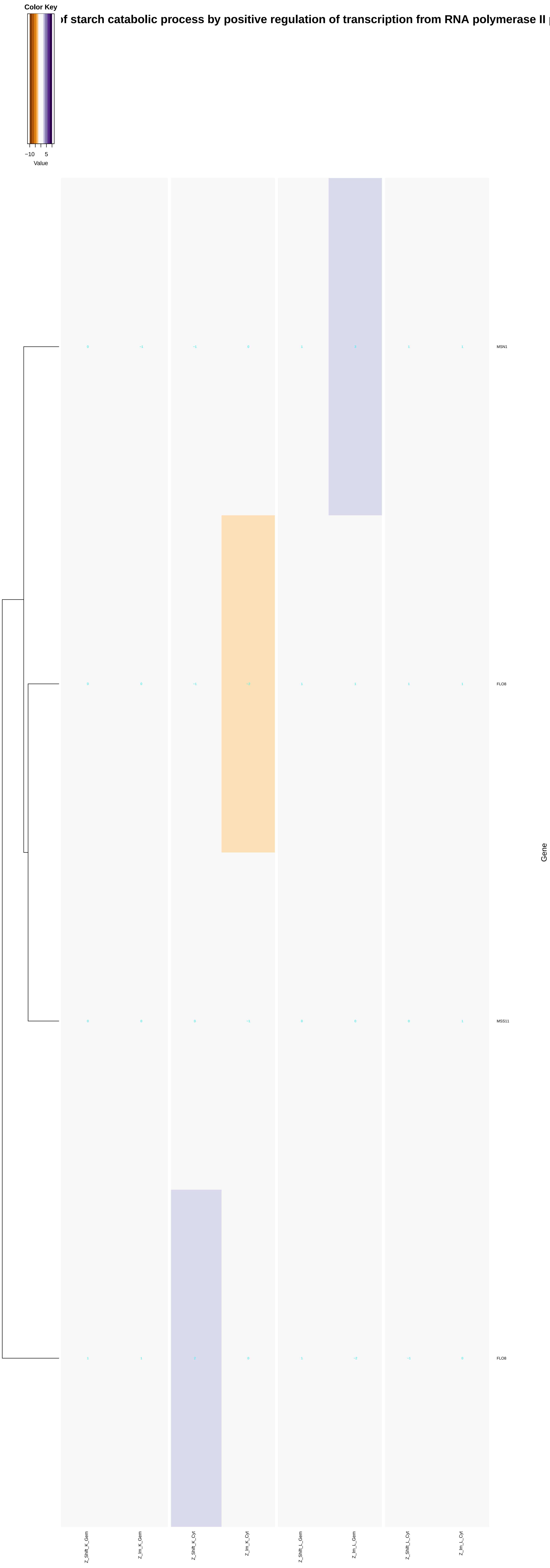

Color Key
−10
5
Value
of starch catabolic process by positive regulation of transcription from RNA polymerase II p
MSN1
FLO8
MSS11
FLO8
Gene
Z_Shift_K_Gem
Z_Im_K_Gem
Z_Shift_K_Cyt
Z_Im_K_Cyt
Z_Shift_L_Gem
Z_Im_L_Gem
Z_Shift_L_Cyt
Z_Im_L_Cyt

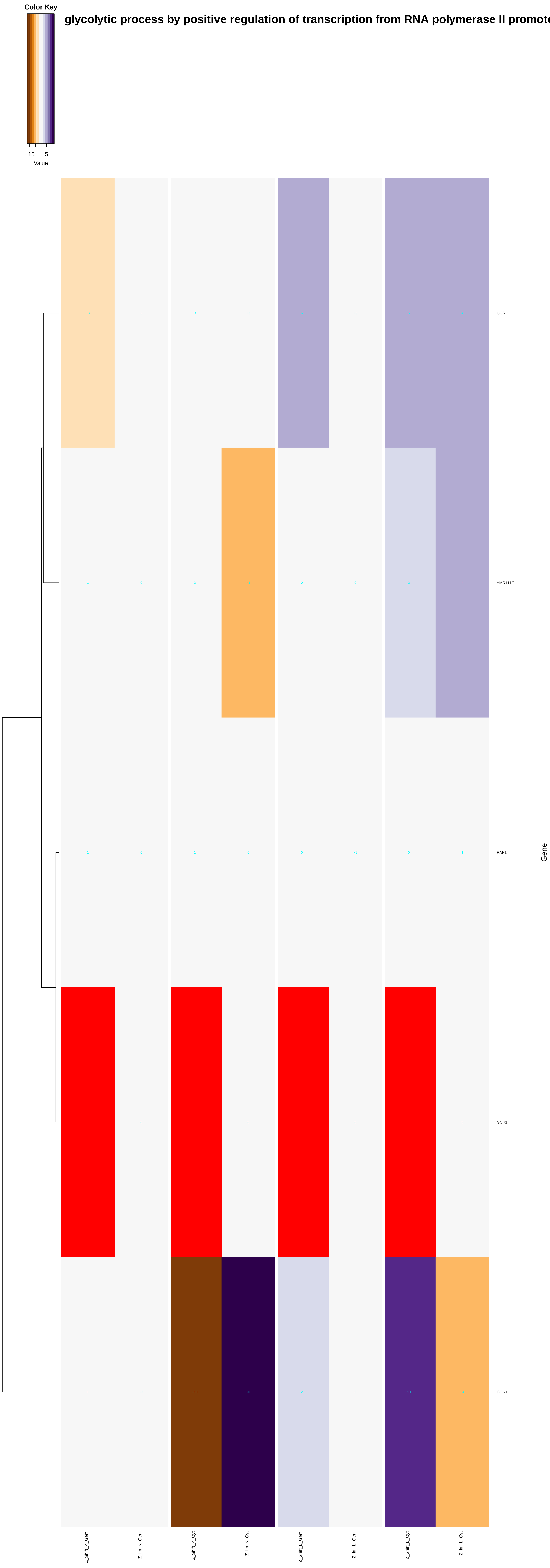
glycolytic process by positive regulation of transcription from RNA polymerase II promote
Color Key
−10
5
Value
GCR2
YMR111C
RAP1
GCR1
GCR1
Gene
Z_Shift_K_Gem
Z_Im_K_Gem
Z_Shift_K_Cyt
Z_Im_K_Cyt
Z_Shift_L_Gem
Z_Im_L_Gem
Z_Shift_L_Cyt
Z_Im_L_Cyt

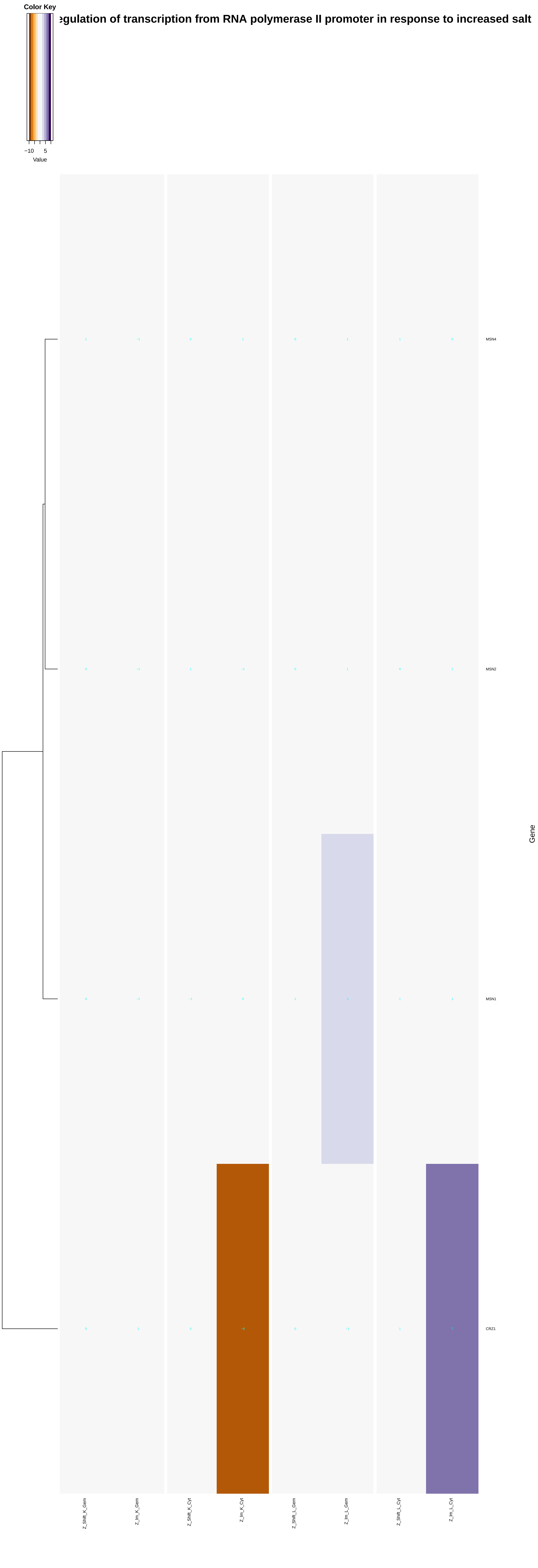

egulation of transcription from RNA polymerase II promoter in response to increased salt
Color Key
−10
5
Value
MSN4
MSN2
MSN1
CRZ1
Gene
Z_Shift_K_Gem
Z_Im_K_Gem
Z_Shift_K_Cyt
Z_Im_K_Cyt
Z_Shift_L_Gem
Z_Im_L_Gem
Z_Shift_L_Cyt
Z_Im_L_Cyt
